# Supplementary material for: Video inoculation against election misinformation across 12 EU nations
Source: Commun Psychol. 2026 Mar 17;4:77. doi: 10.1038/s44271-025-00379-3 (PMC13144327; doi:10.1038/s44271-025-00379-3)
Supplement: Supplementary file 2 — Supplement [file 44271_2025_379_MOESM2_ESM.pdf]

Video inoculation against election misinformation across 12 EU nations

Supplement

BLINDED

Table of Contents

Section 1: Cross protection *5*

Scapegoating videos **5**
Decontextualization content 5
Discrediting content 7

Decontextualization videos **9**
Scapegoating content 9
Discrediting content 11

Discrediting videos **13**
Scapegoating content 13
Decontextualization content 15

Section 2: Interactions *17*

Scapegoating videos **18**
Manipulativeness assessments of manipulative scapegoating content 18
Manipulation discernment of scapegoating content 19
Technique recognition of manipulative scapegoating content 20
Technique recognition of non-manipulative scapegoating content 28
Technique discernment of scapegoating content 30
,Sharing decisions for scapegoating content 36

Decontextualization videos **41**
Manipulativeness assessments of decontextualization content 41
Technique recognition of manipulative decontextualization content 43
Technique recognition of non-manipulative decontextualization content 48
Technique discernment of decontextualization content 54
Willingness to share manipulative decontextualization content 56
Willingness to share non-manipulative decontextualization content 57

Discrediting videos **61**
Manipulativeness assessments of manipulative discrediting content 61
Manipulativeness assessments of non-manipulative discrediting content 63
Manipulation discernment of discrediting content 64
Technique recognition of manipulative discrediting content 68
Technique recognition of non-manipulative discrediting content 73
Technique discernment of discrediting content 74
Willingness to share manipulative discrediting content 76
Willingness to share non-manipulative discrediting content 78
Sharing decisions for the discrediting content 83

Confidence in detecting manipulation **88**
Scapegoating videos 88
Decontextualization videos 90
Discrediting videos 96

Section 3: Effects by Survey *97*

Manipulation discernment **97**

Technique discernment **98**

Sharing decisions **100**

Section 4: Study materials *102*

Item rating task **102**
Manipulative scapegoating content 102
Non-manipulative scapegoating content 103
Manipulative decontextualization content 104
Non-manipulative decontextualization content 106
Manipulative discrediting content 107
Non-manipulative discrediting content 108

Digital literacy scale **109**

Political tolerance scale **110**

Section 5: Comparisons between participants who had seen, had not seen, or who were unsure of whether they had seen the videos ***110***

Scapegoating content **110**
Manipulativeness assessments 110
Technique recognition 111
Willingness to share 111

Decontextualization content **112**
Manipulativeness assessments 112
Technique recognition 112
Willingness to share 112

Discrediting content **113**
Manipulativeness assessments 113
Technique recognition 113
Willingness to share 114

Confidence in detecting manipulation **114**

Section 6: Comparison of intervention effects by video length ***120***

Manipulativeness assessments **120**
Scapegoating 120
Decontextualization 120
Discrediting 120

Technique recognition **120**
Scapegoating 120
Decontextualization 121
Discrediting 121
Willingness to share 121
Scapegoating 121
Decontextualization 121
Discrediting 122

Confidence in detecting relevant manipulation **122**

Section 7: Exploratory multilevel models by individual item variation ***122***

Manipulation discernment **122**
Scapegoating 122
Decontextualization 123
Discrediting 124

Technique discernment **124**
Scapegoating 124
Decontextualization 125
Discrediting 125

Sharing discernment **126**
Scapegoating 126
Decontextualization 126

Discrediting 126

Section 8: Violin Plots for Means between Conditions *127*

Manipulativeness assessments **127**

Manipulative scapegoating content 127
Non-Manipulative scapegoating content 127
Manipulation discernment of Scapegoating content 128
Manipulative decontextualization content 128
Non-manipulative decontextualization content 129
Manipulation discernment of Decontextualization content 130
Manipulative Discrediting content 130
Non-manipulative Discrediting content 131
Manipulation discernment of Discrediting content 131

Technique recognition **132**

Manipulative Scapegoating content 132
Non-manipulative Scapegoating content 133
Technique discernment of Scapegoating content 133
Manipulative Decontextualization content 134
Non-manipulative Decontextualization content 134
Technique discernment of Decontextualization content 135
Manipulative Discrediting content 136
Non-manipulative Discrediting content 136
Manipulation discernment of Discrediting content 137

Sharing decisions **137**

Manipulative Scapegoating content 137
Non-manipulative Scapegoating content 138
Sharing discernment of Scapegoating content 139
Manipulative Decontextualization content 139
Non-manipulative Decontextualization content 140
Sharing discernment of Decontextualization content 140
Manipulative Discrediting content 141
Non-manipulative Discrediting content 142
Sharing discernment of Discrediting content 142

Confidence in detecting manipulation **143**

Section 9: Skepticism and Naïveté *143*

Manipulativeness assessments **143**

Technique recognition **144**

Section 1: Cross protection

Here, we report the results for the effects of the three inoculation videos on manipulation discernment, technique discernment, and sharing decisions for unrelated content (i.e., cross-protection). These results are from the same multilevel models reported in the main text.

Scapegoating videos

Decontextualization content

Manipulativeness assessments. The long scapegoating video (vs. control) significantly increased manipulativeness assessments of the manipulative decontextualization content, $t(18{,}300) = 2.74$, $p = .006$, $d = 0.06$, but this effect was non-significant for the short scapegoating video (vs. control), $t(15{,}360) = 0.57$, $p = .569$, $d = -0.02$ (see Figure S1). The effects of the long, $t(18{,}720) = 0.06$, $p = .956$, $d = 0.01$, and short scapegoating videos (vs. control), $t(16{,}530) = 0.25$, $p = .803$, $d = -0.01$, on manipulativeness assessments of the non-manipulative decontextualization content were non-significant (see Figure S1). The effects of the long, $t(17{,}580) = 1.48$, $p = .140$, $d = 0.04$, and short scapegoating videos (vs. control), $t(12{,}760) = 0.05$, $p = .963$, $d = -0.01$, on manipulation discernment of the decontextualization content were non-significant (see Figure S1).

Technique recognition. The long, $t(18{,}720) = 9.26$, $p < .001$, $d = 0.21$, and short scapegoating videos (vs. control), $t(18{,}670) = 4.78$, $p < .001$, $d = 0.17$, significantly increased the technique recognition of manipulative decontextualization content (see Figure S1). The long, $t(18{,}720) = 10.01$, $p < .001$, $d = -0.23$, and short scapegoating videos (vs. control), $t(18{,}600) = 5.62$, $p < .001$, $d = -0.20$, significantly decreased the technique recognition of non-manipulative decontextualization content (see Figure S1). The effects of the long, $t(19{,}440) = 0.97$, $p = .331$, $d = -0.02$, and short scapegoating videos (vs. control), $t(19{,}300) =$

0.75, $p = .456$, $d = -0.03$, on technique discernment of the decontextualization content were non-significant (see Figure S1).

Willingness to share. The effects of the long, $t(18{,}300) = 1.14$, $p = .256$, $d = 0.03$, and short scapegoating videos (vs. control), $t(18{,}260) = 1.03$, $p = .304$, $d = 0.04$, on willingness to share the manipulative decontextualization content were non-significant (see Figure S1). The effects of the long, $t(18{,}720) = 0.90$, $p = .367$, $d = 0.02$, and short scapegoating videos (vs. control), $t(18{,}710) = 0.17$, $p = .862$, $d = 0.01$, on willingness to share the non-manipulative decontextualization content were non-significant (see Figure S1). The effects of the long, $t(17{,}580) = 0.30$, $p = .762$, $d = 0.01$, and short scapegoating videos (vs. control), $t(14{,}330) = 1.30$, $p = .195$, $d = -0.05$, on sharing decisions for the decontextualization content were non-significant (see Figure S1).

Figure S1.

Forest Plot of Unstandardized Beta Coefficients and 95% Confidence Intervals for the Multilevel Model Effects of the Scapegoating Videos on all Decontextualization Outcomes.

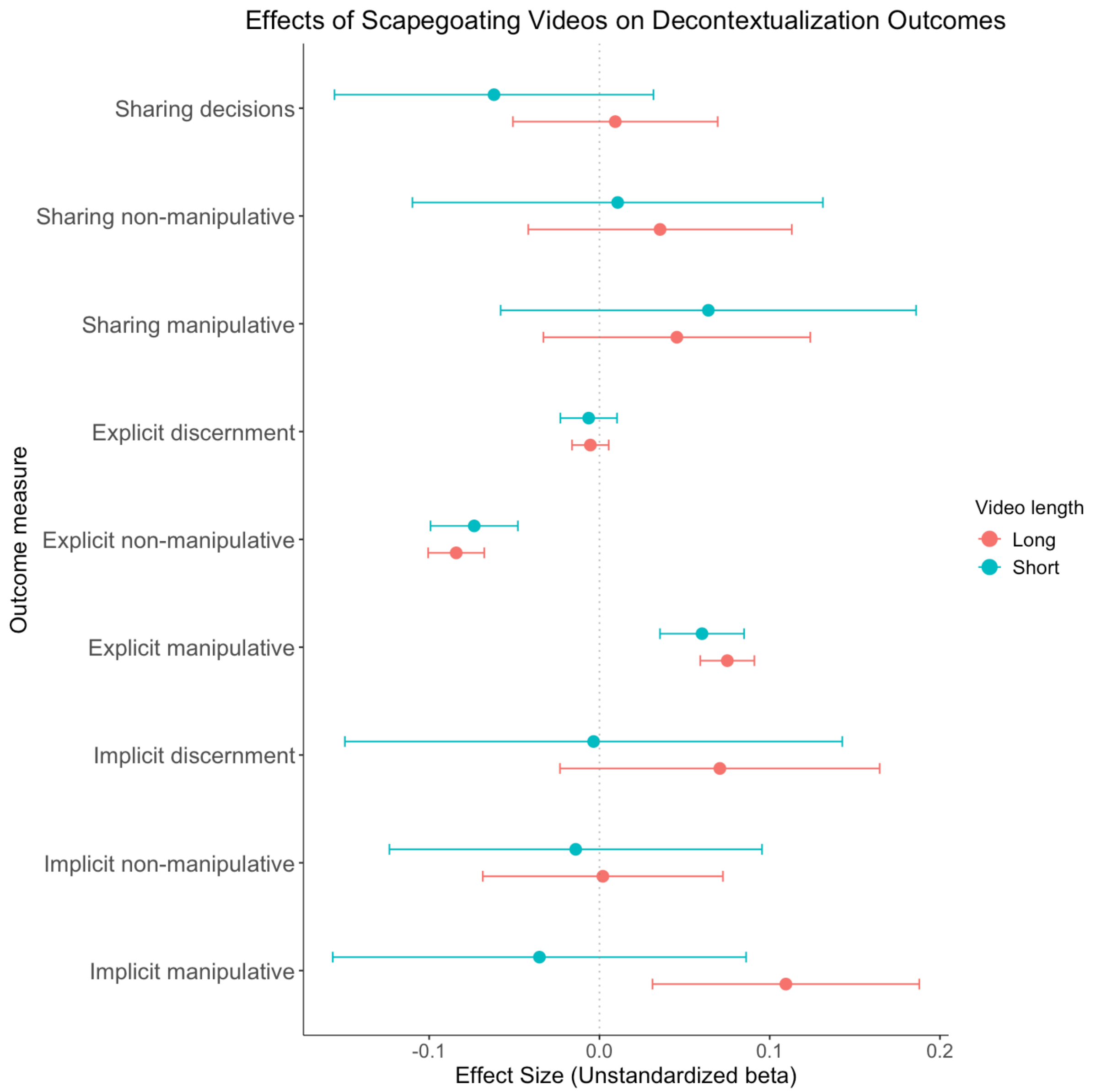

Discrediting content

Manipulativeness assessments. The long scapegoating video (vs. control) significantly increased manipulativeness assessments of the manipulative discrediting content, $t(18{,}270) = 2.01$, $p = .044$, $d = 0.05$ (see Figure S2). However, the effect of the short scapegoating video (vs. control) was non-significant, $t(15{,}990) = 0.57$, $p = .571$, $d = -0.02$ (see Figure S2). The effects of the long, $t(18{,}640) = 0.56$, $p = .574$, $d = -0.01$, and short scapegoating videos (vs. control), $t(16{,}160) = 0.73$, $p = .468$, $d = -0.03$, on manipulativeness assessments of the non-manipulative discrediting content were non-significant (see Figure S2). The effects of the long, $t(17{,}490) = 1.52$, $p = .128$, $d = 0.04$, and short scapegoating

videos (vs. control), $t(15,980) = 0.25$, $p = .806$, $d = 0.01$, on manipulation discernment of the discrediting content were non-significant (see Figure S2).

Technique recognition. The long, $t(18,660) = 6.12$, $p < .001$, $d = 0.14$, and short scapegoating videos (vs. control), $t(18,640) = 4.57$, $p < .001$, $d = 0.16$, significantly increased technique recognition of the manipulative discrediting content (see Figure S2). The long, $t(17,880) = 6.50$, $p < .001$, $d = -0.15$, and short scapegoating videos (vs. control), $t(16,360) = 3.01$, $p = .003$, $d = -0.11$, significantly decreased technique recognition of the non-manipulative discrediting content (see Figure S2). The effects of the long, $t(19,420) = 1.18$, $p = .238$, $d = -0.03$, and short scapegoating videos (vs. control), $t(19,390) = 0.51$, $p = .609$, $d = -0.02$, on technique discernment of the discrediting content were non-significant (see Figure S2).

Willingness to share. The long, $t(18,270) = 2.34$, $p = .019$, $d = 0.05$, and short scapegoating videos (vs. control), $t(18,250) = 2.01$, $p = .044$, $d = 0.07$, significantly increased willingness to share manipulative discrediting content (see Figure S2). The long scapegoating video (vs. control) significantly increased willingness to share non-manipulative discrediting content, $t(18,640) = 2.90$, $p = .004$, $d = 0.07$ (see Figure S2). However, the effect of the short scapegoating video (vs. control) on willingness to share non-manipulative discrediting content was non-significant, $t(18,630) = 0.95$, $p = .345$, $d = 0.03$ (see Figure S2). The effects of the long, $t(17,490) = 0.08$, $p = .936$, $d = 0.01$, and short scapegoating videos (vs. control), $t(13,230) = 0.31$, $p = .755$, $d = -0.01$, on sharing decisions for the discrediting content were non-significant (see Figure S2).

Figure S2.

Forest Plot of Unstandardized Beta Coefficients and 95% Confidence Intervals for the Multilevel Model Effects of the Scapegoating Videos on all Discrediting Outcomes.

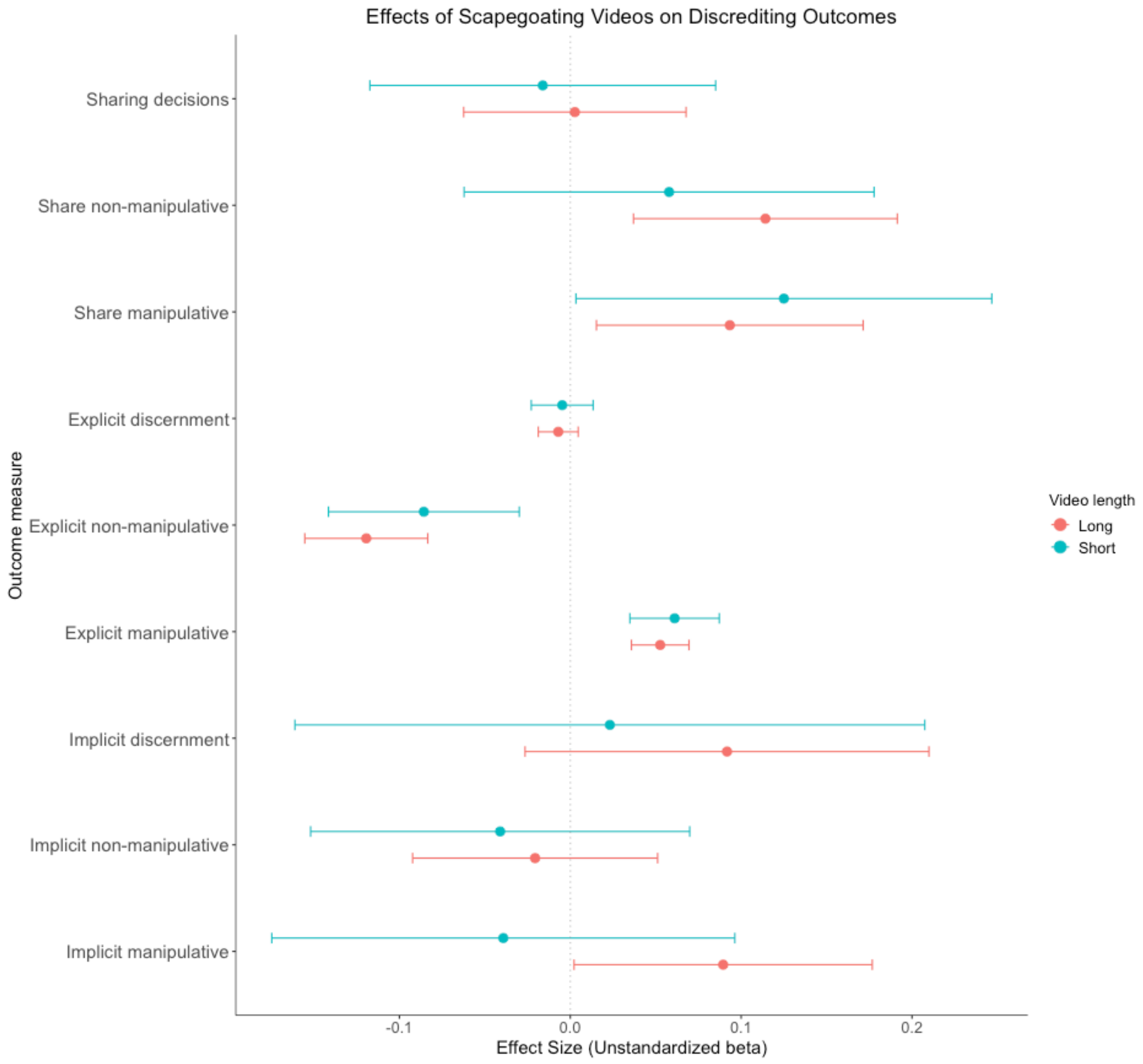

Decontextualization videos

Scapegoating content

Manipulativeness assessments. The long decontextualization video (vs. control) significantly increased manipulativeness assessments of the manipulative scapegoating content, $t(18{,}270) = 3.51$, $p < .001$, $d = 0.08$ (see Figure S3). However, the effect of the short decontextualization video was non-significant, $t(12{,}340) = 1.72$, $p = .085$, $d = 0.06$ (see Figure S3). The long decontextualization video (vs. control) significantly decreased manipulativeness assessments of the non-manipulative scapegoating content, $t(18{,}640) = 2.36$, $p = .018$, $d = -0.05$ (see Figure S3). However, the effect of the short decontextualization video was non-significant, $t(18{,}080) = 0.64$, $p = .525$, $d = -0.02$ (see Figure S3). The long,

$t(17{,}530) = 4.24$, $p < .001$, $d = 0.10$, and short decontextualization videos (vs. control), $t(16{,}530) = 2.13$, $p = .033$, $d = 0.08$, significantly increased manipulation discernment of the scapegoating content (see Figure S3).

Technique recognition. The long, $t(18{,}640) = 6.23$, $p < .001$, $d = 0.14$, and short decontextualization videos (vs. control), $t(18{,}640) = 5.41$, $p < .001$, $d = 0.19$, significantly increased technique recognition of the manipulative scapegoating content (see Figure S3). The long, $t(18{,}640) = 6.82$, $p < .001$, $d = -0.16$, and short decontextualization videos (vs. control), $t(18{,}000) = 4.18$, $p < .001$, $d = -0.15$, significantly decreased technique recognition of the non-manipulative scapegoating content (see Figure S3). The effects of the long, $t(19{,}380) = 0.77$, $p = .439$, $d = -0.02$, and short decontextualization videos (vs. control), $t(19{,}340) = 0.15$, $p = .880$, $d = -0.01$, on technique discernment of the scapegoating content were non-significant (see Figure S3).

Willingness to share. The effects of the long, $t(18{,}270) = 1.84$, $p = .066$, $d = -0.04$, and short decontextualization videos (vs. control), $t(18{,}260) = 0.12$, $p = .906$, $d = 0.01$, on willingness to share the manipulative scapegoating content were non-significant (see Figure S3). The long decontextualization video (vs. control) significantly decreased willingness to share the non-manipulative scapegoating content, $t(18{,}640) = 2.00$, $p = .046$, $d = -0.05$ (see Figure S3). However, the effect of the short decontextualization video was non-significant, $t(18{,}630) = 0.21$, $p = .834$, $d = 0.01$ (see Figure S3). The effects of the long, $t(17{,}530) = 0.01$, $p = .991$, $d = 0.01$, and short decontextualization videos (vs. control), $t(13{,}100) = 0.12$, $p = .903$, $d = -0.01$, on sharing decisions for the scapegoating content were non-significant (see Figure S3).

Figure S3.

Forest Plot of Unstandardized Beta Coefficients and 95% Confidence Intervals for the Multilevel Model Effects of the Decontextualization Videos on all Scapegoating Outcomes.

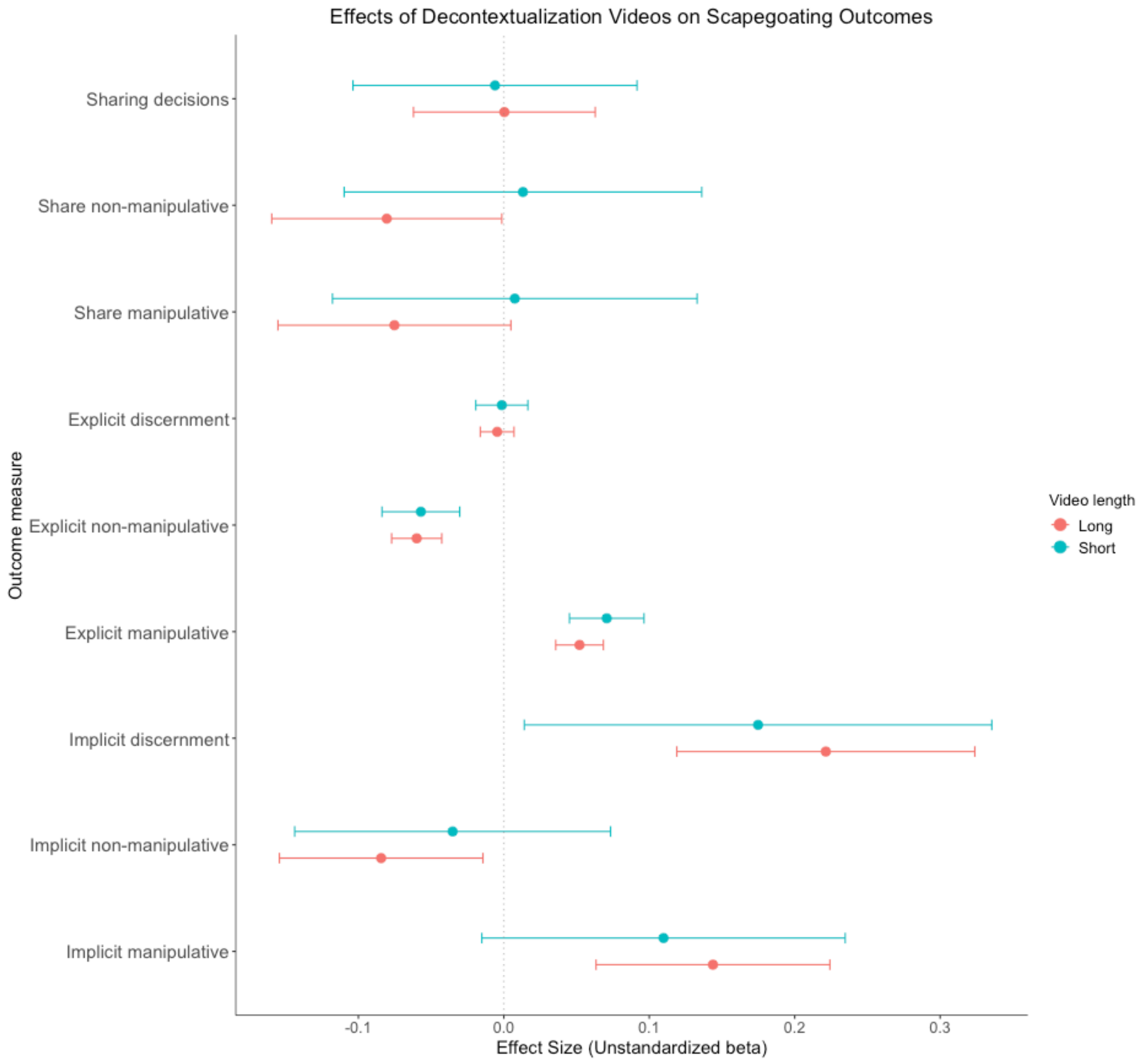

Discrediting content

Manipulativeness assessments. The long decontextualization video (vs. control) significantly increased manipulativeness assessments of the manipulative discrediting content, $t(18{,}270) = 4.74$, $p < .001$, $d = 0.11$ (see Figure S4). However, the effect of the short decontextualization video (vs. control) was non-significant, $t(16{,}000) = 0.74$, $p = .459$, $d = 0.03$ (see Figure S4). The effects of the long, $t(18{,}640) = 0.60$, $p = .549$, $d = -0.02$, and short decontextualization videos (vs. control), $t(16{,}190) = 1.55$, $p = .121$, $d = -0.06$, on manipulativeness assessments of the non-manipulative discrediting content were non-significant (see Figure S4). The long decontextualization video (vs. control), $t(17{,}490) = 3.89$, $p < .001$, $d = 0.09$, significantly increased manipulation discernment of the discrediting

content (see Figure S4). However, the effect of the short decontextualization video (vs. control), $t(16{,}000) = 1.72$, $p = .085$, $d = 0.06$, on manipulation discernment of the discrediting content was non-significant (see Figure S4).

Technique recognition. The long, $t(18{,}660) = 7.60$, $p < .001$, $d = 0.18$, and short decontextualization videos (vs. control), $t(18{,}640) = 5.04$, $p < .001$, $d = 0.18$, significantly increased technique recognition of the manipulative discrediting content (see Figure S4). The long, $t(17{,}880) = 5.59$, $p < .001$, $d = -0.13$, and short decontextualization videos (vs. control), $t(16{,}370) = 3.02$, $p = .003$, $d = -0.11$, significantly decreased technique recognition of the non-manipulative discrediting content (see Figure S4). The effects of the long, $t(19{,}420) = 0.27$, $p = .791$, $d = 0.01$, and short decontextualization videos (vs. control), $t(19{,}390) = 0.30$, $p = .763$, $d = 0.01$, on technique discernment of the discrediting content were non-significant (see Figure S4).

Willingness to share. The effects of the long, $t(18{,}270) = 0.37$, $p = .714$, $d = -0.01$, and short decontextualization videos (vs. control), $t(18{,}250) = 1.18$, $p = .237$, $d = 0.04$, on willingness to share manipulative discrediting content were non-significant (see Figure S4). The effects of the long, $t(18{,}270) = 0.37$, $p = .714$, $d = -0.01$, and short decontextualization videos (vs. control), $t(18{,}250) = 1.18$, $p = .237$, $d = 0.02$, on willingness to share non-manipulative discrediting content were non-significant (see Figure S4). The effects of the long, $t(17{,}490) = 0.16$, $p = .872$, $d = 0.01$, and short decontextualization videos (vs. control), $t(13{,}280) = 0.12$, $p = .907$, $d = -0.01$, on sharing decisions for the discrediting content were non-significant (see Figure S4).

Figure S4.

Forest Plot of Unstandardized Beta Coefficients and 95% Confidence Intervals for the Multilevel Model Effects of the Decontextualization Videos on all Discrediting Outcomes.

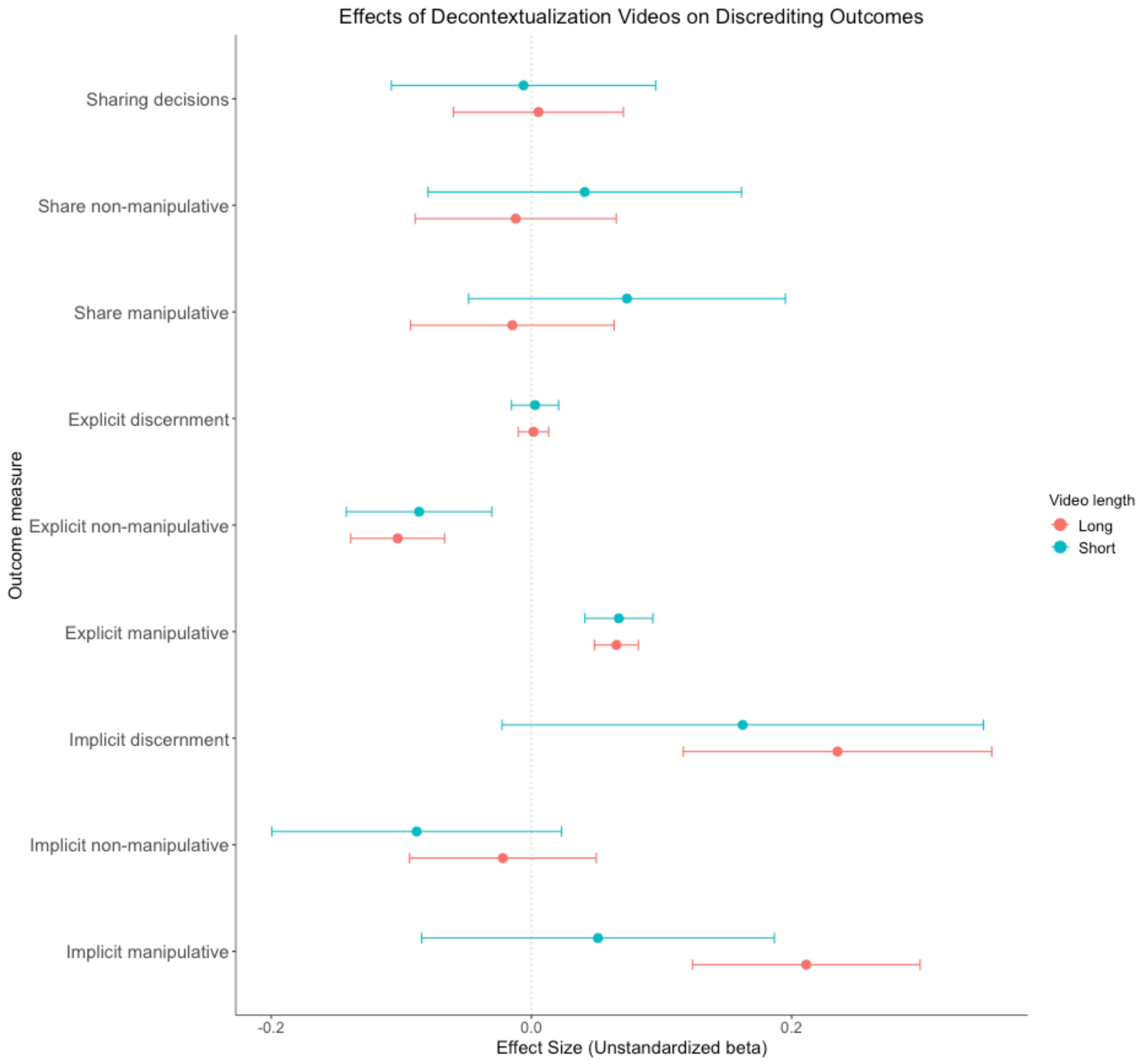

Discrediting videos

Scapegoating content

Manipulativeness assessments. The long discrediting video (vs. control) significantly increased manipulativeness assessments of the manipulative scapegoating content, $t(18{,}270) = 2.44$, $p = .015$, $d = 0.06$ (see Figure S5). However, the effect of the short discrediting video was non-significant, $t(12{,}340) = 0.23$, $p = .818$, $d = -0.01$ (see Figure S5). The long, $t(18{,}640) = 2.69$, $p = .007$, $d = -0.06$, and short discrediting videos (vs. control), $t(18{,}090) = 2.13$, $p = .033$, $d = -0.08$, significantly decreased manipulativeness assessments of the non-manipulative scapegoating content (see Figure S5). The long discrediting video (vs. control) significantly increased manipulation discernment of the scapegoating

content, $t(17{,}530) = 3.52$, $p < .001$, $d = 0.08$ (see Figure S5). However, the effect of the short discrediting video (vs. control) was non-significant, $t(16{,}530) = 1.57$, $p = .116$, $d = 0.06$ (see Figure S5).

Technique recognition. The long, $t(18{,}640) = 7.50$, $p < .001$, $d = 0.17$, and short discrediting videos (vs. control), $t(18{,}640) = 4.99$, $p < .001$, $d = 0.18$, significantly increased technique recognition of the manipulative scapegoating content (see Figure S5). The long, $t(18{,}640) = 3.16$, $p = .002$, $d = -0.07$, and short discrediting videos (vs. control), $t(18{,}010) = 1.37$, $p = .172$, $d = -0.05$, significantly decreased technique recognition of the non-manipulative scapegoating content (see Figure S5). The long, $t(19{,}380) = 2.57$, $p = .010$, $d = 0.06$, and short discrediting videos (vs. control), $t(19{,}340) = 2.24$, $p = .025$, $d = 0.08$, significantly increased technique discernment of the scapegoating content (see Figure S5).

Willingness to share. The effects of the long, $t(18{,}270) = 1.12$, $p = .261$, $d = -0.03$, and short discrediting videos (vs. control), $t(18{,}260) = 0.46$, $p = .649$, $d = 0.02$, on willingness to share the manipulative scapegoating content were non-significant (see Figure S5). The effects of the long, $t(18{,}640) = 0.60$, $p = .552$, $d = 0.01$, and short discrediting videos (vs. control), $t(18{,}630) = 0.25$, $p = .800$, $d = -0.01$, on willingness to share the non-manipulative scapegoating content were non-significant (see Figure S5). The long discrediting video significantly increased sharing decisions for the scapegoating content, $t(17{,}530) = 2.57$, $p = .010$, $d = 0.06$ (see Figure S5). However, the effect of the short discrediting video was non-significant, $t(13{,}120) = 0.88$, $p = .377$, $d = -0.03$ (see Figure S5).

Figure S5.

Forest Plot of Unstandardized Beta Coefficients and 95% Confidence Intervals for the Multilevel Model Effects of the Discrediting Videos on all Scapegoating Outcomes.

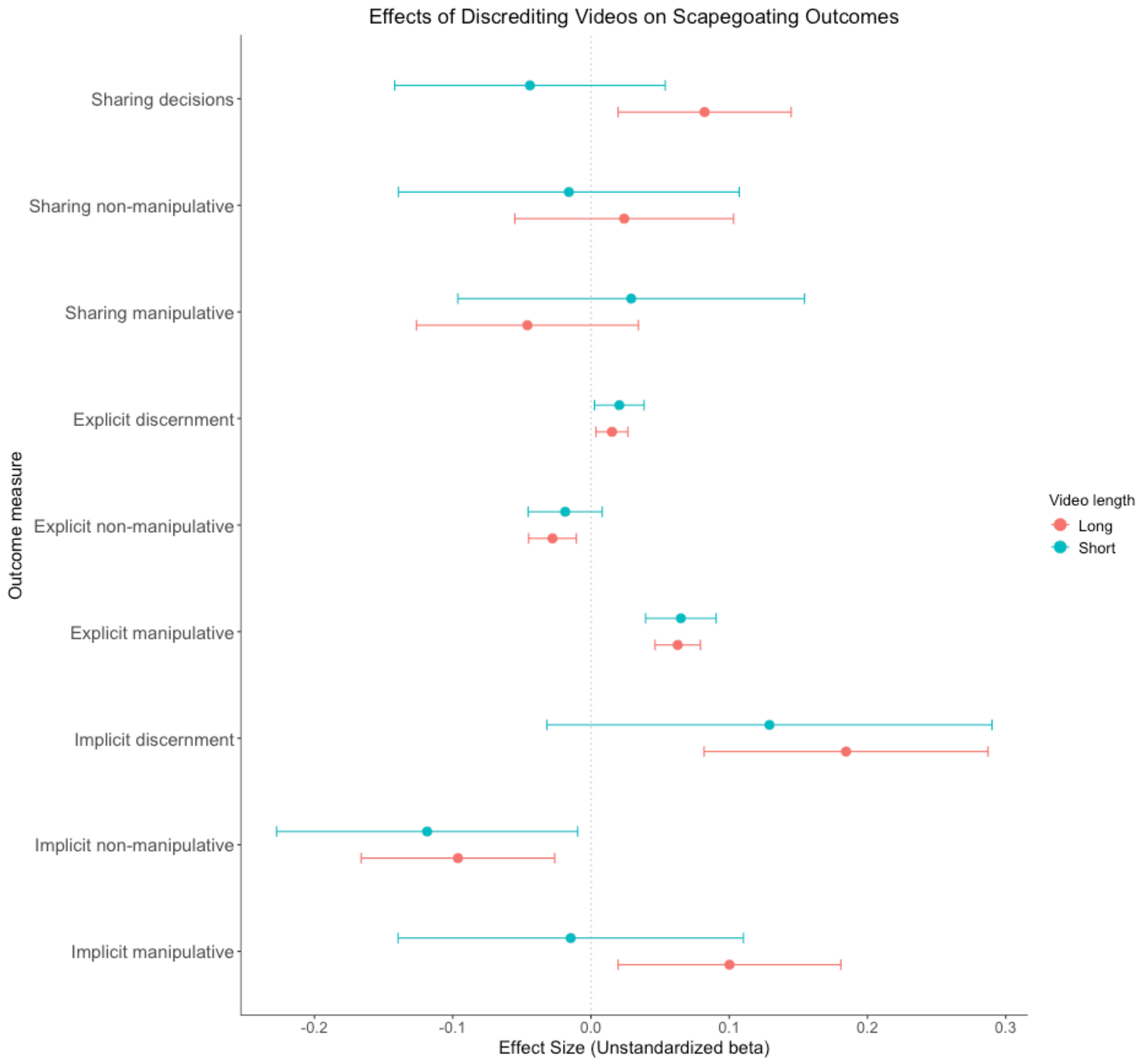

Decontextualization content

Manipulativeness assessments. The effects of the long, *t*(18,300) = 1.07, *p* = .287, *d* = 0.02, and short discrediting videos (vs. control), *t*(15,430) = 0.49, *p* = .622, *d* = -0.02, on manipulativeness assessments of the manipulative decontextualization content were non-significant (see Figure S6). The effects of the long, *t*(18,720) = 0.94, *p* = .350, *d* = -0.02, and short discrediting videos (vs. control), *t*(16,510) = 1.33, *p* = .183, *d* = -0.05, on manipulativeness assessments of the non-manipulative decontextualization content were non-significant (see Figure S6). The effects of the long, *t*(17,580) = 0.94, *p* = .348, *d* = 0.02, and short discrediting videos (vs. control), *t*(12,810) = 0.53, *p* = .596, *d* = 0.02, on manipulation discernment of the decontextualization content were non-significant (see Figure S6).

Technique recognition. The long, $t(18{,}720) = 6.94$, $p < .001$, $d = 0.16$, and short discrediting videos (vs. control), $t(18{,}680) = 4.08$, $p < .001$, $d = 0.15$, significantly increased the technique recognition of manipulative decontextualization content (see Figure S6). The long, $t(18{,}720) = 7.16$, $p < .001$, $d = -0.16$, and short discrediting videos (vs. control), $t(18{,}590) = 2.36$, $p = .018$, $d = -0.08$, significantly decreased the technique recognition of non-manipulative decontextualization content (see Figure S6). The effects of the long, $t(19{,}440) = 0.38$, $p = .703$, $d = -0.01$, and short discrediting videos (vs. control), $t(19{,}300) = 0.81$, $p = .418$, $d = 0.03$, on technique discernment of the decontextualization content were non-significant (see Figure S6).

Willingness to share. The effects of the long, $t(18{,}300) = 1.41$, $p = .158$, $d = -0.03$, and short discrediting videos (vs. control), $t(18{,}260) = 0.14$, $p = .886$, $d = -0.01$, on willingness to share the manipulative decontextualization content were non-significant (see Figure S6). The effects of the long, $t(18{,}720) = 0.42$, $p = .676$, $d = 0.01$, and short discrediting videos (vs. control), $t(18{,}710) = 0.13$, $p = .895$, $d = -0.01$, on willingness to share the non-manipulative decontextualization content were non-significant (see Figure S6). The positive effect of the long discrediting video (vs. control) on sharing decisions for the decontextualization content was significant, $t(17{,}580) = 2.47$, $p = .014$, $d = 0.06$, but the effect of the short discrediting video (vs. control) was non-significant, $t(14{,}370) = 0.35$, $p = .725$, $d = -0.01$ (see Figure S6).

Figure S6.

Forest Plot of Unstandardized Beta Coefficients and 95% Confidence Intervals for the Multilevel Model Effects of the Discrediting Videos on all Decontextualization Outcomes.

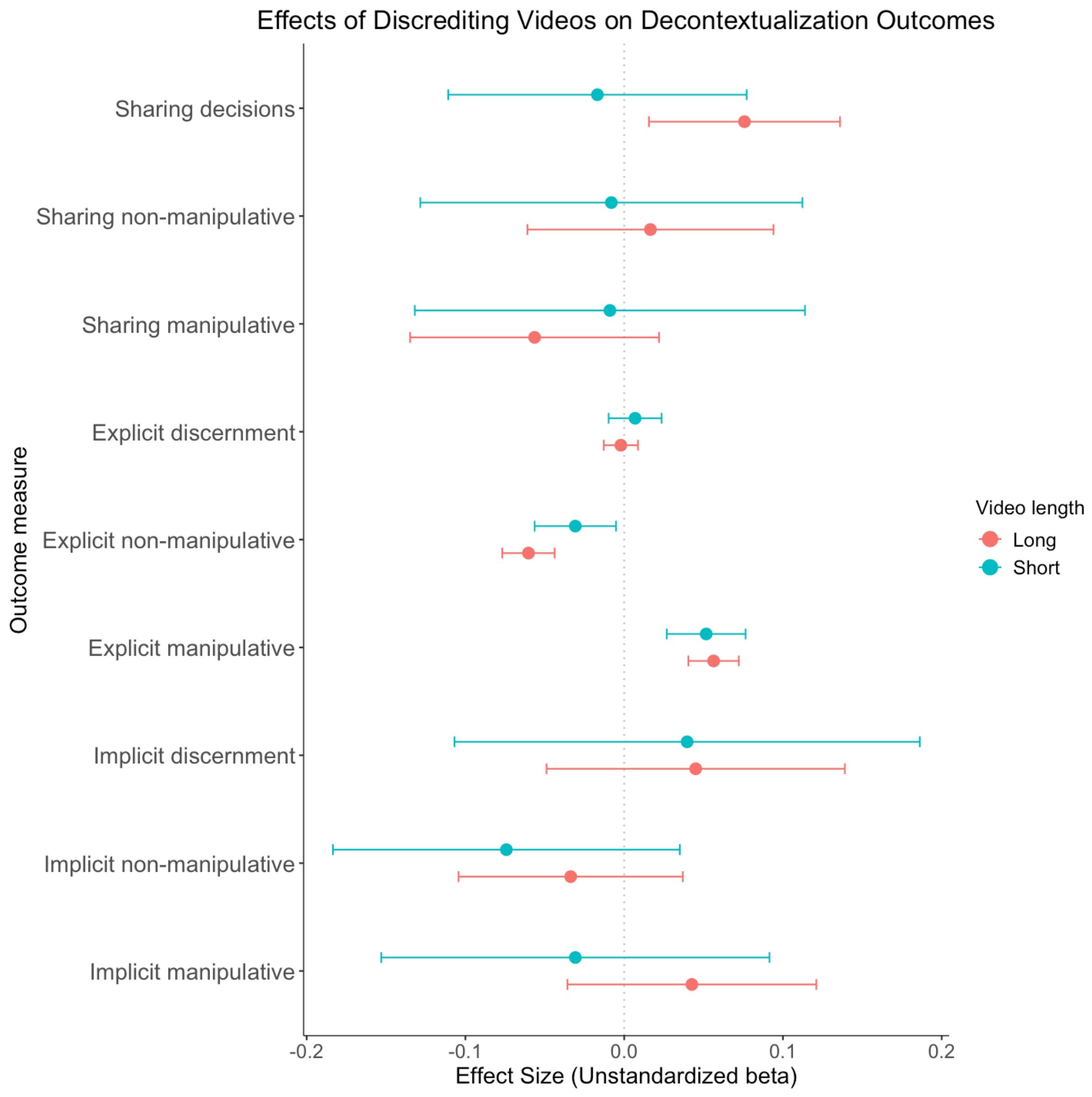

Section 2: Interactions

To test interaction effects, a series of multilevel models were conducted with participants nested within surveys to ensure convergence. Our moderator variables included general manipulation discernment ability, digital literacy, political tolerance, political ideology, WEIRDness of the sample nation (longitude, education index, industrialization index, GDP per capita, and democratic index), voting behavior in the June 2024 EU elections, age, gender, and educational attainment. In each respective model, interaction terms between the respective moderator variable and experimental conditions were included

as predictors of the respective outcome variable. For readability, only significant interaction effects for significant main effects on relevant content are reported here. Please see OSF repository for full data analysis: https://osf.io/tkymv/?view_only=5d902ce84daf4823bb466201de5605e3.

Scapegoating videos

Manipulativeness assessments of manipulative scapegoating content

There was a significant interaction between the long scapegoating video (vs. control) and gender when predicting manipulativeness assessments of the manipulative scapegoating content, b = -0.18, SE = 0.08, t(18,210) = 2.29, p = .022, such that the effect was positive and significant among women, b = 0.29, SE = 0.06, p < .001, but not among men, b = 0.10, SE = 0.06, p = .999 (see Figure S7).

Figure S7.

Simple Slopes Plot of the Interaction Effect between the Long Scapegoating Video (vs. Control) and Gender when Predicting Manipulativeness Assessments of the Manipulative Scapegoating Content.

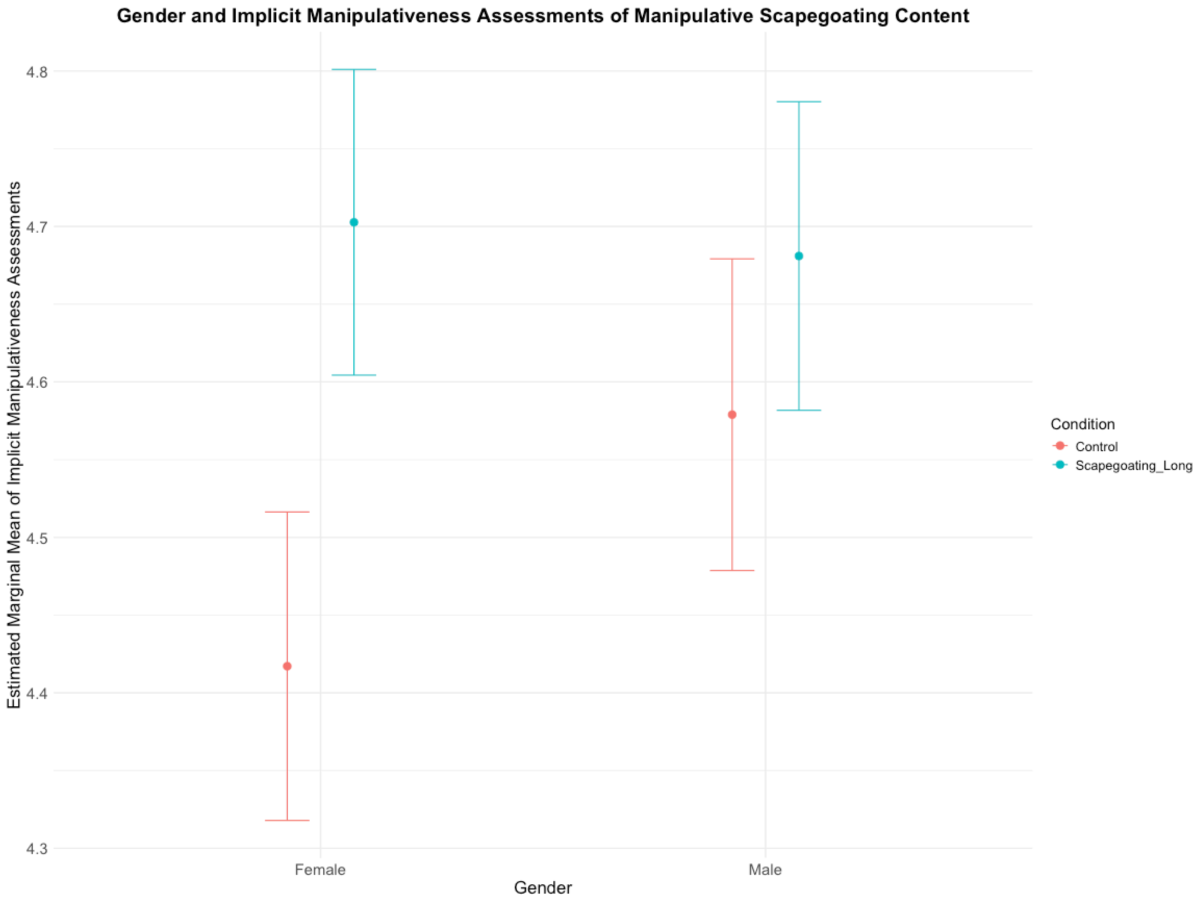

Manipulation discernment of scapegoating content

There was a significant interaction between the long scapegoating video (vs. control) and political tolerance when predicting manipulation discernment of the scapegoating content, $b = 0.11$, $SE = 0.05$, $t(17{,}270) = 2.17$, $p = .030$, such that the effect was only positive and significant at higher, $b = 0.30$, $SE = 0.07$, $p = .004$, and more moderate levels of political tolerance, $b = 0.16$, $SE = 0.05$, $p = .027$, but not at lower levels, $b = 0.05$, $SE = 0.07$, $p = .999$ (see Figure S8). There was also a significant interaction between the long scapegoating video (vs. control) and intentions to share the video within one's social network when predicting manipulation discernment of the scapegoating content, $b = 0.09$, $SE = 0.03$, $t(17{,}200) = 3.29$, $p < .001$, such that the effect was only positive and significant at higher, $b = 0.39$, $SE = 0.08$, $p < .001$, and more moderate levels of intentions to share the video, $b = 0.21$, $SE = 0.05$, $p < .001$, but not at lower levels, $b = 0.03$, $SE = 0.06$, $p = .999$ (see Figure S8).

Figure S8.

Simple Slopes Plot of the Interaction Effects between the Long Scapegoating Video (vs. Control) and both Political Tolerance (Right) and Intentions to Share the Video within One's Social Network (Right) when Predicting Manipulation Discernment of the Scapegoating Content.

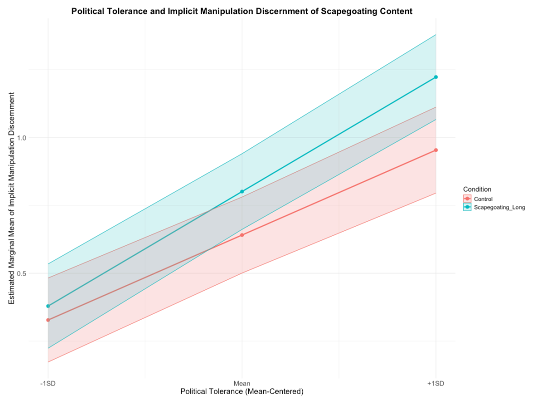

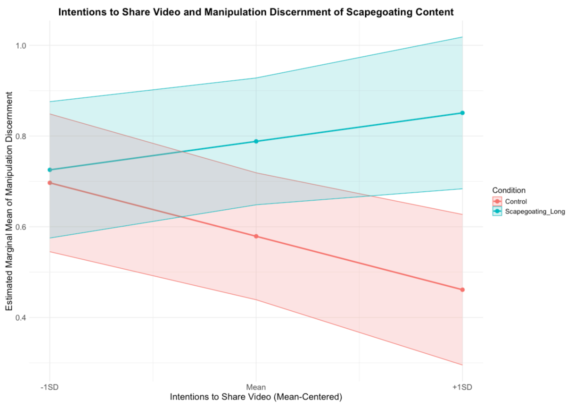

Technique recognition of manipulative scapegoating content

There was a significant interaction between the short scapegoating video (vs. control) and general manipulation discernment ability when predicting technique recognition of the manipulative scapegoating content, b = -0.01, SE = 0.01, t(18,630) = 1.96, p = .050, such that the positive significant effect was strongest at lower levels of general manipulation discernment ability, b = 0.10, SE = 0.02, p < .001, weaker at moderate levels, b = 0.08, SE = 0.01, p < .001, and weakest at higher levels, b = 0.05, SE = 0.02, p = .021 (see Figure S9).

Figure S9.

Simple Slopes Plot of the Interaction Effect between the Short Scapegoating Video (vs. Control) and General Manipulation Discernment Ability when Predicting Technique Recognition of the Manipulative Scapegoating Content.

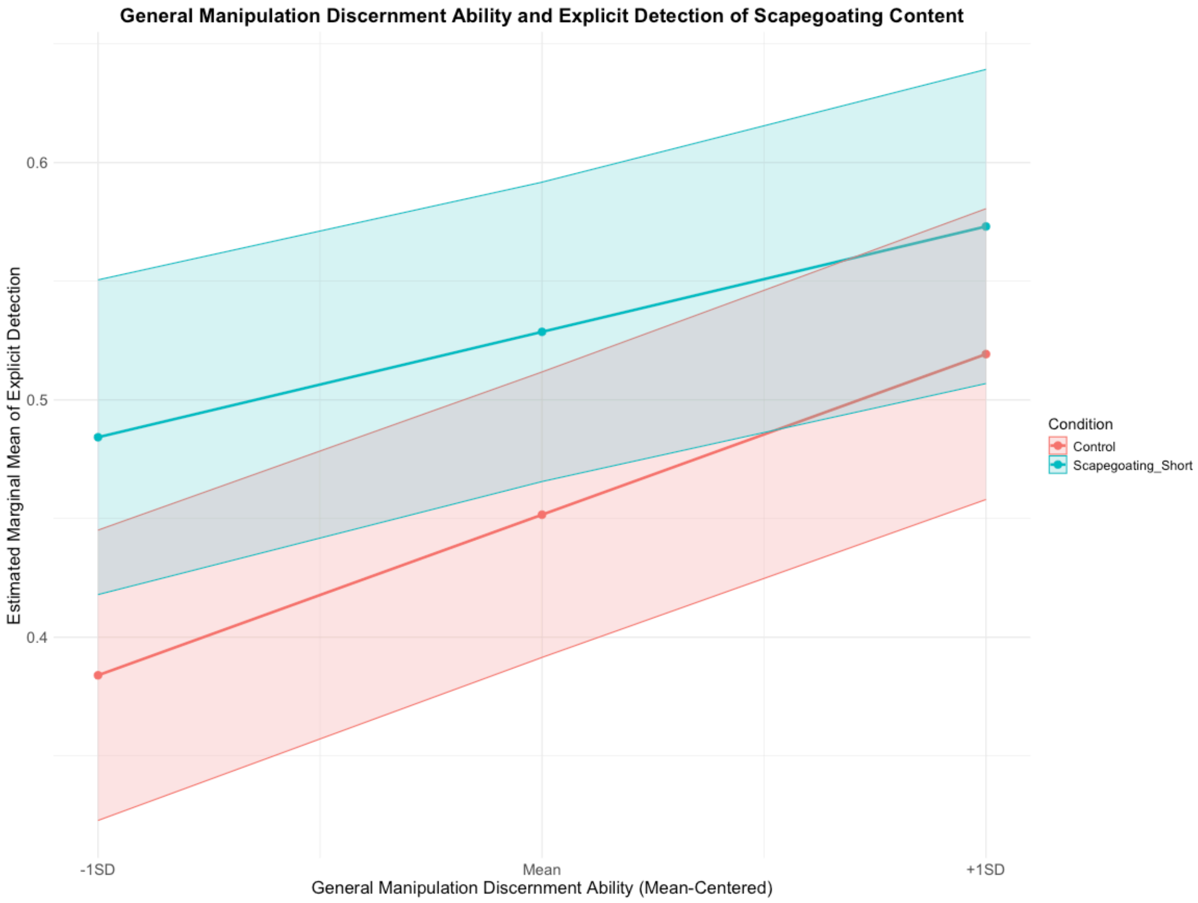

There was a significant interaction between the short scapegoating video (vs. control) and political tolerance when predicting technique recognition of the manipulative scapegoating content, $b = -0.03$, $SE = 0.01$, $t(18{,}340) = 2.10$, $p = .035$, such that the effect was only positive and significant at lower, $b = 0.09$, $SE = 0.02$, $p < .001$, and more moderate levels of political tolerance, $b = 0.07$, $SE = 0.01$, $p < .001$, but not at higher levels, $b = 0.04$, $SE = 0.02$, $p = .119$ (see Figure S10).

Figure S10.

Simple Slopes Plot of the Interaction Effect between the Short Scapegoating Video (vs. Control) and Political Tolerance when Predicting Technique Recognition of the Manipulative Scapegoating Content.

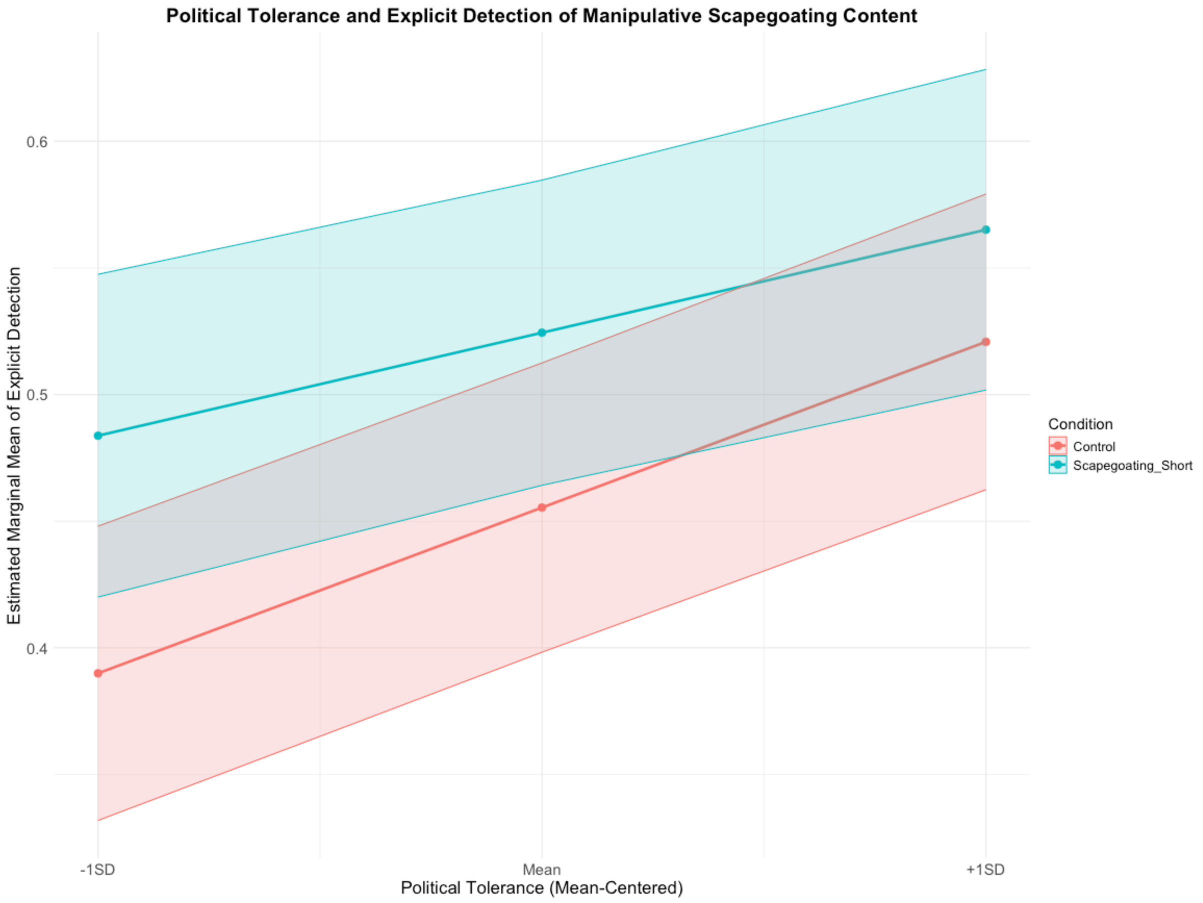

There was a significant interaction between the long scapegoating video (vs. control) and the longitude of nations when predicting technique recognition of the manipulative scapegoating content, b = -0.01, SE = 0.01, t(18,630) = 3.66, p < .001, such that the positive significant effect was strongest among more Western European nations, b = 0.16, SE = 0.01, p < .001, weaker among more central European nations, b = 0.13, SE = 0.01, p < .001, and weakest among more Eastern European nations, b = 0.10, SE = 0.01, p < .001 (see Figure S11).

Figure S11.

Simple Slopes Plot of the Interaction Effect between the Long Scapegoating Video (vs. Control) and Longitude when Predicting Technique Recognition of the Manipulative Scapegoating Content.

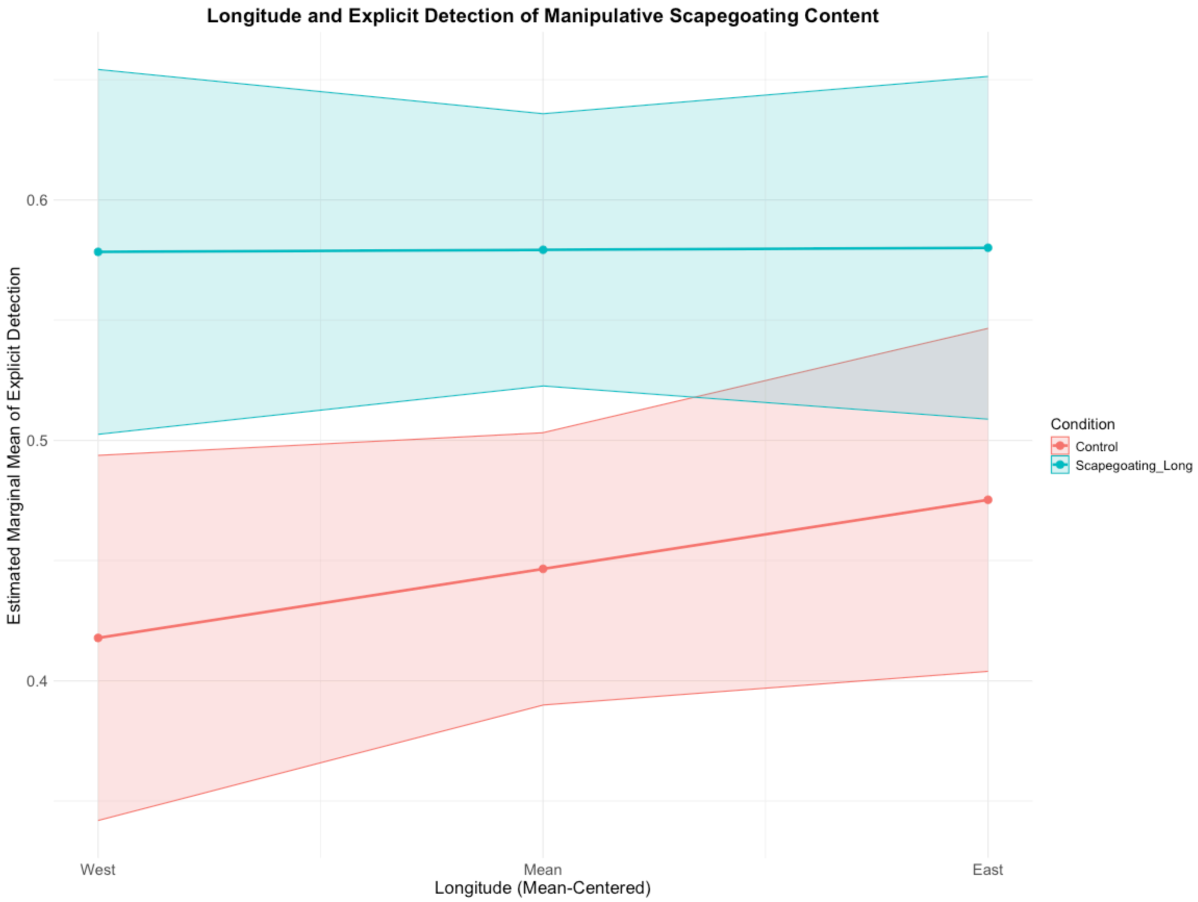

There was a significant interaction between the long scapegoating video (vs. control) and the education indices of nations when predicting technique recognition of the manipulative scapegoating content, $b = 0.35$, $SE = 0.15$, $t(18{,}630) = 2.41$, $p = .016$, such that the positive significant effect was strongest among nations with higher education indices, $b = 0.15$, $SE = 0.01$, $p < .001$, weaker among nations with more moderate education indices, $b = 0.13$, $SE = 0.01$, $p < .001$, and weakest among nations with lower education indices, $b = 0.11$, SE 0.01, $p < .001$ (see Figure S12).

Figure S12.

Simple Slopes Plot of the Interaction Effect between the Long Scapegoating Video (vs. Control) and Education Index when Predicting Technique Recognition of the Manipulative Scapegoating Content.

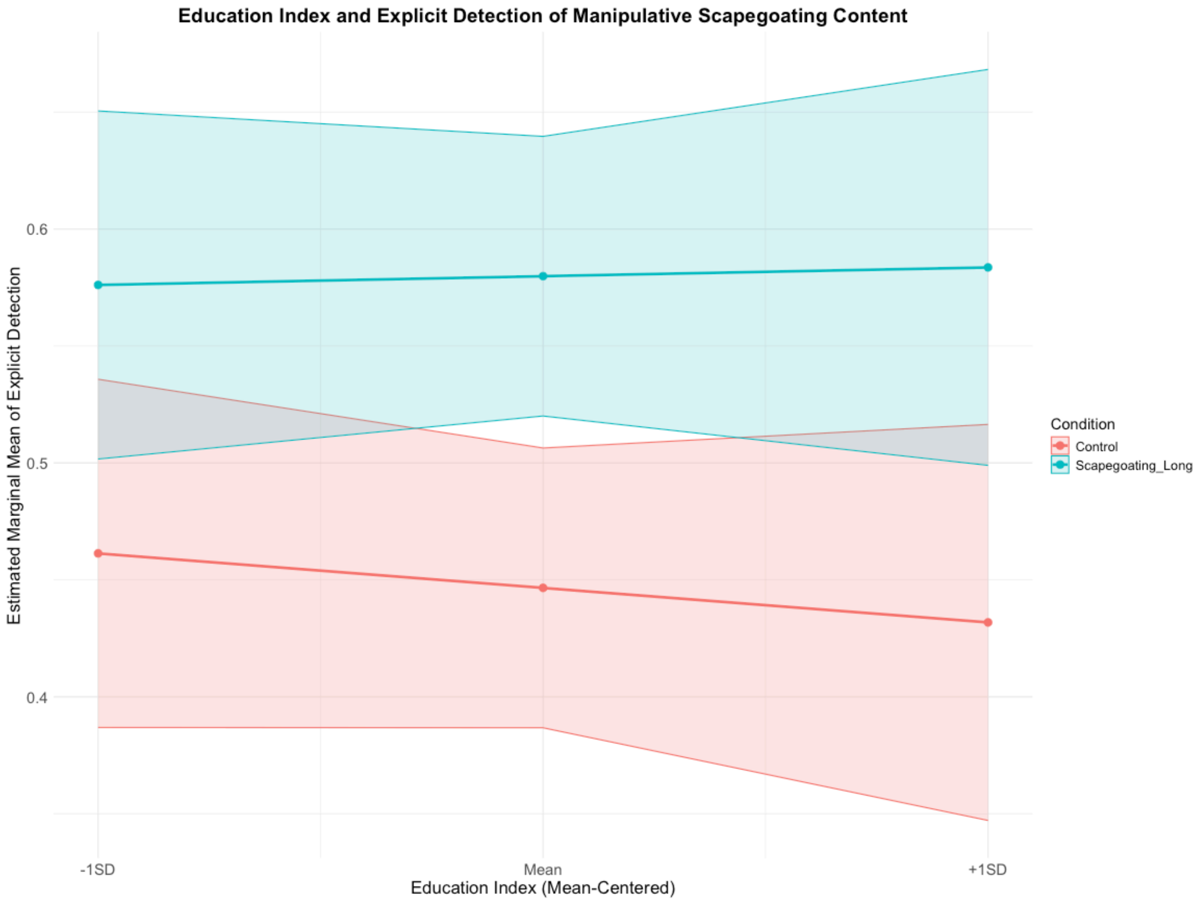

There was a significant interaction between the long scapegoating video (vs. control) and the industrialization indices of nations when predicting technique recognition of the manipulative scapegoating content, *b* = -0.01, *SE* = 0.01, *t*(18,630) = 2.32, *p* = .020, such that the positive significant effect was strongest among nations with lower industrialization indices, *b* = 0.15, *SE* = 0.01, *p* < .001, weaker among nations with more moderate industrialization indices, *b* = 0.13, *SE* = 0.01, *p* < .001, and weakest among nations with higher industrialization indices, *b* = 0.12, *SE* 0.01, *p* < .001 (see Figure S13).

Figure S13.

Simple Slopes Plot of the Interaction Effect between the Long Scapegoating Video (vs. Control) and Industrialization Index when Predicting Technique Recognition of the Manipulative Scapegoating Content.

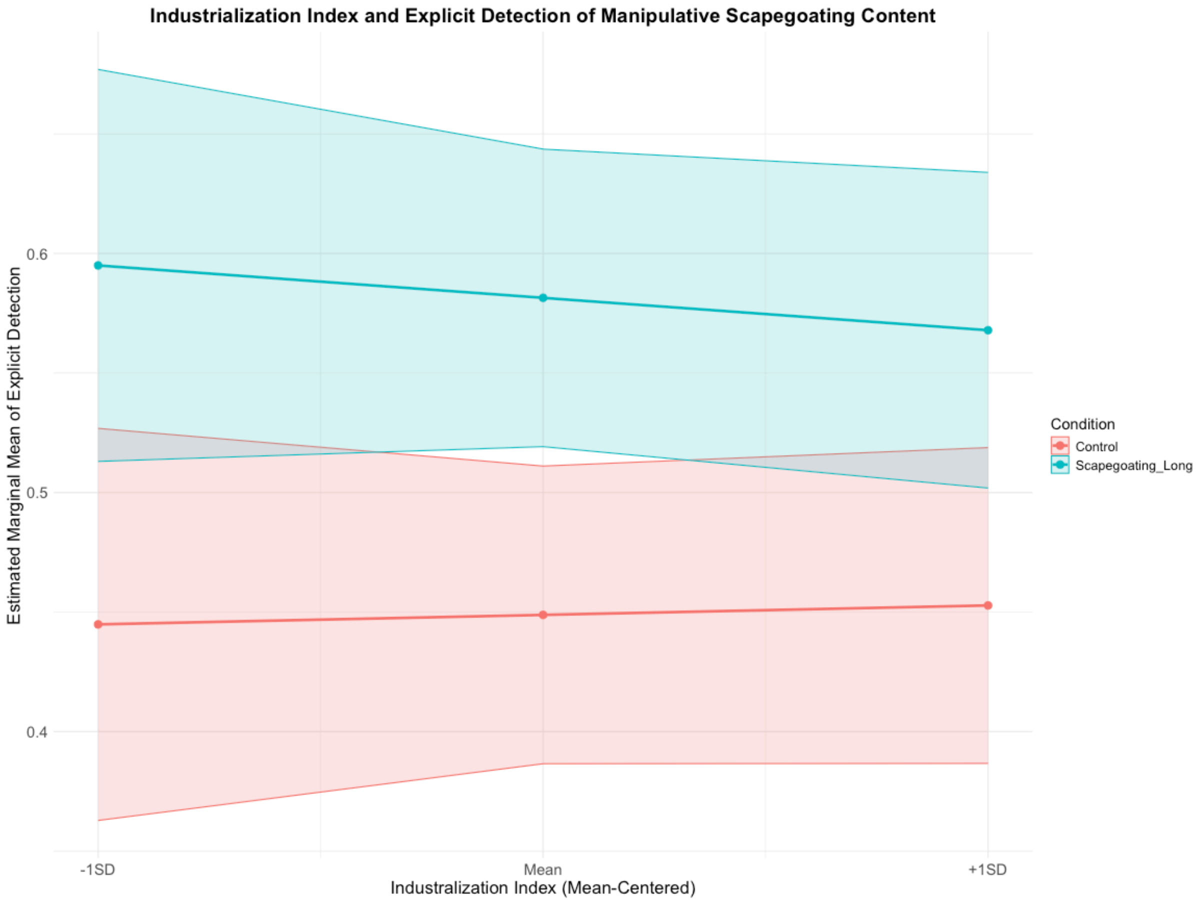

There was a significant interaction between the long scapegoating video (vs. control) and the GDP per capita of nations when predicting technique recognition of the manipulative scapegoating content, $b = 0.01$, $SE = 0.01$, $t(18{,}630) = 3.56$, $p < .001$, such that the positive significant effect was strongest among nations with higher GDPs per capita, $b = 0.16$, $SE = 0.01$, $p < .001$, weaker among nations with more moderate GDPs per capita, $b = 0.13$, $SE = 0.01$, $p < .001$, and weakest among nations with lower GDPs per capita, $b = 0.11$, SE 0.01, $p < .001$ (see Figure S14).

Figure S14.

Simple Slopes Plot of the Interaction Effect between the Long Scapegoating Video (vs. Control) and GDP Per Capita when Predicting Technique Recognition of the Manipulative Scapegoating Content.

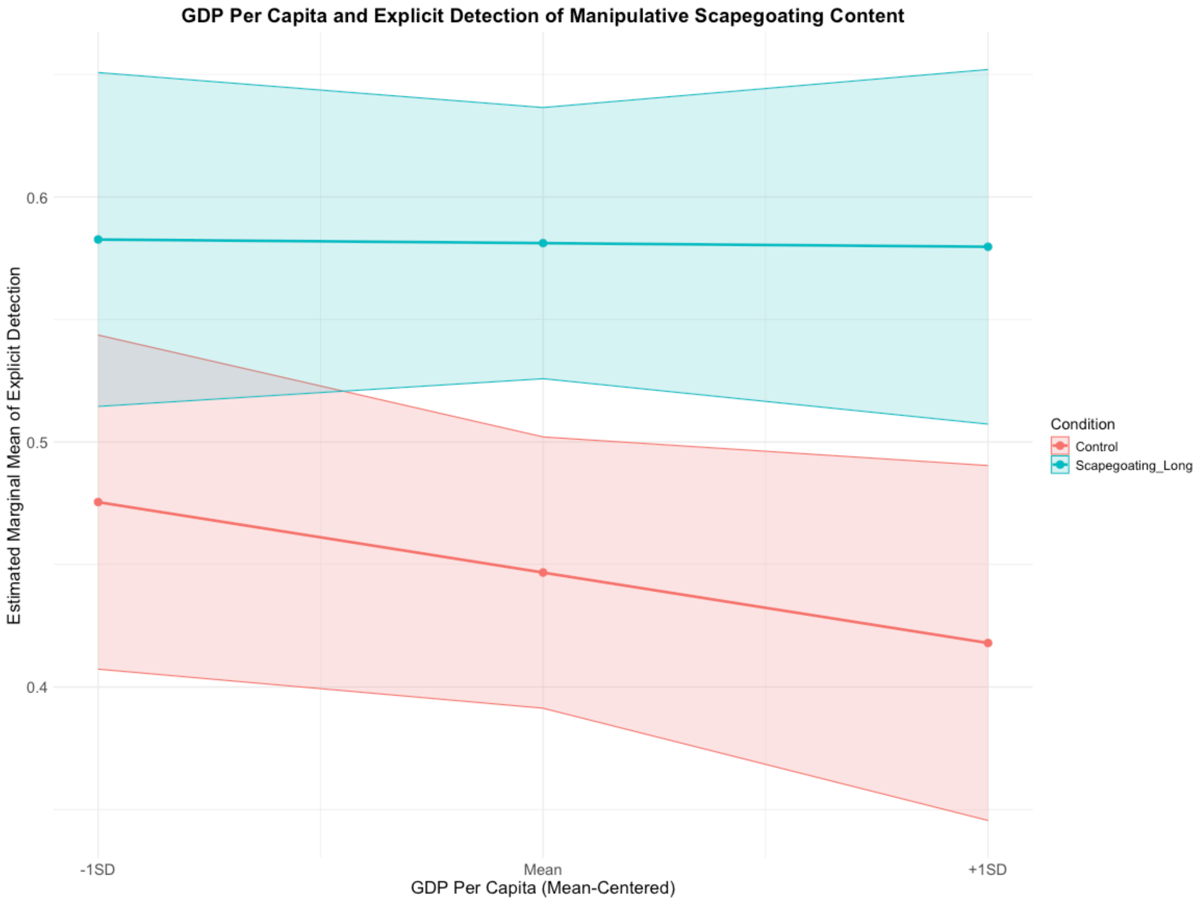

There was a significant interaction between the long scapegoating video (vs. control) and the democratic indices of nations when predicting technique recognition of the manipulative scapegoating content, $b = 0.04$, $SE = 0.01$, $t(18,630) = 4.36$, $p < .001$, such that the positive significant effect was strongest among nations with higher democratic indices, $b = 0.17$, $SE = 0.01$, $p < .001$, weaker among nations with more moderate democratic indices, $b = 0.13$, $SE = 0.01$, $p < .001$, and weakest among nations with lower democratic indices, $b = 0.10$, *SE* 0.01, $p < .001$ (see Figure S15).

Figure S15.

Simple Slopes Plot of the Interaction Effect between the Long Scapegoating Video (vs. Control) and Democratic Index when Predicting Technique Recognition of the Manipulative Scapegoating Content.

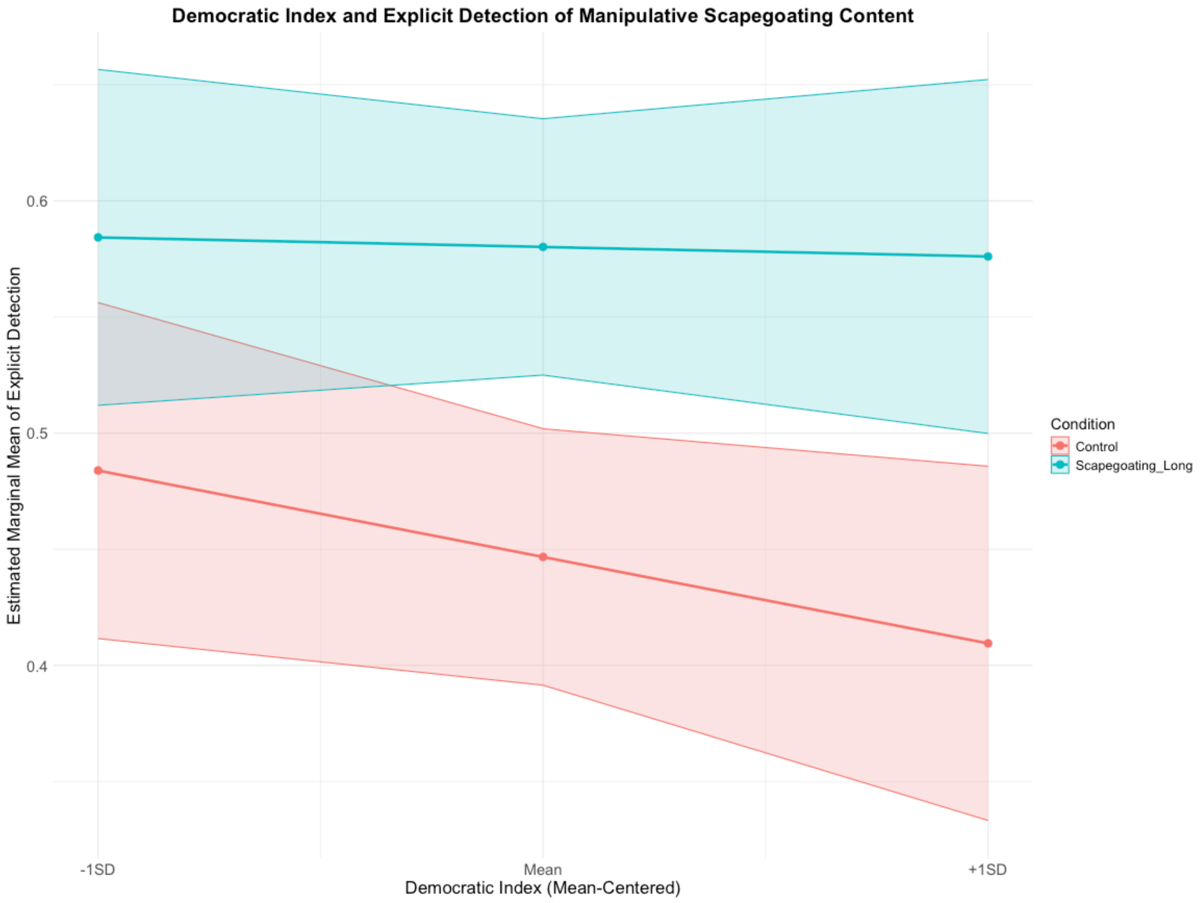

There were significant interactions between both the long, *b* = 0.02, *SE* = 0.04, *t*(18,640) = 3.54, $p < .001$, and short scapegoating videos (vs. control), *b* = 0.03, *SE* = 0.06, *t*(18,640) = 4.58, $p < .001$, and intentions to share the video within one's social network when predicting technique recognition of the manipulative scapegoating content (see Figure S16). For the long scapegoating video (vs. control), the effect was positive and significant at higher, *b* = 0.17, *SE* = 0.01, $p < .001$, more moderate, *b* = 0.14, *SE* = 0.01, $p < .001$, and lower levels of intentions to share, *b* = 0.10, *SE* = 0.01, $p < .001$. For the short scapegoating video (vs. control), the effect was positive and significant at higher, *b* = 0.15, *SE* = 0.02, $p < .001$, and more moderate levels of intentions to share, *b* = 0.09, *SE* = 0.01, $p < .001$, but not when intentions were lower, *b* = 0.03, *SE* = 0.02, *p* = .936.

Figure S16.

Simple Slopes Plot of the Interaction Effects between the Long (Left) and Short (Right) Scapegoating Videos (vs. Control) and Intentions to Share the Video within one's Social Network when Predicting Technique Recognition of the Manipulative Scapegoating Content.

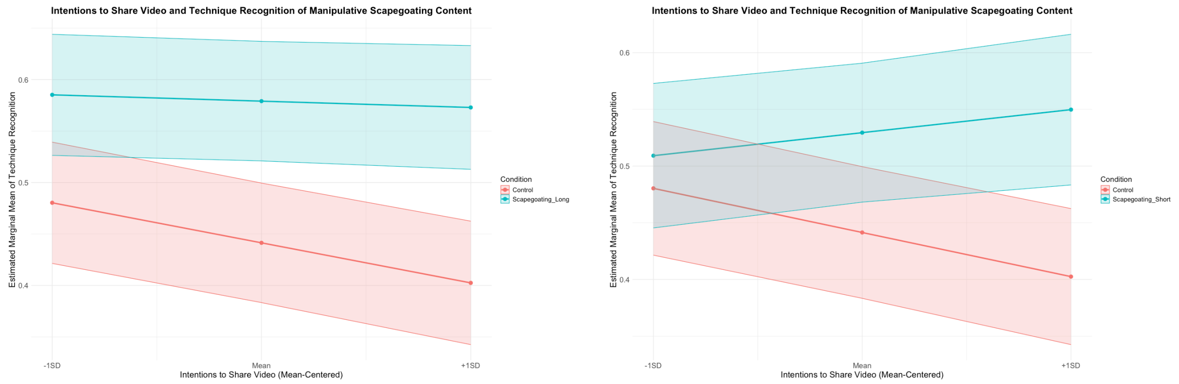

Technique recognition of non-manipulative scapegoating content

There was a significant interaction between the long scapegoating video (vs. control) and political tolerance when predicting technique recognition of the non-manipulative scapegoating content, $b = 0.02$, $SE = 0.01$, $t(18{,}350) = 2.43$, $p = .015$, such that the negative significant effect was strongest at lower levels of political tolerance, $b = -0.10$, $SE = 0.01$, $p < .001$, weaker at more moderate levels, $b = -0.08$, $SE = 0.01$, $p < .001$, and weakest at higher levels, $b = -0.06$, $SE = 0.01$, $p < .001$ (see Figure S17).

Figure S17.

Simple Slopes Plot of the Interaction Effect between the Long Scapegoating Video (vs. Control) and Political Tolerance when Predicting Technique Recognition of the Non-Manipulative Scapegoating Content.

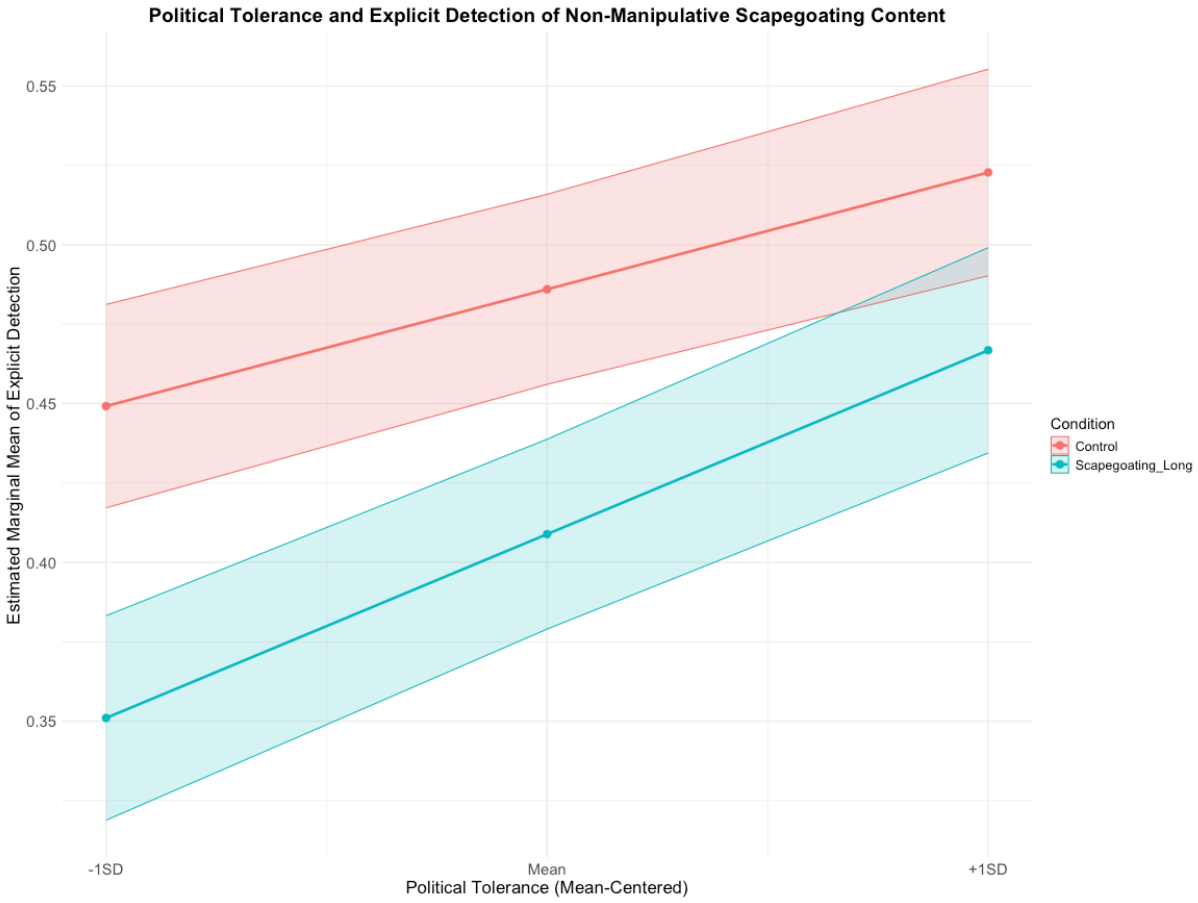

There was a significant interaction between the long scapegoating video (vs. control) and education attainment when predicting technique recognition of the non-manipulative scapegoating content, $b = 0.02$, $SE = 0.01$, $t(18{,}630) = 2.45$, $p = .014$, such that the negative significant effect was strongest at lower levels of educational attainment, $b = -0.10$, $SE = 0.01$, $p < .001$, weaker at more moderate levels, $b = -0.08$, $SE = 0.01$, $p < .001$, and weakest at higher levels, $b = -0.06$, $SE = 0.01$, $p < .001$ (see Figure S18).

Figure S18.

Simple Slopes Plot of the Interaction Effect between the Long Scapegoating Video (vs. Control) and Educational Attainment when Predicting Technique Recognition of the Non-Manipulative Scapegoating Content.

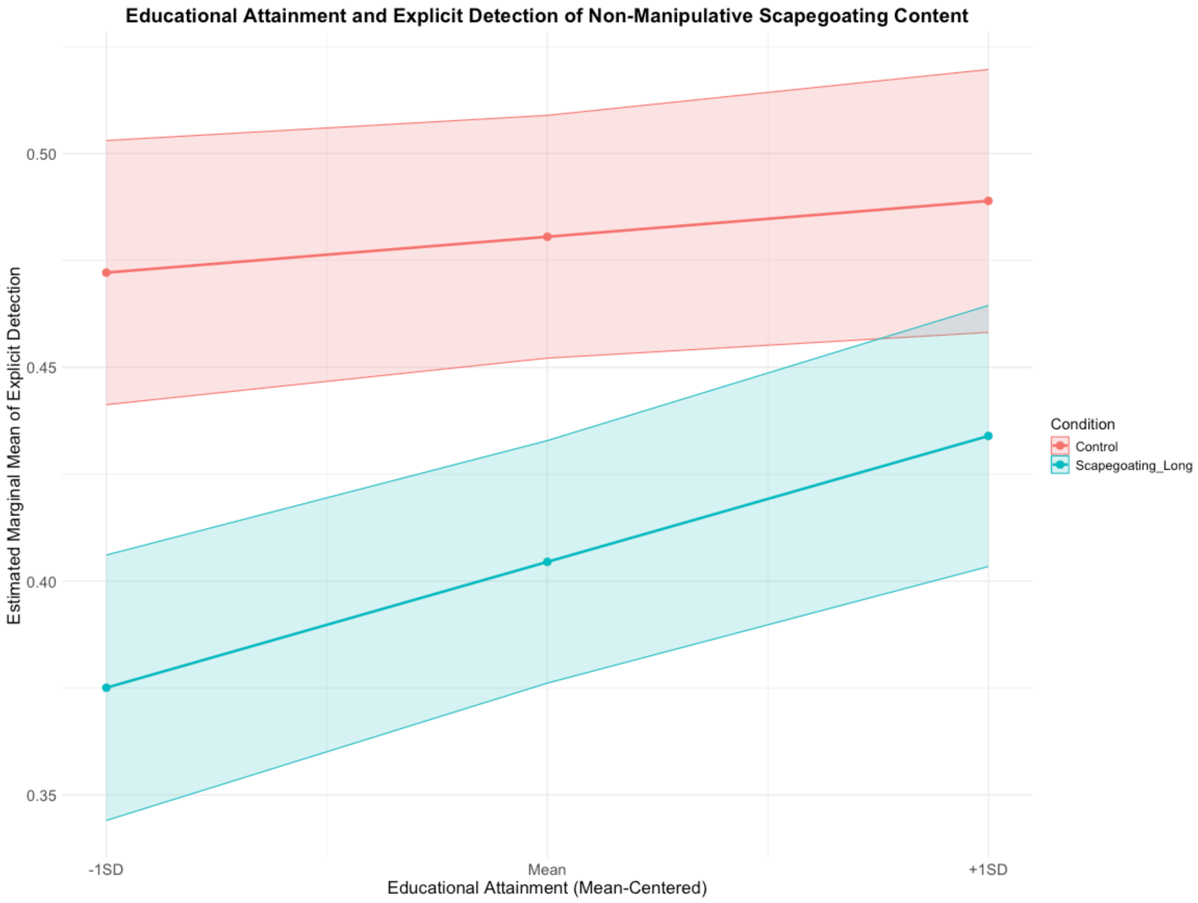

Technique discernment of scapegoating content

There was a significant interaction between the long scapegoating video (vs. control) and longitude when predicting technique discernment of the scapegoating content, $b = -0.01$, $SE = 0.01$, $t(19{,}370) = 3.53$, $p < .001$, such that the effect was only positive and significant among more Western European, $b = 0.04$, $SE = 0.01$, $p < .001$, and central European nations, $b = 0.02$, $SE = 0.01$, $p = .003$, but not among more Eastern European nations, $b = 0.01$, $SE = 0.01$, $p = .999$ (see Figure S19).

Figure S19.

Simple Slopes Plot of the Interaction Effect between the Long Scapegoating Video (vs. Control) and Longitude when Predicting Technique Discernment of the Scapegoating Content.

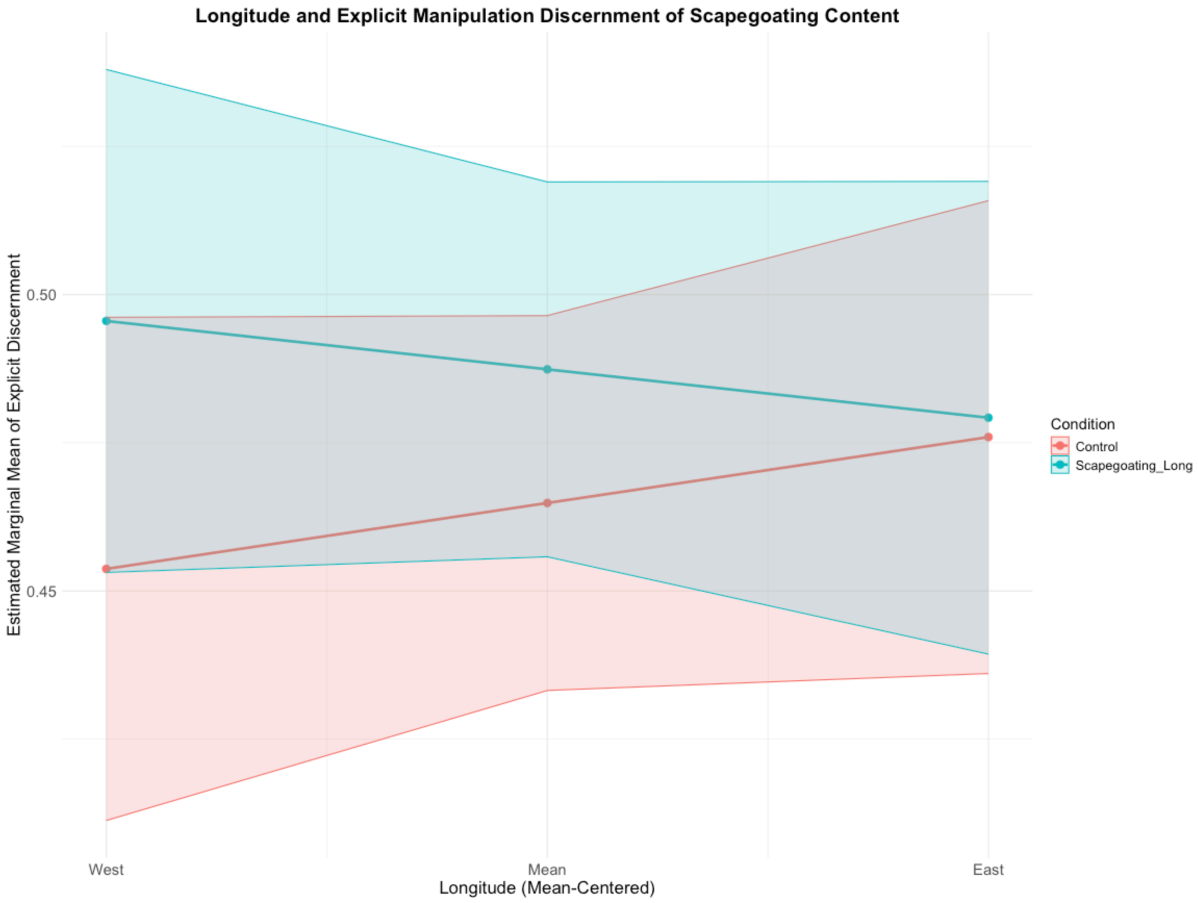

There was a significant interaction between the long scapegoating video (vs. control) and the education indices of nations when predicting technique discernment of the scapegoating content, $b = 0.30$, $SE = 0.10$, $t(19{,}370) = 2.91$, $p = .004$, such that the effect was only positive and significant among nations with higher, $b = 0.04$, $SE = 0.01$, $p < .001$, and more moderate education indices, $b = 0.02$, $SE = 0.01$, $p = .002$, but not among nations with lower education indices, $b = 0.01$, $SE = 0.01$, $p = .999$ (see Figure S20).

Figure S20.

Simple Slopes Plot of the Interaction Effect between the Long Scapegoating Video (vs. Control) and Education Index when Predicting Technique Discernment of the Scapegoating Content.

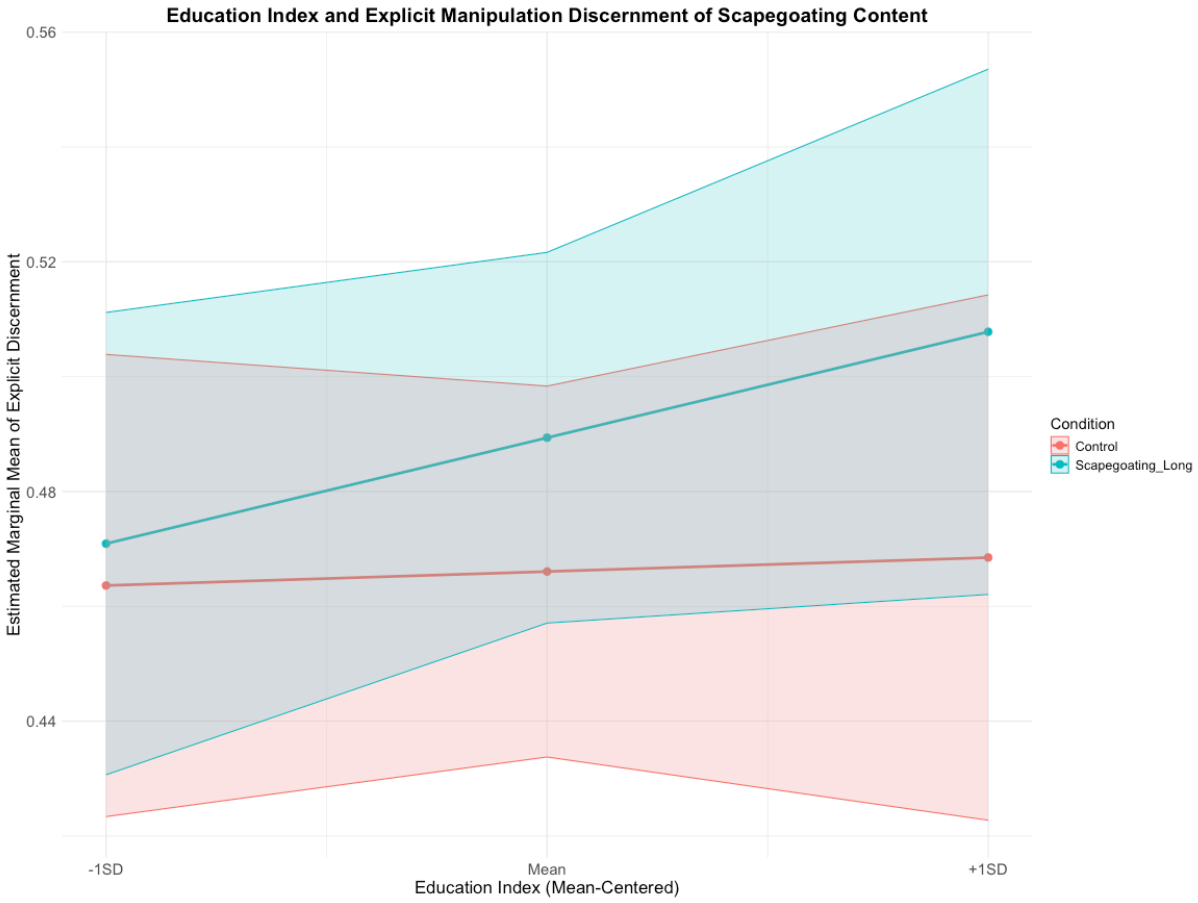

There was a significant interaction between the long scapegoating video (vs. control) and the GDP per capita of nations when predicting technique discernment of the scapegoating content, $b = 0.01$, $SE = 0.01$, $t(19{,}370) = 2.52$, $p = .012$, such that the effect was only positive and significant among nations with higher, $b = 0.04$, $SE = 0.01$, $p < .001$, and more moderate GDPs per capita, $b = 0.02$, $SE = 0.01$, $p = .002$, but not among nations with lower GDPs per capita, $b = 0.01$, $SE = 0.01$, $p = .999$ (see Figure S21).

Figure S21.

Simple Slopes Plot of the Interaction Effect between the Long Scapegoating Video (vs. Control) and GDP Per Capita when Predicting Technique Discernment of the Scapegoating Content.

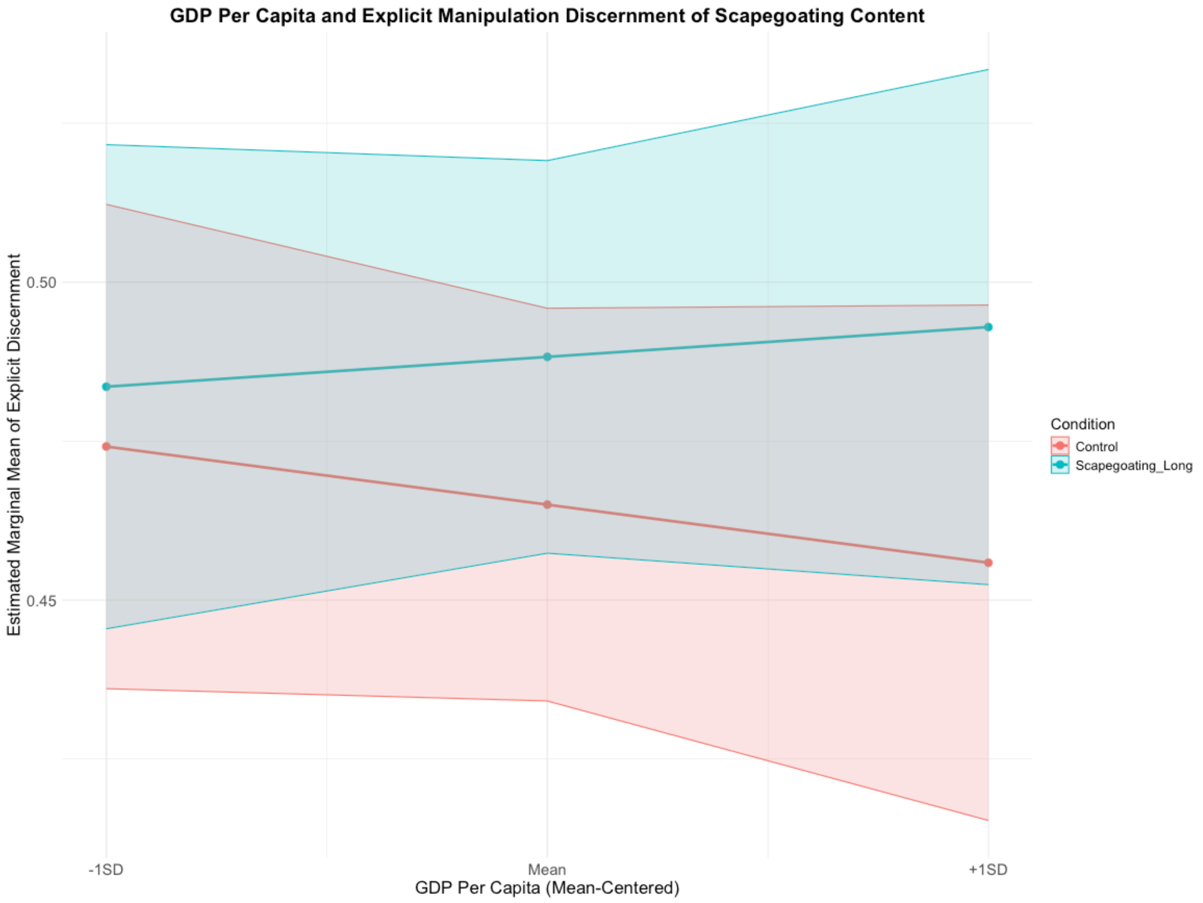

There was a significant interaction between the long scapegoating video (vs. control) and the democratic indices of nations when predicting technique discernment of the scapegoating content, $b = 0.03$, $SE = 0.01$, $t(19{,}370) = 4.13$, $p < .001$, such that the effect was only positive and significant among nations with higher, $b = 0.05$, $SE = 0.01$, $p < .001$, and more moderate democratic indices, $b = 0.02$, $SE = 0.01$, $p = .002$, but not among nations with lower democratic indices, $b = 0.01$, $SE = 0.01$, $p = .999$ (see Figure S22).

Figure S22.

Simple Slopes Plot of the Interaction Effect between the Long Scapegoating Video (vs. Control) and Democratic Index when Predicting Technique Discernment of the Scapegoating Content.

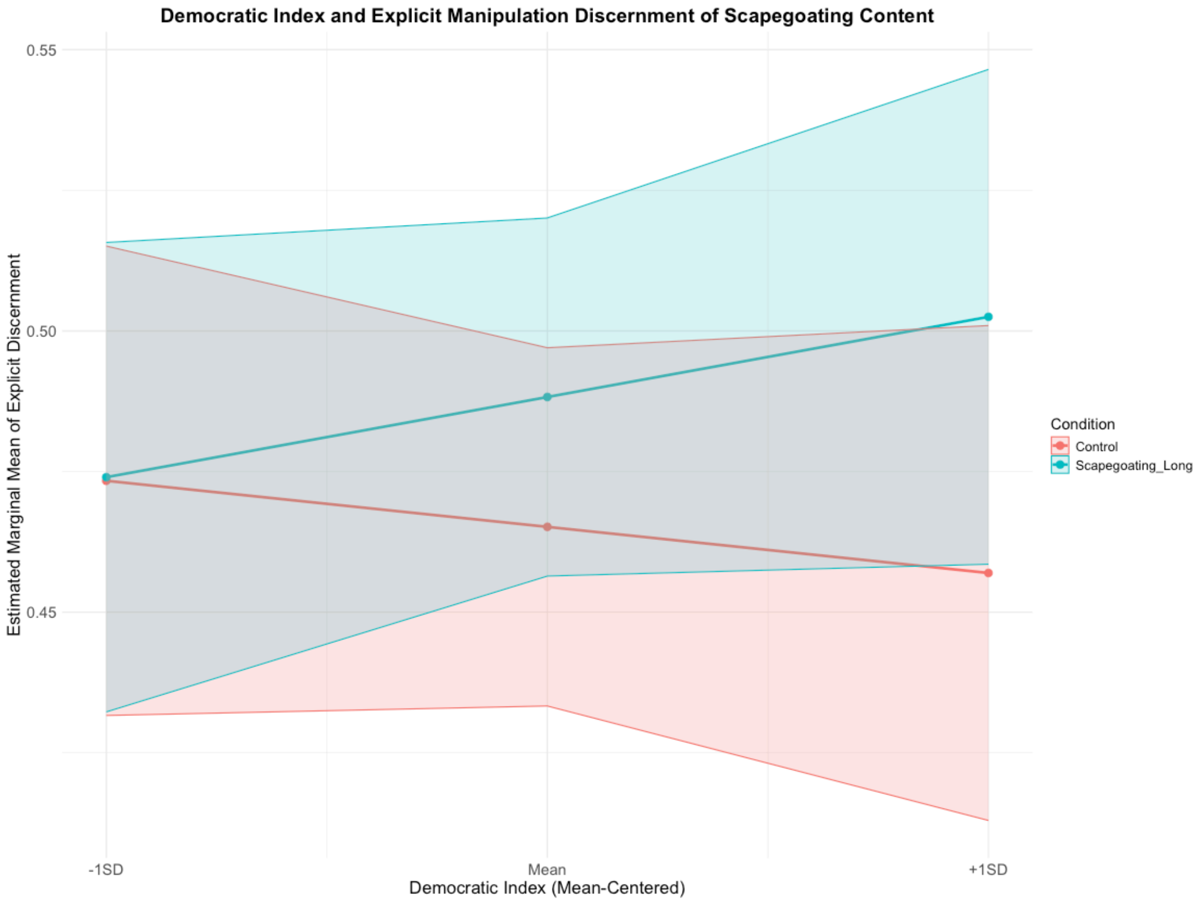

There was a significant interaction between the long scapegoating video (vs. control) and educational attainment when predicting technique discernment of the scapegoating content, $b = 0.01$, $SE = 0.01$, $t(19{,}370) = 2.26$, $p = .024$, such that the effect was only positive and significant among higher, $b = 0.03$, $SE = 0.01$, $p < .001$, and more moderate levels of educational attainment, $b = 0.02$, $SE = 0.01$, $p = .010$, but not at lower levels, $b = 0.01$, $SE = 0.01$, $p = .999$ (see Figure S23).

Figure S23.

Simple Slopes Plot of the Interaction Effect between the Long Scapegoating Video (vs. Control) and Educational Attainment when Predicting Technique Discernment of the Scapegoating Content.

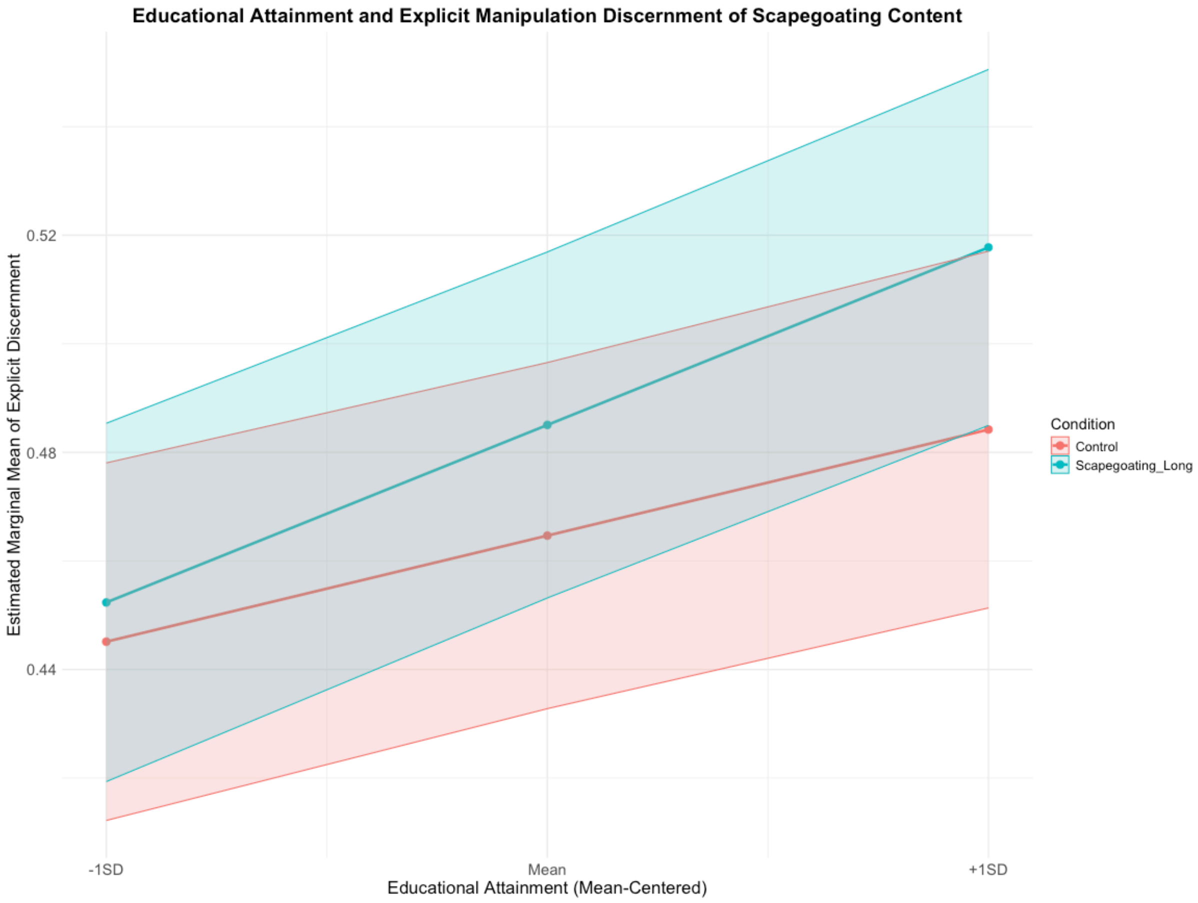

There was a significant interaction between the short scapegoating video (vs. control) and intentions to share the video within one's social network when predicting technique discernment of the scapegoating content, $b = 0.02$, $SE = 0.01$, $t(17{,}900) = 2.11$, $p = .035$ (see Figure S24). The effect of the short scapegoating video (vs. control) was positive and significant at higher, $b = -0.19$, $SE = 0.03$, $p < .001$, more moderate, $b = -0.15$, $SE = 0.02$, $p < .001$, and lower levels of intentions to share, $b = -0.11$, $SE = 0.02$, $p < .001$.

Figure S24.

Simple Slopes Plot of the Interaction Effect between the Short Scapegoating Video (vs. Control) and Intentions to Share the Video Within One's Social Network when Predicting Technique Discernment of the Scapegoating Content.

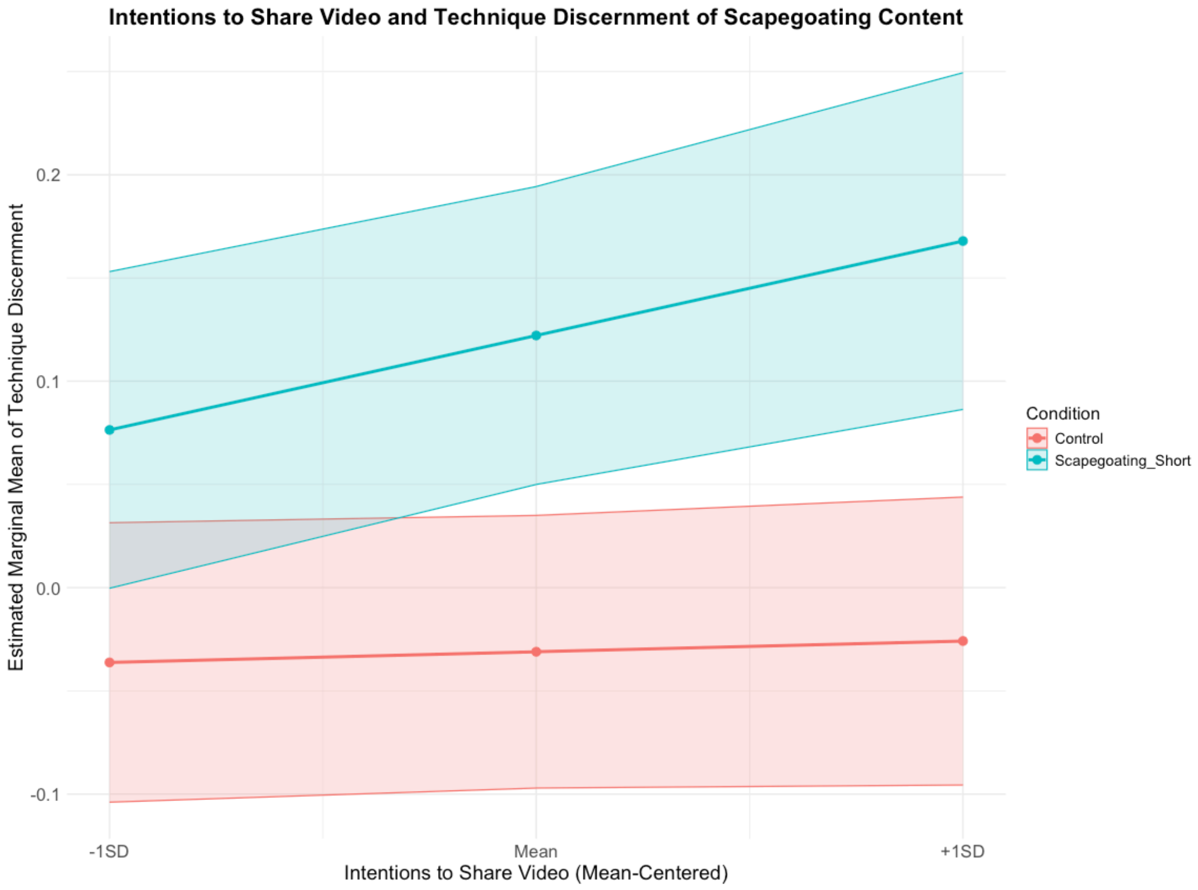

,Sharing decisions for scapegoating content

There was a significant interaction between the long scapegoating video (vs. control) and general manipulation discernment ability when predicting sharing decisions for the scapegoating content, b = 0.04, SE = 0.02, t(17,530) = 2.64, p = .008, such that the effect was only positive and significant at higher, b = 0.17, SE = 0.04, p = .001, and more moderate levels of general manipulation discernment ability, b = 0.09, SE = 0.03, p = .044, but not at lower levels, b = 0.01, SE = 0.04, p = .999 (see Figure S25).

Figure S25.

Simple Slopes Plot of the Interaction Effect between the Long Scapegoating Video (vs. Control) and General Manipulation Discernment Ability when Predicting Sharing Decisions for the Scapegoating Content.

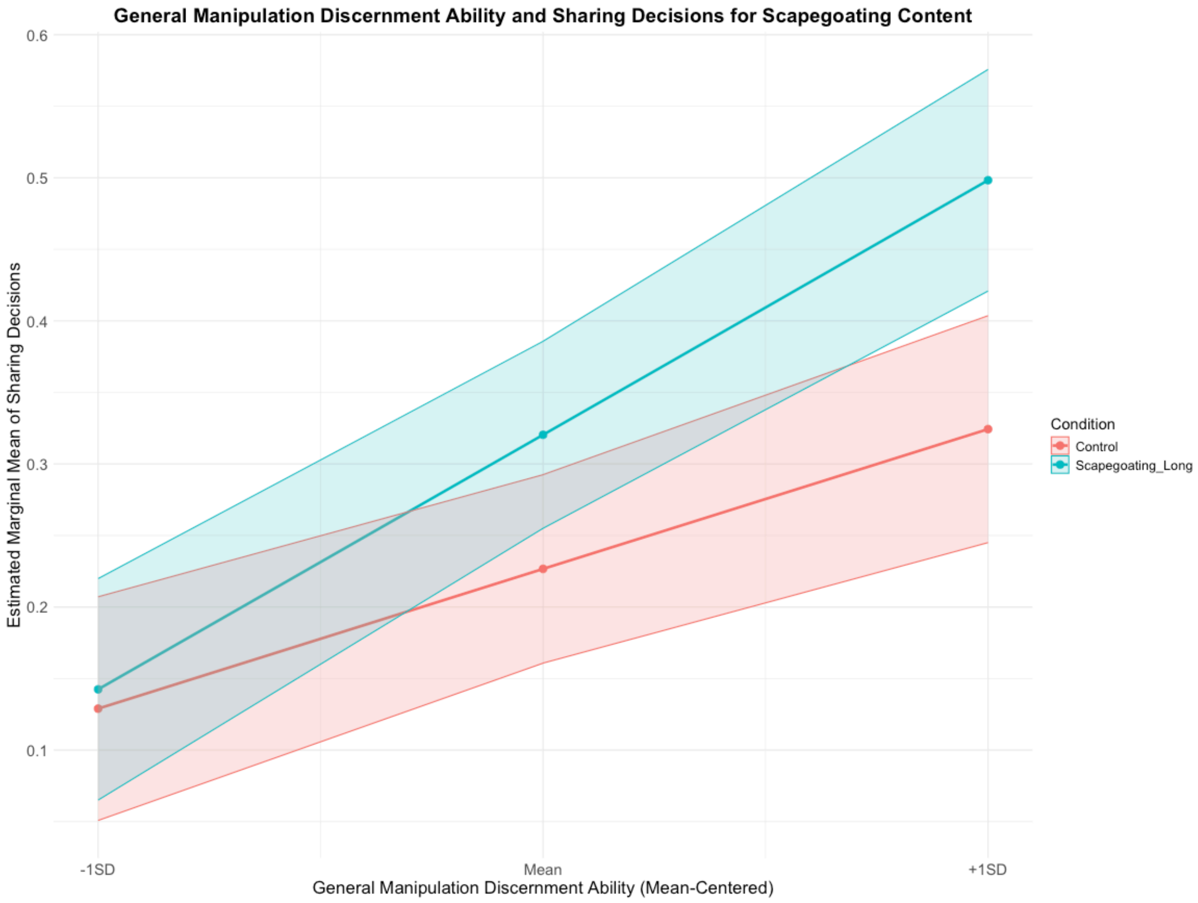

There was a significant interaction between the long scapegoating video (vs. control) and digital literacy when predicting sharing decisions for the scapegoating content, $b = 0.08$, $SE = 0.03$, $t(17{,}350) = 2.30$, $p = .022$, such that the effect was only positive and significant at higher, $b = 0.16$, $SE = 0.04$, $p = .003$, and more moderate levels of digital literacy, $b = 0.09$, $SE = 0.03$, $p = .047$, but not at lower levels, $b = 0.02$, $SE = 0.04$, $p = .999$ (see Figure S26).

Figure S26.

Simple Slopes Plot of the Interaction Effect between the Long Scapegoating Video (vs. Control) and Digital Literacy when Predicting Sharing Decisions for the Scapegoating Content.

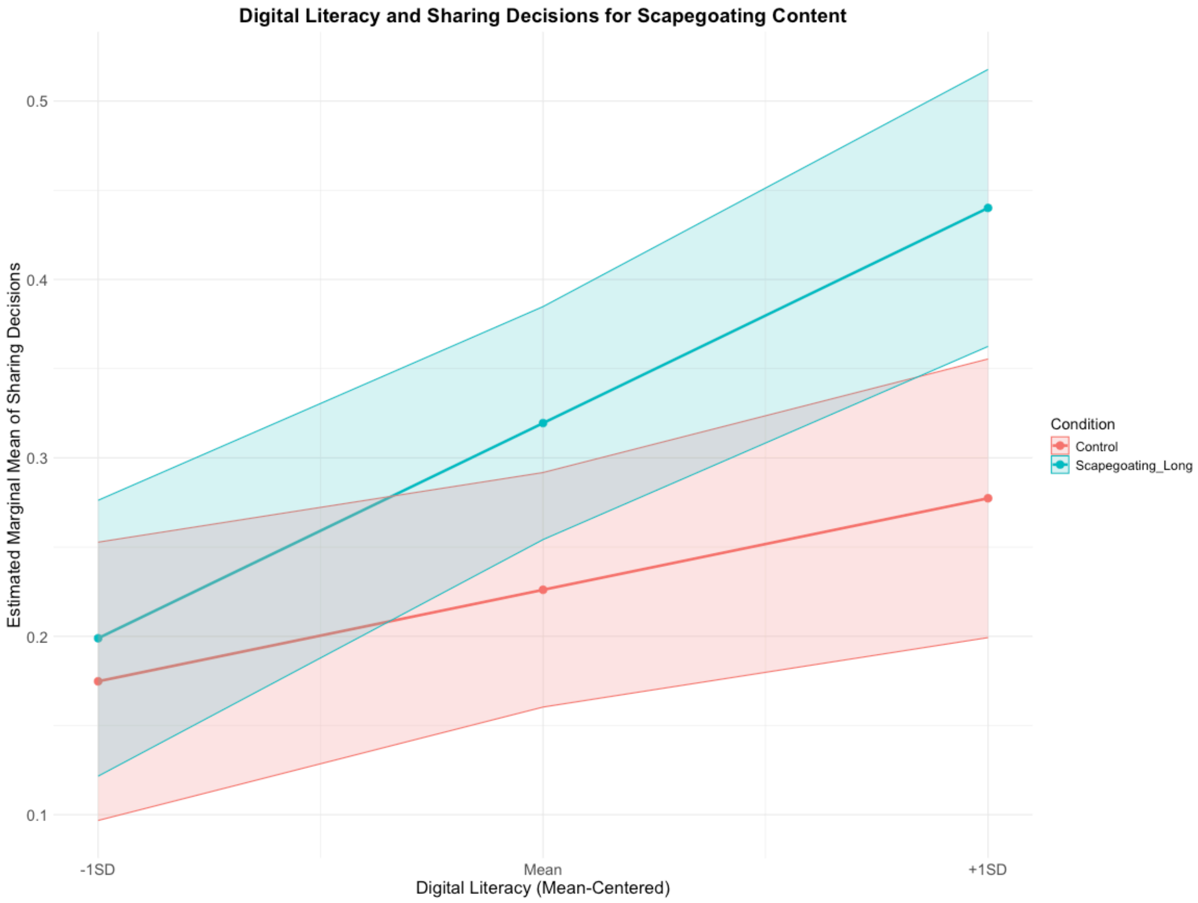

There was a significant interaction between the short scapegoating video (vs. control) and political ideology when predicting sharing decisions for the scapegoating content, *b* = -0.06, *SE* = 0.03, *t*(17,530) = 1.98, *p* = .048*,* such that the effect was only positive and significant among more politically left-wing participants, *b* = 0.19, *SE* = 0.06, *p* = .047, but not among more politically centrist, *b* = 0.10, *SE* = 0.05, *p* = .360, or right-wing participants, *b* = 0.02, *SE* = 0.06, *p* = .999 (see Figure S27).

Figure S27.

Simple Slopes Plot of the Interaction Effect between the Short Scapegoating Video (vs. Control) and Political Ideology when Predicting Sharing Decisions for the Scapegoating Content.

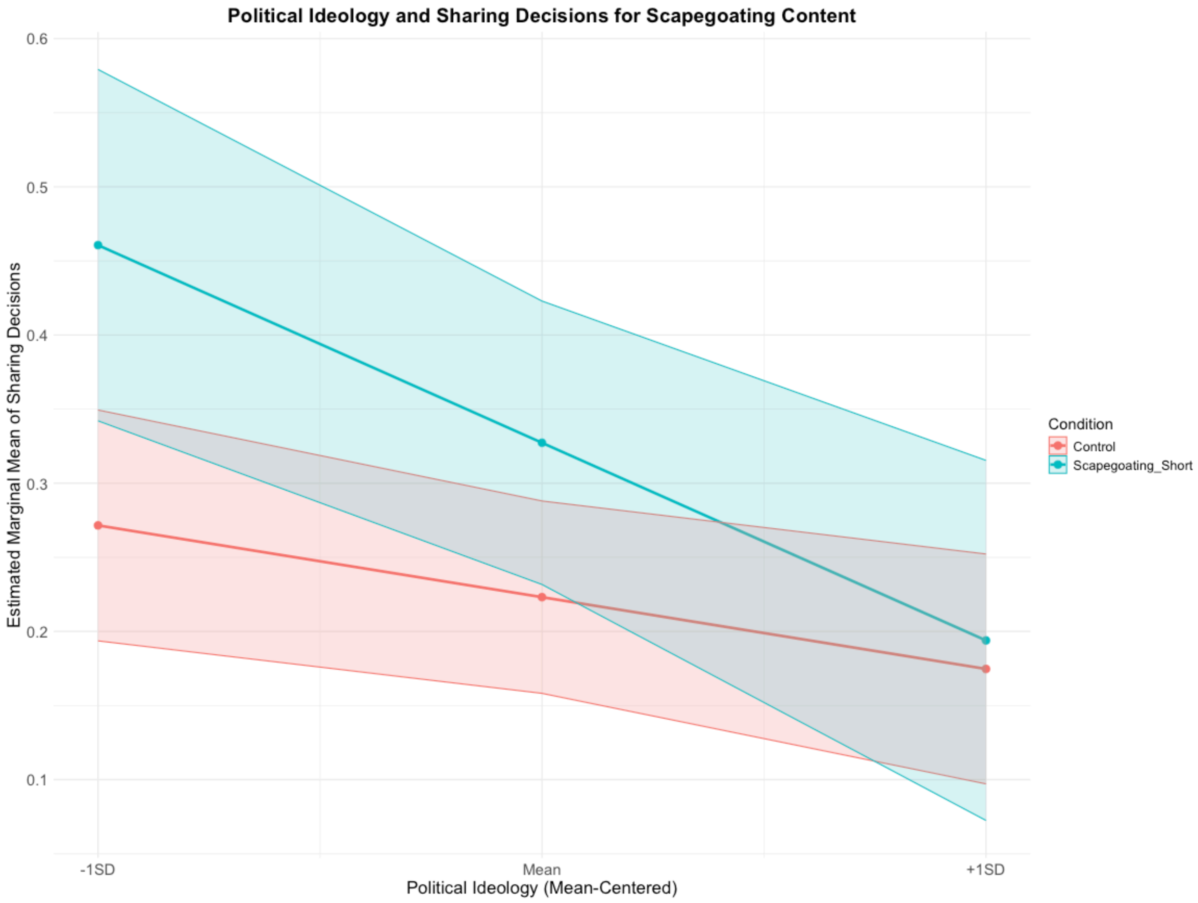

There was a significant interaction between the long scapegoating video (vs. control) and educational attainment when predicting sharing decisions for the scapegoating content, $b = 0.06$, $SE = 0.03$, $t(17{,}530) = 2.25$, $p = .025$, such that the effect was only positive and significant at higher levels of educational attainment, $b = 0.16$, $SE = 0.04$, $p = .003$, but not at more moderate, $b = 0.09$, $SE = 0.03$, $p = .058$, or lower levels, $b = 0.02$, $SE = 0.04$, $p = .999$ (see Figure S28).

Figure S28.

Simple Slopes Plot of the Interaction Effect between the Long Scapegoating Video (vs. Control) and Educational Attainment when Predicting Sharing Decisions for the Scapegoating Content.

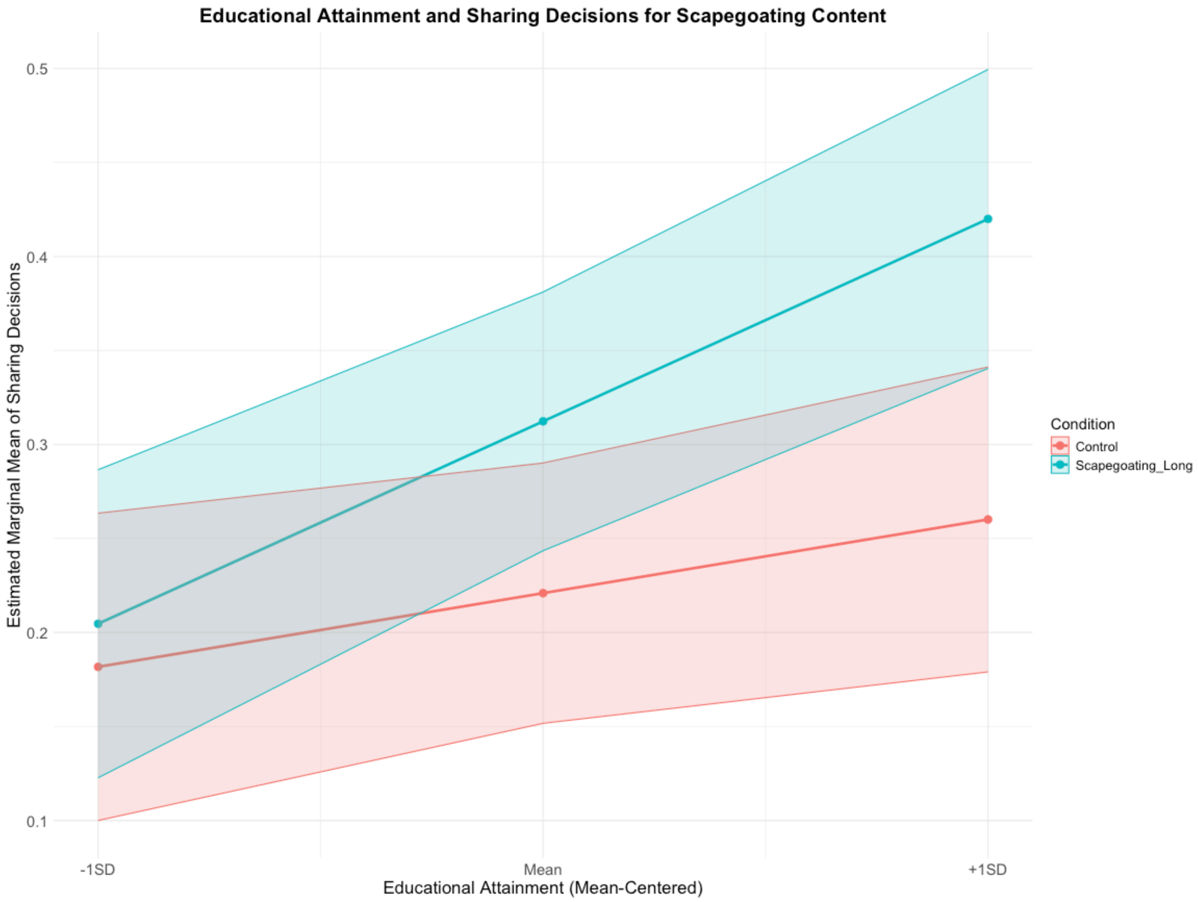

There was a significant interaction between the long scapegoating video (vs. control) and intentions to share the video within one's social network when predicting willingness to share non-manipulative scapegoating content, $b = -0.14$, $SE = 0.02$, $t(18{,}610) = -6.91$, $p < .001$ (see Figure S29). The effect of the long scapegoating video (vs. control) was positive and significant at lower, $b = 0.48$, $SE = 0.05$, $p < .001$, and more moderate levels of intentions to share, $b = 0.18$, $SE = 0.04$, $p < .001$, but not when intentions were or higher, $b = 0.11$, $SE = 0.06$, $p = .678$.

Figure S29.

Simple Slopes Plot of the Interaction Effect between the Long Scapegoating Video (vs. Control) and Intentions to Share the Video Within One's Network when Predicting Willingness to Share the Non-Manipulative Scapegoating Content.

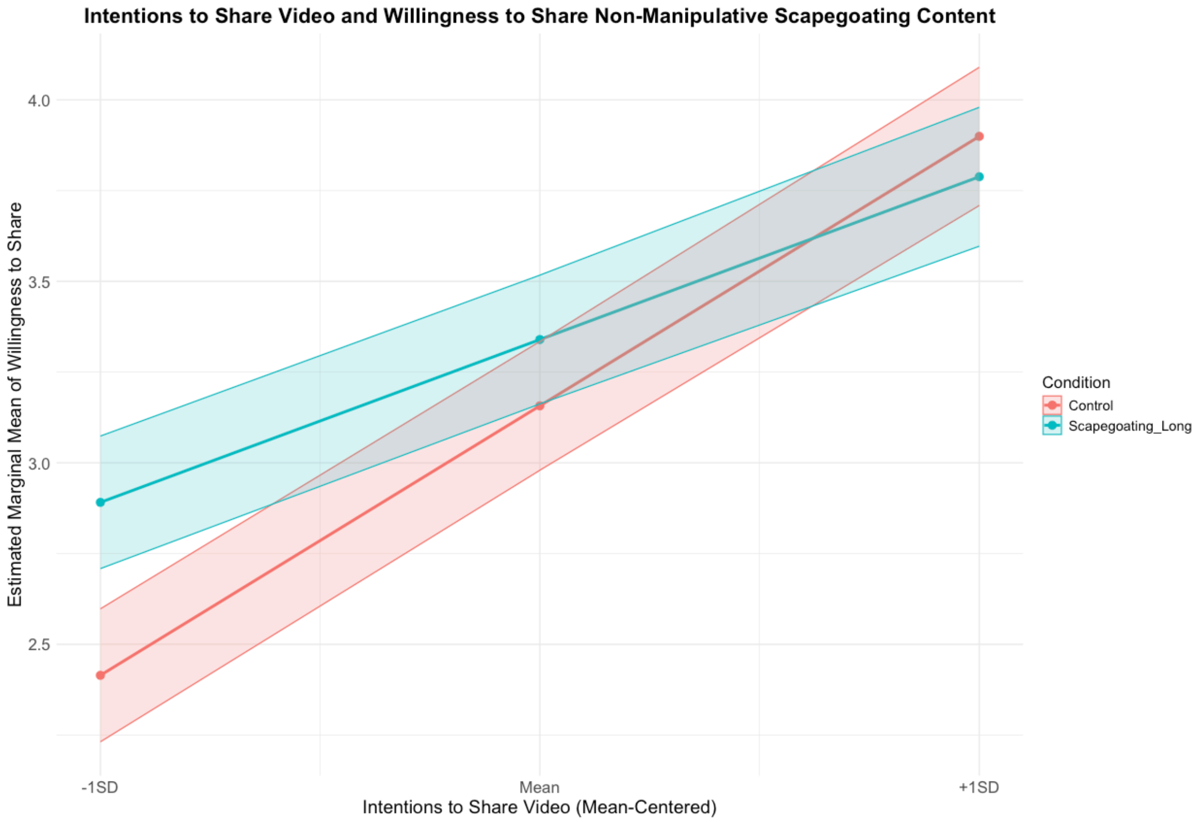

Decontextualization videos

Manipulativeness assessments of decontextualization content

There was a significant interaction between the long decontexutalization video (vs. control) and intentions to share the video within one's social network when predicting manipulativeness assessments of the manipulative decontextualization content, $b = 0.05$, $SE = 0.02$, $t(18,720) = 2.47$, $p = .014$, such that the effect was only positive and significant at higher, $b = 0.23$, $SE = 0.06$, $p < .001$, and more moderate levels of intentions to share the video, $b = 0.13$, $SE = 0.04$, $p = .016$, but not at lower levels, $b = 0.03$, $SE = 0.06$, $p = .999$ (see Figure S30). There was also a significant interaction between the long decontextualization video (vs. control) and intentions to share the video within one's social network when predicting manipulativeness assessments of the non-manipulative decontextualization content, $b = 0.05$, $SE = 0.02$, $t(18,720) = 2.72$, $p = .007$, such that the effect was only negative and significant at higher levels of intentions to share the video, $b = -0.18$, $SE = 0.05$, $p = .008$, but not at more moderate, $b = -0.08$, $SE = 0.04$, $p = .518$, or lower levels, $b = 0.02$, $SE = 0.05$,

p = .999 (see Figure S30). There was also a significant interaction between the long decontextualization video (vs. control) and intentions to share the video within one's social network when predicting manipulation discernment of the decontextualization content, *b* = 0.09, *SE* = 0.02, *t*(17,990) = 4.03, *p* < .001, such that the effect was only positive and significant at higher, *b* = 0.41, *SE* = 0.07, *p* < .001, and more moderate levels of intentions to share the video, *b* = 0.21, *SE* = 0.05, *p* < .001, but not at lower levels, *b* = 0.02, *SE* = 0.07, *p* = .999 (see Figure S30).

Figure S30.

Simple Slopes Plot of the Interaction Effect between the Long Decontextualization Video (vs. Control) and Intentions to Share the Video Within One's Social Network when Predicting Manipulativeness Assessments of the Manipulative (Top Left) and Non-Manipulative (Top Right) Decontextualization Content, as well as Manipulation Discernment of the Decontextualization Content (Bottom Left).

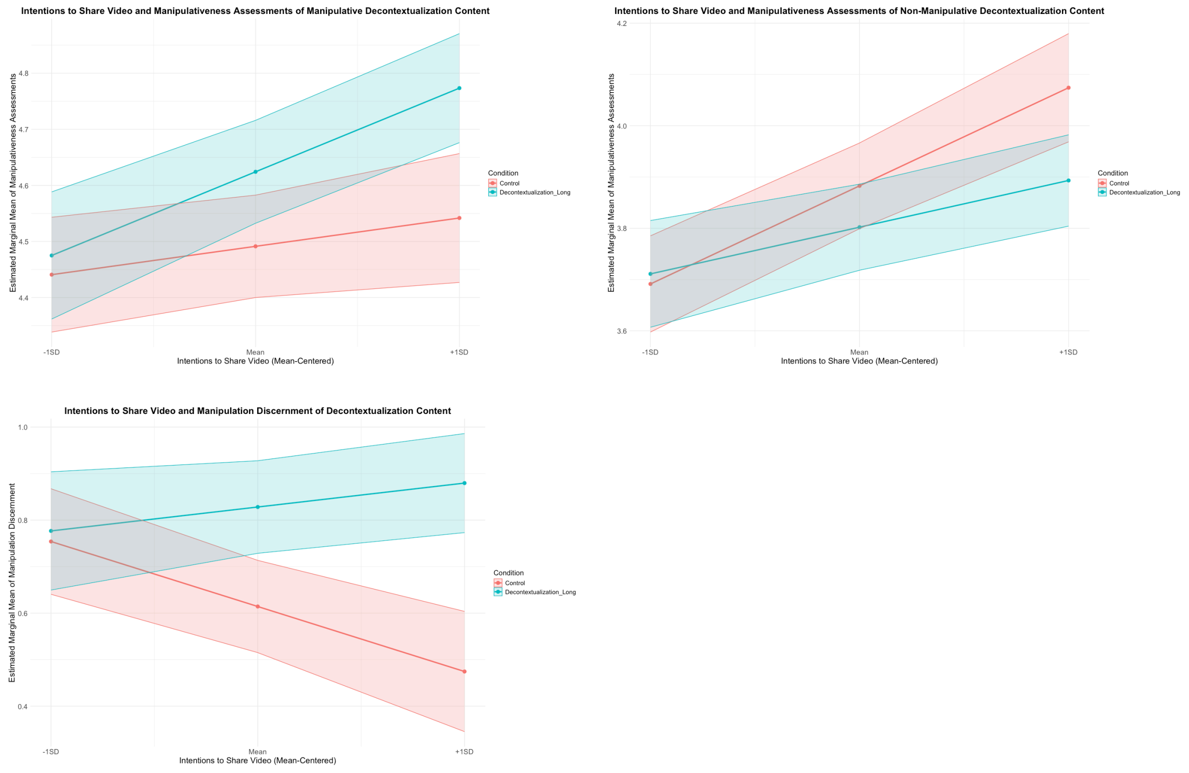

Technique recognition of manipulative decontextualization content

There was a significant interaction between the long decontextualization video (vs. control) and general manipulation discernment ability when predicting technique recognition of the manipulative decontextualization content, $b = -0.01$, $SE = 0.01$, $t(18{,}710) = 2.66$, $p = .008$, such that the positive significant effect was strongest at lower levels of general manipulation discernment ability, $b = 0.13$, $SE = 0.01$, $p < .001$, weaker at more moderate levels, $b = 0.11$, $SE = 0.01$, $p < .001$, and weakest at higher levels, $b = 0.09$, $SE = 0.01$, $p < .001$ (see Figure S31).

Figure S31.

Simple Slopes Plot of the Interaction Effect between the Long Decontextualization Video (vs. Control) and General Manipulation Discernment Ability when Predicting Technique Recognition of the Manipulative Decontextualization Content.

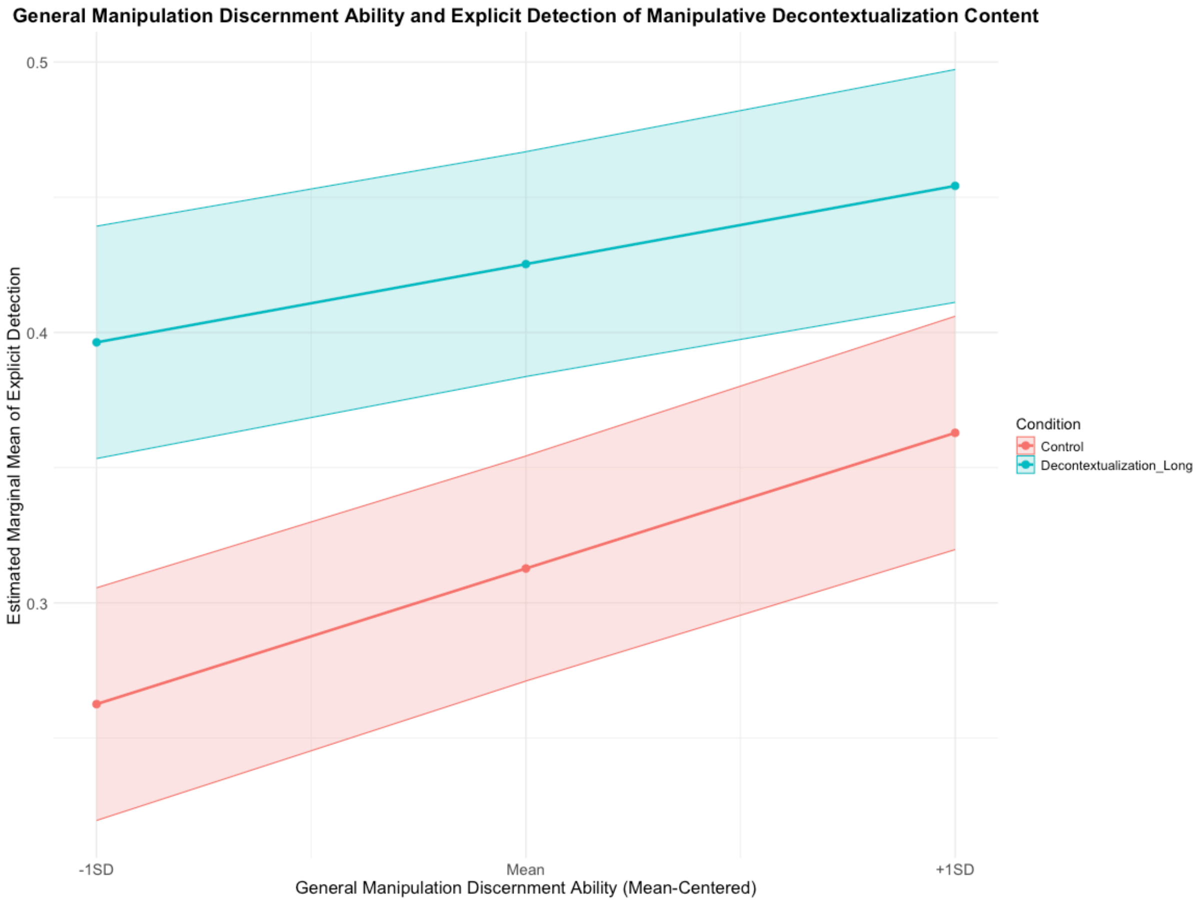

There was a significant interaction between the long decontextualization video (vs. control) and digital literacy when predicting technique recognition of the manipulative decontextualization content, *b* = -0.02, *SE* = 0.01, *t*(18,460) = 2.67, *p* = .008, such that the positive significant effect was strongest at lower levels of digital literacy, *b* = 0.13, *SE* = 0.01, *p* < .001, weaker at more moderate levels, *b* = 0.11, *SE* = 0.01, *p* < .001, and weakest at higher levels, *b* = 0.09, *SE* = 0.01, *p* < .001 (see Figure S32).

Figure S32.

Simple Slopes Plot of the Interaction Effect between the Long Decontextualization Video (vs. Control) and Digital Literacy when Predicting Technique Recognition of the Manipulative Decontextualization Content.

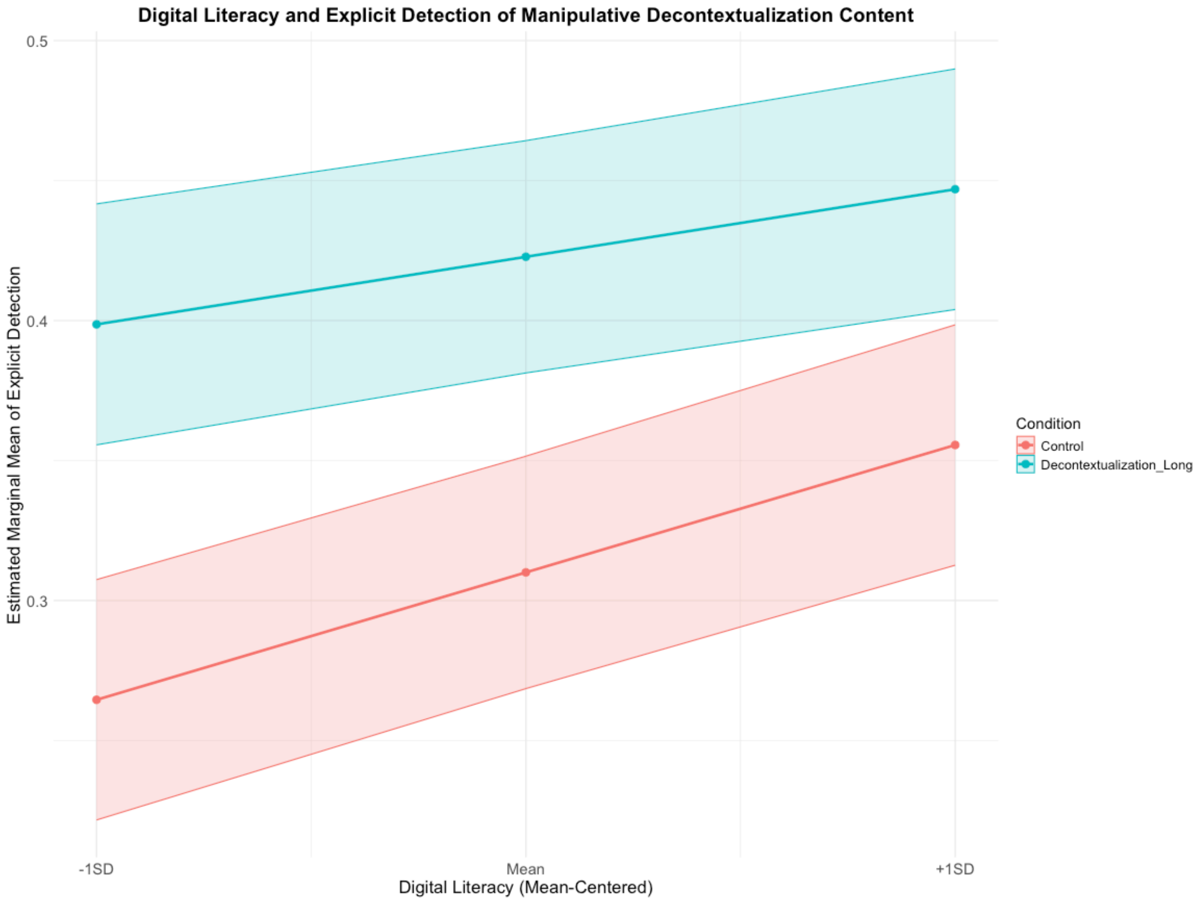

There was a significant interaction between the short decontextualization video (vs. control) and political ideology when predicting technique recognition of the manipulative

decontextualization content, $b = -0.02$, $SE = 0.01$, $t(18{,}710) = 2.21$, $p = .027$, such that the positive significant effect was strongest among more politically left-wing participants, $b = 0.13$, $SE = 0.02$, $p < .001$, weaker among more politically centrist participants, $b = 0.10$, $SE = 0.01$, $p < .001$, and weakest among more politically right-wing participants, $b = 0.07$, $SE = 0.02$, $p < .001$ (see Figure S33).

Figure S33.

Simple Slopes Plot of the Interaction Effect between the Short Decontextualization Video (vs. Control) and Political Ideology when Predicting Technique Recognition of the Manipulative Decontextualization Content.

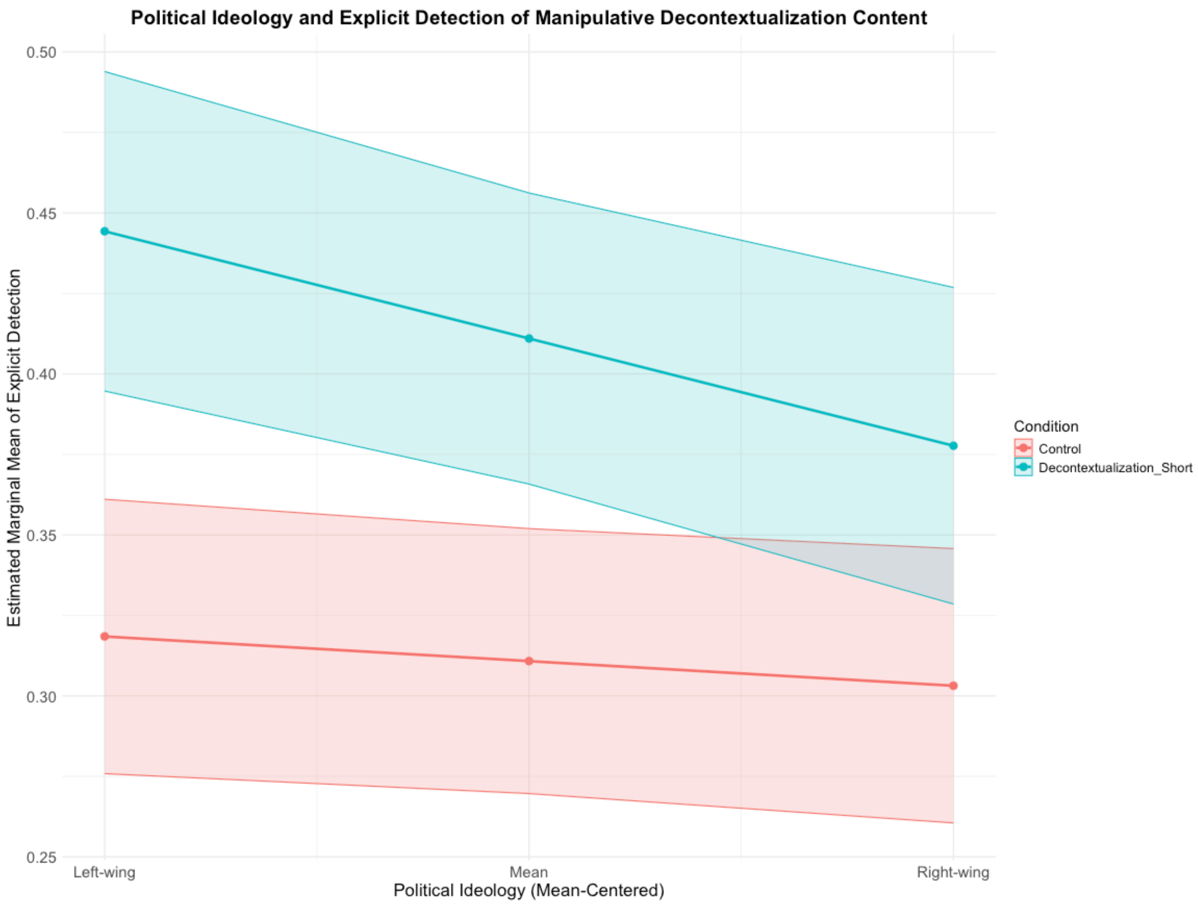

There was a significant interaction between the short decontextualization video (vs. control) and the education indices of nations when predicting technique recognition of the manipulative decontextualization content, $b = 1.59$, $SE = 0.57$, $t(18{,}530) = 2.78$, $p = .005$, such that the effect was only positive and significant among nations with higher, $b = 0.15$, *SE*

= 0.02, $p < .001$, and more moderate education indices, $b = 0.06$, $SE = 0.02$, $p = .009$, but not among nations with lower education indices, $b = -0.02$, $SE = 0.04$, $p = .999$ (see Figure S34).

Figure S34.

Simple Slopes Plot of the Interaction Effect between the Short Decontextualization Video (vs. Control) and Education Index when Predicting Technique Recognition of the Manipulative Decontextualization Content.

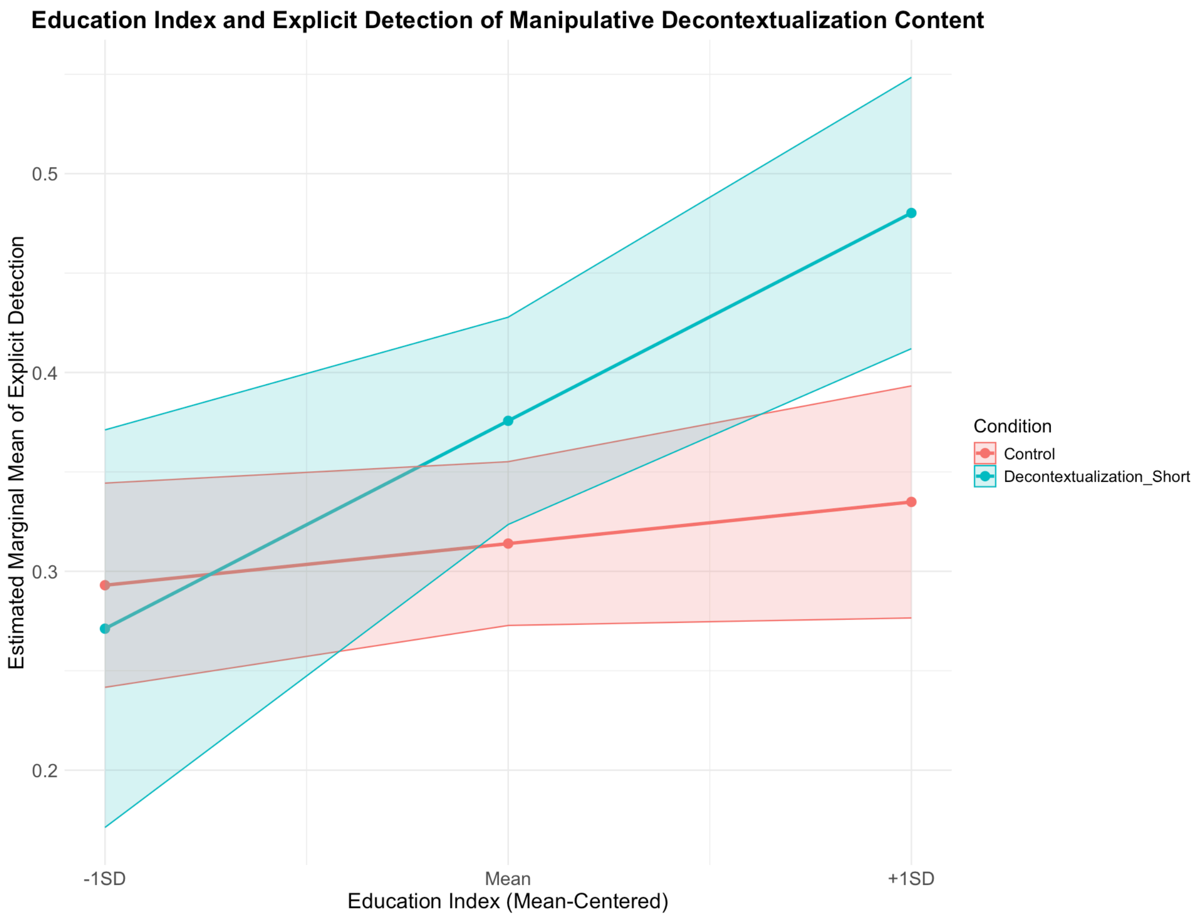

There were significant interactions between both the long, $b = 0.01$, $SE = 0.01$, $t(18{,}710) = 4.24$, $p < .001$, and short decontextualization videos (vs. control), $b = 0.01$, $SE = 0.01$, $t(18{,}550) = 3.05$, $p = .002$, and the GDP per capita the of nations when predicting technique recognition of the manipulative decontextualization content (see Figure S35). For the long decontextualization video (vs. control), the positive significant effect was strongest among nations with higher GDPs per capita, $b = 0.15$, $SE = 0.01$, $p < .001$, weaker among

nations with more moderate GDPs per capita, *b* = 0.12, *SE* = 0.01, *p* < .001, and weakest among nations with lower GDPs per capita, *b* = 0.09, *SE* = 0.01, *p* < .001 (see Figure S35). For the short decontextualization video (vs. control), the effect was only positive and significant among nations with higher GDPs per capita, *b* = 0.17, *SE* = 0.02, *p* < .001, but not among nations with more moderate, *b* = 0.05, *SE* = 0.02, *p* = .279, or lower GDPs per capita, *b* = -0.07, *SE* = 0.06, *p* = .999 (see Figure S35).

Figure S35.

Simple Slopes Plot of the Interaction Effects between Both the Long (Left) and Short (Right) Decontextualization Videos (vs. Control) and GDP Per Capita when Predicting Technique Recognition of the Manipulative Decontextualization Content.

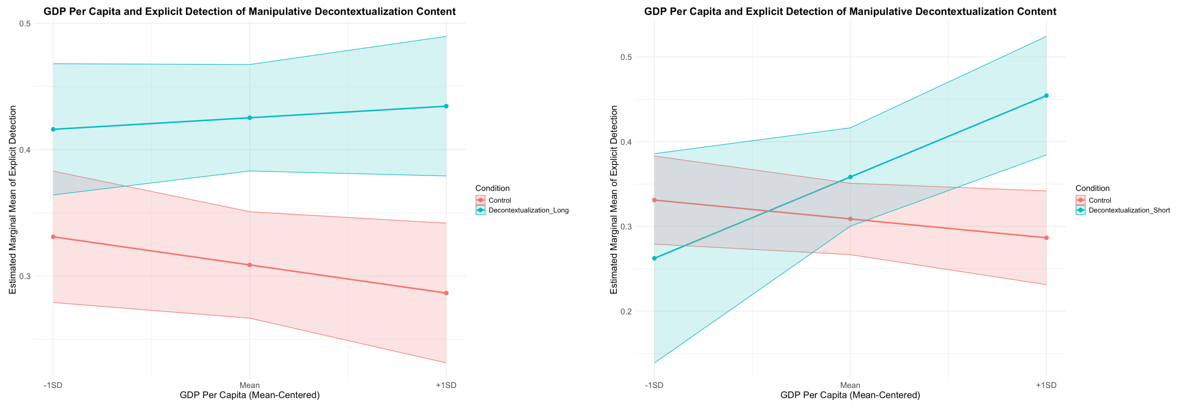

There was a significant interaction between the short decontextualization video (vs. control) and the democratic indices of nations when predicting technique recognition of the manipulative decontextualization content, *b* = 0.10, *SE* = 0.03, *t*(18,580) = 2.94, *p* = .003, such that the effect was only positive and significant among nations with higher, *b* = 0.16, *SE* = 0.02, *p* < .001, and more moderate democratic indices, *b* = 0.08, *SE* = 0.01, *p* < .001, but not among nations with lower democratic indices, *b* = 0.01, *SE* = 0.04, *p* = .999 (see Figure S36).

Figure S36.

Simple Slopes Plot of the Interaction Effect between the Short Decontextualization Video (vs. Control) and Democratic Index when Predicting Technique Recognition of the Manipulative Decontextualization Content.

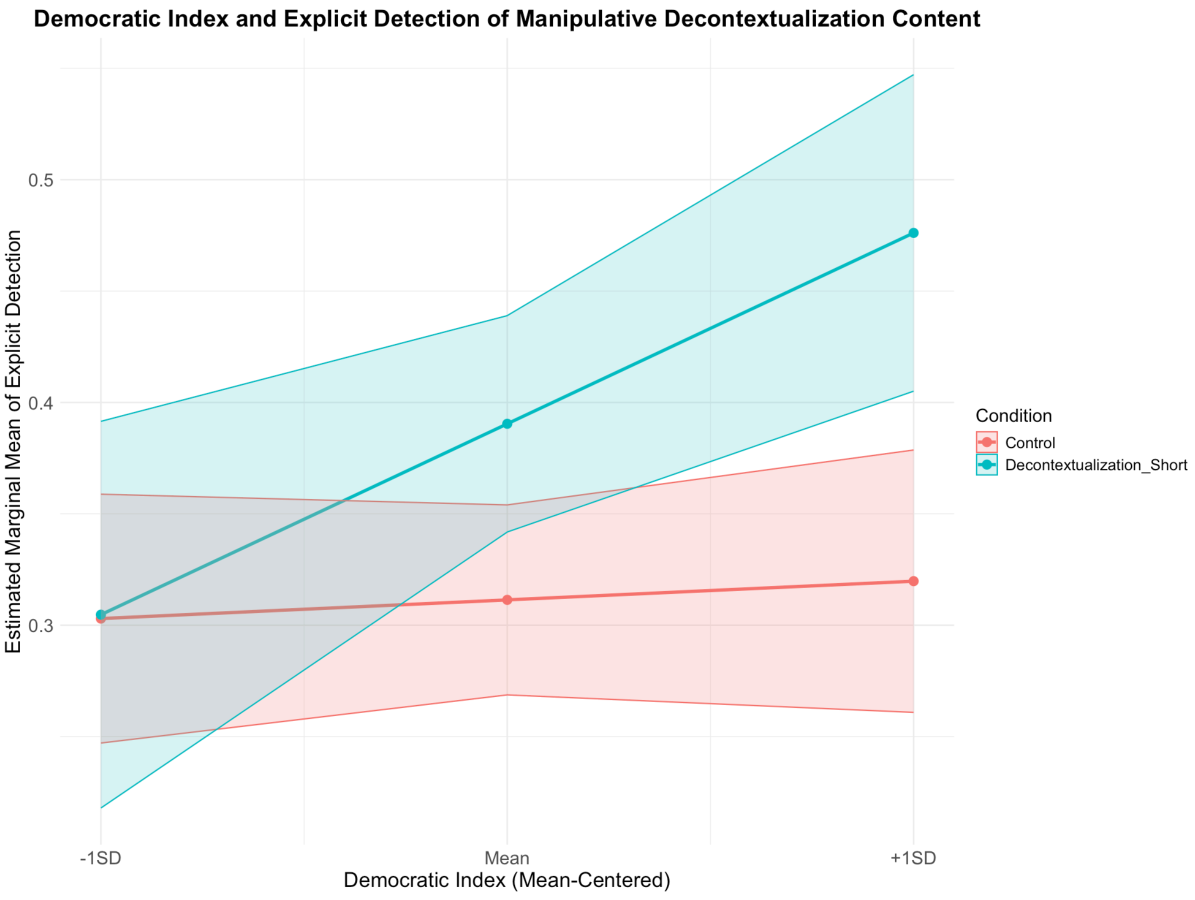

Technique recognition of non-manipulative decontextualization content

There was a significant interaction between the short decontextualization video (vs. control) and digital literacy when predicting technique recognition of the non-manipulative decontextualization content, b = 0.04, SE = 0.01, t(18,450) = 2.58, p = .010, such that the negative significant effect was strongest at lower levels of digital literacy, b = -0.12, SE = 0.02, p < .001, weaker at more moderate levels, b = -0.09, SE = 0.01, p < .001, and weakest and higher levels, b = -0.06, SE = 0.02, p = .010 (see Figure S37).

Figure S37.

Simple Slopes Plot of the Interaction Effect between the Short Decontextualization Video (vs. Control) and Digital Literacy when Predicting Technique Recognition of the Non-Manipulative Decontextualization Content.

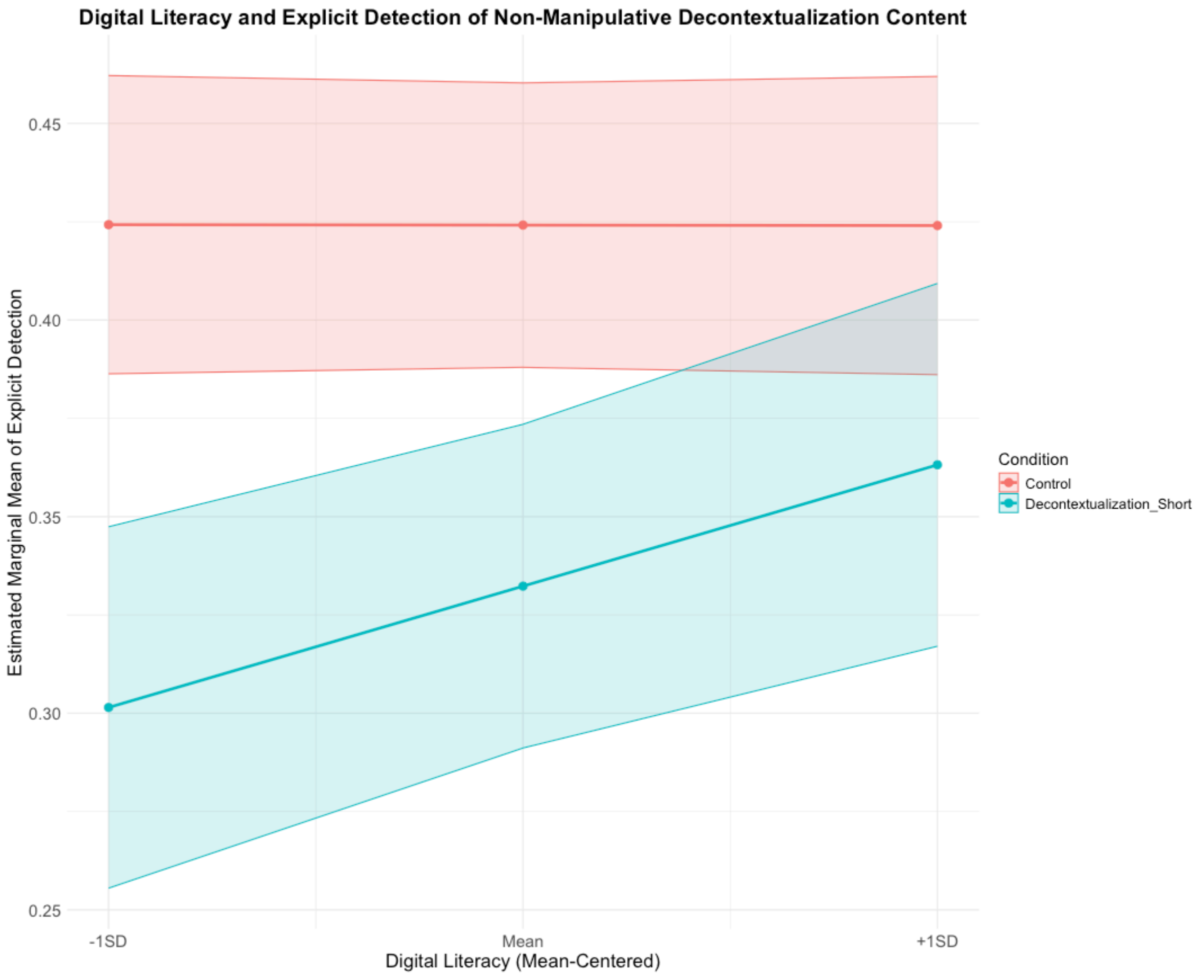

There was a significant interaction between the short decontextualization video (vs. control) and political tolerance when predicting technique recognition of the non-manipulative decontextualization content, $b = 0.03$, $SE = 0.01$, $t(18{,}380) = 2.00$, $p = .046$, such that the negative significant effect was strongest at lower levels of political tolerance, $b = -0.11$, $SE = 0.02$, $p < .001$, weaker at more moderate levels, $b = -0.09$, $SE = 0.01$, $p < .001$, and weakest and higher levels, $b = -0.07$, $SE = 0.02$, $p = .004$ (see Figure S38).

Figure S38.

Simple Slopes Plot of the Interaction Effect between the Short Decontextualization Video (vs. Control) and Political Tolerance when Predicting Technique Recognition of the Non-Manipulative Decontextualization Content.

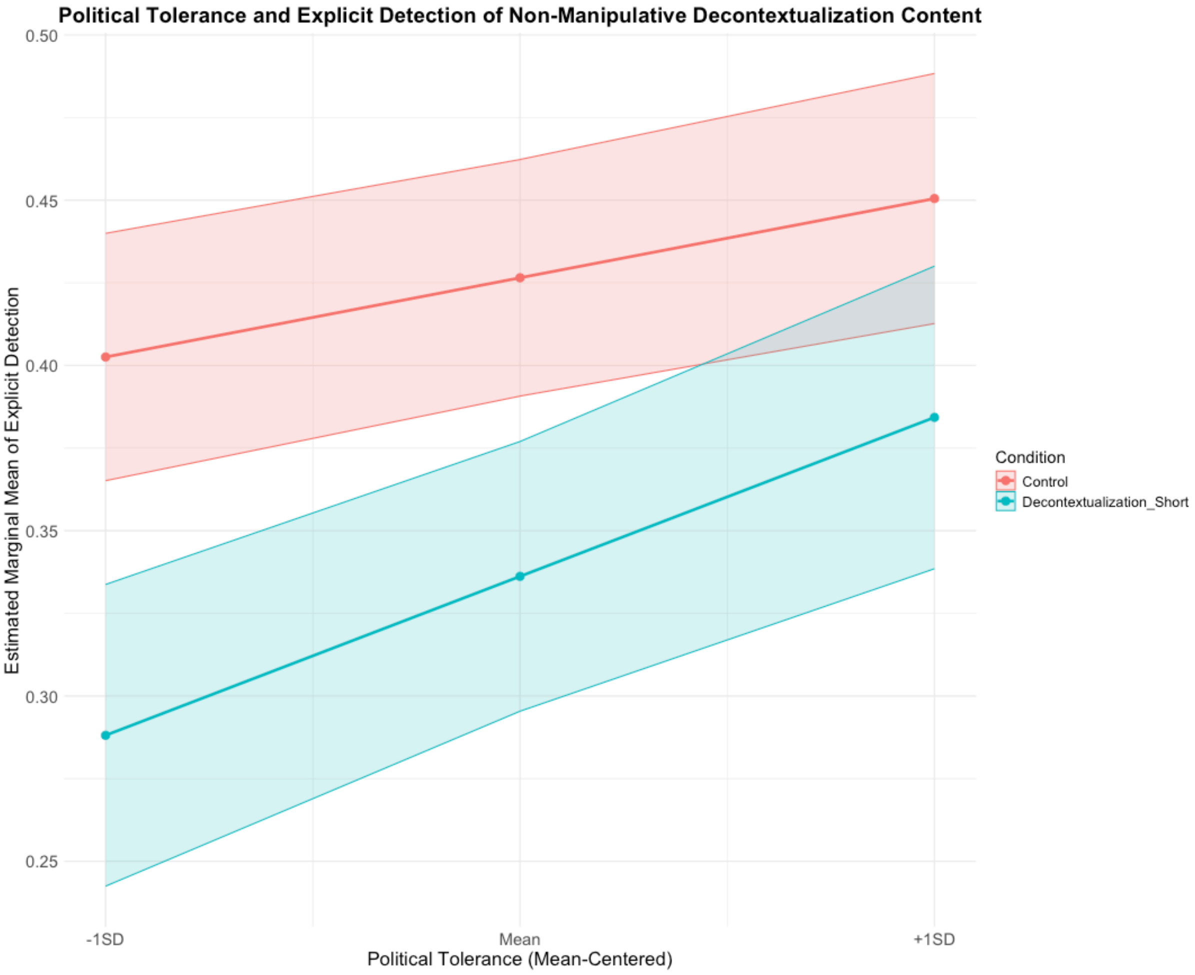

There was a significant interaction between the short decontextualization video (vs. control) and the education indices of nations when predicting technique recognition of the non-manipulative decontextualization content, b = -1.70, SE = 0.59, t(17,600) = 2.86, p = .004*,* such that the effect was only negative and significant among nations with higher education indices, b = -0.14, SE = 0.02, p < .001, but not among nations with more moderate, b = 0.05, SE = 0.02, p = .119, or lower education indices, b = 0.05, SE = 0.05, p = .999 (see Figure S39).

Figure S39.

Simple Slopes Plot of the Interaction Effect between the Short Decontextualization Video (vs. Control) and Education Index when Predicting Technique Recognition of the Non-Manipulative Decontextualization Content.

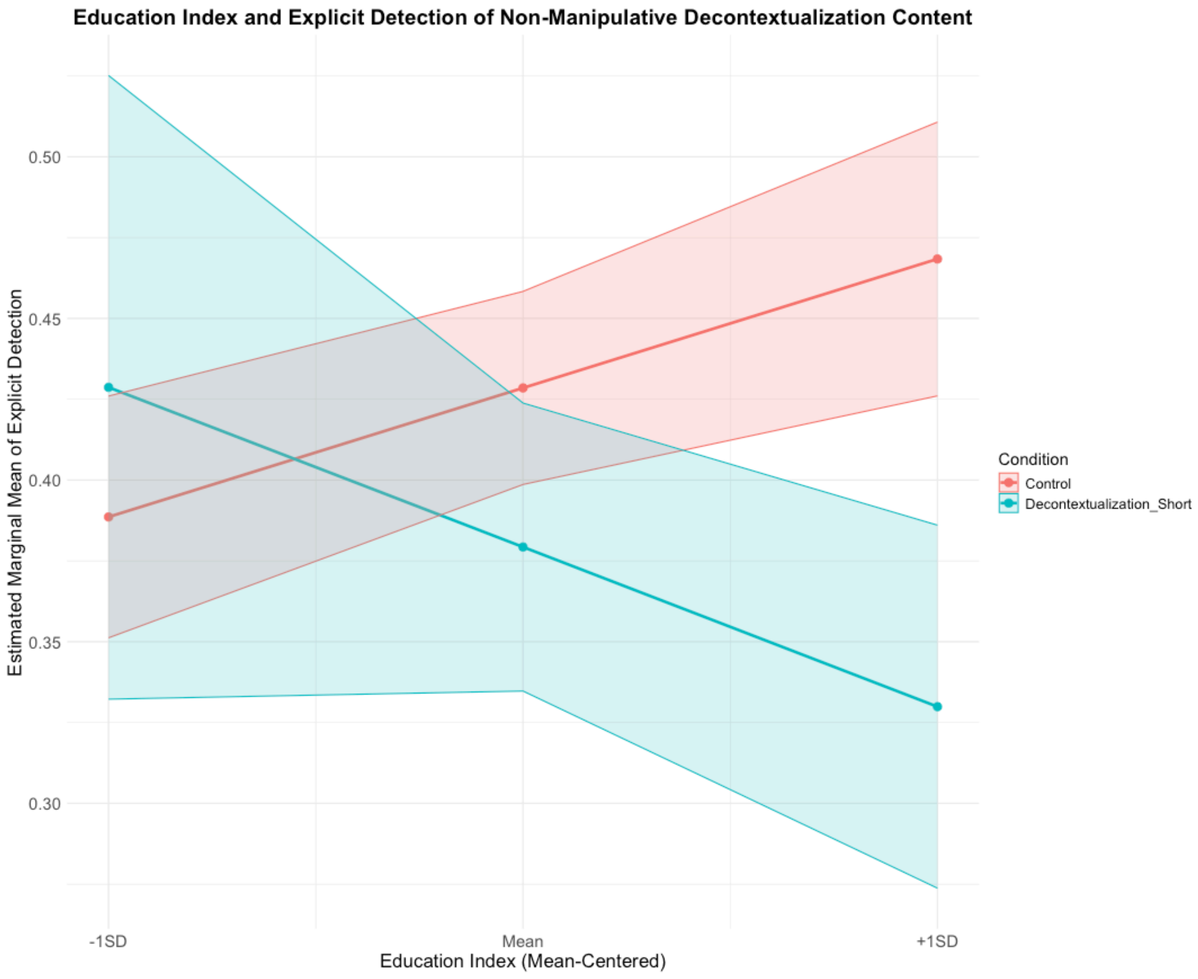

There was a significant interaction between the short decontextualization video (vs. control) and the industrialization indices of nations when predicting technique recognition of the non-manipulative decontextualization content, $b = -0.02$, $SE = 0.01$, $t(18{,}330) = 2.70$, $p = .007$, such that the effect was only negative and significant among nations with higher, $b = -0.23$, $SE = 0.05$, $p < .001$, and more moderate industrialization indices, $b = -0.12$, $SE = 0.02$, $p < .001$, but not among nations with lower industrialization indices, $b = -0.01$, $SE = 0.03$, $p = .999$ (see Figure S40).

Figure S40.

Simple Slopes Plot of the Interaction Effect between the Short Decontextualization Video (vs. Control) and Industrialization Index when Predicting Technique Recognition of the Non-Manipulative Decontextualization Content.

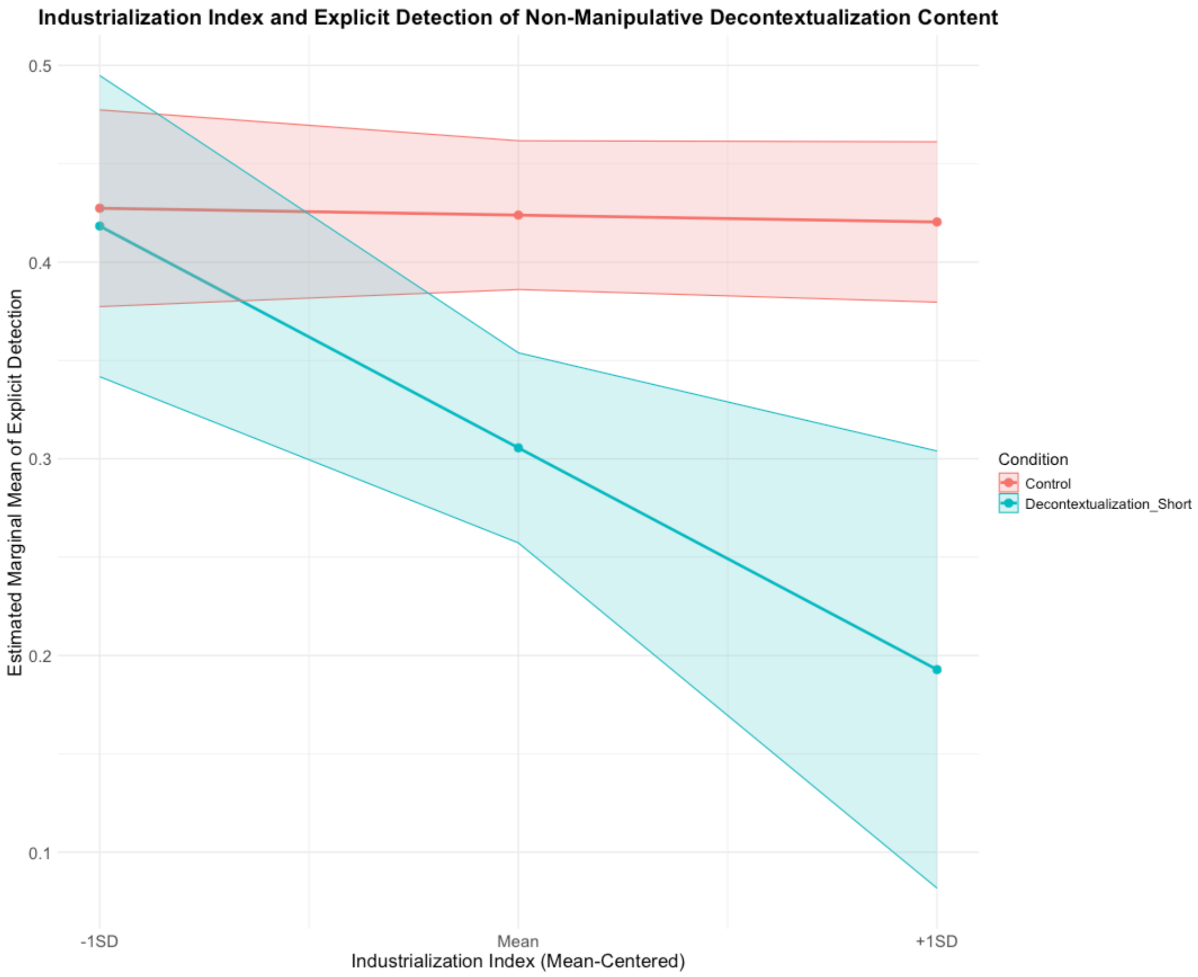

There were significant interactions between both the long, *b* = -0.01, *SE* = 0.01, *t*(18,710) = 2.45, *p* = .014, and short decontextualization videos (vs. control), *b* = -0.01, *SE* = 0.01, *t*(18,100) = 3.32, *p* < .001, and the GDP per capita of nations when predicting technique recognition of the non-manipulative decontextualization content (see Figure S41). For the long decontextualization video (vs. control), the negative significant effect was strongest at higher levels of GDP per capita, *b* = -0.09, *SE* = 0.01, *p* < .001, weaker at more moderate levels, *b* = -0.07, *SE* = 0.01, *p* < .001, and weakest at lower levels, *b* = -0.05, *SE* = 0.01, *p* < .001 (see Figure S41). For the short decontextualization video (vs. control), the effect was only negative and significant among nations with higher GDPs per capita, *b* =-0.16, *SE* =

0.03, p < .001, but not among nations with more moderate, b = -0.03, 0.02, p = .999, or lower GDPs per capita, b = 0.11, 0.06, p = .843(see Figure S41).

Figure S41.

Simple Slopes Plot of the Interaction Effects between Both the Long (Left) and Short (Right) Decontextualization Videos (vs. Control) and GDP Per Capita when Predicting Technique Recognition of the Non-Manipulative Decontextualization Content.

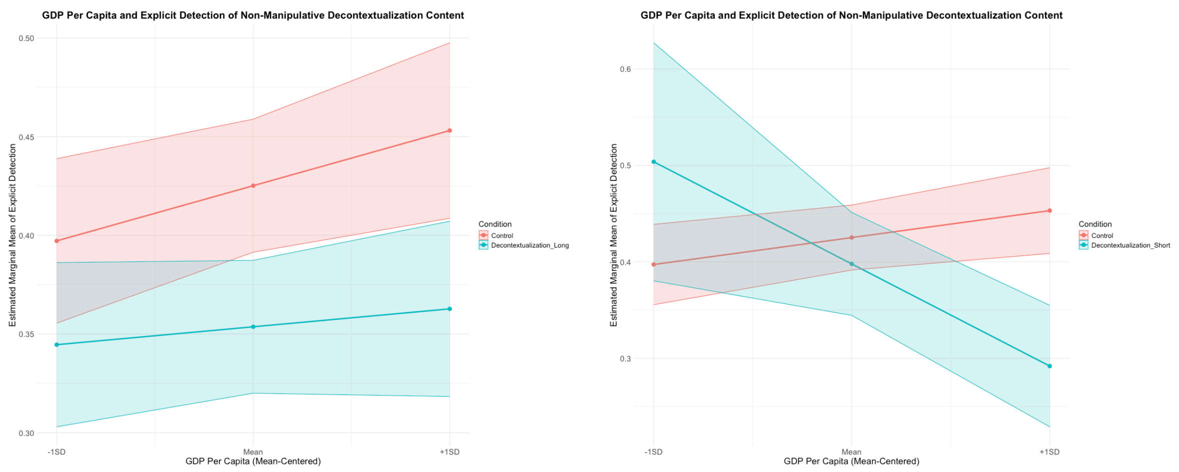

There was a significant interaction between the short decontextualization video (vs. control) and the democratic indices of nations when predicting technique recognition of the non-manipulative decontextualization content, b = -0.11, SE = 0.03, t(17,660) = 3.12, p = .002, such that the effect was only negative and significant among nations with higher, b = -0.15, SE = 0.02, p < .001, and more moderate democratic indices, b = -0.06, SE = 0.01, p < .001, but not among nations with lower democratic indices, b = 0.02, SE = 0.04, p = .999 (see Figure S42).

Figure S42.

Simple Slopes Plot of the Interaction Effect between the Short Decontextualization Video (vs. Control) and Democratic Index when Predicting Technique Recognition of the Non-Manipulative Decontextualization Content.

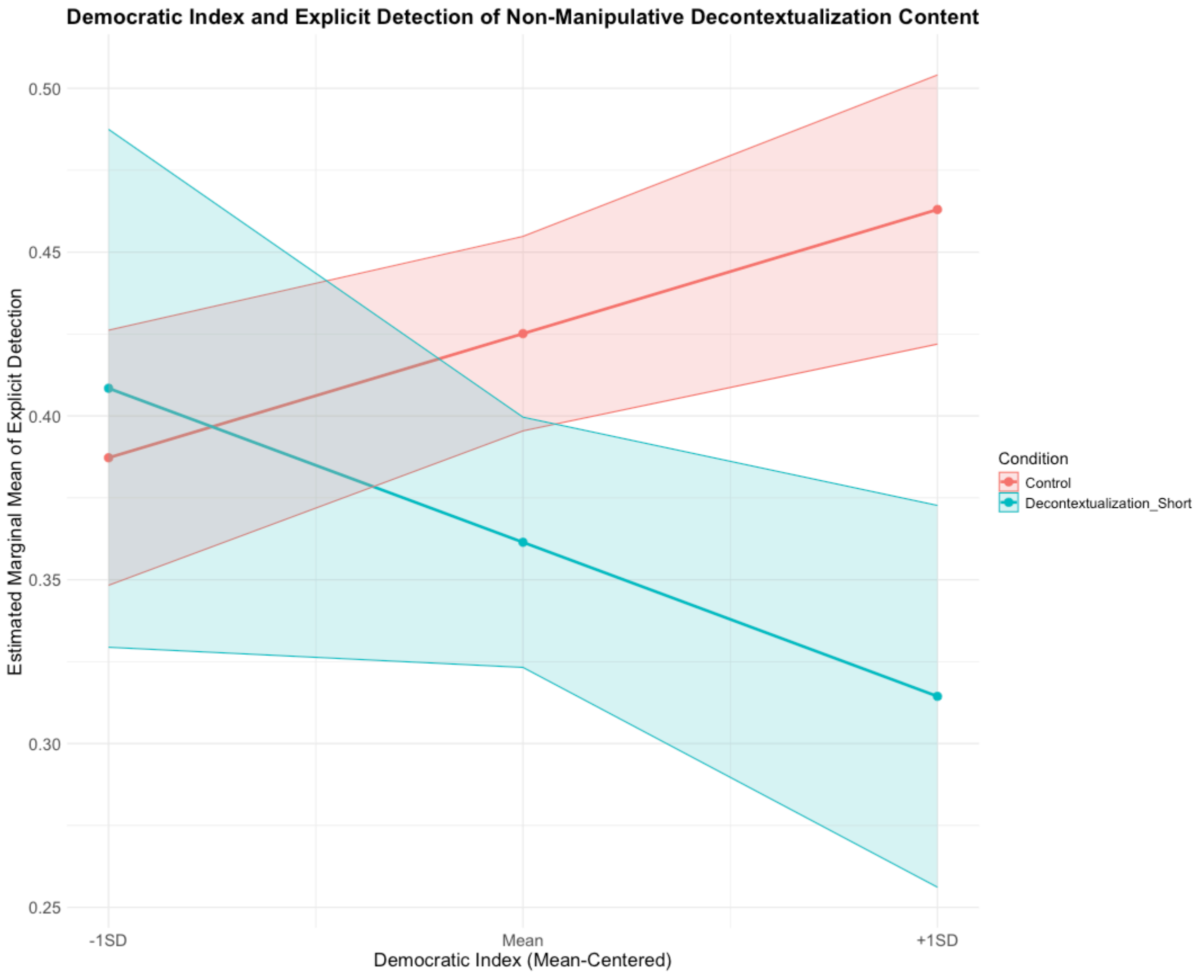

Technique discernment of decontextualization content

There was a significant interaction between the long decontextualization video (vs. control) and the education indices of nations when predicting technique discernment of the decontextualization content, b = -0.20, SE = 0.10, t(19,440) = 2.09, p = .036, such that the effect was only positive and significant among nations with lower, b = 0.03, SE = 0.01, p < .001, and more moderate education indices, b = 0.02, SE = 0.01, p = .007, but not among nations with higher education indices, b = 0.01, SE = 0.01, p = .999 (see Figure S43).

Figure S43.

Simple Slopes Plot of the Interaction Effect between the Long Decontextualization Video (vs. Control) and Education Index when Predicting Technique Discernment of the Decontextualization Content.

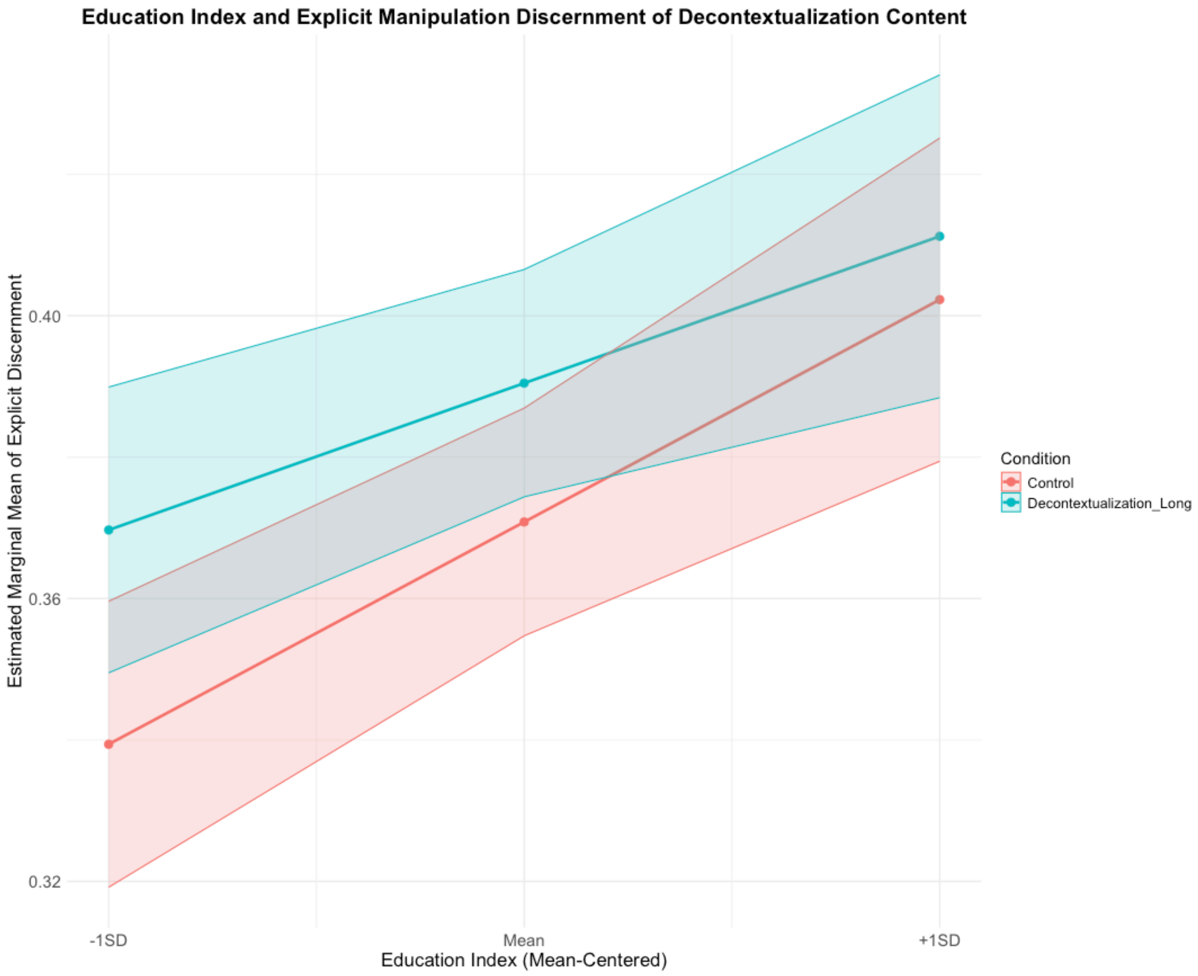

There were significant interactions between both the long, *b* = 0.03, *SE* = 0.00, *t*(18,710) = 6.51, *p* < .001, and short decontextualization videos (vs. control), *4*, and intentions to share the video within one's social network when predicting technique recognition of the manipulative decontextualization content (see Figure S44). For the long decontextualization video (vs. control), the effect was positive and significant at higher, *b* = 0.16, *SE* = 0.01, *p* < .001, more moderate, *b* = 0.11, *SE* = 0.01, *p* < .001, and lower levels of intentions to share, *b* = 0.05, *SE* = 0.01, *p* < .001. For the short decontextualization video (vs. control), the effect was positive and significant at higher, *b* = 0.15, *SE* = 0.02, *p* < .001, and more moderate levels of intentions to share, *b* = 0.10, *SE* = 0.01, *p* < .001, but not at lower levels, *b* = 0.05, *SE* = 0.02, *p* = .052.

Figure S44.

Simple Slopes Plots of the Interaction Effects between the Long (Left) and Short (Right) Decontextualization Videos (vs. Control) and Intentions to Share the Video Within One's Social Network when Predicting Technique Discernment of the Decontextualization Content.

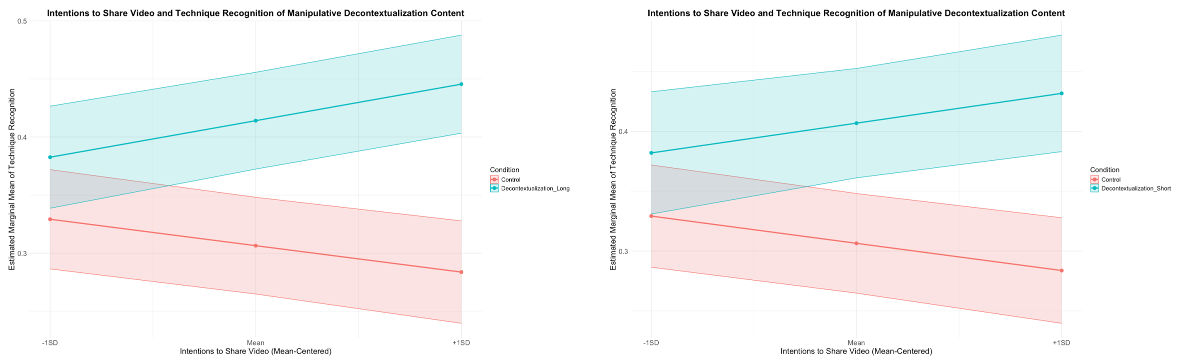

Willingness to share manipulative decontextualization content

There were significant interactions between both the long, $b = -0.12$, $SE = 0.02$, $t(18,710) = -6.42$, $p < .001$, and short decontextualization videos (vs. control), $b = -0.13$, $SE = 0.03$, $t(18,720) = -4.72$, $p < .001$, and intentions to share the video within one's social network when predicting willingness to share manipulative decontextualization content (see Figure S45). For the long decontextualization video (vs. control), the effect was negative and significant at higher, $b = -0.59$, $SE = 0.05$, $p < .001$, and more moderate levels of intentions to share, $b = -0.34$, $SE = 0.04$, $p < .001$, but not when intentions were lower, $b = 0.09$, $SE = 0.06$, $p = .959$. For the short decontextualization video (vs. control), the effect was negative and significant at higher, $b = -0.45$, $SE = 0.08$, $p < .001$, and more moderate levels of intentions to share, $b = -0.18$, $SE = 0.06$, $p = .030$, but not when intentions were lower, $b = -0.09$, $SE = 0.08$, $p = .999$.

Figure S45.

Simple Slopes Plots of the Interaction Effects between the Long (Left) and Short (Right) Decontextualization Videos (vs. Control) and Intentions to Share the Video Within One's Social Network when Predicting Willingness to Share the Manipulative Decontextualization Content.

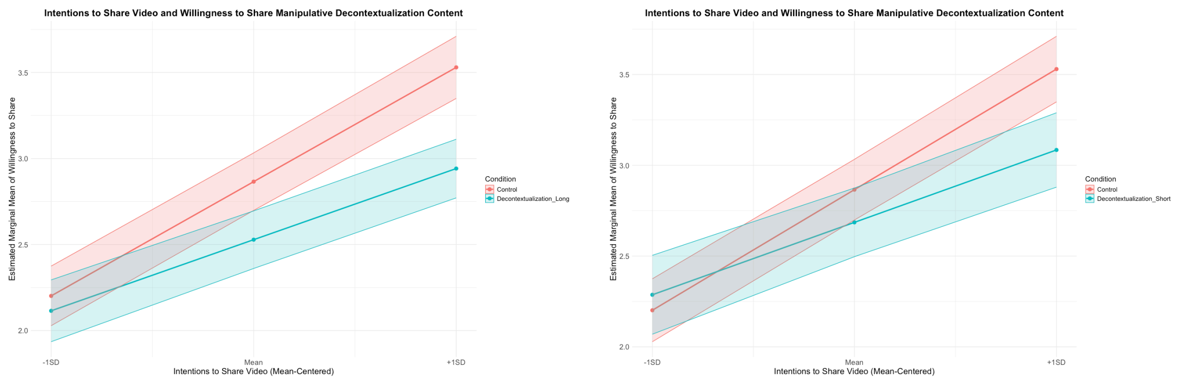

Willingness to share non-manipulative decontextualization content

There was a significant interaction between the long decontextualization video (vs. control) and the education indices of nations when predicting willingness to share the non-manipulative decontextualization content, b = 1.42, SE = 0.69, t(18,710) = 2.07, p = .039, such that the effect was only positive and significant among nations with higher, b = 0.21, SE = 0.06, p = .003, and more moderate education indices, b = 0.14, SE = 0.04, p = .007, but not among nations with lower education indices, b = 0.07, SE = 0.05, p = .999 (see Figure S46).

Figure S46.

Simple Slopes Plot of the Interaction Effect between the Long Decontextualization Video (vs. Control) and Education Index when Predicting Willingness to Share the Non-Manipulative Decontextualization Content.

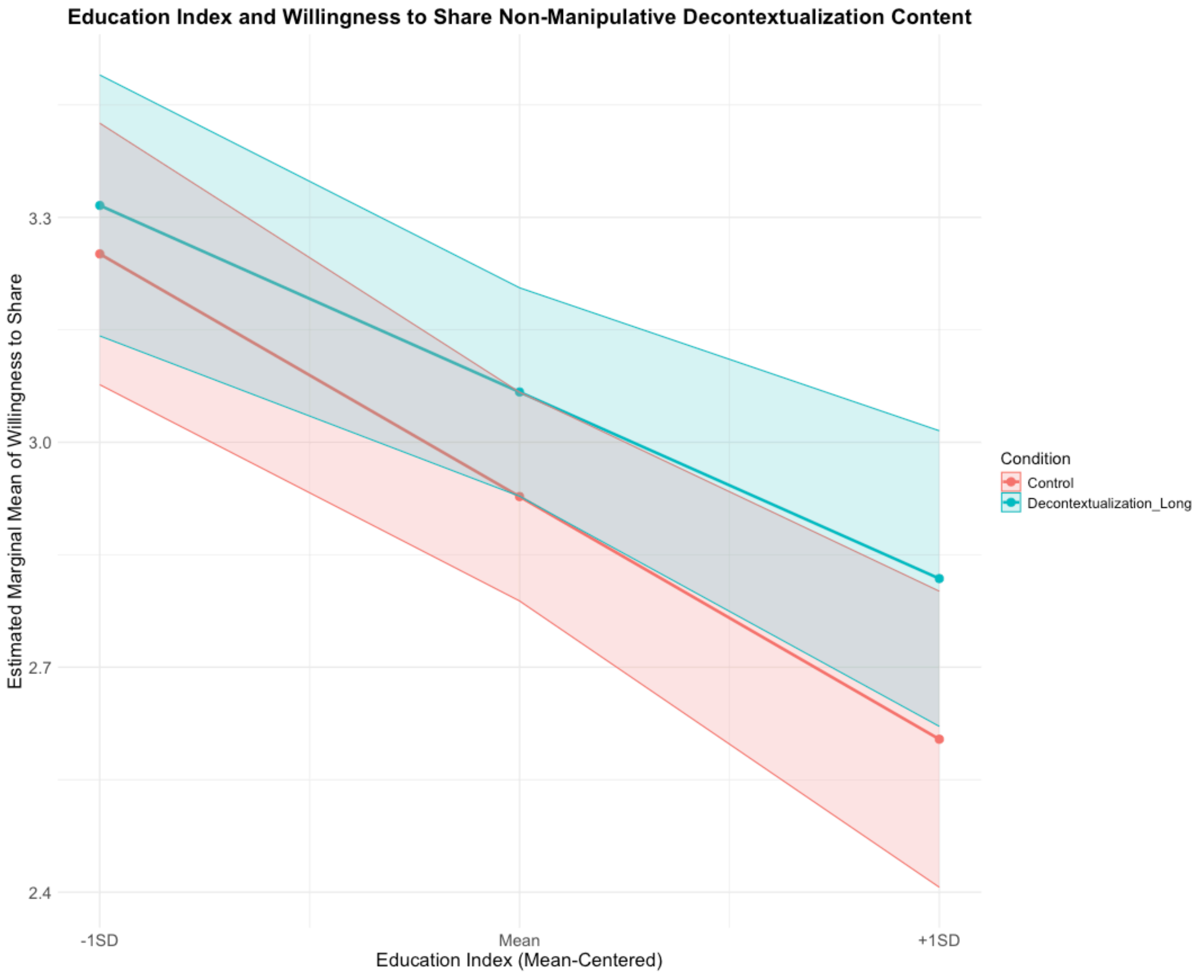

There was a significant interaction between the long decontextualization video (vs. control) and the democratic indices of nations when predicting willingness to share the non-manipulative decontextualization content, $b = 0.09$, $SE = 0.05$, $t(18{,}710) = 1.98$, $p = .048$, such that the effect was only positive and significant among nations with higher, $b = 0.21$, $SE = 0.05$, $p = .003$, and more moderate democratic indices, $b = 0.14$, $SE = 0.04$, $p = .009$, but not among nations with lower democratic indices, $b = 0.07$, $SE = 0.05$, $p = .999$ (see Figure S47).

Figure S47.

Simple Slopes Plot of the Interaction Effect between the Long Decontextualization Video (vs. Control) and Democratic Index when Predicting Willingness to Share the Non-Manipulative Decontextualization Content.

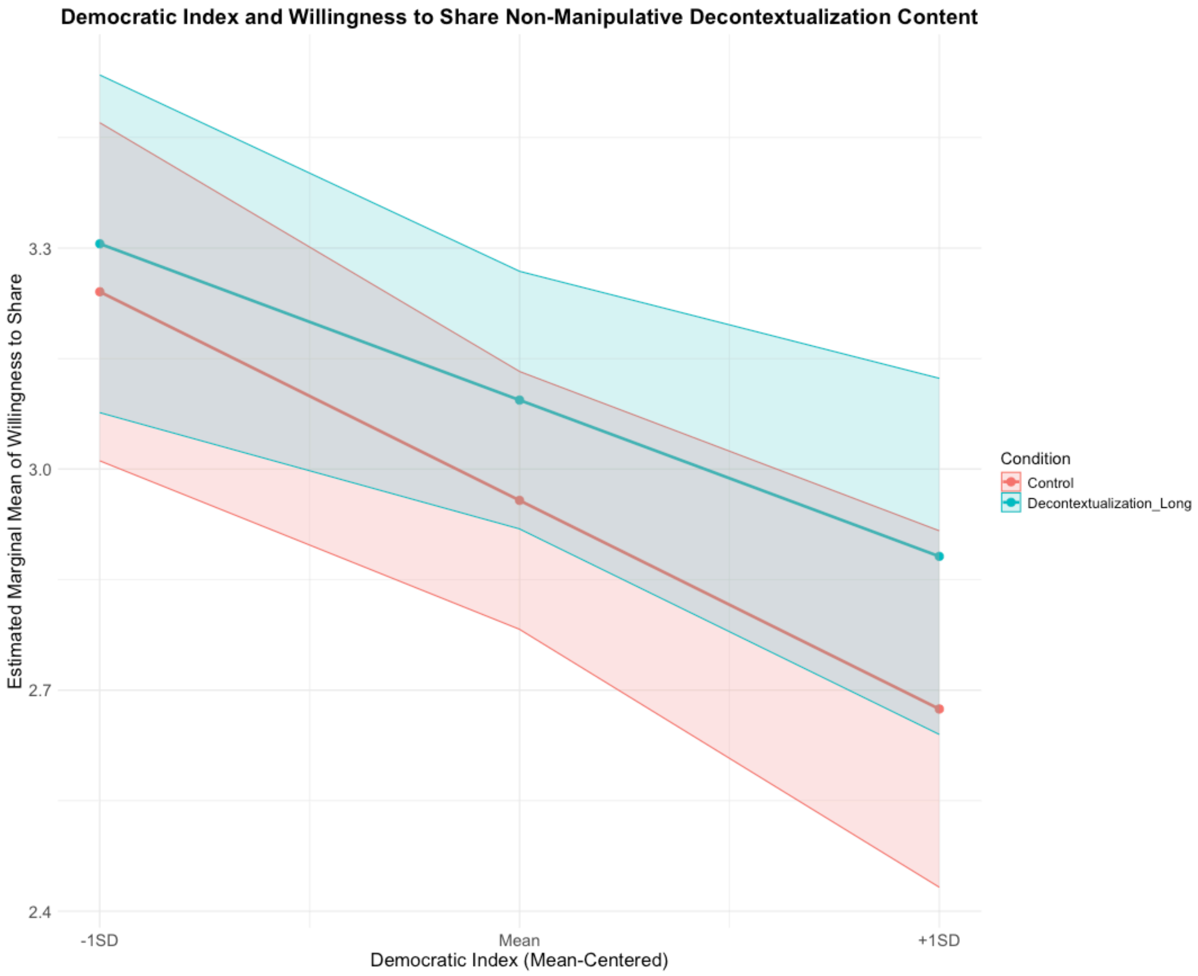

There was a significant interaction between the long decontextualization video (vs. control) and intentions to share the video within one's social network when predicting willingness to share non-manipulative decontextualization content, b = -0.06, SE = 0.02, t(18,720) = -3.14, p = .002 (see Figure S48). The effect of the long decontextualization video (vs. control) was negative and significant at higher, b = -0.34, SE = 0.05, p < .001, and more moderate levels of intentions to share, b = -0.22, SE = 0.04, p < .001, but not when intentions were lower, b = -0.10, SE = 0.05, p = .765.

Figure S48.

Simple Slopes Plot of the Interaction Effect between the Long Decontextualization Video (vs. Control) and Intentions to Share the Video Within One' Social Network when Predicting Willingness to Share the Non-Manipulative Decontextualization Content.

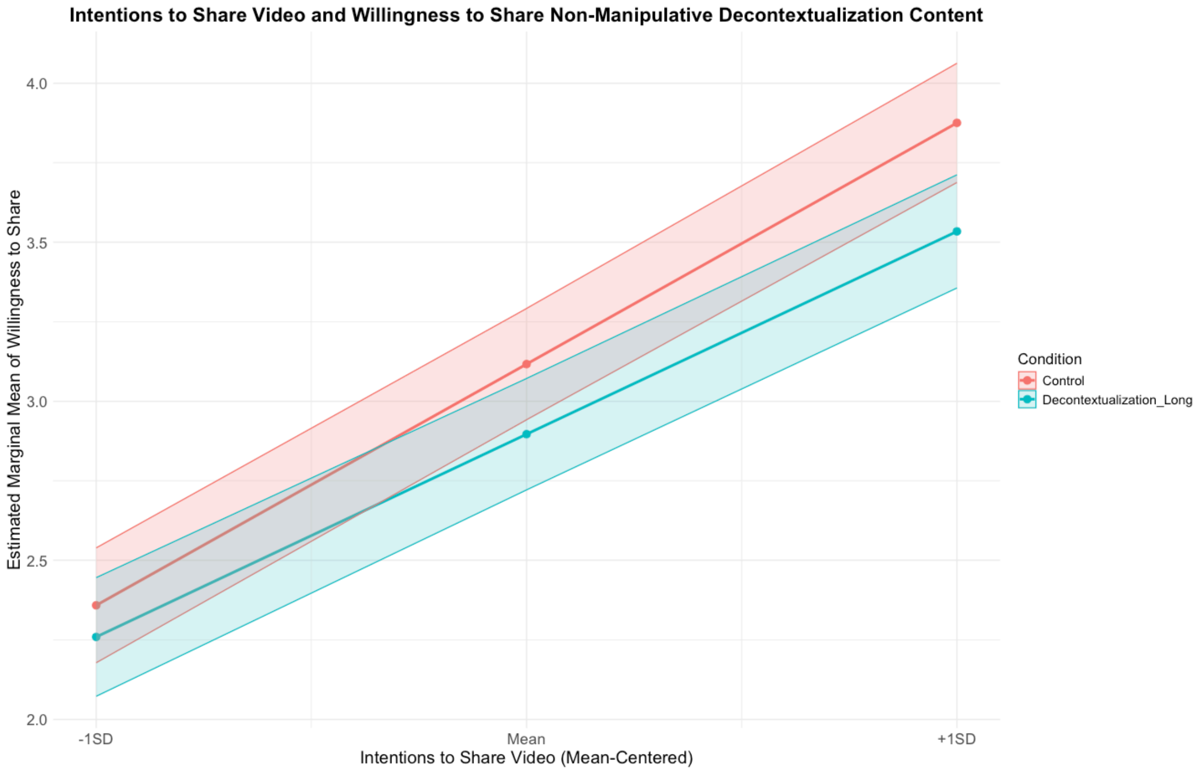

There was a significant interaction between the long decontextualization video (vs. control) and intentions to share the video within one's social network when predicting sharing decisions for the decontextualization content, $b = 0.07$, $SE = 0.01$, $t(17{,}970) = 4.79$, $p < .001$ (see Figure S49). The effect of the long decontextualization video (vs. control) on sharing decisions for the decontextualization content was positive and significant at higher, $b = 0.26$, $SE = 0.04$, $p < .001$, and more moderate intentions to share, $b = 0.12$, $SE = 0.03$, $p < .001$, but not when intentions to share were lower, $b = 0.02$, $SE = 0.04$, $p = .999$.

Figure S49.

Simple Slopes Plot of the Interaction Effect between the Long Decontextualization Video (vs. Control) and Intentions to Share the Video Within One' Social Network when Predicting Sharing Decisions for the Decontextualization Content.

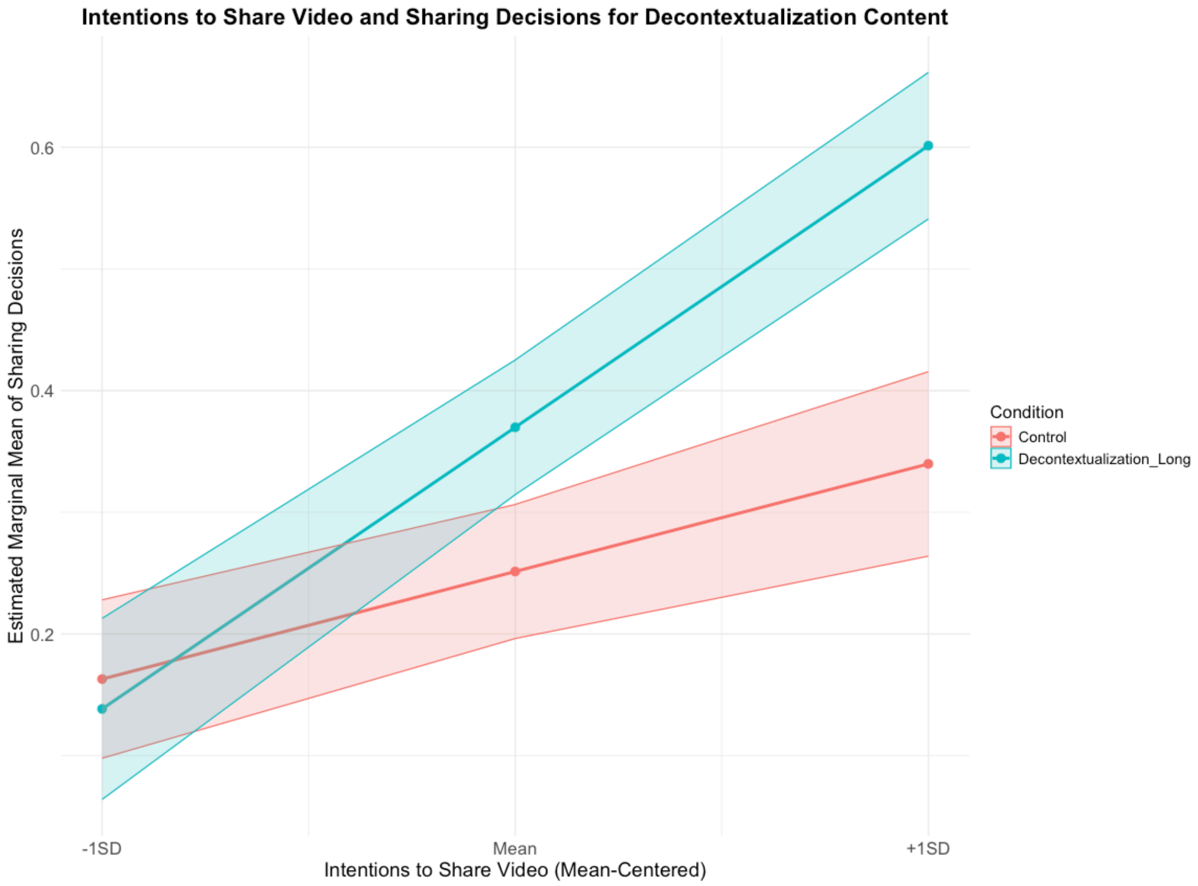

Discrediting videos

Manipulativeness assessments of manipulative discrediting content

There was a significant interaction between the long discrediting video (vs. control) and political tolerance when predicting manipulativeness assessments of the manipulative discrediting content, b = -0.09, SE = 0.05, t(17,970) = 2.02, p = .044, such that the effect was only positive and significant at lower, b = 0.33, SE = 0.06, p < .001, and more moderate levels of political tolerance, b = 0.24, SE = 0.04, p < .001, but not at higher levels, b = 0.15, SE = 0.06, p = .240 (see Figure S50). Furthermore, there was a significant interaction between the long discrediting video (vs. control) and intentions to share the video within one's social network, b = 0.06, SE = 0.02, t(18,660) = 2.67, p = .008, such that the effect was only positive and significant at higher, b = 0.35, SE = 0.06, p < .001, and more moderate levels of intentions to share, b = 0.23, SE = 0.04, p < .001, but not at lower levels, b = 0.11, SE = 0.06, p = .840 (see Figure S50).

Figure S50.

Simple Slopes Plot of the Interaction Effect between the Long Discrediting Video (vs. Control) and both Political Tolerance (Left) and Intentions to Share the Video Withtin On'es Social Circle (Right) when Predicting Manipulativeness Assessments of the Manipulative Discrediting Content.

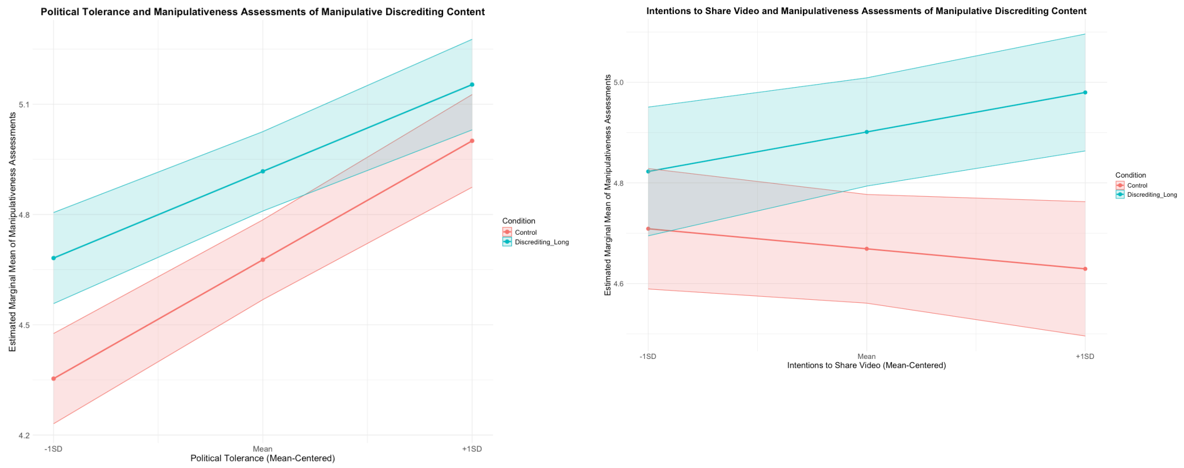

There was a significant interaction between the long discrediting video (vs. control) and gender when predicting manipulativeness assessments of the manipulative discrediting content, $b = -0.21$, $SE = 0.09$, $t(18{,}200) = -2.38$, $p = .018$, such that the effect was only positive and significant among women, $b = 0.35$, $SE = 0.06$, $p < .001$, but not men, $b = 0.14$, $SE = 0.06$, $p = .375$ (see Figure S51).

Figure S51.

Simple Slopes Plot of the Interaction Effect between the Long Discrediting Video (vs. Control) and Gender when Predicting Manipulativeness Assessments of the Manipulative Discrediting Content.

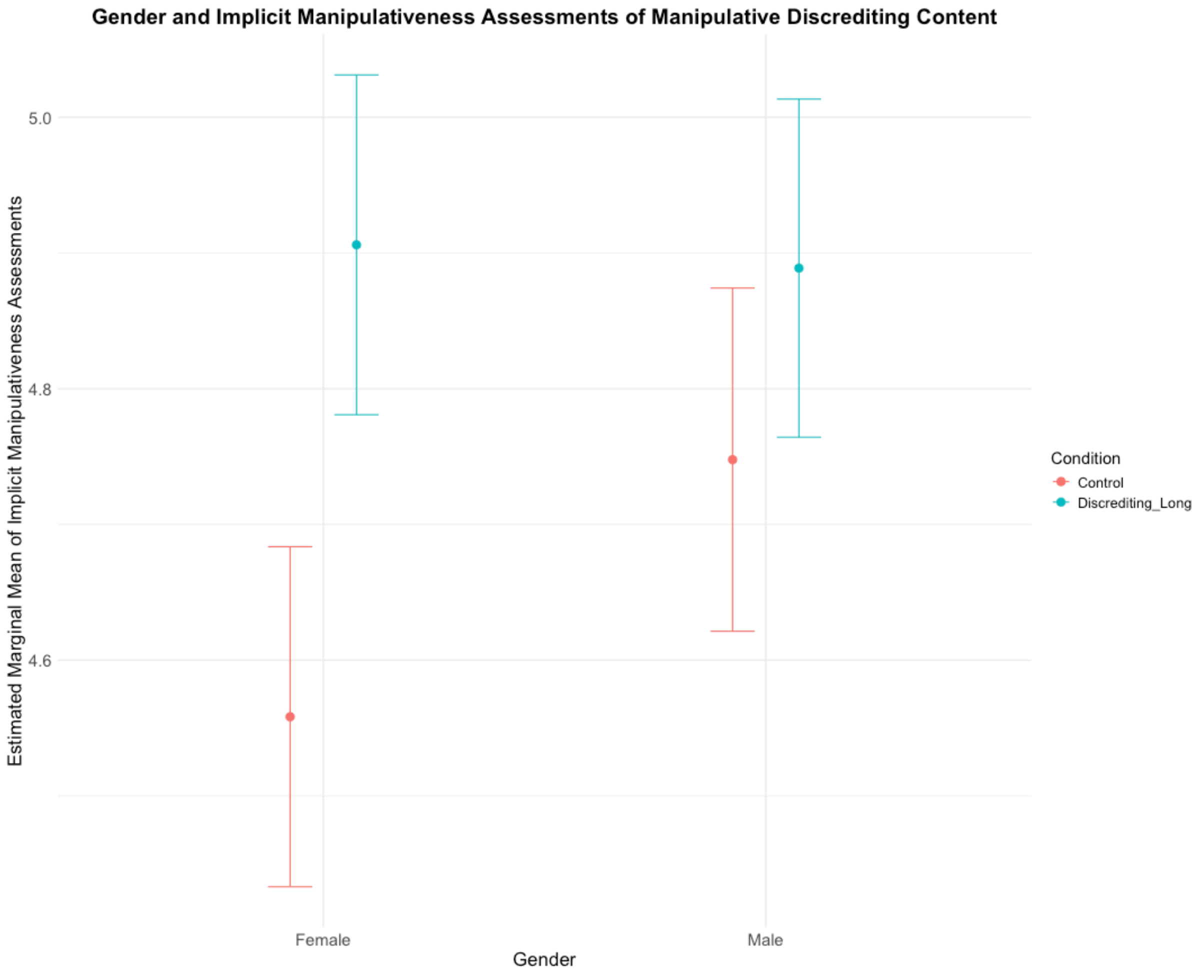

Manipulativeness assessments of non-manipulative discrediting content

There were significant interactions between both the long, $b = 0.09$, $SE = 0.02$, $t(17{,}460) = 5.31$, $p < .001$, and short, $b = 0.12$, $SE = 0.03$, $t(18{,}390) = 4.58$, $p < .001$, discrediting videos (vs. control) and intentions to share the video within one's social network when predicting manipulativeness assessments of the non-manipulative discrediting content. For the long discrediting video (vs. control), this effect was only negative and significant when intentions to share were higher, $b = -0.32$, $SE = 0.05$, $p < .001$, or more moderate, $b = -0.12$, $SE = 0.04$, $p = .025$, but not at lower levels, $b = 0.08$, $SE = 0.05$, $p = .999$ (see Figure S*52*). For the short discrediting video (vs. control), this effect was only negative and significant when intentions to share were higher, $b = -0.38$, $SE = 0.09$, $p < .001$, but not when they were more moderate, $b = -0.13$, $SE = 0.06$, $p = .436$, or lower, $b = 0.13$, $SE = 0.07$, $p = .999$ (see Figure S52).

Figure S52.

Simple Slopes Plot of the Interaction Effects between the Long (Left) and Short (Right) Discrediting Video (vs. Control) and Intentions to Share the Video Within One's Social Network when Predicting Manipulativeness Assessments of the Non-Manipulative Discrediting Content.

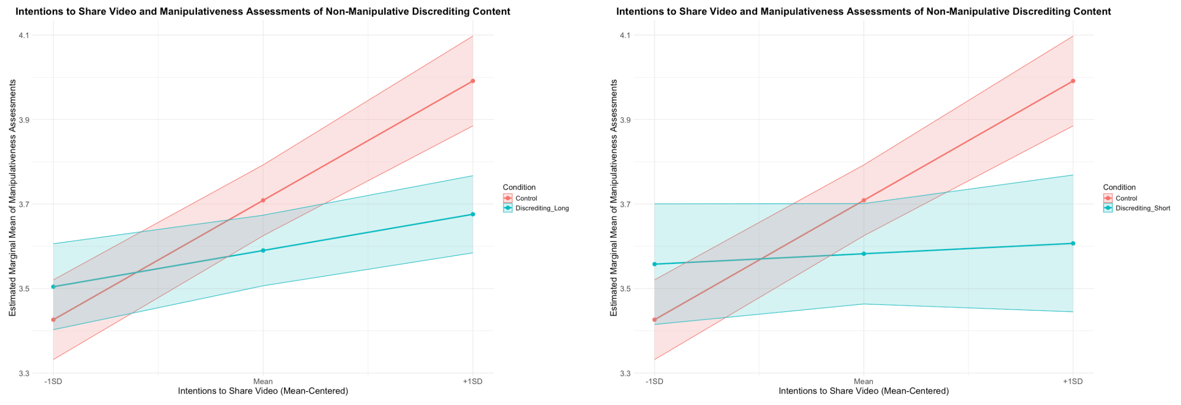

Manipulation discernment of discrediting content

There was a significant interaction between the short discrediting video (vs. control) and the education indices of nations when predicting manipulation discernment of the discrediting content, b = -8.28, SE = 4.13, t(9,943) = -2.00, p = .045, such that the effect was only positive and significant among nations with more moderate education indices, b = 0.41, SE = 0.13, p = .026, but not among nations with lower, b = 0.85, SE = 0.32, p = .147, or higher education indices, b = -0.02, SE = 0.15, p = .999 (see Figure S53).

Figure S53.

Simple Slopes Plot of the Interaction Effect between the Short Discrediting Video (vs. Control) and Education Index when Predicting Manipulation Discernment of the Discrediting Content.

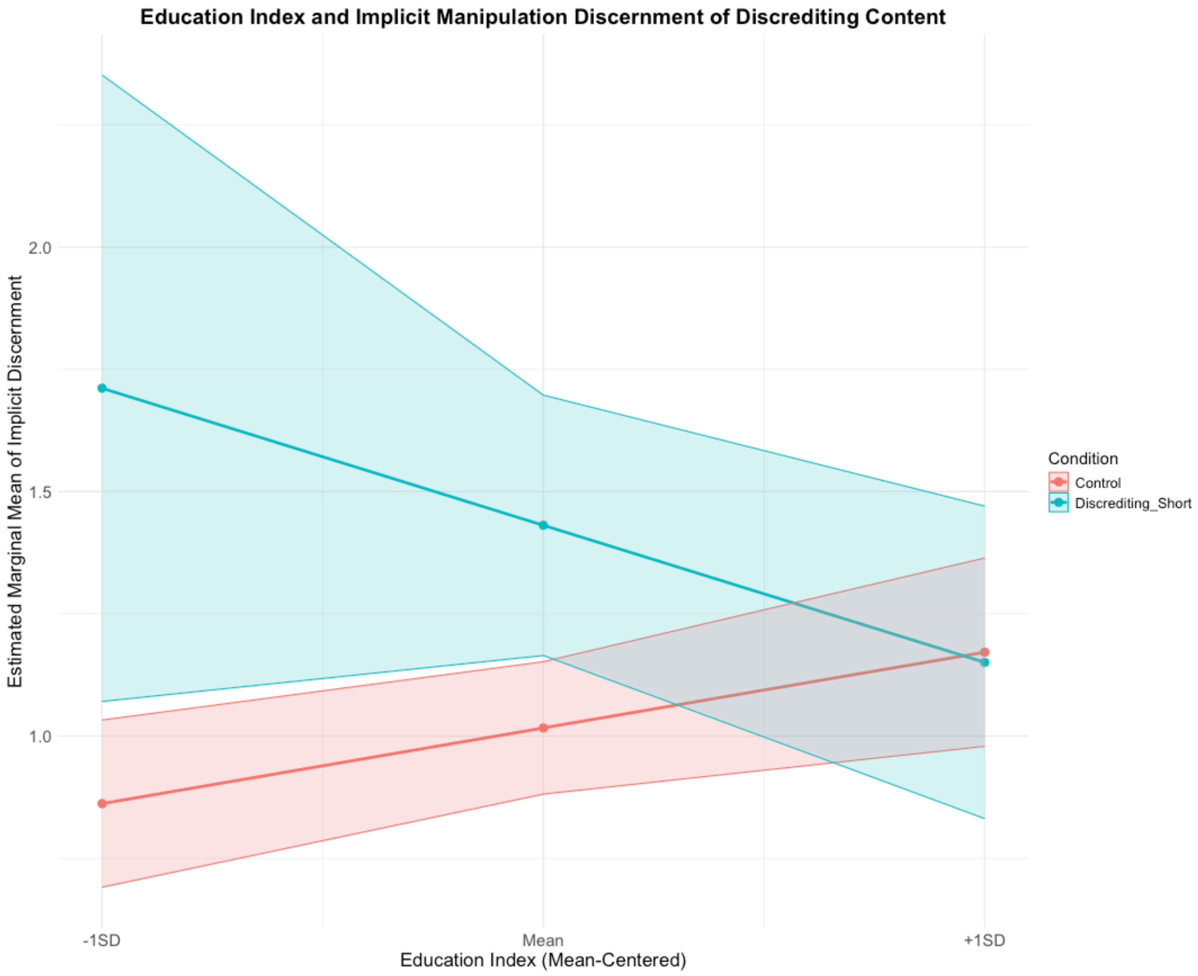

There was a significant interaction between the long discrediting video (vs. control) and the GDP per capita of nations when predicting manipulation discernment of the discrediting content, $b = -0.01$, $SE = 0.01$, $t(17{,}490) = -2.15$, $p = .032$, such that the effect was only positive and significant among nations with lower, $b = 0.41$, $SE = 0.08$, $p < .001$, and more moderate GDPs per capita, $b = 0.29$, $SE = 0.06$, $p < .001$, but not among nations with higher GDPs per capita, $b = 0.17$, $SE = 0.08$, $p = .765$ (see Figure S54).

Figure S54.

Simple Slopes Plot of the Interaction Effect between the Long Discrediting Video (vs. Control) and GDP Per Capita when Predicting Manipulation Discernment of the Discrediting Content.

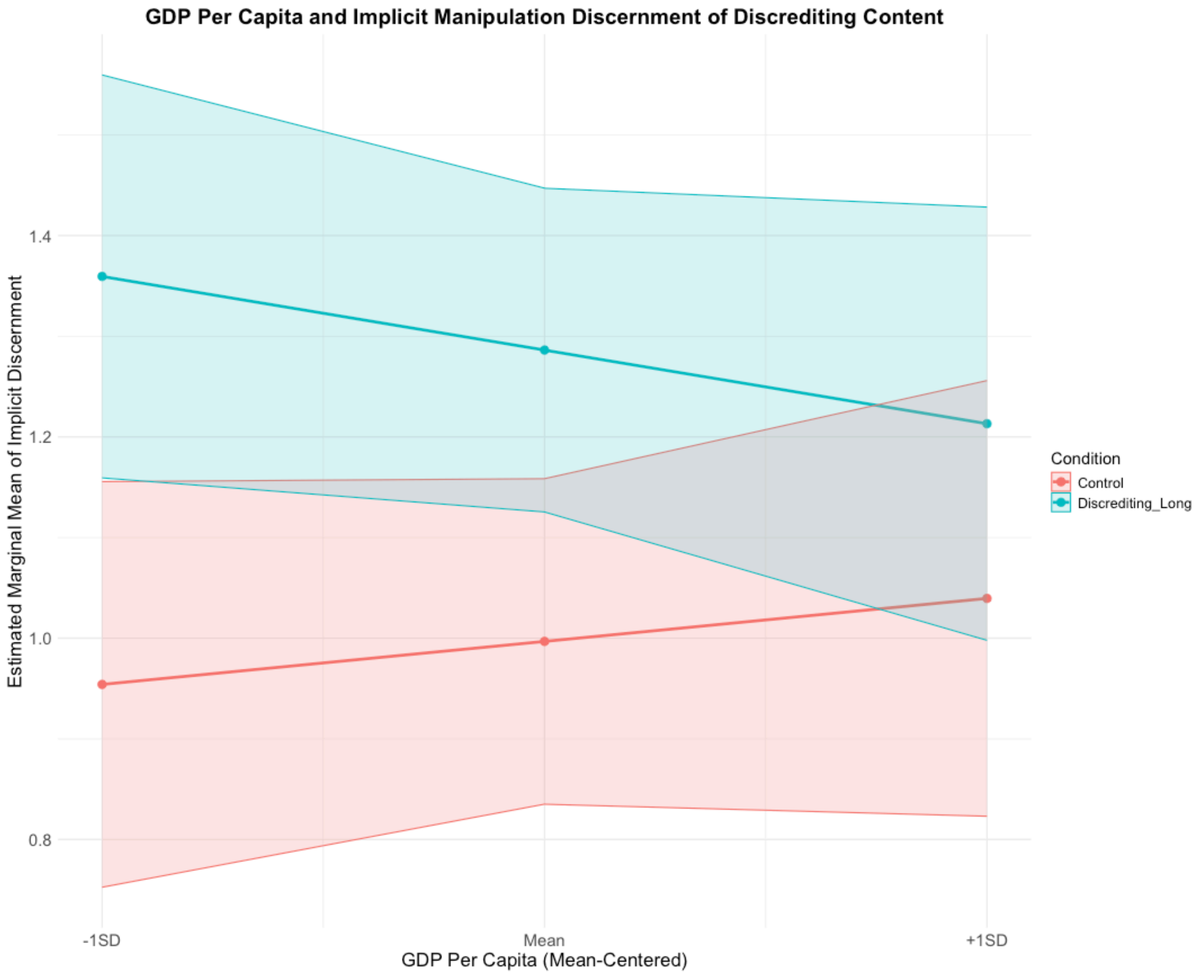

There was a significant interaction between the short discrediting video (vs. control) and age when predicting manipulation discernment of the discrediting content, b = -0.021, SE = 0.010, t(17,490) = -2.05, p = .040, such that the effect was only positive and significant among younger participants, b = 0.41, SE = 0.12, p = .018, but not among participants whose age was closer to the mean, b = 0.24, SE = 0.09, p = .135, or older participants, b = 0.06, SE = 0.12, p = .999 (see Figure S55).

Figure S55.

Simple Slopes Plot of the Interaction Effect between the Short Discrediting Video (vs. Control) and Age when Predicting Manipulation Discernment of the Discrediting Content.

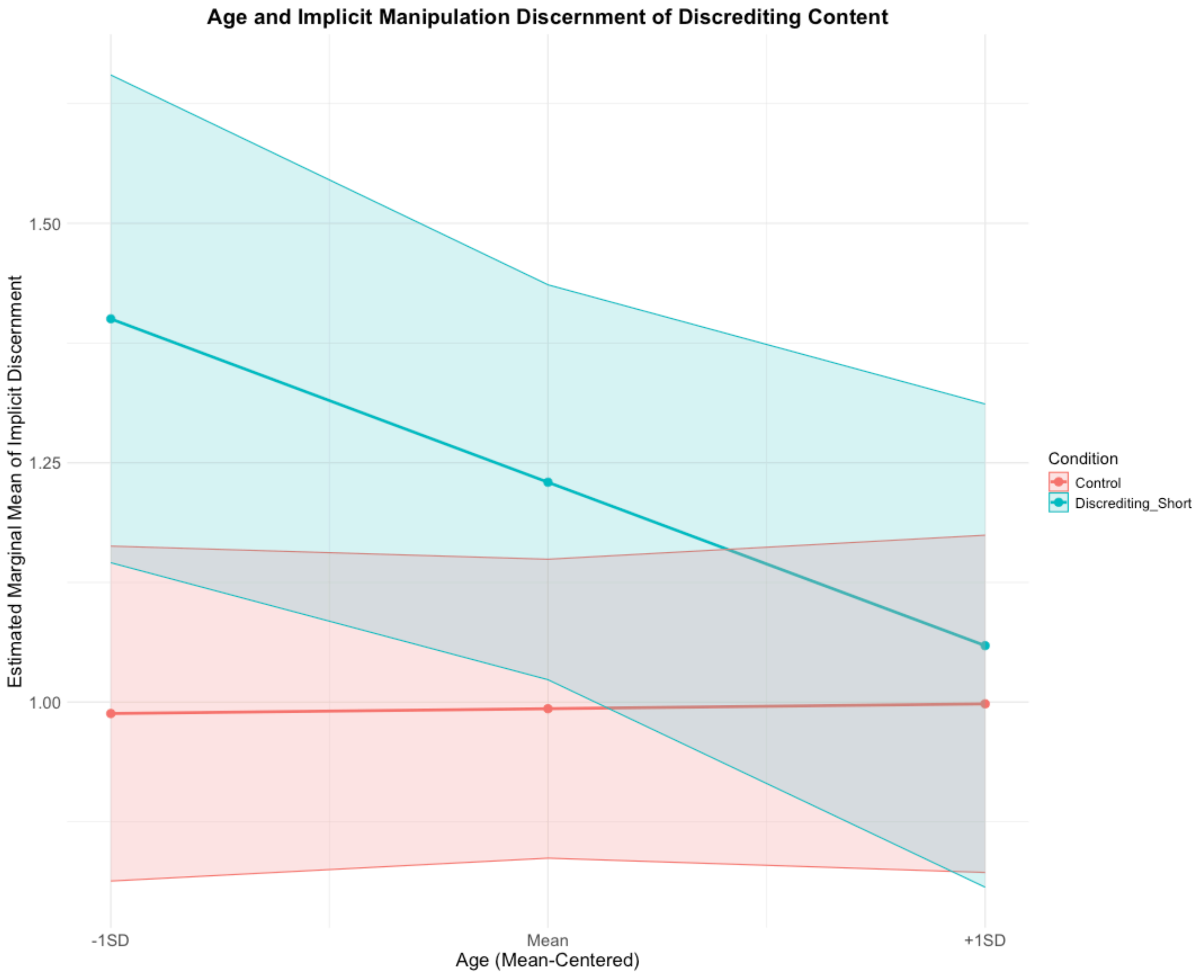

There were significant interactions between both the long, $b = 0.15$, $SE = 0.03$, $t(17{,}880) = 5.28$, $p < .001$, and short discrediting videos (vs. control), $b = 0.15$, $SE = 0.04$, $t(17{,}750) = 3.62$, $p < .001$, and intentions to share the video within one's social network when predicating manipulation discernment of the discrediting content (see Figure S56). For the long discrediting video (vs. control), the effect was only positive and significant at higher, $b = 0.67$, $SE = 0.08$, $p < .001$, or more moderate levels of intentions to share, $b = 0.36$, $SE = 0.06$, $p < .001$, but not when intentions were lower, $b = 0.04$, $SE = 0.08$, $p = .999$. Similarly, for the short discrediting video (vs. control), the effect was only positive and significant at higher, $b = 0.64$, $SE = 0.14$, $p < .001$, or more moderate levels of intentions to share, $b = 0.31$, $SE = 0.09$, $p = .011$, but not when intentions were lower, $b = -0.02$, $SE = 0.12$, $p = .999$.

Figure S56.

Simple Slopes Plot of the Interaction Effects between Both the Long (Left) and Short (Right) Discrediting Videos (vs. Control) and Intentions to Share the Video Within One's Social Network when Predicting Manipulation Discernment of the Discrediting Content.

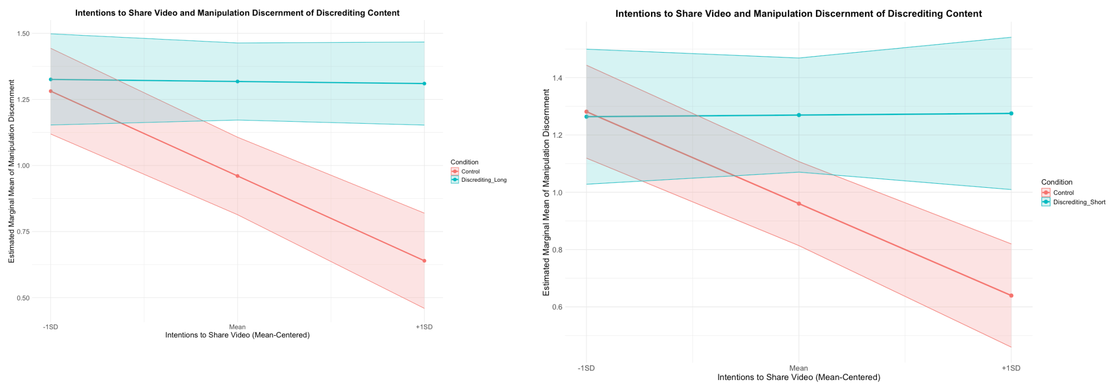

Technique recognition of manipulative discrediting content

There were significant interactions between both the long, *b* = -0.013, *SE* = 0.01, *t*(18,650) = -3.25, *p* = .001, and short discrediting videos (vs. control), *b* = -0.017, *SE* = 0.01, *t*(18,650) = -2.81, *p* = .005, and general manipulation discernment ability when predicting technique recognition of the manipulative discrediting content (see Figure S57). For the long discrediting video (vs. control), the positive significant effect was strongest at lower levels of general manipulation discernment ability, *b* = 0.10, *SE* = 0.01, *p* < .001, weaker at more moderate levels, *b* = 0.08, *SE* = 0.01, *p* < .001, and weakest at higher levels, *b* = 0.05, *SE* = 0.01, *p* < .001 (see Figure S57). For the short discrediting video (vs. control), the effect was only positive and significant at lower, *b* = 0.07, *SE* = 0.02, *p* < .001, and more moderate levels of general manipulation discernment ability, *b* = 0.04, *SE* = 0.01, *p* = .024, but not at higher levels, *b* = 0.01, *SE* = 0.02, *p* = .999 (see Figure S57).

Figure S57.

Simple Slopes Plot of the Interaction Effects between Both the Long (Left) and Short (Right) Discrediting Video (vs. Control) and General Manipulation Discernment when Predicting Technique Recognition of the Manipulative Discrediting Content.

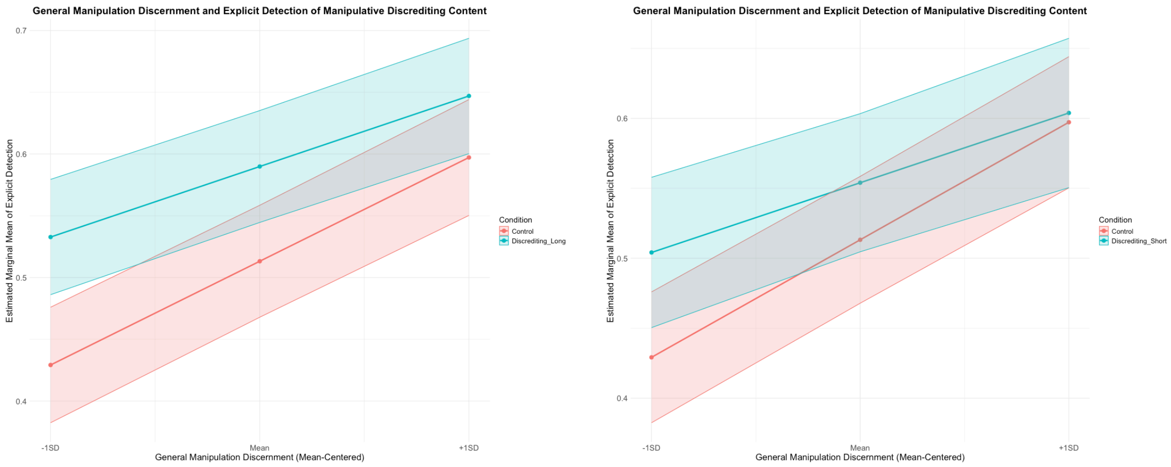

There was a significant interaction between the long discrediting video (vs. control) and digital literacy when predicting technique recognition of the manipulative discrediting content, $b = -0.03$, $SE = 0.01$, $t(18,420) = -2.84$, $p = .005$, such that the positive significant effect was strongest at lower levels of digital literacy, $b = 0.10$, $SE = 0.01$, $p < .001$, weaker at more moderate levels, $b = 0.08$, $SE = 0.01$, $p < .001$, and weakest at higher levels, $b = 0.05$, $SE = 0.01$, $p < .001$ (see Figure S58).

Figure S58.

Simple Slopes Plot of the Interaction Effect between the Long Discrediting Video (vs. Control) and Digital Literacy when Predicting Technique Recognition of the Manipulative Discrediting Content.

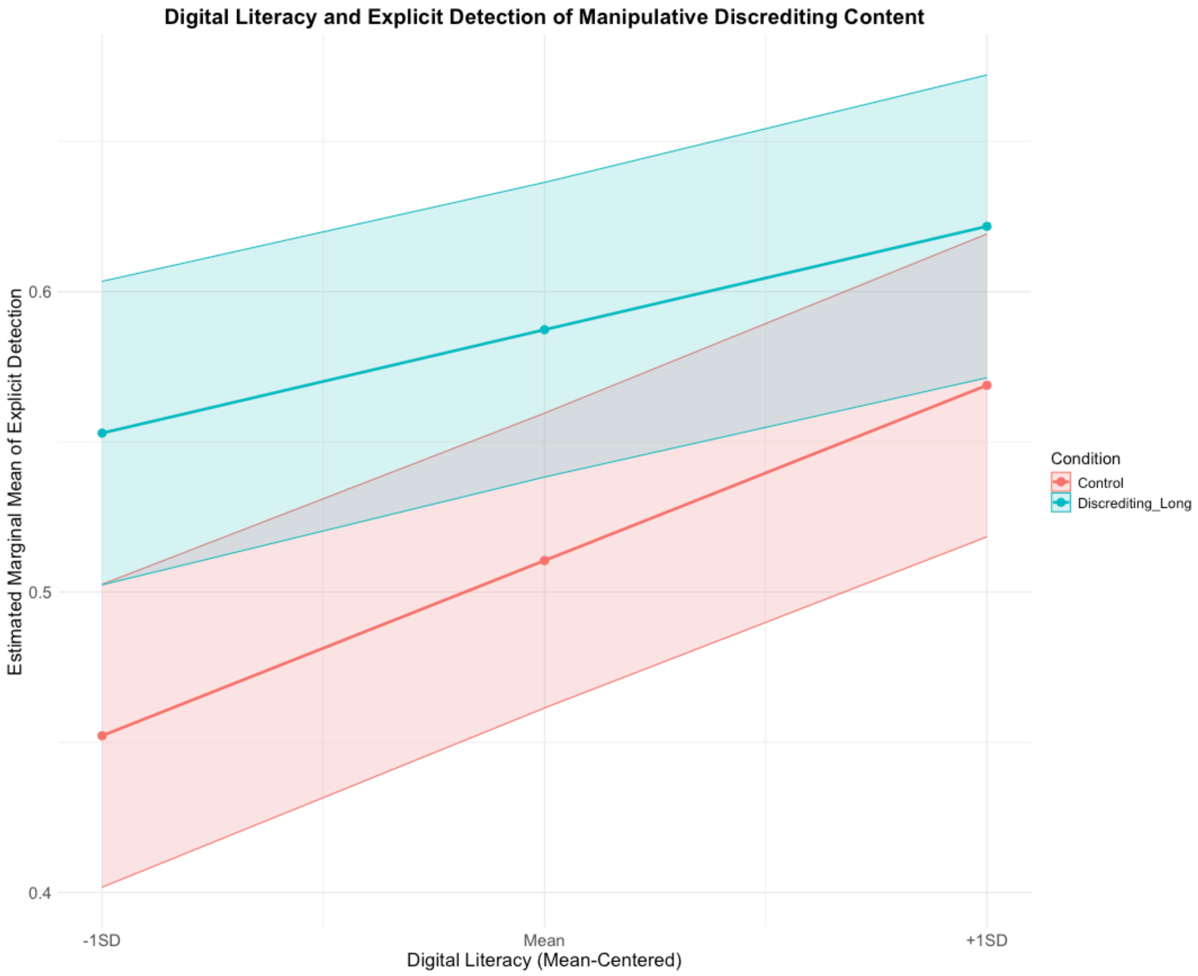

There were significant interactions between both the long, *b* = -0.02, *SE* = 0.01, *t*(18,340) = -2.75, *p* = .006, and short discrediting videos (vs. control), *b* = -0.04, *SE* = 0.01, *t*(18,350) = -2.84, *p* = .005, and political tolerance when predicting technique recognition of the manipulative discrediting content (see Figure S59). For the long discrediting video (vs. control), the positive significant effect was strongest at lower levels of political tolerance, *b* = 0.09, *SE* = 0.01, *p* < .001, weaker at more moderate levels, *b* = 0.07, *SE* = 0.01, *p* < .001, and weakest at higher levels, *b* = 0.05, *SE* = 0.01, *p* < .001 (see Figure S59). For the short discrediting video (vs. control), the effect was only positive and significant at lower levels of political literacy, *b* = 0.06, *SE* = 0.02, *p* = .008, but not at more moderate, *b* = 0.03, *SE* = 0.01, *p* = .277, or higher levels, *b* = -0.01, *SE* = 0.02, *p* = .999 (see Figure S59).

Figure S59.

Simple Slopes Plot of the Interaction Effects between Both the Long (Left) and Short (Right) Discrediting Video (vs. Control) and Political Tolerance when Predicting Technique Recognition of the Manipulative Discrediting Content.

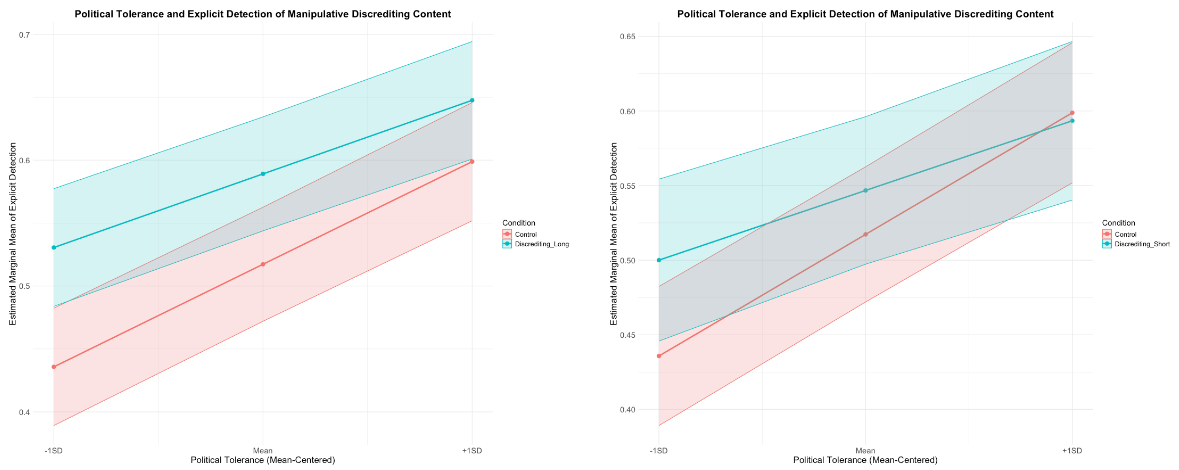

There was a significant interaction between the short discrediting video (vs. control) and longitude when predicting technique recognition of the manipulative discrediting content, $b = -0.01$, $SE = 0.01$, $t(18,540) = -2.05$, $p = .041$, such that the effect was only positive and significant among more Western European nations, $b = 0.08$, $SE = 0.02$, $p = .016$, but not among more central, $b = 0.03$, $SE = 0.01$, $p = .382$, or Eastern European nations, $b = -0.02$, $SE = 0.03$, $p = .999$ (see Figure S60).

Figure S60.

Simple Slopes Plot of the Interaction Effect between the Short Discrediting Video (vs. Control) and Longitude when Predicting Technique Recognition of the Manipulative Discrediting Content.

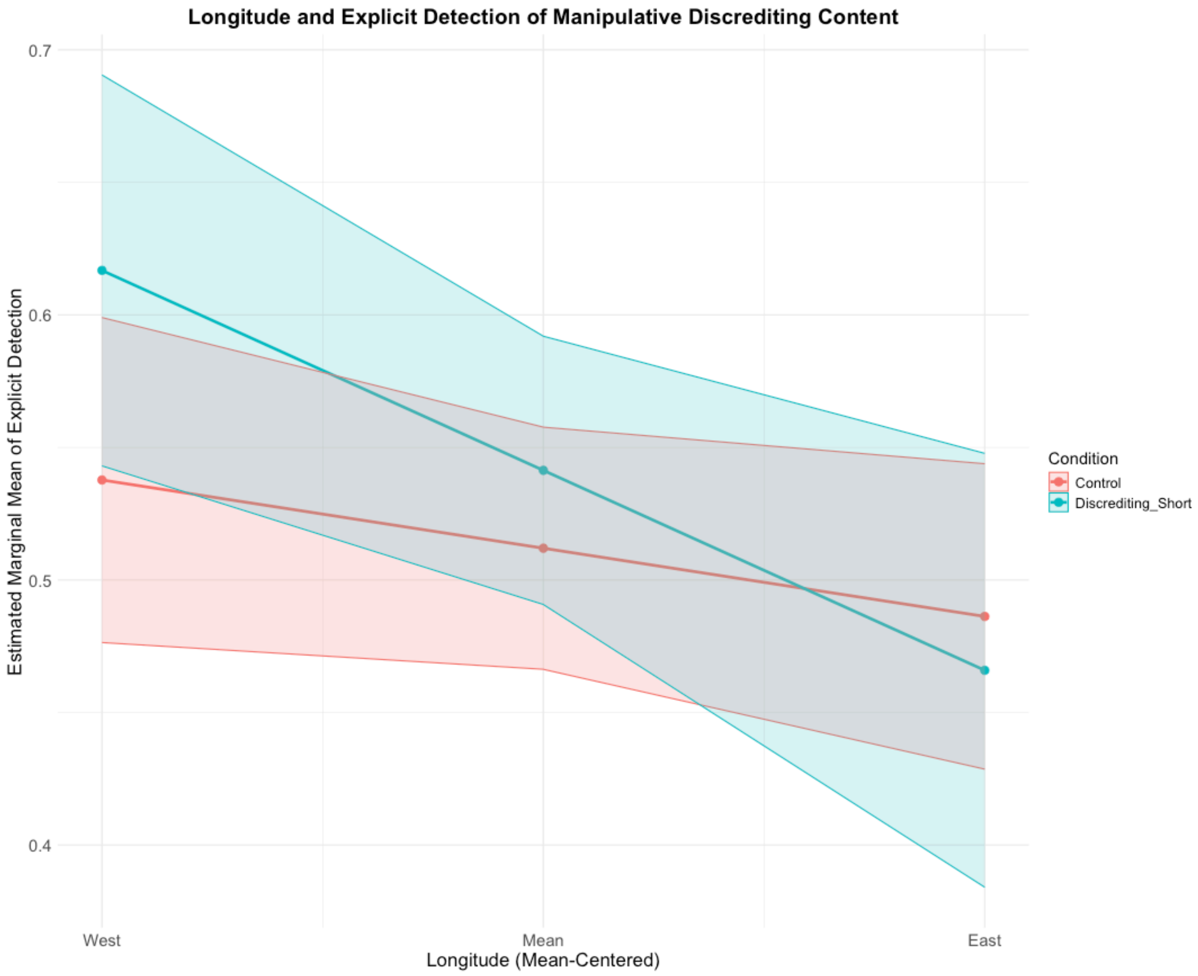

There were significant interactions between both the long, $b = 0.02$, $SE = 0.01$, $t(18{,}650) = 4.19$, $p < .001$, and short discrediting videos (vs. control), $b = 0.02$, $SE = 0.01$, $t(18{,}660) = 2.43$, $p = .015$, and intentions to share the video within one's social network when predicting technique recognition of the manipulative discrediting content (see Figure S61). For the long discrediting video (vs. control), the effect was positive and significant at higher, $b = 0.12$, $SE = 0.01$, $p < .001$, more moderate, $b = 0.08$, $SE = 0.01$, $p < .001$, and lower levels of intentions to share, $b = 0.05$, $SE = 0.01$, $p = .002$. For the short discrediting video (vs. control), the effect was positive and significant at higher, $b = 0.08$, $SE = 0.02$, $p = .002$, and more moderate intentions to share, $b = 0.05$, $SE = 0.01$, $p = .006$, but not when intentions to share were lower, $b = 0.01$, $SE = 0.02$, $p = .999$.

Figure S61.

Simple Slopes Plots of the Interaction Effects between the Long (Left) and Short (Right) Discrediting Videos (vs. Control) and Intentions to Share the Video within one's Social Network when Predicting Technique Recognition of the Manipulative Discrediting Content.

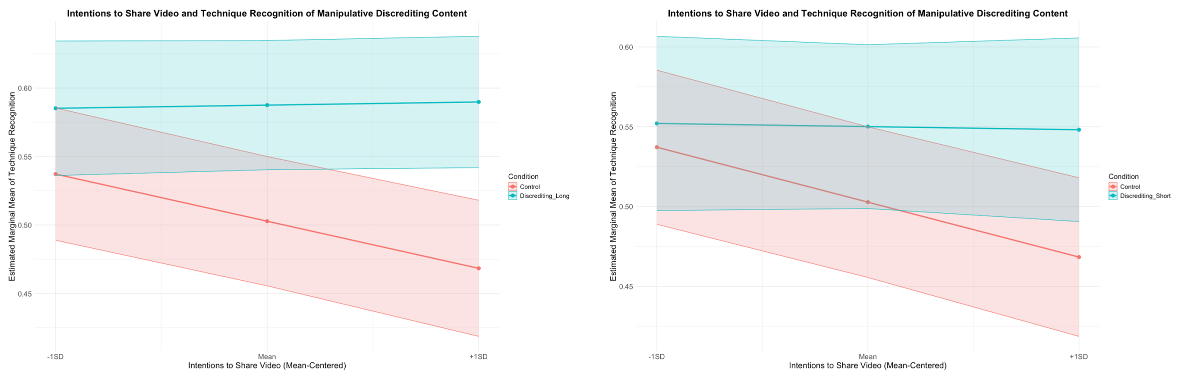

Technique recognition of non-manipulative discrediting content

There was a significant interaction between the long discrediting video (vs. control) and political tolerance when predicting technique recognition of the non-manipulative discrediting content, $b = 0.06$, $SE = 0.02$, $t(17{,}610) = 3.45$, $p < .001$, such that the effect was only negative and significant at lower, $b = -0.16$, $SE = 0.03$, $p < .001$, and more moderate levels of political tolerance, $b = -0.10$, $SE = 0.02$, $p < .001$, but not at higher levels, $b = -0.04$, $SE = 0.03$, $p = .999$ (see Figure S62).

Figure S62.

Simple Slopes Plot of the Interaction Effect between the Long Discrediting Video (vs. Control) and Political Tolerance when Predicting Technique Recognition of the Non-Manipulative Discrediting Content.

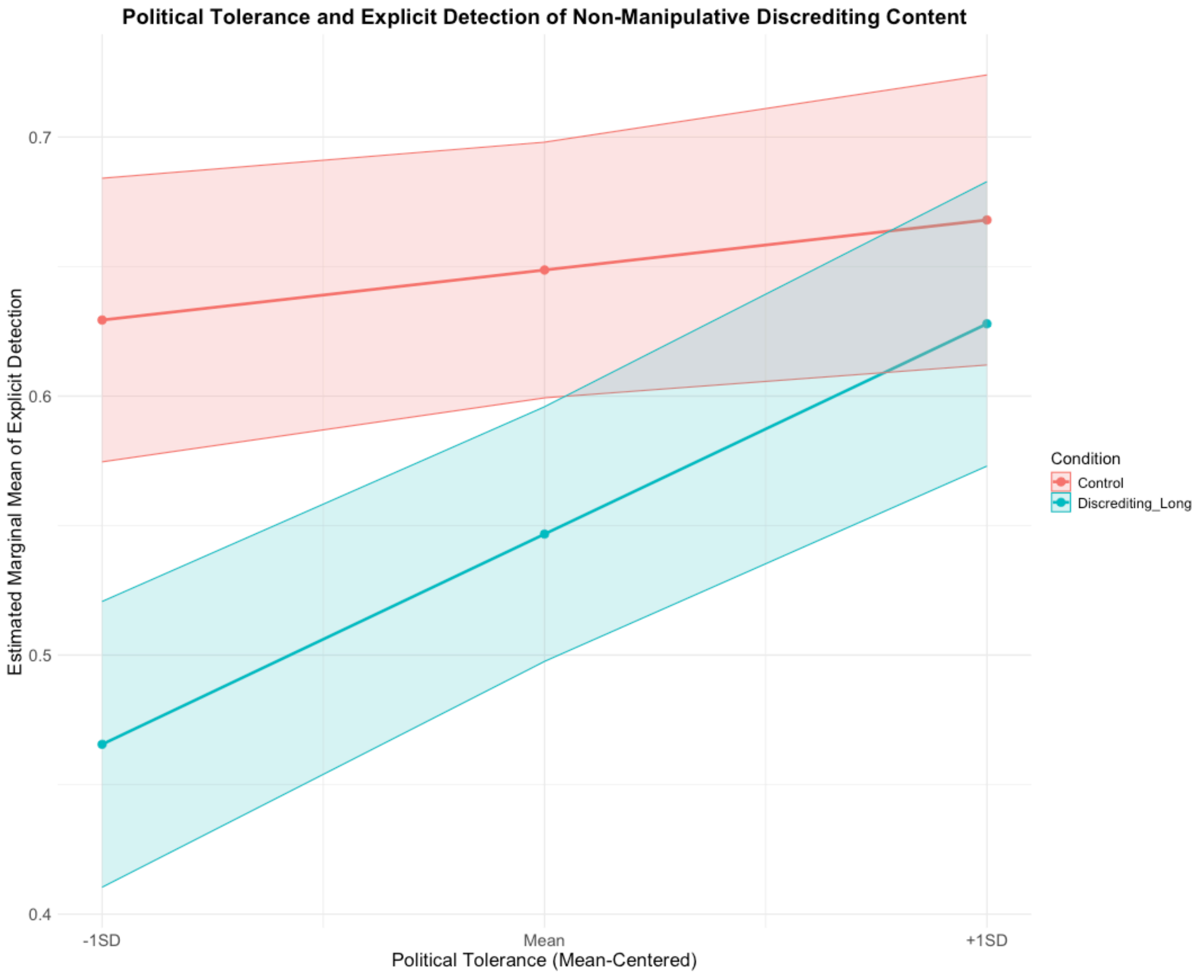

Technique discernment of discrediting content

There was a significant interaction between the long discrediting video (vs. control) and political tolerance when predicting technique discernment of the discrediting content, b = 0.02, SE = 0.01, t(19,060) = 2.54, p = .011, such that the effect was only positive and significant at higher levels of political tolerance, b = 0.03, SE = 0.01, p = .026, but not at more moderate, b = 0.01, SE = 0.01, p = .799, or lower levels, b = -0.01, SE = 0.01, p = .999 (see Figure S63).

Figure S63.

Simple Slopes Plot of the Interaction Effect between the Long Discrediting Video (vs. Control) and Political Tolerance when Predicting Technique Discernment of the Discrediting Content.

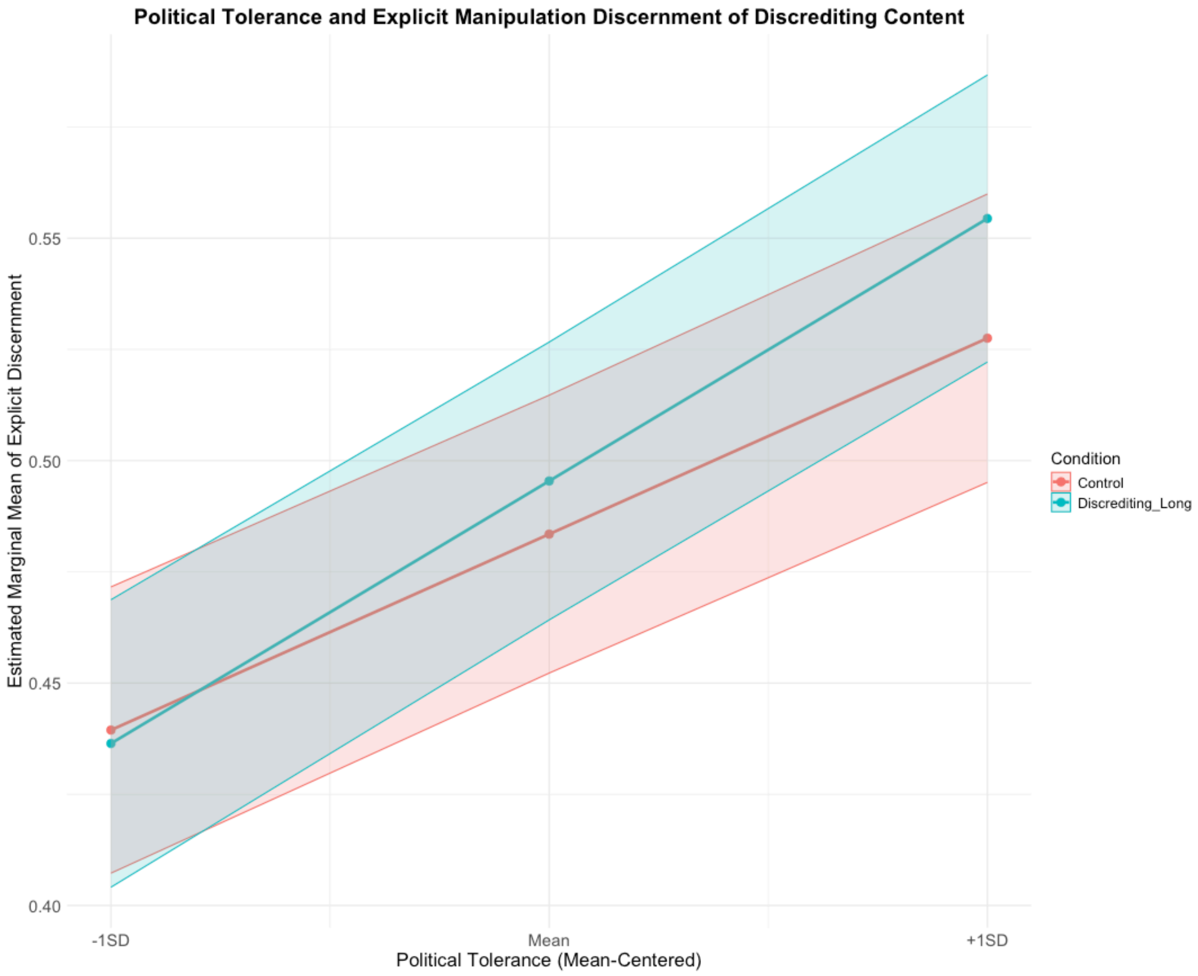

There was a significant interaction between the long discrediting video (vs. control) and educational attainment when predicting technique discernment of the discrediting content, $b = 0.01$, $SE = 0.01$, $t(19{,}410) = 2.45$, $p = .014$, such that the effect was only positive and significant at higher levels of educational attainment, $b = 0.03$, $SE = 0.01$, $p = .010$, but not at more moderate, $b = 0.01$, $SE = 0.01$, $p = .339$, or lower levels, $b = -0.01$, $SE = 0.01$, $p = .999$ (see Figure S64).

Figure S64.

Simple Slopes Plot of the Interaction Effect between the Long Discrediting Video (vs. Control) and Educational Attainment when Predicting Technique Discernment of the Discrediting Content.

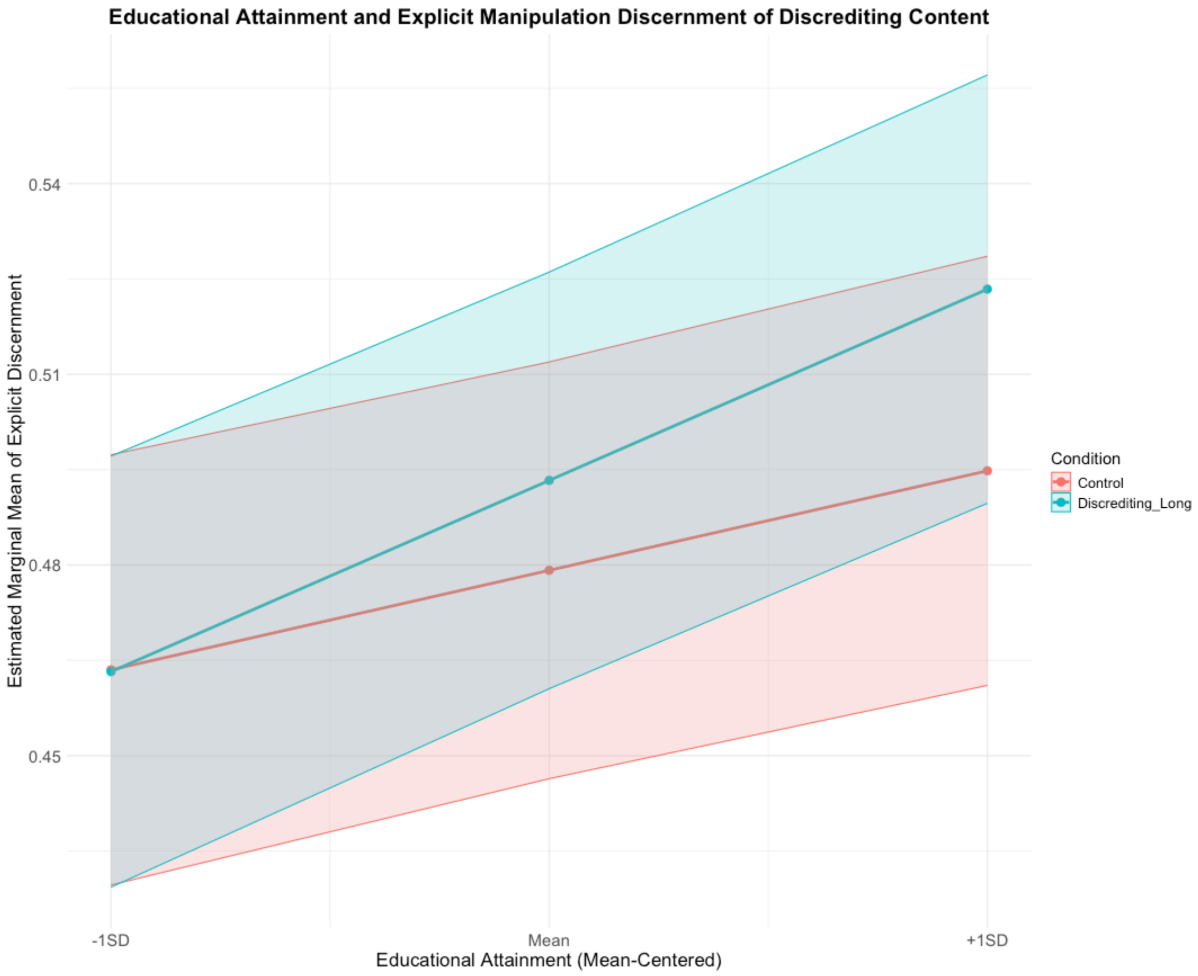

Willingness to share manipulative discrediting content

There was a significant interaction between the short discrediting video (vs. control) and digital literacy when predicting willingness to share the manipulative discrediting content, b = -0.17, SE = 0.07, t(18,040) = -2.55, p = .011, such that the effect was only positive and significant at lower levels of digital literacy, b = 0.26, SE = 0.08, p = .021, but not at more moderate, b = 0.12, SE = 0.06, p = .777, or lower levels, b = -0.03, SE = 0.08, p = .999 (see Figure S65).

Figure S65.

Simple Slopes Plot of the Interaction Effect between the Short Discrediting Video (vs. Control) and Digital Literacy when Predicting Willingness to Share the Manipulative Discrediting Content.

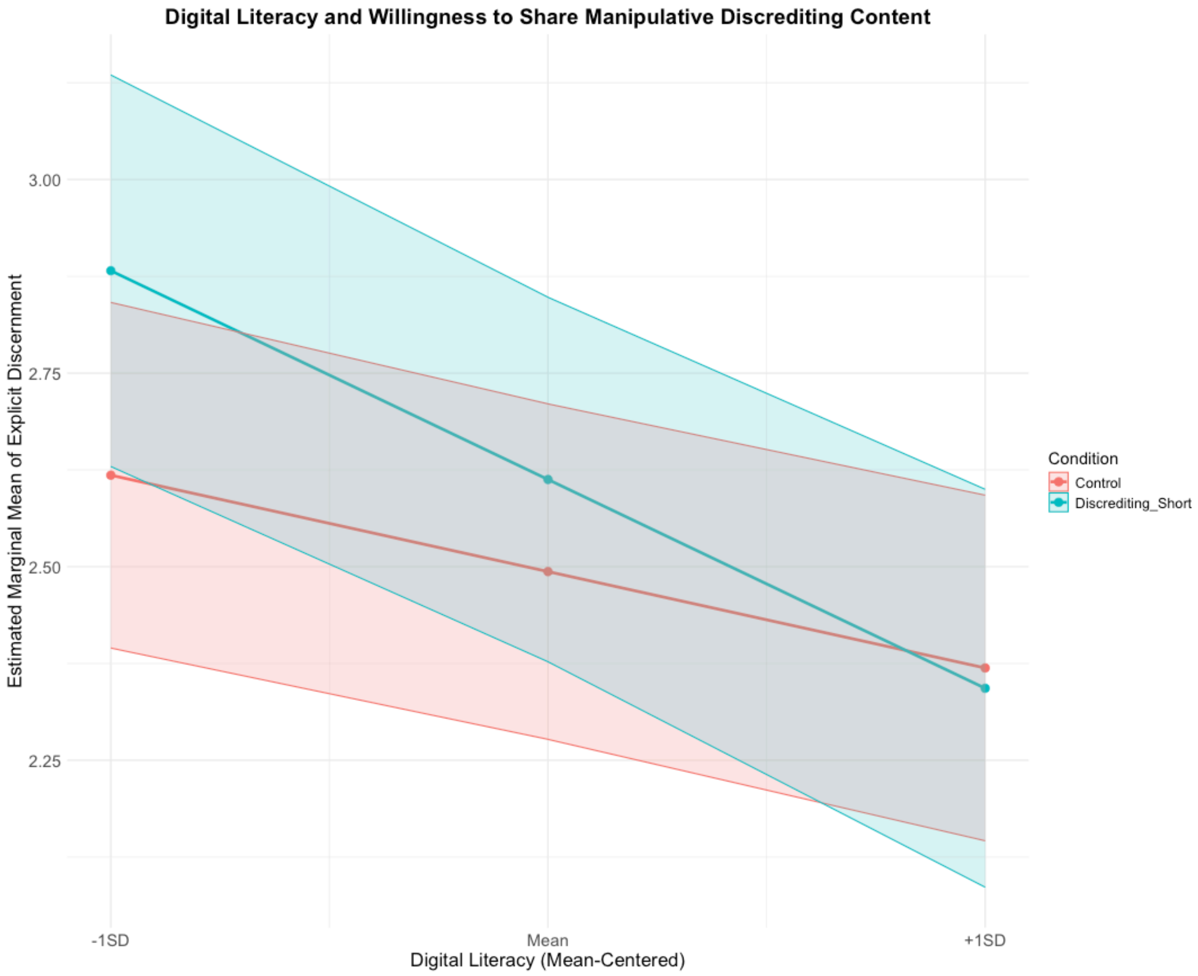

There was a significant interaction between the long discrediting video (vs. control) and intentions to share the video within one's social network when predicting willingness to share the manipulative discrediting content, $b = -0.14$, $SE = 0.02$, $t(18{,}650) = -7.43$, $p < .001$ (see Figure S66). The effect of the long discrediting video (vs. control) on willingness to share the manipulative discrediting content was negative and significant at higher, $b = -0.48$, $SE = 0.06$, $p < .001$, and at more moderate intentions to share, $b = -0.19$, $SE = 0.04$, $p < .001$, but was not significant when intentions to share were lower, $b = -0.10$, $SE = 0.05$, $p = .639$.

Figure S66.

Simple Slopes Plot of the Interaction Effect between the Long Discrediting Video (vs. Control) and Intentions to Share the Video Within One's Social Network when Predicting Willingness to Share the Manipulative Discrediting Content.

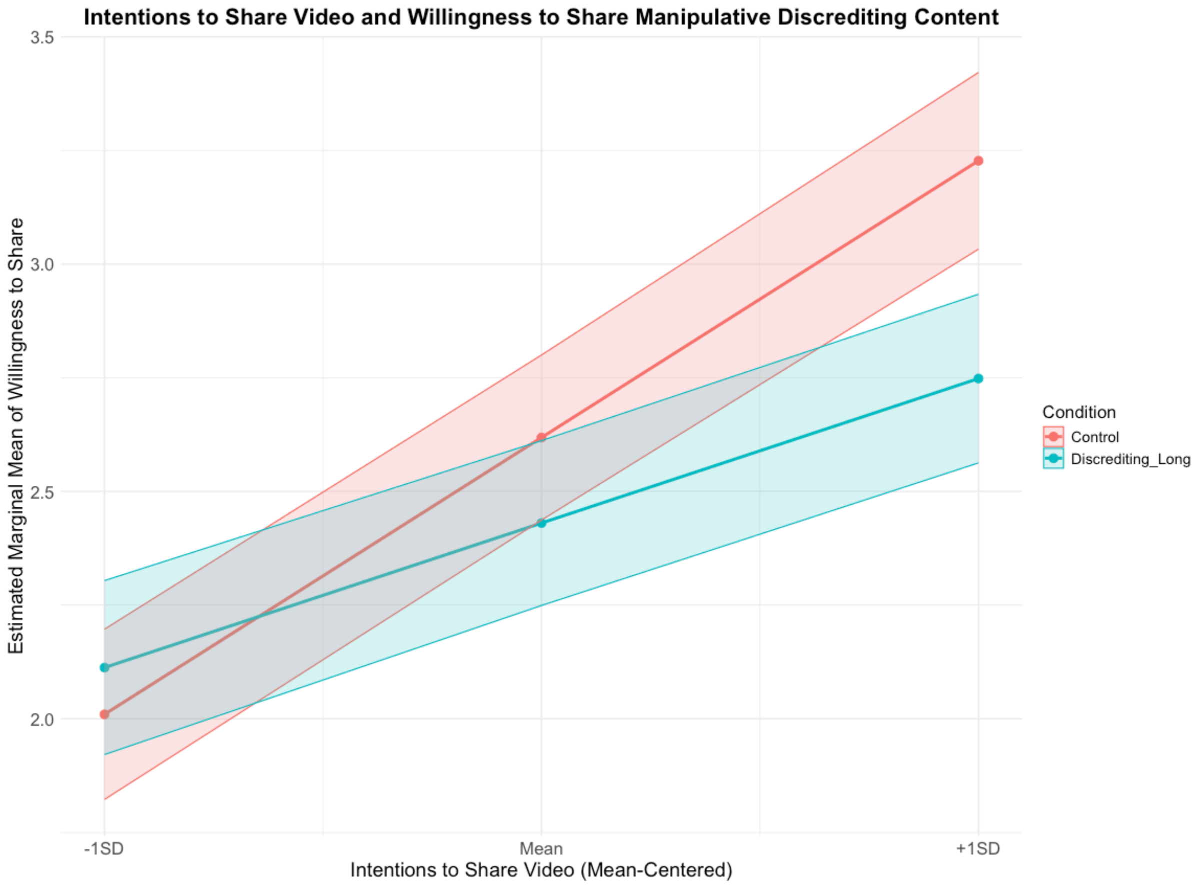

Willingness to share non-manipulative discrediting content

There were significant interactions between both the long, $b = 0.05$, $SE = 0.02$, $t(18{,}630) = 2.64$, $p = .008$, and short discrediting videos (vs. control), $b = 0.06$, $SE = 0.03$, $t(18{,}630) = 2.10$, $p = .036$, and general manipulation discernment ability when predicting willingness to share the non-manipulative discrediting content, such that the effects were only significant at higher levels of general manipulation discernment ability for the long, $b = 0.36$, $SE = 0.05$, $p < .001$, and short discrediting videos (vs. control), $b = 0.35$, $SE = 0.08$, $p < .001$, and more moderate levels of general manipulation discernment ability for the long, $b = 0.26$, $SE = 0.04$, $p < .001$, and short discrediting videos (vs. control), $b = 0.23$, $SE = 0.06$, $p = .002$, but not lower levels of general manipulation discernment ability for the long, $b = 0.15$, $SE = 0.05$, $p = .084$, and short discrediting videos (vs. control), $b = 0.12$, $SE = 0.08$, $p = .999$ (see Figure S67).

Figure S67.

Simple Slopes Plot of the Interaction Effects between the Long (Left) and Short (Right) Discrediting Videos (vs. Control) and General Manipulation Discernment Ability when Predicting Willingness to Share the non-Manipulative Discrediting Content.

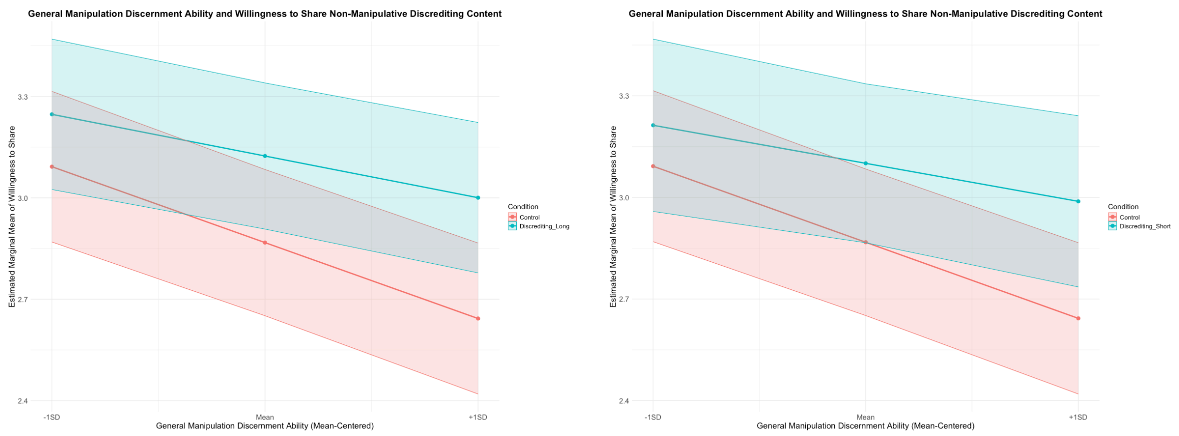

There were significant interactions between both the long, $b = 0.14$, $SE = 0.04$, $t(18{,}320) = 3.50$, $p < .001$, and short discrediting videos (vs. control), $b = 0.11$, $SE = 0.06$, $t(18{,}320) = 1.96$, $p = .050$, and political tolerance when predicting willingness to share the non-manipulative discrediting content, such that the effects were only significant at higher levels of political tolerance for the long, $b = 0.40$, $SE = 0.05$, $p < .001$, and short discrediting videos (vs. control), $b = 0.38$, $SE = 0.08$, $p < .001$, and more moderate levels of political tolerance for the long, $b = 0.27$, $SE = 0.04$, $p < .001$, and short discrediting videos (vs. control), $b = 0.27$, $SE = 0.06$, $p < .001$, but not lower levels of political tolerance for the long, $b = 0.13$, $SE = 0.05$, $p = .282$, and short discrediting videos (vs. control), $b = 0.16$, $SE = 0.08$, $p = .999$ (see Figure S68).

Figure S68.

Simple Slopes Plot of the Interaction Effects between the Long (Left) and Short (Right) Discrediting Videos (vs. Control) and Political Tolerance when Predicting Willingness to Share the non-Manipulative Discrediting Content.

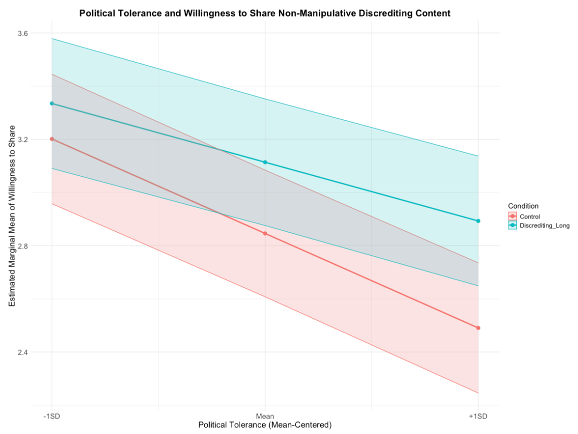

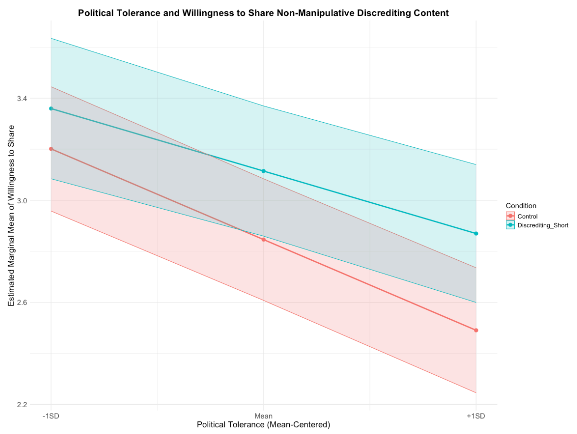

There was a significant interaction between the short discrediting video (vs. control) and political ideology when predicting willingness to share the non-manipulative discrediting content, b = -0.08, SE = 0.04, t(18,630) = -2.04, p = .041, such that the effect was only positive and significant among more politically left-wing, b = 0.35, SE = 0.08, p < .001, and more politically centrist participants, b = 0.24, SE = 0.06, p = .001, but not among more politically right-wing participants, b = 0.13, SE = 0.08, p = .999 (see Figure S69).

Figure S69.

Simple Slopes Plot of the Interaction Effect between the Short Discrediting Video (vs. Control) and Political Ideology when Predicting Willingness to Share the Non-Manipulative Discrediting Content.

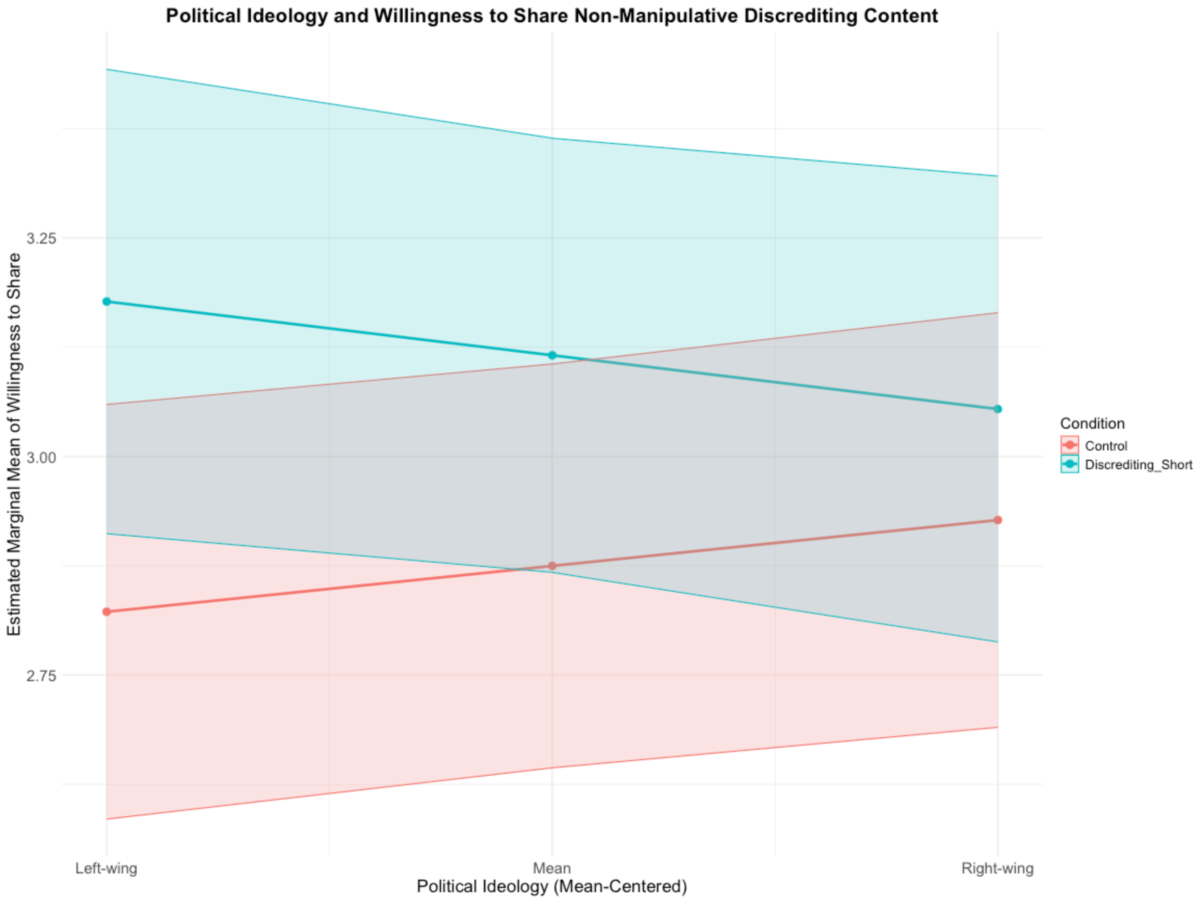

There was a significant interaction between the long discrediting video (vs. control) and the industrialization indices of nations when predicting willingness to share the non-manipulative discrediting content, $b = 0.01$, $SE = 0.01$, $t(18{,}630) = 2.23$, $p = .026$, such that the positive significant effect was strongest at higher levels of industrialization indices, $b = 0.33$, $SE = 0.05$, $p < .001$, weaker at more moderate levels, $b = 0.25$, $SE = 0.04$, $p < .001$, and weakest and lower levels, $b = 0.17$, $SE = 0.05$, $p = .037$ (see Figure S70).

Figure S70.

Simple Slopes Plot of the Interaction Effect between the Long Discrediting Video (vs. Control) and Industrialization Index when Predicting Willingness to Share the Non-Manipulative Discrediting Content.

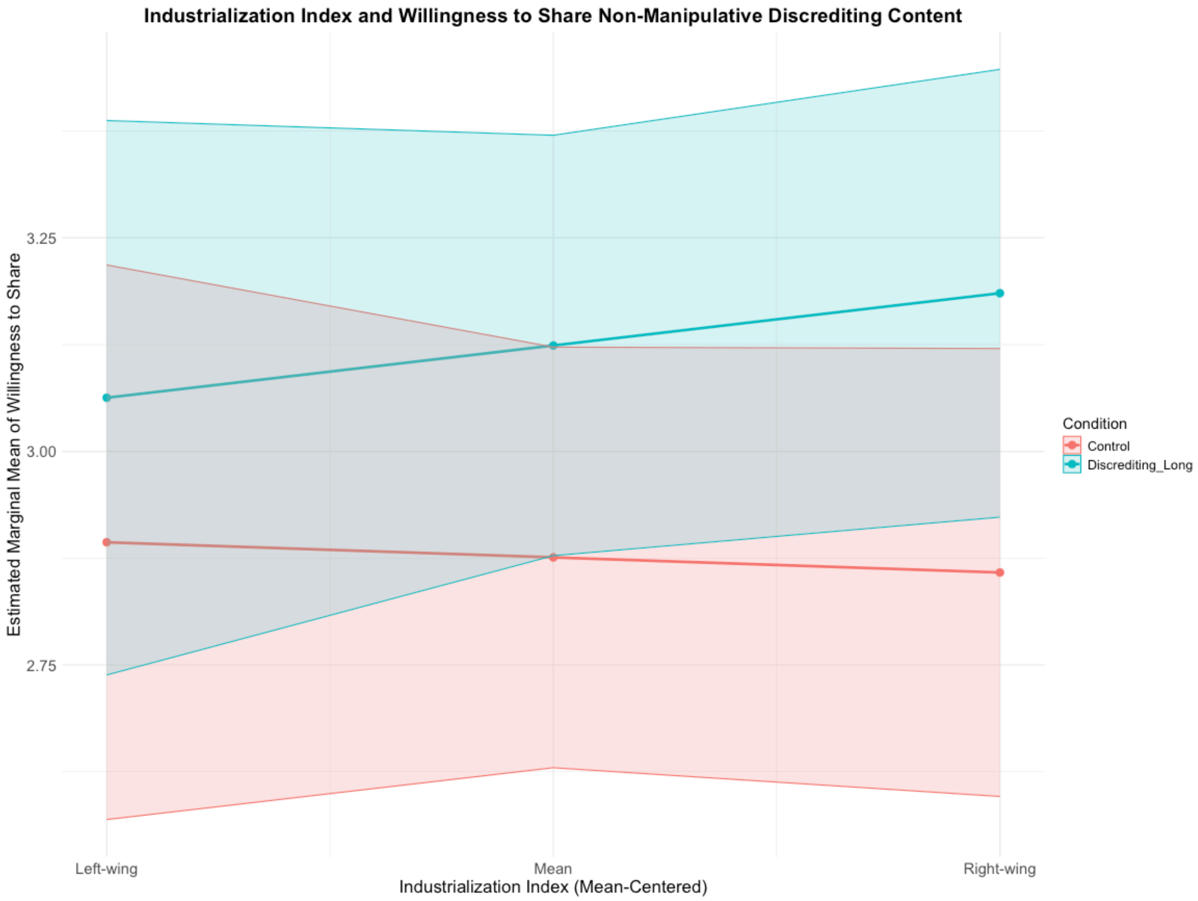

There was a significant interaction between the long discrediting video (vs. control) and gender when predicting willingness to share the non-manipulative discrediting content, *b* = -0.16, *SE* = 0.08, *t*(18,580) = -2.07, *p* = .038, such that the positive significant effect was strongest among women, *b* = 0.33, *SE* = 0.05, *p* < .001, and weaker among men, *b* = 0.17, *SE* = 0.05, *p* = .035 (see Figure S71).

Figure S71.

Simple Slopes Plot of the Interaction Effect between the Long Discrediting Video (vs. Control) and Gender when Predicting Willingness to Share the Non-Manipulative Discrediting Content.

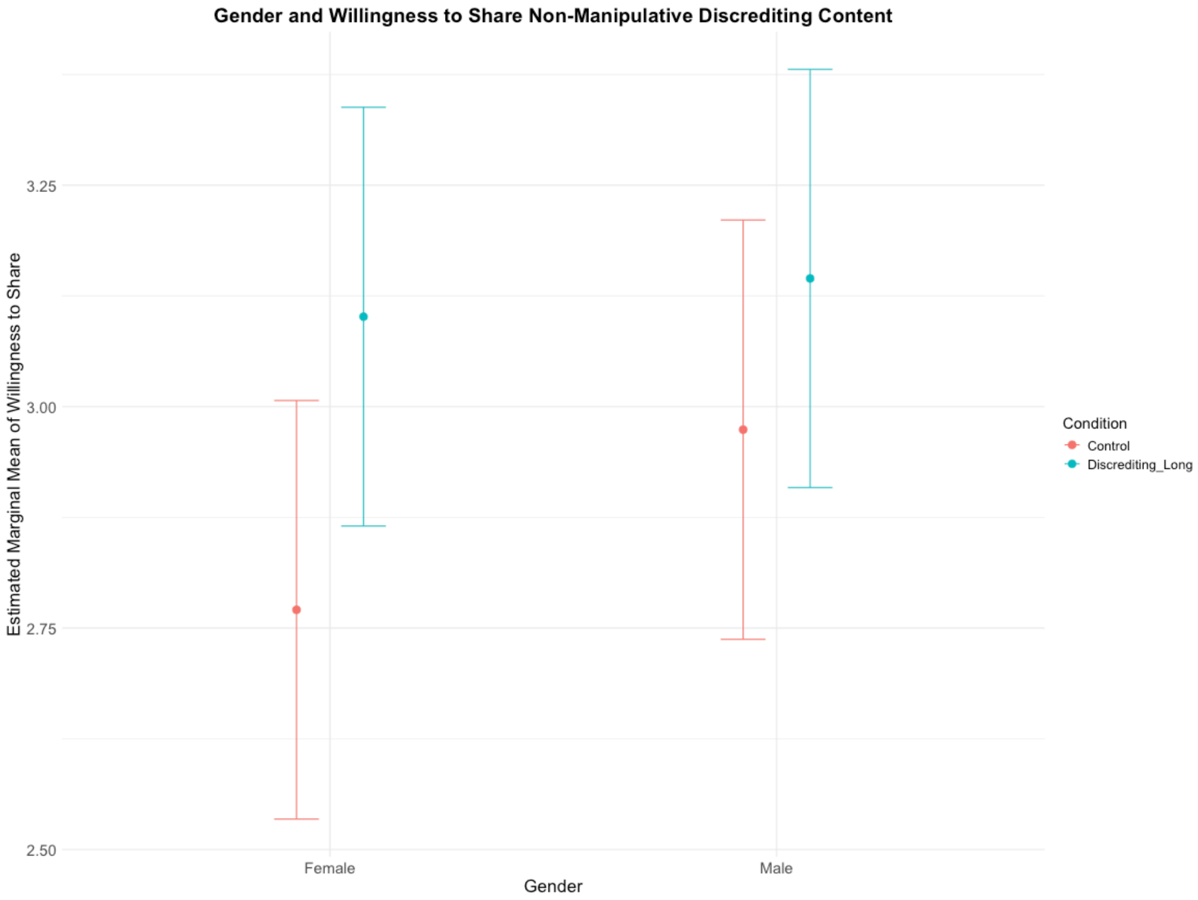

Sharing decisions for the discrediting content

There was a significant interaction between the long discrediting video (vs. control) and general manipulation discernment ability when predicting sharing decisions for the discrediting content, $b = 0.04$, $SE = 0.02$, $t(17{,}490) = 2.42$, $p = .015$, such that the positive significant effect was strongest at higher levels of general manipulation discernment ability, $b = 0.32$, $SE = 0.04$, $p < .001$, weaker at more moderate levels, $b = 0.24$, $SE = 0.03$, $p < .001$, and weakest at lower levels, $b = 0.17$, $SE = 0.04$, $p = .004$ (see Figure S72).

Figure S72.

Simple Slopes Plot of the Interaction Effect between the Long Discrediting Video (vs. Control) and General Manipulation Discernment Ability when Predicting Sharing Decisions for the Discrediting Content.

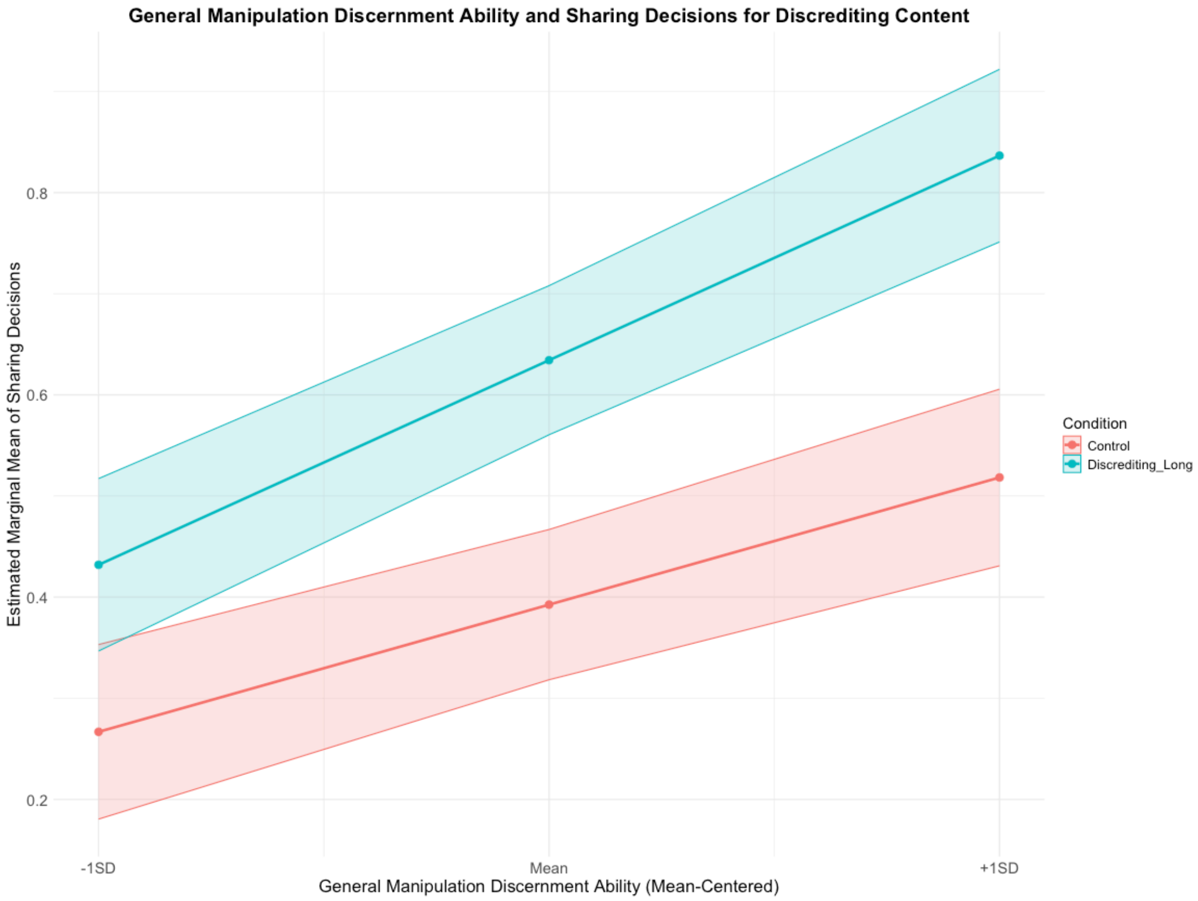

There were significant interactions between both the long, $b = -0.06$, $SE = 0.02$, $t(17{,}490) = -2.73$, $p = .006$, and short discrediting videos (vs. control), $b = -0.08$, $SE = 0.03$, $t(17{,}490) = -2.37$, $p = .018$, and political ideology when predicting sharing decisions for the discrediting content (see Figure S73). For the long discrediting video (vs. control), the positive significant effect was strongest among more politically left-wing participants, $b = 0.33$, $SE = 0.04$, $p < .001$, weaker among more politically centrist participants, $b = 0.24$, $SE = 0.03$, $p < .001$, and weakest among more politically right-wing participants, $b = 0.16$, $SE = 0.04$, $p = .009$ (see Figure S73). For the short discrediting video (vs. control), the effect was only positive and significant among more politically left-wing participants, $b = 0.24$, $SE = 0.07$, $p = .004$, but not among more politically centrist, $b = 0.14$, $SE = 0.05$, $p = .085$, or more politically right-wing participants, $b = 0.03$, $SE = 0.07$, $p = .999$ (see Figure S73).

Figure S73.

Simple Slopes Plot of the Interaction Effects between the Long (Left) and Short (Right) Discrediting Video (vs. Control) and Political Ideology when Predicting Sharing Decisions for the Discrediting Content.

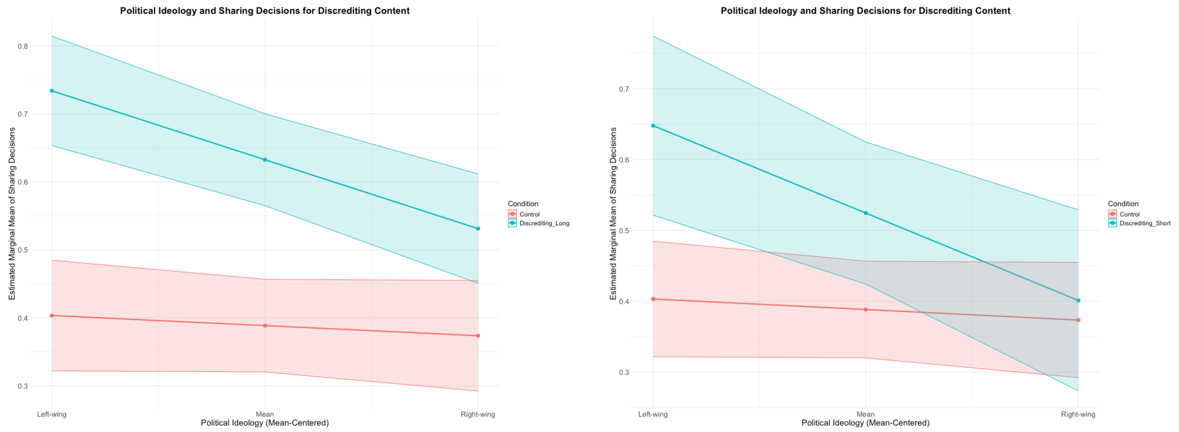

There was a significant interaction between the long discrediting video (vs. control) and educational attainment when predicting sharing decisions for the discrediting content, $t(17{,}490) = 1.96$, $p = .050$, such that the positive significant effect was strongest at higher levels of educational attainment, $b = 0.30$, $SE = 0.04$, $p < .001$, weaker at more moderate levels, $b = 0.24$, $SE = 0.03$, $p < .001$, and weakest at lower levels, $b = 0.18$, $SE = 0.05$, $p = .002$ (see Figure S74).

Figure S74.

Simple Slopes Plot of the Interaction Effect between the Long Discrediting Video (vs. Control) and Educational Attainment when Predicting Sharing Decisions for the Discrediting Content.

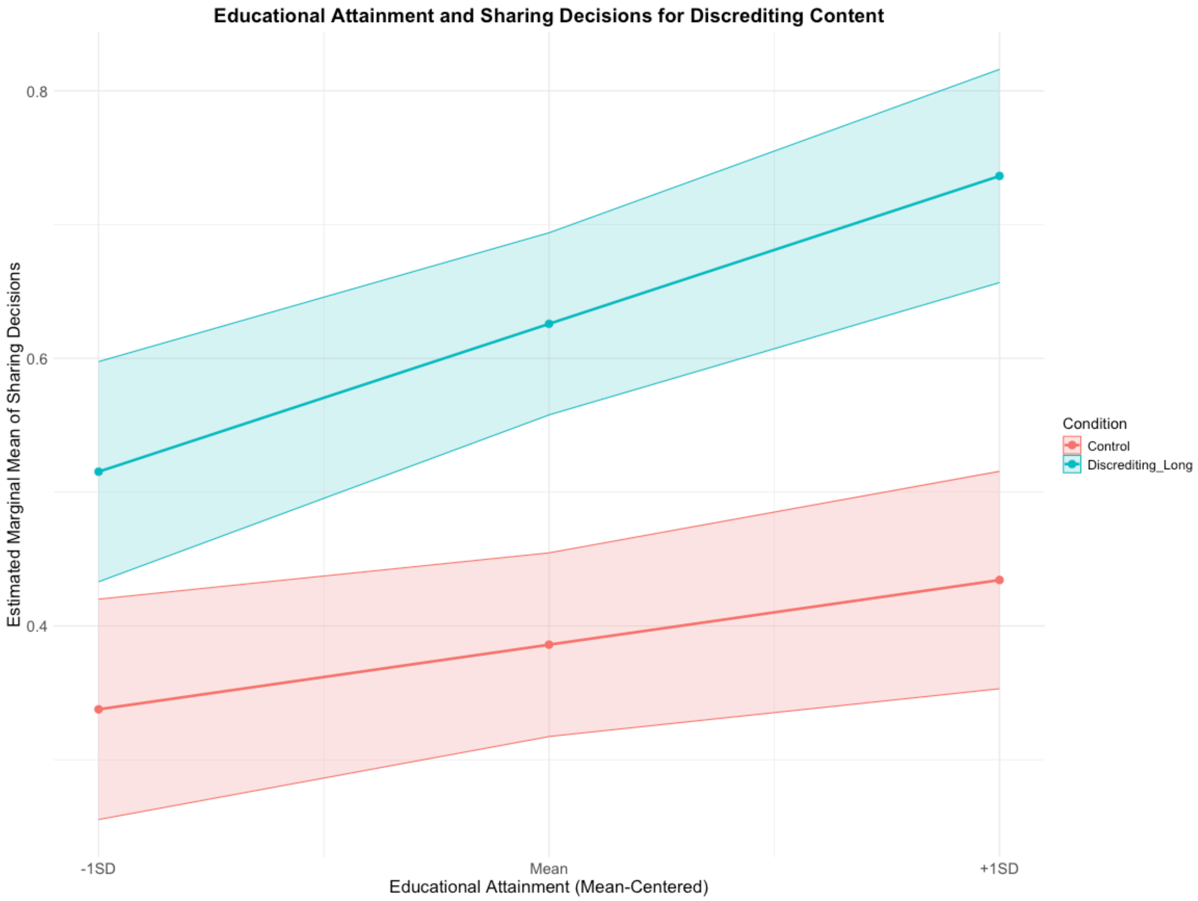

There was a significant interaction between the long discrediting video (vs. control) and gender when predicting sharing decisions for the discrediting content, $t(17{,}430) = 2.70$, $p = .007$, such that the positive significant effect was strongest among women, $b = 0.33$, $SE = 0.04$, $p < .001$, and weaker among men, $b = 0.16$, $SE = 0.05$, $p = .010$ (see Figure S75).

Figure S75.

Simple Slopes Plot of the Interaction Effect between the Long Discrediting Video (vs. Control) and Gender when Predicting Sharing Decisions for the Discrediting Content.

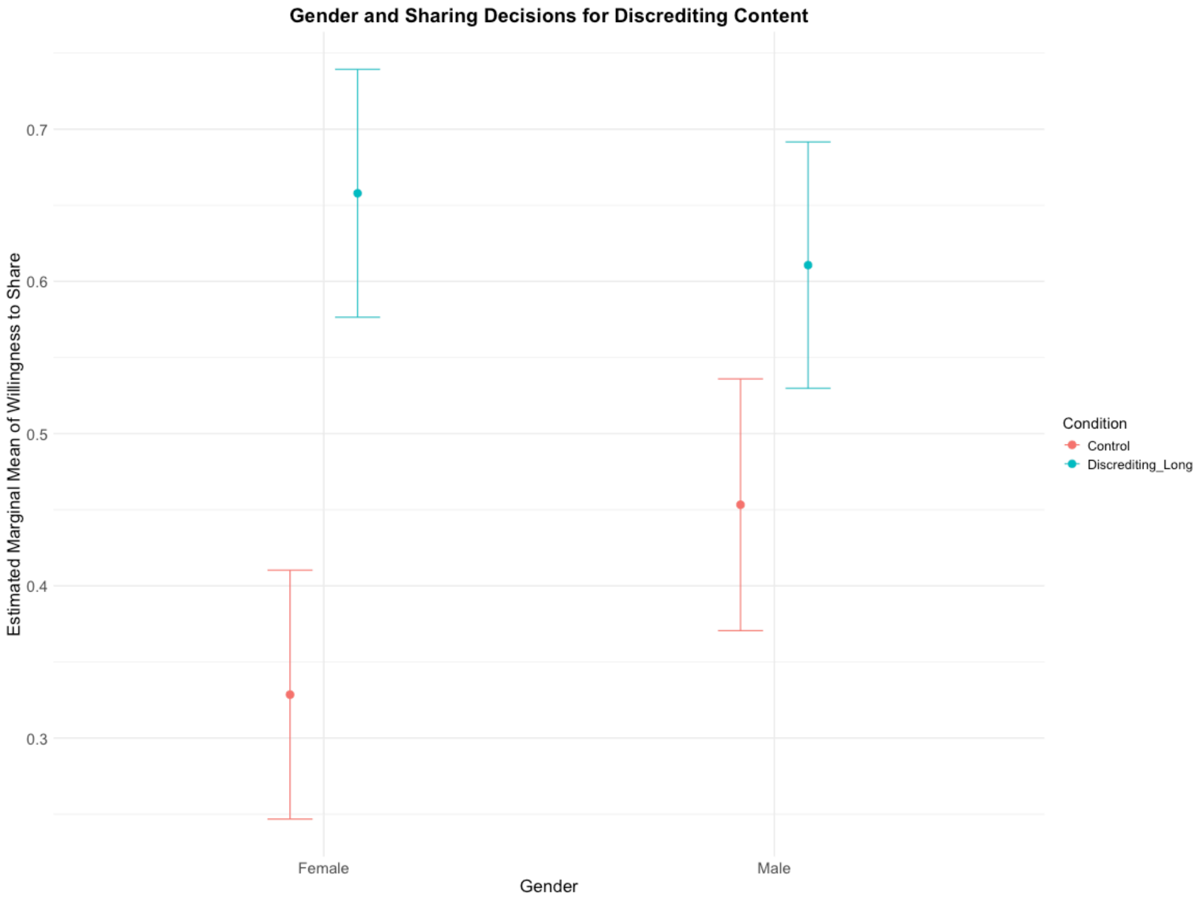

There was a significant interaction between the long discrediting video (vs. control) and intentions to share the video within one's social network when predicting sharing decisions for the discrediting content, $b = 0.04$, $SE = 0.02$, $t(17{,}850) = 2.83$, $p = .005$ (see Figure S76). The effect of the long discrediting video (vs. control) on sharing decisions for the discrediting content was positive and significant at higher, $b = 0.33$, $SE = 0.05$, $p < .001$, and at more moderate levels of intentions to share, $b = 0.16$, $SE = 0.03$, $p < .001$, but was not significant when intentions to share were lower, $b = -0.08$, $SE = 0.04$, $p = .547$.

Figure S76.

Simple Slopes Plot of the Interaction Effect between the Long Discrediting Video (vs. Control) and Intentions to Share the Video Within One's Social Network when Predicting Sharing Decisions for the Discrediting Content.

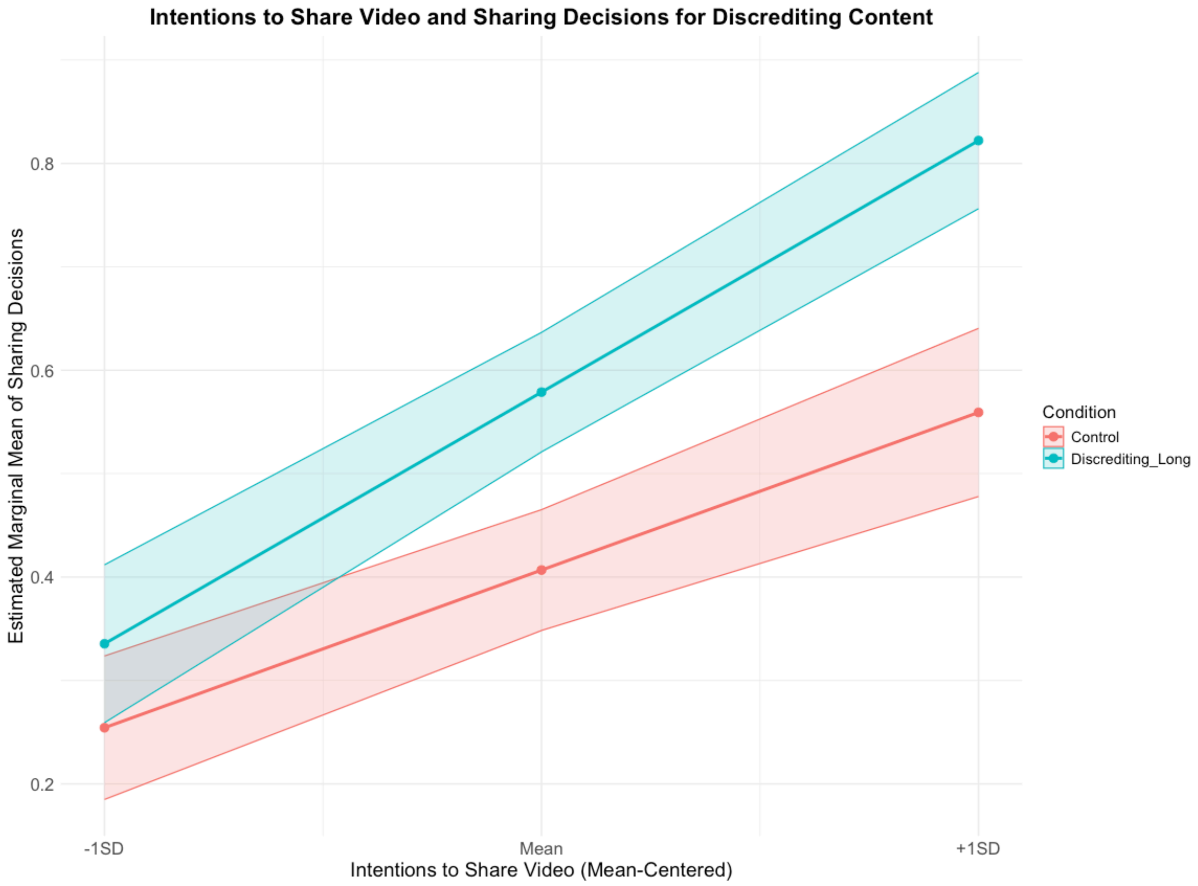

Confidence in detecting manipulation

Scapegoating videos

There was a significant interaction between the long scapegoating video (vs. control) and GDP per capita when predicting confidence in detecting manipulation, b = -0.01, SE = 0.01, t(16,120) = 2.17, p = .030, such that the effect was only positive and significant among nations with lower, b = 0.20, SE = 0.04, p < .001, and more moderate GDPs per capita, b = 0.14, SE = 0.03, p = .001, but not among nations with higher GDPs per capita, b = 0.07, SE = 0.05, p = .999 (see Figure S77).

Figure S77.

Simple Slopes Plot of the Interaction Effect between the Long Scapegoating Video (vs. Control) and GDP per capita when Predicting Confidence in Detecting Manipulation.

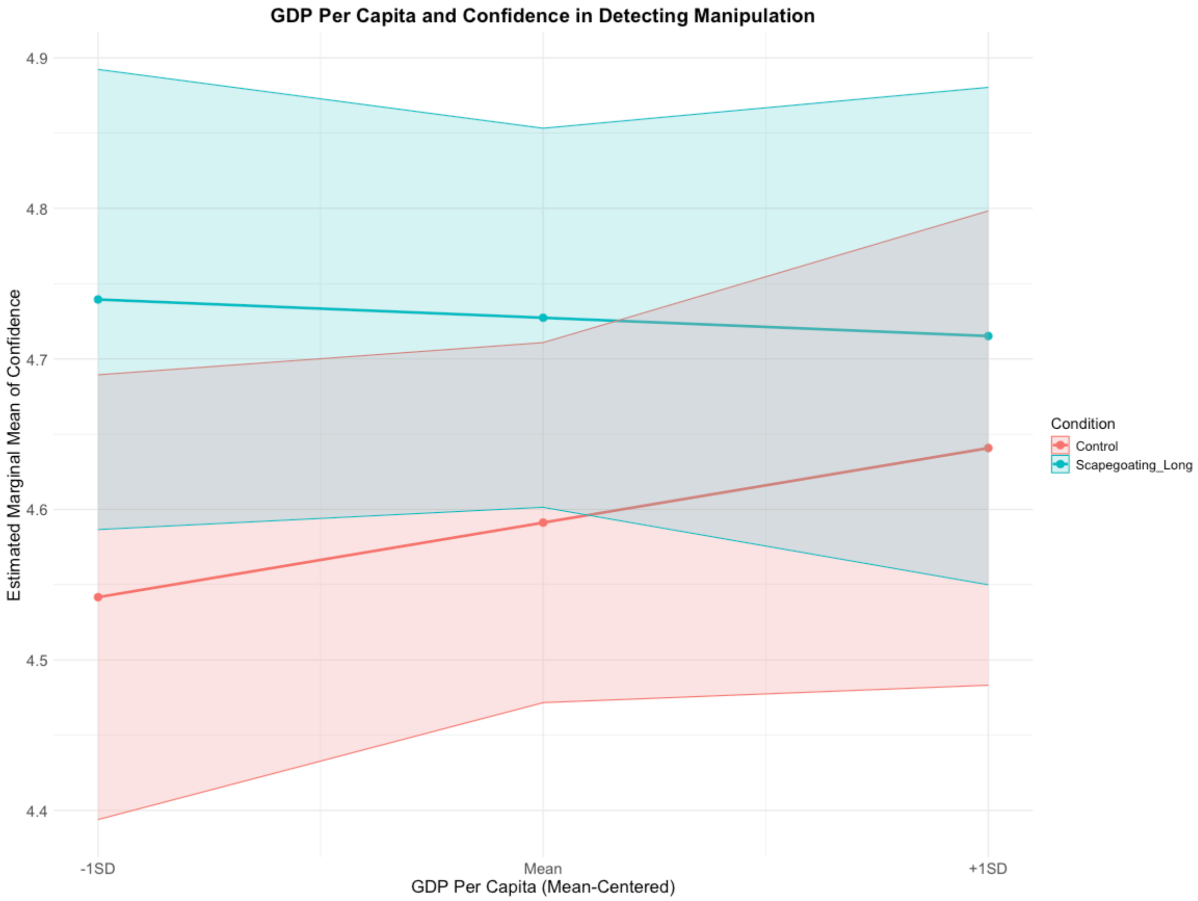

There was a significant interaction between the long scapegoating video (vs. control) and voting behaviour when predicting confidence in detecting manipulation, b = 0.18, SE = 0.09, t(16,120) = 2.14, p = .033, such that the positive significant effect was stronger among those who did not vote in the June 2024 EU elections, b = 0.30, SE = 0.08, p = .004, and weaker among those who did vote, b = 0.11, SE = 0.04, p = .030 (see Figure S78).

Figure S78.

Simple Slopes Plot of the Interaction Effect between the Long Scapegoating Video (vs. Control) and Voting Behaviour when Predicting Confidence in Detecting Manipulation.

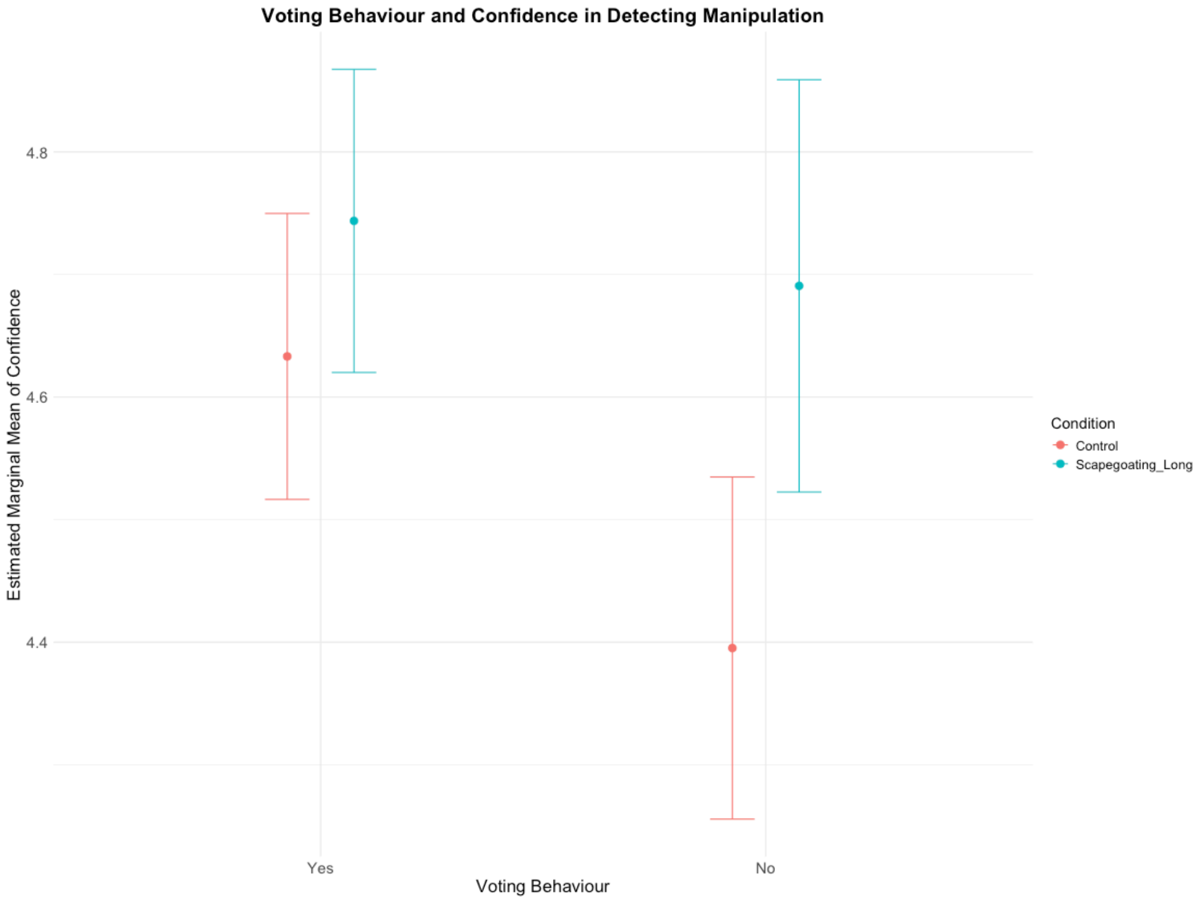

Decontextualization videos

There was a significant interaction between the short decontextualization video (vs. control) and general manipulation discernment ability when predicting confidence in detecting manipulation, *b* = -0.04, *SE* = 0.02, *t*(16,130) = 2.08, *p* = .038, such that the effect was only negative and significant at higher levels of general manipulation discernment ability, *b* = -0.17, *SE* = 0.06, *p* = .028, but not at more moderate, *b* = -0.09, *SE* = 0.04, *p* = .385, or lower levels, *b* = -0.01, *SE* = 0.06, *p* = .999 (see Figure S79).

Figure S79.

Simple Slopes Plot of the Interaction Effect between the Short Decontextualization Video (vs. Control) and General Manipulation Discernment Ability when Predicting Confidence in Detecting Manipulation.

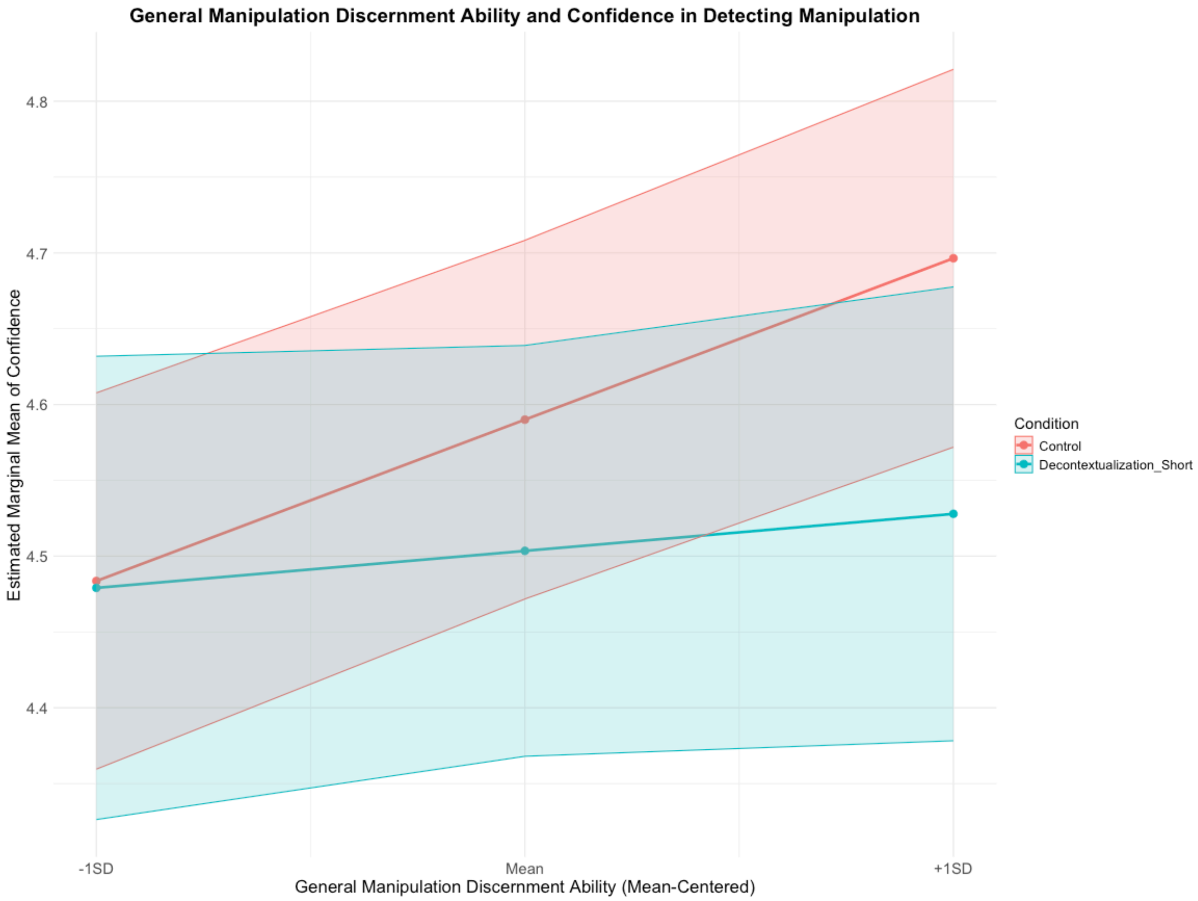

There was a significant interaction between the long decontextualization video (vs. control) and longitude when predicting confidence in detecting manipulation, $b = 0.01$, $SE = 0.01$, $t(16{,}130) = 2.52$, $p = .012$, such that the effect was only negative and significant among more Western, $b = -0.16$, $SE = 0.04$, $p < .001$, and central European nations, $b = -0.10$, $SE = 0.03$, $p = .008$, but not among more Eastern European nations, $b = -0.03$, $SE = 0.04$, $p = .999$ (see Figure S80).

Figure S80.

Simple Slopes Plot of the Interaction Effect between the Long Decontextualization Video (vs. Control) and Longitude when Predicting Confidence in Detecting Manipulation.

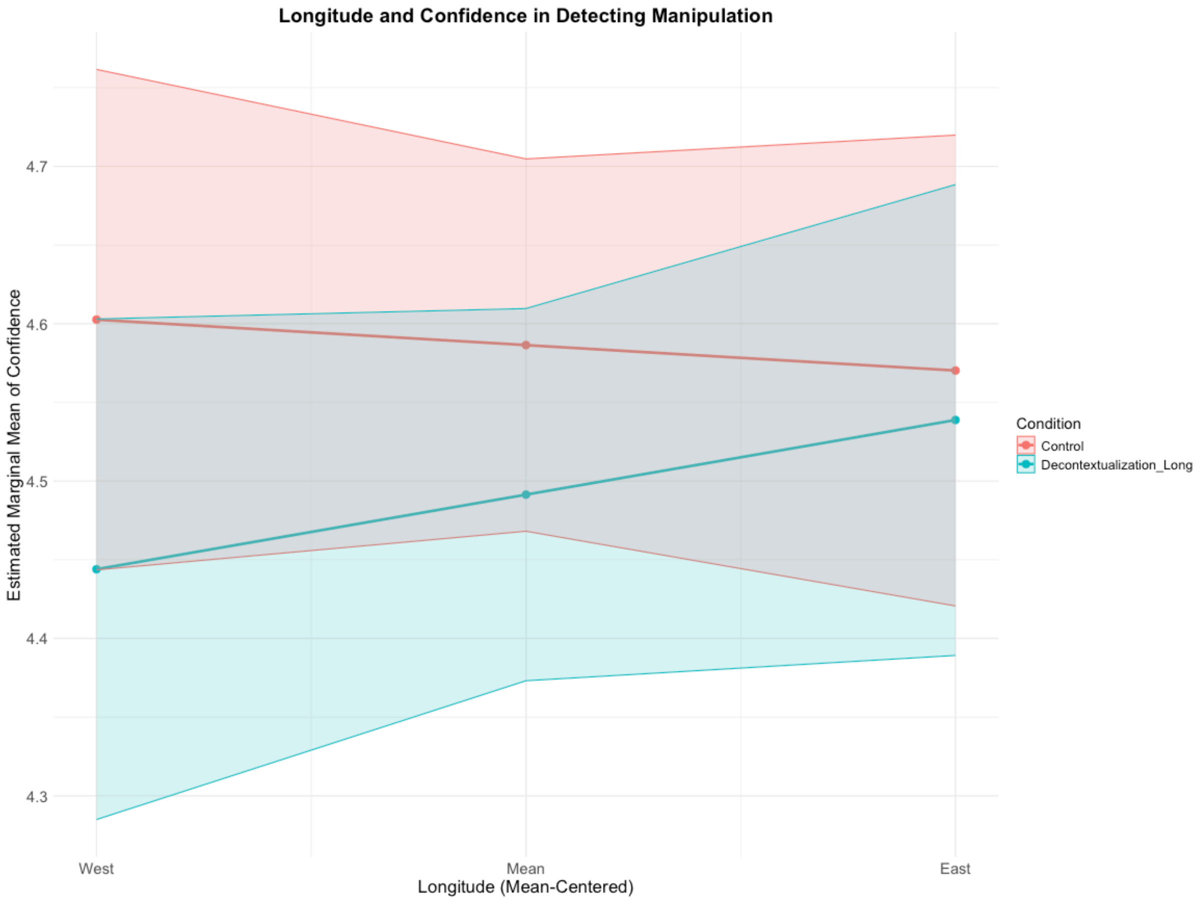

There were significant interactions between both the long, *b* = -1.18, *SE* = 0.49, *t*(16,130) = 2.43, *p* = .01, and short decontextualization videos (vs. control), *b* = -4.27, *SE* = 1.99, *t*(15,330) = 2.15, *p* = .032, and the education indices of nations when predicting confidence in detecting manipulation (see Figure S81). For the long decontextualization video (vs. control), the effect was only negative and significant at higher, *b* = -0.16, *SE* = 0.04, *p* < .001, and more moderate levels of education index, *b* = -0.10, *SE* = 0.03, *p* = .005, but not at lower levels, *b* = -0.04, *SE* = 0.04, *p* = .999 (see Figure S81). For the short decontextualization video (vs. control), the effect was only negative among nations with higher education indices, *b* = -0.22, *SE* = 0.07, *p* = .030, but not among nations with more moderate, *b* = 0.01, *SE* = 0.06, *p* = .999, or lower levels, *b* = 0.23, *SE* = 0.16, *p* = .999 (see Figure S81).

Figure S81.

Simple Slopes Plot of the Interaction Effects between Both the Long (Left) and Short (Right) Decontextualization Videos (vs. Control) and Education Index when Predicting Confidence in Detecting Manipulation.

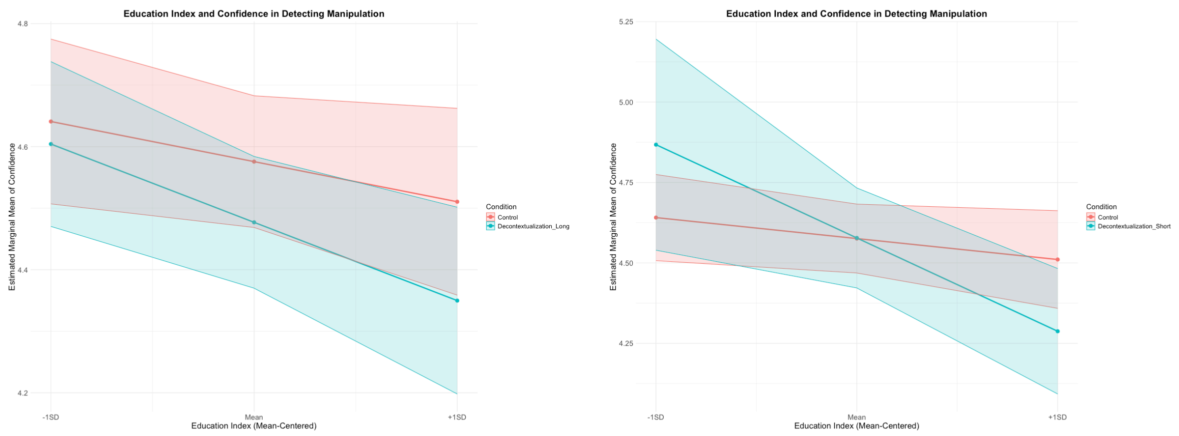

There was a significant interaction between the long decontextualization video (vs. control) and GDP per capita when predicting confidence in detecting manipulation, $b = -0.01$, $SE = 0.01$, $t(16,130) = 4.40$, $p < .001$, such that the effect was only negative and significant among nations with higher, $b = -0.22$, $SE = 0.04$, $p < .001$, and more moderate GDPs per capita, $b = -0.10$, $SE = 0.03$, $p = .002$, but not among nations with lower GDPs per capita, $b = 0.01$, $SE = 0.04$, $p = .999$ (see Figure S82).

Figure S82.

Simple Slopes Plot of the Interaction Effect between the Long Decontextualization Video (vs. Control) and GDP per capita when Predicting Confidence in Detecting Manipulation.

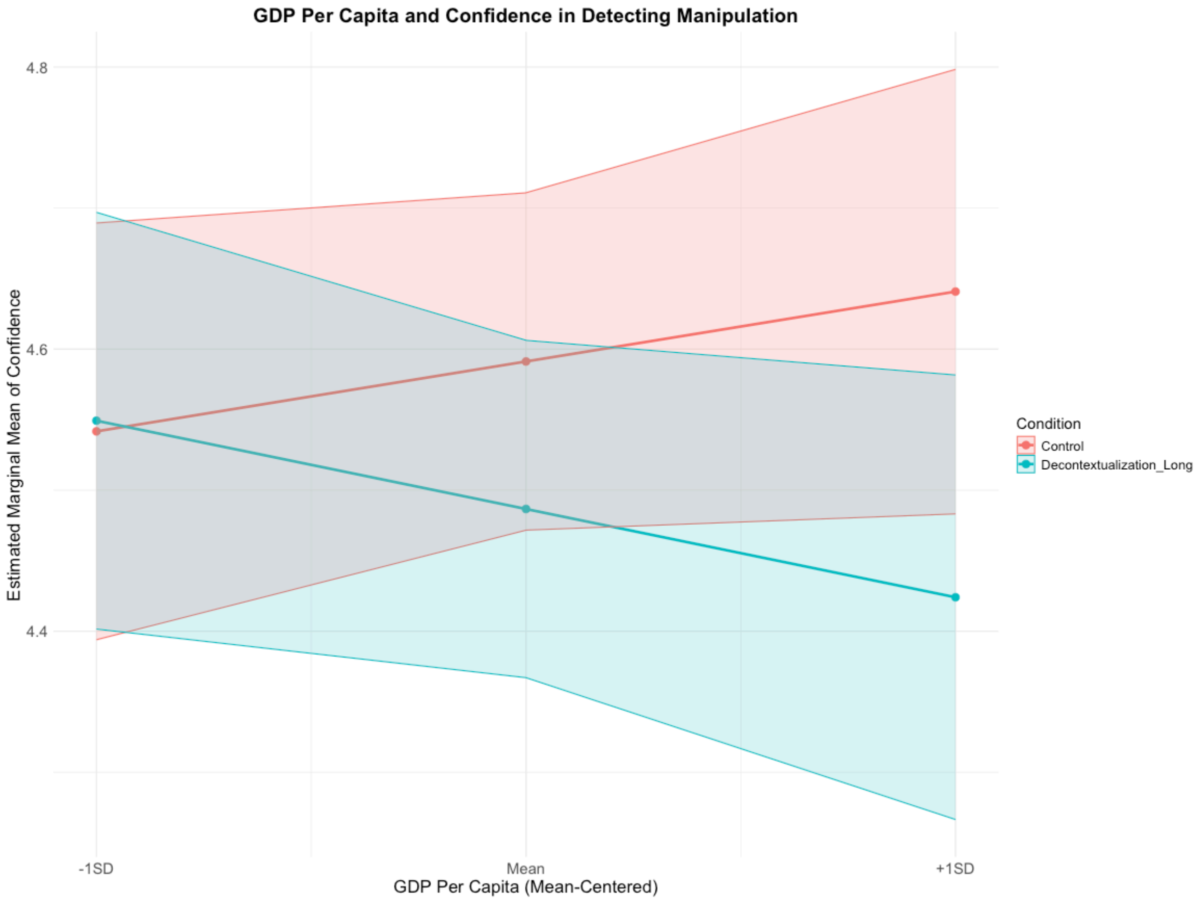

There were significant interactions between both the long, *b* = -0.10, *SE* = 0.03, *t*(16,130) = 3.10, *p* = .002, and short decontextualization videos (vs. control), *b* = -0.24, *SE* = 0.12, *t*(15,790) = 2.04, *p* = .041, and the democratic indices of nations when predicting confidence in detecting manipulation (see Figure S83). For the long decontextualization video (vs. control), the effect was only negative and significant at higher, *b* = -0.18, *SE* = 0.04, *p* < .001, and more moderate levels of democratic index, *b* = -0.10, *SE* = 0.03, *p* = .005, but not at lower levels, *b* = -0.02, *SE* = 0.04, *p* = .999 (see Figure S83). For the short decontextualization video (vs. control), the effect was only negative and significant among nations with higher democratic indices, *b* = -0.23, *SE* = 0.08, *p* = .040, but not among nations with more moderate, *b* = -0.05, *SE* = 0.05, *p* = .999, or lower levels, *b* = 0.14, *SE* = 0.12, *p* = .999 (see Figure S83).

Figure S83.

Simple Slopes Plot of the Interaction Effects between Both the Long (Left) and Short (Right) Decontextualization Videos (vs. Control) and Democratic Index when Predicting Confidence in Detecting Manipulation.

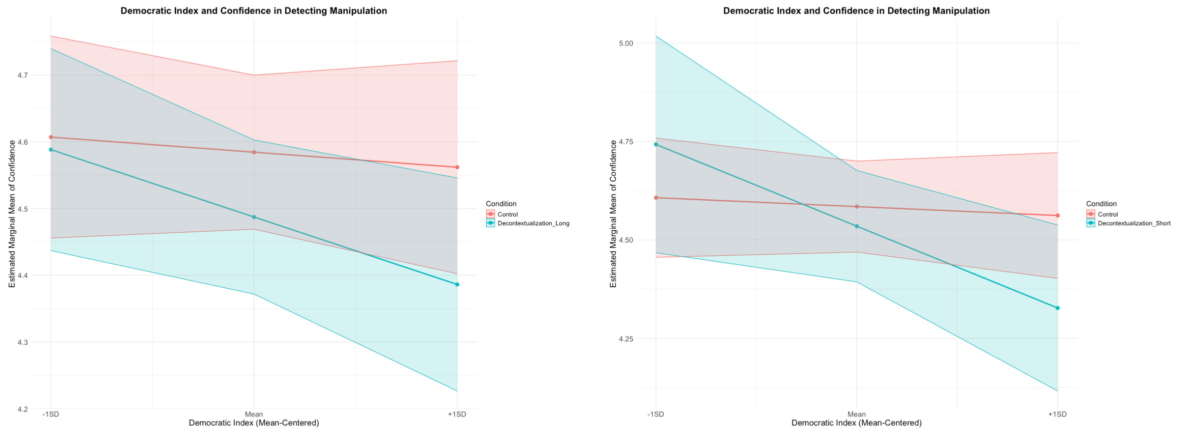

There was a significant interaction between the long decontextualization video (vs. control) and voting behaviour when predicting confidence in detecting manipulation, *b* = 0.14, *SE* = 0.07, *t*(16,120) = 1.97, *p* = .049, such that the effect was only negative and significant among those who voted in the June 2024 EU elections, *b* = -0.12, *SE* = 0.03, *p* < .001, but not among those who did not vote, *b* = 0.02, *SE* = 0.06, *p* = .999 (see Figure S84).

Figure S84.

Simple Slopes Plot of the Interaction Effect between the Long Decontextualization Video (vs. Control) and Voting Behaviour when Predicting Confidence in Detecting Manipulation.

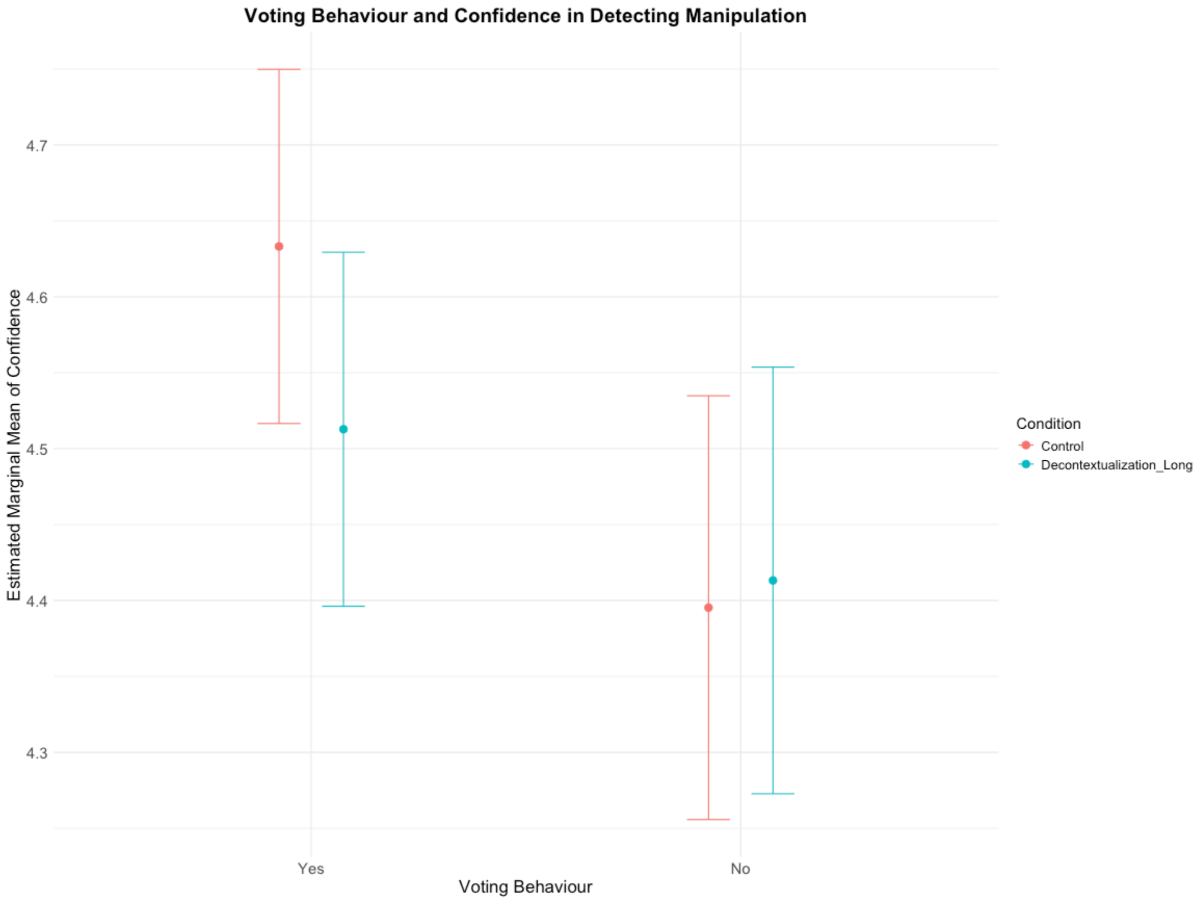

Discrediting videos

There was a significant interaction between the long discrediting video (vs. control) and age when predicting confidence in detecting manipulation, $b = 0.01$, $SE = 0.01$, $t(16,130) = 2.07$, $p = .039$, such that the effect was only positive and significant among older, $b = 0.19$, $SE = 0.04$, $p < .001$, and participants aged closer to the mean, $b = 0.13$, $SE = 0.03$, $p < .001$, but not among younger participants, $b = 0.07$, $SE = 0.04$, $p = .790$ (see Figure S85).

Figure S85.

Simple Slopes Plot of the Interaction Effect between the Long Discrediting Video (vs. Control) and Age when Predicting Confidence in Detecting Manipulation.

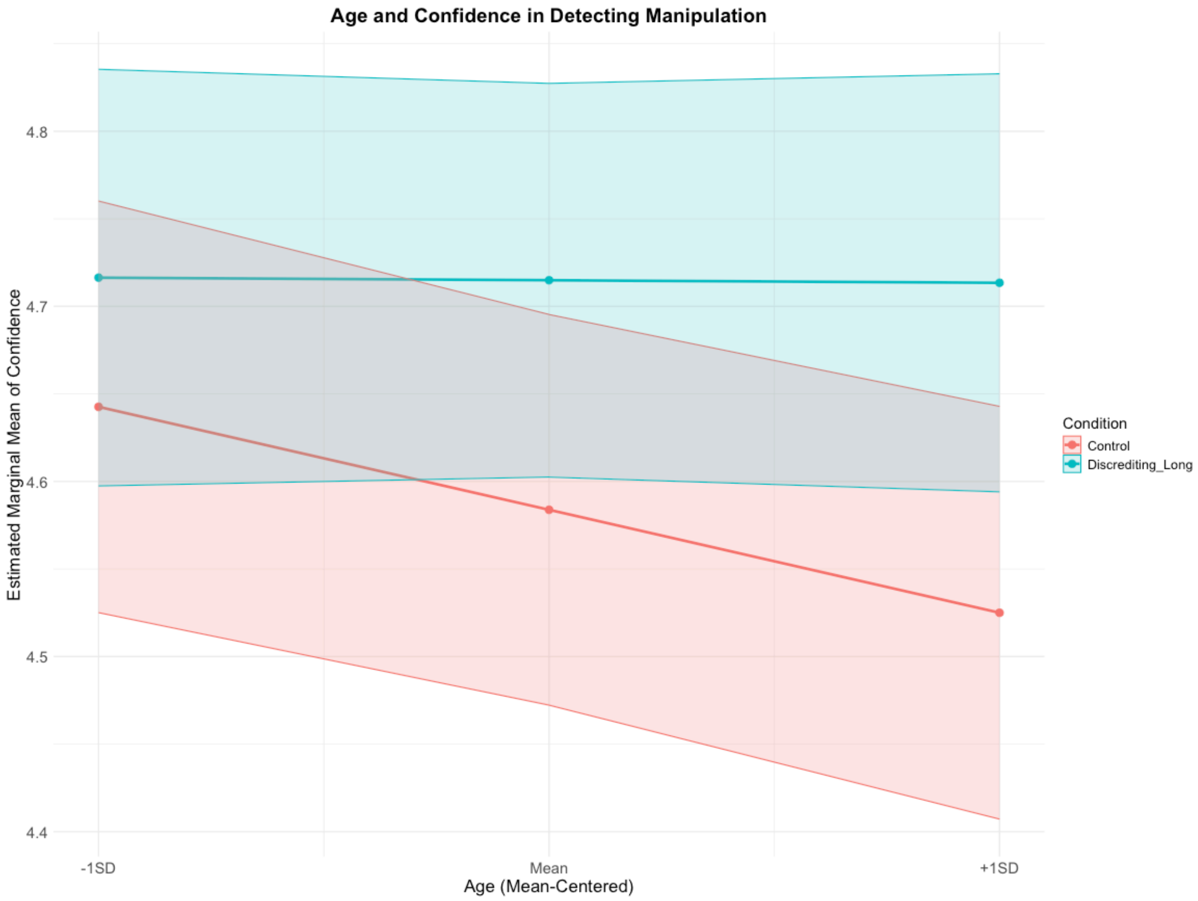

Section 3: Effects by Survey

Manipulation discernment

Manipulation discernment of the scapegoating content was only significantly increased by the long scapegoating video (vs. control) in the Belgium (Flemish), and Germany surveys, and the short scapegoating video (vs. control) did not significantly increase manipulation discernment of the scapegoating content in any of the individual surveys (see Figure S86). The long decontextualization video (vs. control) only significantly increased manipulation discernment of the decontextualization content in the Hungary, Italy, and Poland surveys, and the short decontextualization video (vs. control) did not significantly increase manipulation discernment of the decontextualization content in any of the individual surveys (see Figure S86). The long discrediting video (vs. control) only significantly increased manipulation discernment of the discrediting content in the Belgium (French), Germany, and Poland surveys, and the short discrediting video (vs. control) only significantly

increased manipulation discernment of the discrediting content in the Italy survey (see Figure S86).

Figure S86.

Forest Plot of Cohen's d Effect Sizes and 95% Confidence Intervals for the Effects of all Videos on Manipulation Discernment of Relevant Content by Survey.

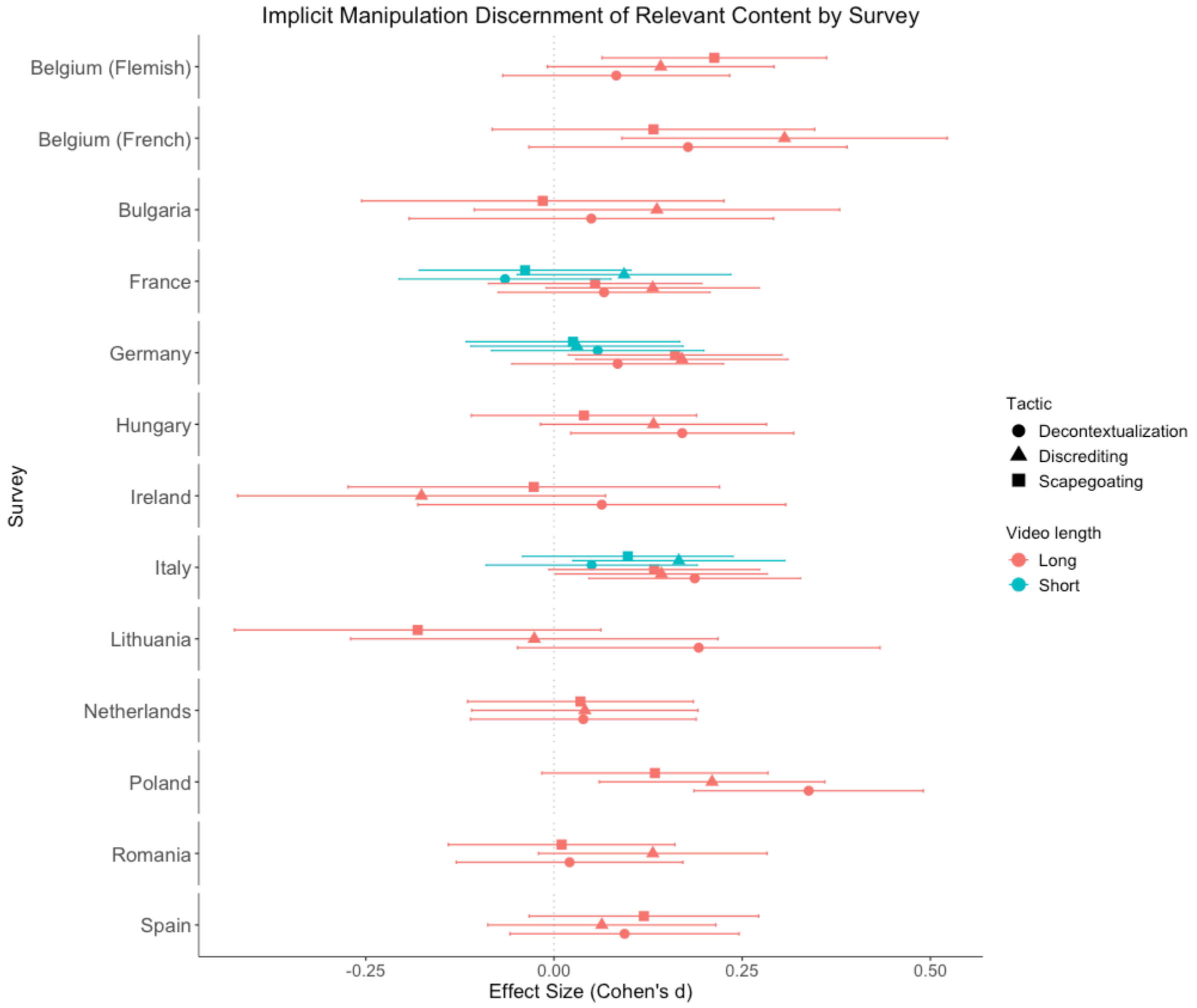

Technique discernment

Technique discernment of the scapegoating content was only significantly increased by the long scapegoating video (vs. control) in the Netherlands and Spain surveys, and the short scapegoating video (vs. control) did not significantly increase technique discernment of the scapegoating content in any of the individual surveys (see Figure S87). In fact, the long scapegoating video (vs. control) significantly decreased technique discernment of the

scapegoating content in the Lithuania survey (see Figure S87). The long decontextualization video (vs. control) only significantly increased technique discernment of the decontextualization content in the Germany, Hungary, Romania, and Spain surveys, and the short decontextualization video (vs. control) did not significantly increase technique discernment of the decontextualization content in any of the individual surveys (see Figure S87). The long discrediting video (vs. control) only significantly increased technique discernment of the discrediting content in the Bulgaria and Spain surveys, and the short discrediting video (vs. control) did not significantly increase technique discernment of the discrediting content in any of the individual surveys (see Figure S87).

Figure S87.

Forest Plot of Cohen's d Effect Sizes and 95% Confidence Intervals for the Effects of all Videos on Technique Discernment of Relevant Content by Survey

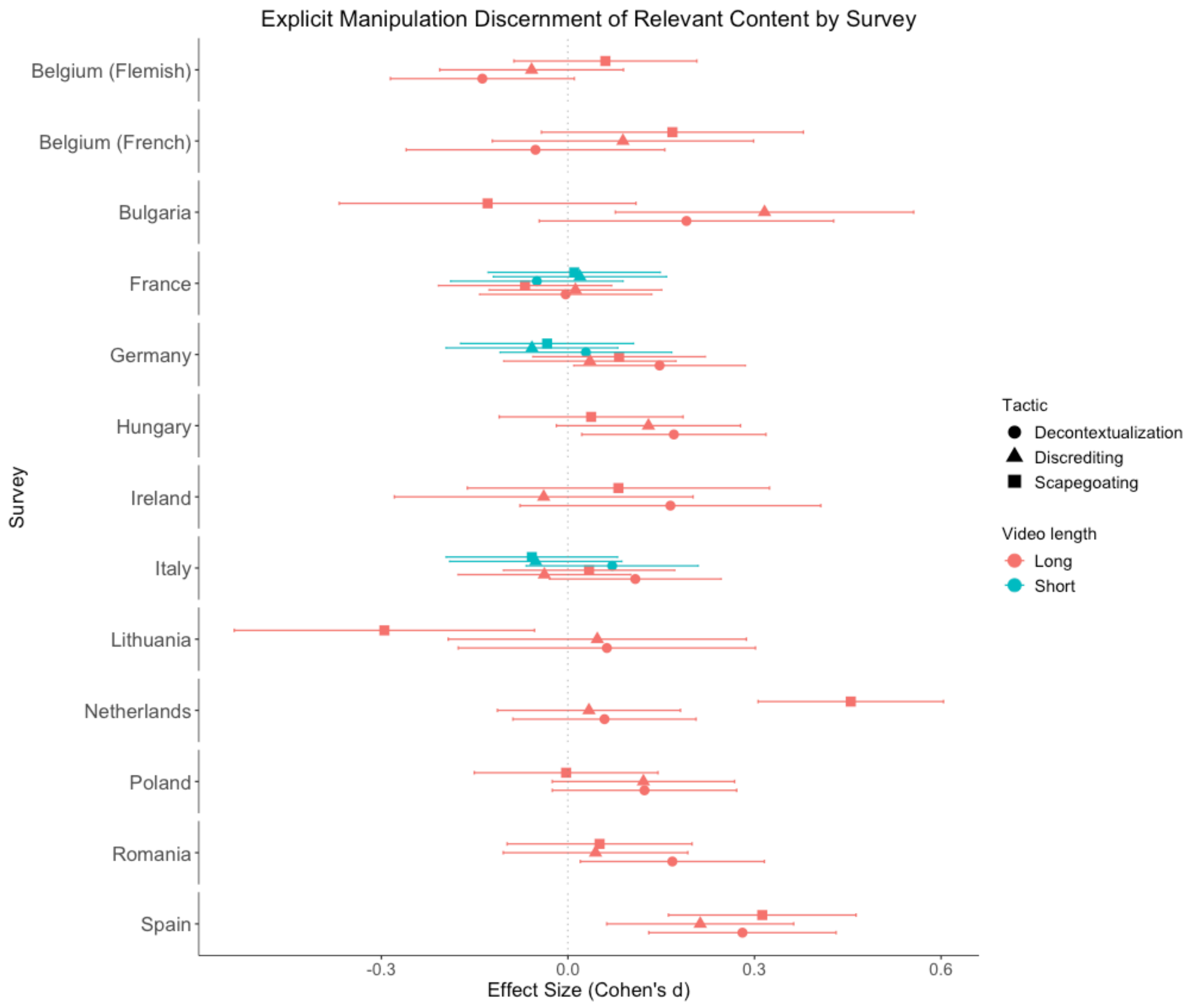

Sharing decisions

Sharing decisions for the scapegoating content were only significantly increased by the long scapegoating video (vs. control) in the Bulgaria and Spain surveys, and the short scapegoating video (vs. control) did not significantly increase sharing decisions for the scapegoating content in any of the individual surveys (see Figure S88). The long decontextualization video (vs. control) only significantly increased sharing decisions for the decontextualization content in the Belgium (French), Bulgaria, France, Germany, Italy, Poland, and Spain surveys, and the short decontextualization video (vs. control) only significantly increased sharing decisions for the decontextualization content in the Germany survey (see Figure S88). The long discrediting video (vs. control) only significantly increased sharing decisions for the discrediting content in the Belgium (Flemish), Bulgaria, France,

Germany, Hungary, Ireland, Italy, and Spain surveys, and the short discrediting video (vs. control) only significantly increased sharing decisions for the discrediting content in the Germany survey (see Figure S88).

Figure S88.

Forest Plot of Cohen's d Effect Sizes and 95% Confidence Intervals for the Effects of all Videos on Sharing Decisions for Relevant Content by Survey

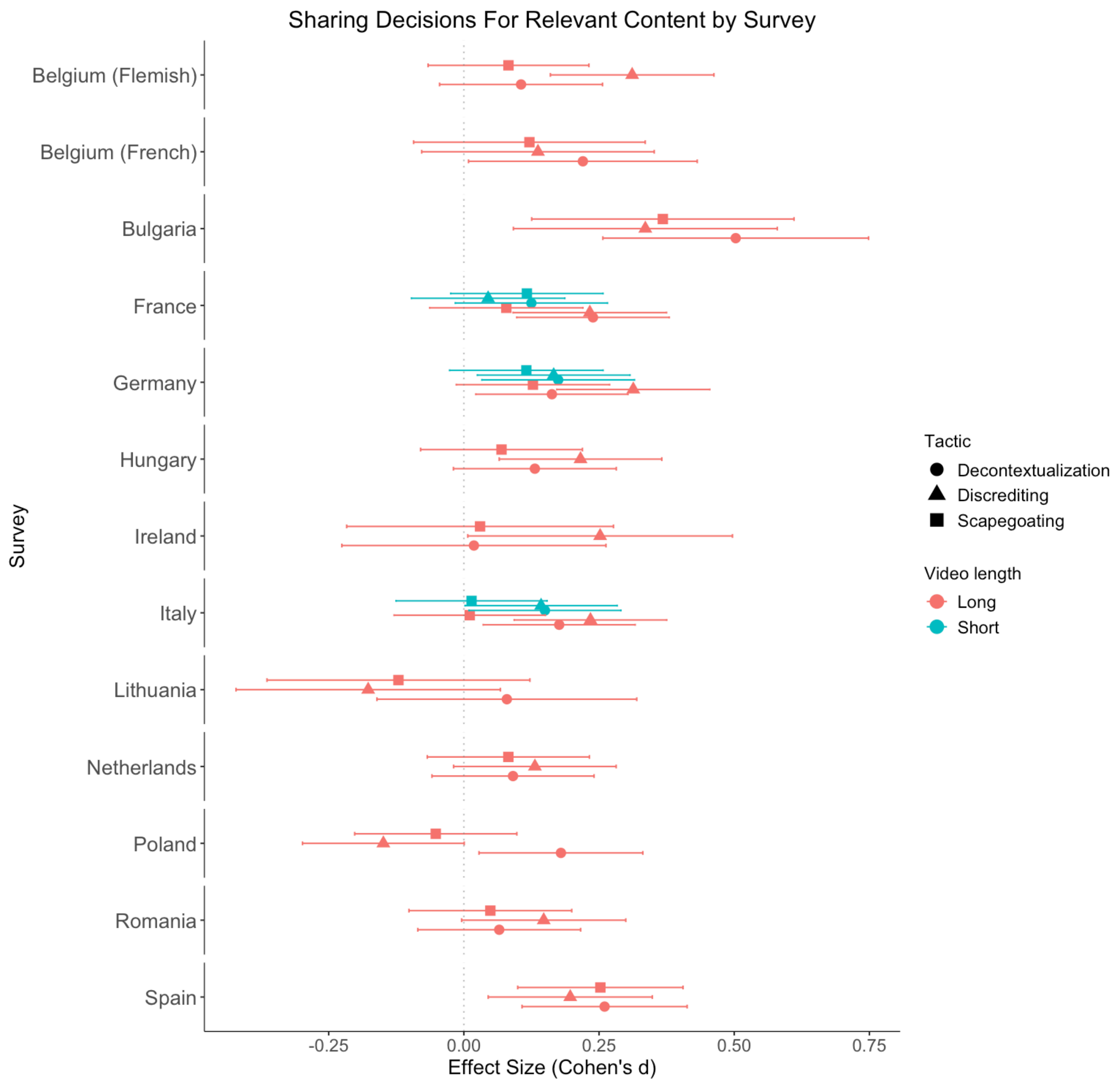

Section 4: Study materials

Item rating task

Manipulative scapegoating content

"Local businesses failing because of teens' online shopping addiction!"

	Strongly disagree 1	2	3	Neither agree nor disagree 4	5	6	Strongly agree 7
This statement is manipulative.	○	○	○	○	○	○	○
I would share this statement with people in my network.	○	○	○	○	○	○	○

This statement uses...

- ○ False dichotomy
- ○ Scapegoating
- ○ Fearmongering
- ○ None of the above

"Traffic jams are worse because cyclists are taking up road space."

	Strongly disagree 1	2	3	Neither agree nor disagree 4	5	6	Strongly agree 7
This statement is manipulative.	○	○	○	○	○	○	○
I would share this statement with people in my network.	○	○	○	○	○	○	○

This statement uses...

- ○ Emotional language
- ○ Fearmongering
- ○ Scapegoating
- ○ None of the above

"Record-high healthcare costs are due to patients getting unneeded tests."

	Strongly disagree 1	2	3	Neither agree nor disagree 4	5	6	Strongly agree 7
This statement is manipulative.	○	○	○	○	○	○	○
I would share this statement with people in my network.	○	○	○	○	○	○	○

This statement uses...

- ○ Cherry picking
- ○ Medical malpractice
- ○ Scapegoating
- ○ None of the above

"Unemployment rates are rising because immigrants are taking all the jobs."

	Strongly disagree 1	2	3	Neither agree nor disagree 4	5	6	Strongly agree 7
This statement is manipulative.	○	○	○	○	○	○	○
I would share this statement with people in my network.	○	○	○	○	○	○	○

This statement uses...

- ○ Scapegoating
- ○ False dichotomy
- ○ Polarization
- ○ None of the above

"Poor educational outcomes are the result of students spending too much time on social media."

	Strongly disagree 1	2	3	Neither agree nor disagree 4	5	6	Strongly agree 7
This statement is manipulative.	○	○	○	○	○	○	○
I would share this statement with people in my network.	○	○	○	○	○	○	○

This statement uses...

- ○ Scapegoating
- ○ False dichotomy
- ○ Fearmongering
- ○ None of the above

"Climate change is accelerating because of pet owners not properly disposing of pet waste."

	Strongly disagree 1	2	3	Neither agree nor disagree 4	5	6	Strongly agree 7
This statement is manipulative.	○	○	○	○	○	○	○
I would share this statement with people in my network.	○	○	○	○	○	○	○

This statement uses...

- ○ Scapegoating
- ○ Circular logic
- ○ Science denial
- ○ None of the above

"Crime rates are increasing because of the rise in popularity of violent video games."

	Strongly disagree			Neither agree nor disagree			Strongly agree
	1	2	3	4	5	6	7
This statement is manipulative.	○	○	○	○	○	○	○
I would share this statement with people in my network.	○	○	○	○	○	○	○

This statement uses...

- ○ Character assassination
- ○ Scapegoating
- ○ False dichotomy
- ○ None of the above

"City pollution levels are high because of people's obsession with food delivery services."

	Strongly disagree			Neither agree nor disagree			Strongly agree
	1	2	3	4	5	6	7
This statement is manipulative.	○	○	○	○	○	○	○
I would share this statement with people in my network.	○	○	○	○	○	○	○

This statement uses...

- ○ Scapegoating
- ○ Emotional language
- ○ Character assassination
- ○ None of the above

Non-manipulative scapegoating content

"Around 5.8% of the US general population currently suffers from shopping addiction."

	Strongly disagree			Neither agree nor disagree			Strongly agree
	1	2	3	4	5	6	7
This statement is manipulative.	○	○	○	○	○	○	○
I would share this statement with people in my network.	○	○	○	○	○	○	○

This statement uses...

- ○ False dichotomy
- ○ Fearmongering
- ○ Scapegoating
- ○ None of the above

"Without new policies, greenhouse gas emissions are set to rise by 50% in 2050."

	Strongly disagree			Neither agree nor disagree			Strongly agree
	1	2	3	4	5	6	7
This statement is manipulative.	○	○	○	○	○	○	○
I would share this statement with people in my network.	○	○	○	○	○	○	○

This statement uses...

- ○ Science denial
- ○ Scapegoating
- ○ Circular logic
- ○ None of the above

"The majority of cyclist fatalities occur on urban roads."

	Strongly disagree			Neither agree nor disagree			Strongly agree
	1	2	3	4	5	6	7
This statement is manipulative.	○	○	○	○	○	○	○
I would share this statement with people in my network.	○	○	○	○	○	○	○

This statement uses...

- ○ Emotional language
- ○ Fearmongering
- ○ Scapegoating
- ○ None of the above

"High medical costs esimated to be responsible for 6.4% of cases where people don't get vital treatment."

	Strongly disagree			Neither agree nor disagree			Strongly agree
	1	2	3	4	5	6	7
This statement is manipulative.	○	○	○	○	○	○	○
I would share this statement with people in my network.	○	○	○	○	○	○	○

This statement uses...

- ○ Medical malpractice
- ○ Cherry picking
- ○ Scapegoating
- ○ None of the above

"More than 50% of video games contain violence."

	Strongly disagree 1	2	3	Neither agree nor disagree 4	5	6	Strongly agree 7
This statement is manipulative.	○	○	○	○	○	○	○
I would share this statement with people in my network.	○	○	○	○	○	○	○

This statement uses...

- ○ Character assassination
- ○ False dichotomy
- ○ Scapegoating
- ○ None of the above

"Adult unemployment rates for men and women are similar in the US."

	Strongly disagree 1	2	3	Neither agree nor disagree 4	5	6	Strongly agree 7
This statement is manipulative.	○	○	○	○	○	○	○
I would share this statement with people in my network.	○	○	○	○	○	○	○

This statement uses...

- ○ False dichotomy
- ○ Scapegoating
- ○ Polarization
- ○ None of the above

"Worldwide food delivery market set to grow by almost 10% between 2024 and 2029."

	Strongly disagree 1	2	3	Neither agree nor disagree 4	5	6	Strongly agree 7
This statement is manipulative.	○	○	○	○	○	○	○
I would share this statement with people in my network.	○	○	○	○	○	○	○

This statement uses...

- ○ Scapegoating
- ○ Character assassination
- ○ Emotional language
- ○ None of the above

"More than half the world's population use social media."

	Strongly disagree 1	2	3	Neither agree nor disagree 4	5	6	Strongly agree 7
This statement is manipulative.	○	○	○	○	○	○	○
I would share this statement with people in my network.	○	○	○	○	○	○	○

This statement uses...

- ○ False dichotomy
- ○ Scapegoating
- ○ Fearmongering
- ○ None of the above

Manipulative decontextualization content

You see a clip where a comedian is said to have made a fan cry.

	Strongly disagree 1	2	3	Neither agree nor disagree 4	5	6	Strongly agree 7
This clip is manipulative.	○	○	○	○	○	○	○
I would share this clip with people in my network.	○	○	○	○	○	○	○

This clip uses...

- ○ Political backsliding
- ○ Decontextualisation
- ○ Economic corruption
- ○ None of the above

You see a peaceful photo with the headline "Violent protest against the law!"

	Strongly disagree 1	2	3	Neither agree nor disagree 4	5	6	Strongly agree 7
This headline is manipulative.	○	○	○	○	○	○	○
I would share this headline with people in my network.	○	○	○	○	○	○	○

This headline uses...

- ○ Oversimplification
- ○ Call for action
- ○ Decontextualisation
- ○ None of the above

You see a funny headline inserted on a serious photo.

	Strongly disagree 1	2	3	Neither agree nor disagree 4	5	6	Strongly agree 7
This headline is manipulative.	○	○	○	○	○	○	○
I would share this headline with people in my network.	○	○	○	○	○	○	○

This headline uses...

- ○ Decontextualisation
- ○ Oversimplification
- ○ Social media analysis
- ○ None of the above

You see a photo of a broken climbing frame in a park with the headline "City in panic over park safety concerns!"

	Strongly disagree 1	2	3	Neither agree nor disagree 4	5	6	Strongly agree 7
This headline is manipulative.	○	○	○	○	○	○	○
I would share this headline with people in my network.	○	○	○	○	○	○	○

This headline uses...

- ○ Local action
- ○ Polarization
- ○ Decontextualisation
- ○ None of the above

You see a photo of some litter in the street with the headline "Neighborhood descending into chaos due to littering!"

	Strongly disagree 1	2	3	Neither agree nor disagree 4	5	6	Strongly agree 7
This headline is manipulative.	○	○	○	○	○	○	○
I would share this headline with people in my network.	○	○	○	○	○	○	○

This headline uses...

- ○ Decontextualisation
- ○ Slander
- ○ False dichotomy
- ○ None of the above

You see a photo of a crowded subway station with the headline "Mass exodus from the city due to fear of natural disaster!"

	Strongly disagree 1	2	3	Neither agree nor disagree 4	5	6	Strongly agree 7
This headline is manipulative.	○	○	○	○	○	○	○
I would share this headline with people in my network.	○	○	○	○	○	○	○

This headline uses...

- ○ Call for action
- ○ Decontextualisation
- ○ Character assassination
- ○ None of the above

You see a picture of people waiting in line with the headline "Chaotic scenes as crowds clash over tickets!"

	Strongly disagree 1	2	3	Neither agree nor disagree 4	5	6	Strongly agree 7
This headline is manipulative.	○	○	○	○	○	○	○
I would share this headline with people in my network.	○	○	○	○	○	○	○

This headline uses...

- ○ Decontextualisation
- ○ Character assassination
- ○ Social media drama
- ○ None of the above

You see a photo of a family having a peaceful dinner with the headline "Angry domestic dispute leads to police intervention!"

	Strongly disagree 1	2	3	Neither agree nor disagree 4	5	6	Strongly agree 7
This headline is manipulative.	○	○	○	○	○	○	○
I would share this headline with people in my network.	○	○	○	○	○	○	○

This headline uses...

- ○ Character assassination
- ○ Decontextualisation
- ○ Cherry picking
- ○ None of the above

Non-manipulative decontextualization content

You see a clip of politicians arguing over inflation.

	Strongly disagree 1	2	3	Neither agree nor disagree 4	5	6	Strongly agree 7
This clip is manipulative.	○	○	○	○	○	○	○
I would share this clip with people in my network.	○	○	○	○	○	○	○

This clip uses...

- ○ Political backsliding
- ○ Economic corruption
- ○ Decontextualisation
- ○ None of the above

The see a photo of a family having dinner with the headline "Tasty recipes that will impress your family at the reunion".

	Strongly disagree 1	2	3	Neither agree nor disagree 4	5	6	Strongly agree 7
This headline is manipulative.	○	○	○	○	○	○	○
I would share this headline with people in my network.	○	○	○	○	○	○	○

This headline uses...

- ○ Decontextualisation
- ○ Character assassination
- ○ Cherry picking
- ○ None of the above

You see a photo of some litter in the street with the headline "Excess littering can frustrate local residents".

	Strongly disagree 1	2	3	Neither agree nor disagree 4	5	6	Strongly agree 7
This headline is manipulative.	○	○	○	○	○	○	○
I would share this headline with people in my network.	○	○	○	○	○	○	○

This headline uses...

- ○ Decontextualisation
- ○ Slander
- ○ False dichotomy
- ○ None of the above

You see a picture of people waiting in line with the headline "People still attending concerts despite cost of living".

	Strongly disagree 1	2	3	Neither agree nor disagree 4	5	6	Strongly agree 7
This headline is manipulative.	○	○	○	○	○	○	○
I would share this headline with people in my network.	○	○	○	○	○	○	○

This headline uses...

- ○ Social media drama
- ○ Decontextualisation
- ○ Character assassination
- ○ None of the above

You see a photo of a climbing frame in a park with the headline "Best parks to take your kids to".

	Strongly disagree 1	2	3	Neither agree nor disagree 4	5	6	Strongly agree 7
This headline is manipulative.	○	○	○	○	○	○	○
I would share this headline with people in my network.	○	○	○	○	○	○	○

This headline uses...

- ○ Decontextualisation
- ○ Local action
- ○ Polarization
- ○ None of the above

You see a photo of a subway train with the headline "New subway trains to be introduced".

	Strongly disagree 1	2	3	Neither agree nor disagree 4	5	6	Strongly agree 7
This headline is manipulative.	○	○	○	○	○	○	○
I would share this headline with people in my network.	○	○	○	○	○	○	○

This headline uses...

- ○ Decontextualisation
- ○ Call for action
- ○ Character assassination
- ○ None of the above

You see a serious headline above a serious photo.

	Strongly disagree 1	2	3	Neither agree nor disagree 4	5	6	Strongly agree 7
This headline is manipulative.	○	○	○	○	○	○	○
I would share this headline with people in my network.	○	○	○	○	○	○	○

This headline uses...

- ○ Oversimplification
- ○ Decontextualisation
- ○ Social media analysis
- ○ None of the above

You see a photo of protestors with the headline "Protests over new laws".

	Strongly disagree 1	2	3	Neither agree nor disagree 4	5	6	Strongly agree 7
This headline is manipulative.	○	○	○	○	○	○	○
I would share this headline with people in my network.	○	○	○	○	○	○	○

This headline uses...

- ○ Oversimplification
- ○ Call for action
- ○ Decontextualisation
- ○ None of the above

Manipulative discrediting content

"Athlete's views on fitness shouldn't be considered since they were caught running a red light."

	Strongly disagree 1	2	3	Neither agree nor disagree 4	5	6	Strongly agree 7
This statement is manipulative.	○	○	○	○	○	○	○
I would share this statement with people in my network.	○	○	○	○	○	○	○

This statement uses...

- ○ Fearmongering
- ○ Discrediting
- ○ Fake outrage
- ○ None of the above

"That architect's building designs can't be trusted since they were late on their taxes last year."

	Strongly disagree 1	2	3	Neither agree nor disagree 4	5	6	Strongly agree 7
This statement is manipulative.	○	○	○	○	○	○	○
I would share this statement with people in my network.	○	○	○	○	○	○	○

This statement uses...

- ○ Discrediting
- ○ Cherry picking
- ○ Hasty generalisation
- ○ None of the above

"This Professor's theories on economics are unreliable because they're divorced."

	Strongly disagree 1	2	3	Neither agree nor disagree 4	5	6	Strongly agree 7
This statement is manipulative.	○	○	○	○	○	○	○
I would share this statement with people in my network.	○	○	○	○	○	○	○

This statement uses...

- ○ Polarization
- ○ Circular reasoning
- ○ Discrediting
- ○ None of the above

"Chef's opinion on nutrition is invalid because they had a failed restaurant ten years ago."

	Strongly disagree 1	2	3	Neither agree nor disagree 4	5	6	Strongly agree 7
This statement is manipulative.	○	○	○	○	○	○	○
I would share this statement with people in my network.	○	○	○	○	○	○	○

This statement uses...

- ○ Discrediting
- ○ Fearmongering
- ○ Hasty generalisation
- ○ None of the above

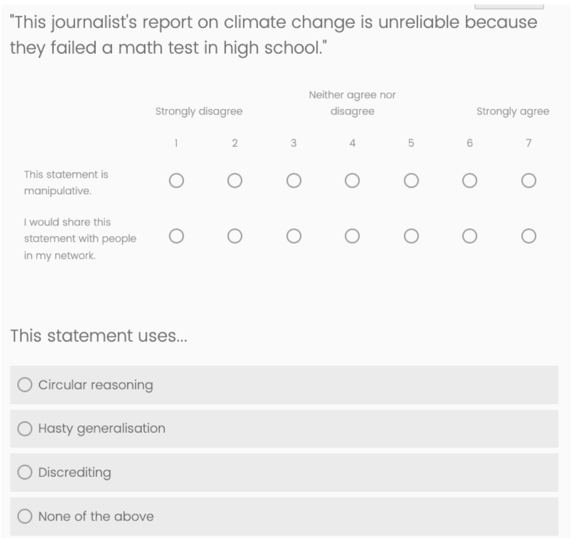

"This journalist's report on climate change is unreliable because they failed a math test in high school."

	Strongly disagree 1	2	3	Neither agree nor disagree 4	5	6	Strongly agree 7
This statement is manipulative.	○	○	○	○	○	○	○
I would share this statement with people in my network.	○	○	○	○	○	○	○

This statement uses...

- ○ Circular reasoning
- ○ Hasty generalisation
- ○ Discrediting
- ○ None of the above

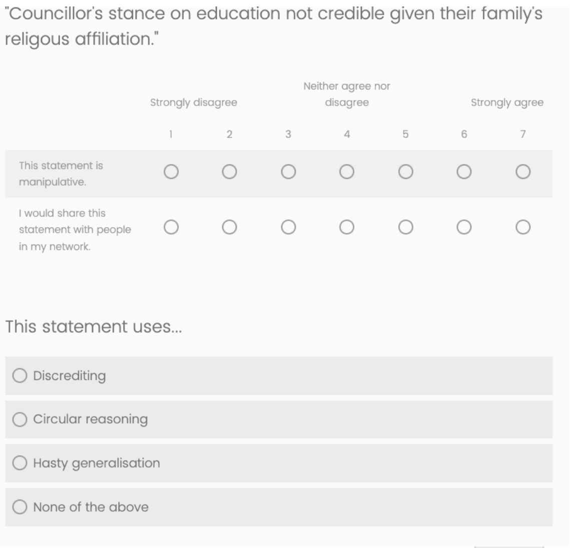

"Councillor's stance on education not credible given their family's religous affiliation."

	Strongly disagree 1	2	3	Neither agree nor disagree 4	5	6	Strongly agree 7
This statement is manipulative.	○	○	○	○	○	○	○
I would share this statement with people in my network.	○	○	○	○	○	○	○

This statement uses...

- ○ Discrediting
- ○ Circular reasoning
- ○ Hasty generalisation
- ○ None of the above

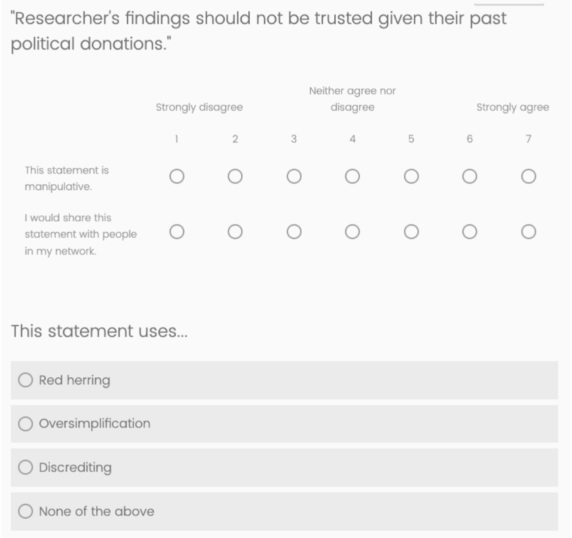

"Researcher's findings should not be trusted given their past political donations."

	Strongly disagree 1	2	3	Neither agree nor disagree 4	5	6	Strongly agree 7
This statement is manipulative.	○	○	○	○	○	○	○
I would share this statement with people in my network.	○	○	○	○	○	○	○

This statement uses...

- ○ Red herring
- ○ Oversimplification
- ○ Discrediting
- ○ None of the above

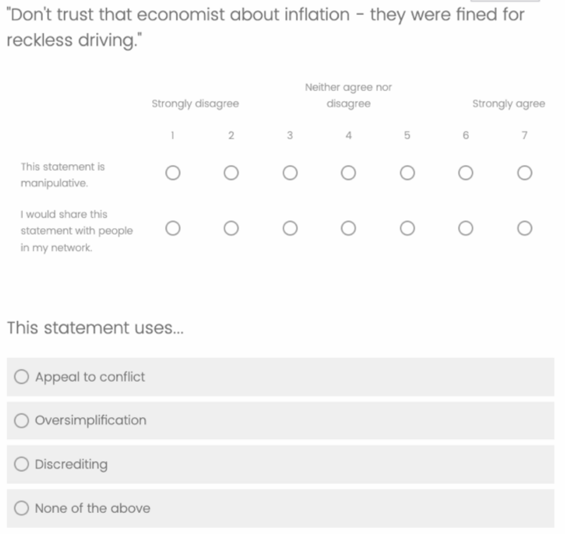

"Don't trust that economist about inflation - they were fined for reckless driving."

	Strongly disagree 1	2	3	Neither agree nor disagree 4	5	6	Strongly agree 7
This statement is manipulative.	○	○	○	○	○	○	○
I would share this statement with people in my network.	○	○	○	○	○	○	○

This statement uses...

- ○ Appeal to conflict
- ○ Oversimplification
- ○ Discrediting
- ○ None of the above

Non-manipulative discrediting content

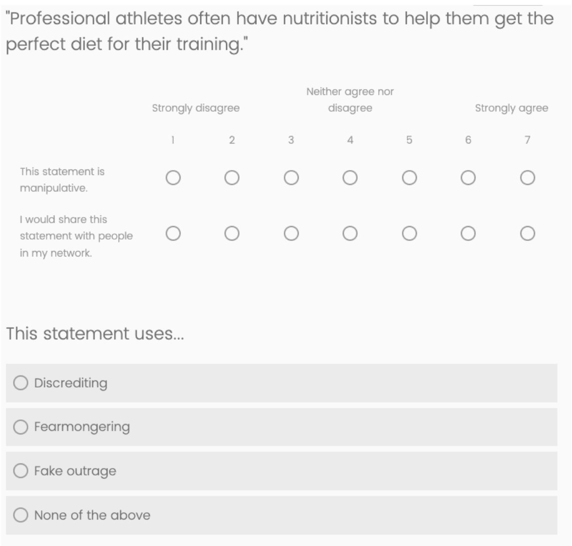

"Professional athletes often have nutritionists to help them get the perfect diet for their training."

	Strongly disagree 1	2	3	Neither agree nor disagree 4	5	6	Strongly agree 7
This statement is manipulative.	○	○	○	○	○	○	○
I would share this statement with people in my network.	○	○	○	○	○	○	○

This statement uses...

- ○ Discrediting
- ○ Fearmongering
- ○ Fake outrage
- ○ None of the above

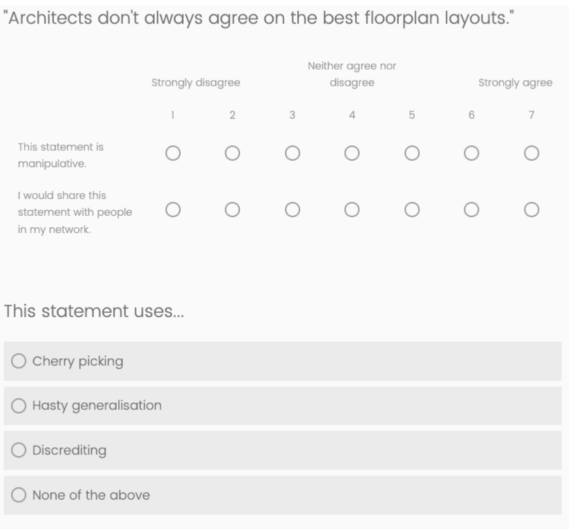

"Architects don't always agree on the best floorplan layouts."

	Strongly disagree 1	2	3	Neither agree nor disagree 4	5	6	Strongly agree 7
This statement is manipulative.	○	○	○	○	○	○	○
I would share this statement with people in my network.	○	○	○	○	○	○	○

This statement uses...

- ○ Cherry picking
- ○ Hasty generalisation
- ○ Discrediting
- ○ None of the above

"Individuals with higher education levels may have lower divorce rates."

	Strongly disagree			Neither agree nor disagree			Strongly agree
	1	2	3	4	5	6	7
This statement is manipulative.	○	○	○	○	○	○	○
I would share this statement with people in my network.	○	○	○	○	○	○	○

This statement uses...

- ○ Discrediting
- ○ Polarization
- ○ Circular reasoning
- ○ None of the above

"Some journalists write more about climate change than others."

	Strongly disagree			Neither agree nor disagree			Strongly agree
	1	2	3	4	5	6	7
This statement is manipulative.	○	○	○	○	○	○	○
I would share this statement with people in my network.	○	○	○	○	○	○	○

This statement uses...

- ○ Circular reasoning
- ○ Hasty generalisation
- ○ Discrediting
- ○ None of the above

"Researchers work hard to receive funding."

	Strongly disagree			Neither agree nor disagree			Strongly agree
	1	2	3	4	5	6	7
This statement is manipulative.	○	○	○	○	○	○	○
I would share this statement with people in my network.	○	○	○	○	○	○	○

This statement uses...

- ○ Discrediting
- ○ Oversimplification
- ○ Red herring
- ○ None of the above

"Vegan chefs cite healthy eating as one inspiration."

	Strongly disagree			Neither agree nor disagree			Strongly agree
	1	2	3	4	5	6	7
This statement is manipulative.	○	○	○	○	○	○	○
I would share this statement with people in my network.	○	○	○	○	○	○	○

This statement uses...

- ○ Discrediting
- ○ Fearmongering
- ○ Hasty generalisation
- ○ None of the above

"Christians vote both Democrat and Republican in the US."

	Strongly disagree			Neither agree nor disagree			Strongly agree
	1	2	3	4	5	6	7
This statement is manipulative.	○	○	○	○	○	○	○
I would share this statement with people in my network.	○	○	○	○	○	○	○

This statement uses...

- ○ Hasty generalisation
- ○ Circular reasoning
- ○ Discrediting
- ○ None of the above

"Economists explain how recessions occur."

	Strongly disagree			Neither agree nor disagree			Strongly agree
	1	2	3	4	5	6	7
This statement is manipulative.	○	○	○	○	○	○	○
I would share this statement with people in my network.	○	○	○	○	○	○	○

This statement uses...

- ○ Oversimplification
- ○ Discrediting
- ○ Appeal to conflict
- ○ None of the above

Digital literacy scale

Please indicate how well each of the following statements describes you, from 1 (not at all true of me) to 5 (very true of me).

1. I know how to send and join a video meeting invitation.
2. I know how to create and collaborate on digital documents.

3. I know how to edit videos and/or pictures.
4. I know how to bookmark a website.
5. I know how to use shortcut keys (e.g., CTRL + V).
6. I know how to open downloaded files.
7. I know how to download/save a photo I found online.
8. I know how to open a new tab in my browser.

Political tolerance scale

Please indicate your level of agreement with the following statements, from 1 (strongly disagree) to 7 (strongly agree).

1. People who disagree with me politically deserve the same rights as I do.
2. *Freedom of speech should not be granted to those who threaten government stability.*
3. Governments should treat all people equally regardless of their beliefs.
4. *Radical beliefs should be suppressed by our government.*
5. *People who are intolerant of others do not deserve freedom of speech.*
6. People with beliefs that are outside of the norm have a right to express their views.

Note. Italicisation denotes reverse-coded items.

Section 5: Comparisons between participants who had seen, had not seen, or who were unsure of whether they had seen the videos

Scapegoating content

Manipulativeness assessments

Manipulativeness assessments of the manipulative scapegoating content were significantly higher among participants who had seen the video before (vs. not seen), b = 0.21, SE = 0.09, t(18,280) = 2.27, p = .023, participants who had seen the video before (vs. unsure), b = 0.39, SE = 0.12, t(18,280) = 3.28, p = .001, and participants who had not seen the video before (vs. unsure), b = 0.18, SE = 0.08, t(18,270) = 2.27, p = .023. Manipulativeness assessments of the non-manipulative scapegoating content were significantly higher among participants who had seen the video before (vs. not seen), b = 0.40, SE = 0.08, t(18,650) = 5.02, p < .001, and unsure whether they had seen the video before (vs. not seen), b = 0.21, SE = 0.07, t(18,650) = 3.12, p = .002. Manipulation discernment of the scapegoating content was significantly lower among participants who were unsure whether they had seen the video before (vs. not seen), b = -0.41, SE = 0.10, t(17,550) = 4.05, p < .001.

Technique recognition

Technique recognition of the manipulative scapegoating content was significantly lower among participants who had seen the video before (vs. not seen), $b = -0.07$, $SE = 0.02$, $t(18{,}640) = 3.63$, $p < .001$, and participants who were unsure whether they had seen the video before (vs. not seen), $b = -0.05$, $SE = 0.02$, $t(18{,}640) = 3.00$, $p = .003$. Technique recognition of the non-manipulative scapegoating content was significantly lower among participants who had seen the video before (vs. not seen), $b = -0.12$, $SE = 0.02$, $t(18{,}650) = 6.22$, $p < .001$, and participants who were unsure whether they had seen the video before (vs. not seen), $b = -0.07$, $SE = 0.02$, $t(18{,}650) = 3.88$, $p < .001$. Technique discernment of the scapegoating content was significantly lower among participants who had seen the video before (vs. not seen), $b = -0.10$, $SE = 0.01$, $t(19{,}380) = 7.68$, $p < .001$, and participants who were unsure whether they had seen the video before (vs. not seen), $b = -0.05$, $SE = 0.01$, $t(19{,}390) = 4.86$, $p < .001$.

Willingness to share

Willingness to share the manipulative scapegoating content was significantly higher among participants who had seen the video before (vs. not seen), $b = 0.96$, $SE = 0.09$, $t(18{,}280) = 10.44$, $p < .001$, and participants who were unsure whether they had seen the video before (vs. not seen), $b = 0.74$, $SE = 0.08$, $t(18{,}280) = 9.40$, $p < .001$. Willingness to share the non-manipulative scapegoating content was significantly higher among participants who had seen the video before (vs. not seen), $b = 1.10$, $SE = 0.09$, $t(18{,}650) = 12.18$, $p < .001$, and participants who were unsure whether they had seen the video before (vs. not seen), $b = 0.80$, $SE = 0.08$, $t(18{,}650) = 10.32$, $p < .001$.

Decontextualization content

Manipulativeness assessments

Manipulativeness assessments of the non-manipulative decontextualization content were significantly higher among participants who had seen the video before (vs. not seen), $b = 0.28$, $SE = 0.08$, $t(18{,}730) = 3.45$, $p < .001$, and unsure whether they had seen the video before (vs. not seen), $b = 0.28$, $SE = 0.07$, $t(18{,}730) = 4.06$, $p < .001$. Manipulation discernment of the decontextualization content was significantly lower among participants who had seen the video before (vs. not seen), $b = -0.23$, $SE = 0.11$, $t(17{,}590) = 2.17$, $p = .030$, and unsure whether they had seen the video before (vs. not seen), $b = -0.29$, $SE = 0.09$, $t(17{,}580) = 3.25$, $p = .001$.

Technique recognition

Technique recognition of the manipulative decontextualization content was significantly lower among participants who were unsure whether they had seen the video before (vs. not seen), $b = -0.04$, $SE = 0.02$, $t(18{,}720) = 2.47$, $p = .014$. Technique recognition of the non-manipulative decontextualization content was significantly lower among participants who had seen the video before (vs. not seen), $b = -0.12$, $SE = 0.02$, $t(18{,}730) = 6.22$, $p < .001$, and participants who were unsure whether they had seen the video before (vs. not seen), $b = -0.05$, $SE = 0.02$, $t(18{,}730) = 3.15$, $p = .002$. Technique discernment of the decontextualization content was significantly lower among participants who had seen the video before (vs. not seen), $b = -0.05$, $SE = 0.01$, $t(19{,}450) = 4.15$, $p < .001$, and participants who were unsure whether they had seen the video before (vs. not seen), $b = -0.04$, $SE = 0.01$, $t(19{,}450) = 4.14$, $p < .001$.

Willingness to share

Willingness to share the manipulative decontextualization content was significantly higher among participants who had seen the video before (vs. not seen), $b = 0.92$, $SE = 0.09$,

$t(18{,}310) = 10.28$, $p < .001$, and participants who were unsure whether they had seen the video before (vs. not seen), $b = 0.72$, $SE = 0.08$, $t(18{,}310) = 9.42$, $p < .001$. Willingness to share the non-manipulative decontextualization content was significantly higher among participants who had seen the video before (vs. not seen), $b = 0.92$, $SE = 0.09$, $t(18{,}720) = 10.41$, $p < .001$, and participants who were unsure whether they had seen the video before (vs. not seen), $b = 0.64$, $SE = 0.07$, $t(18{,}730) = 8.57$, $p < .001$.

Discrediting content

Manipulativeness assessments

Manipulativeness assessments of the manipulative discrediting content were significantly lower among participants who were unsure whether they had seen the video before (vs. not seen), $b = -0.21$, $SE = 0.09$, $t(18{,}280) = 2.48$, $p = .013$. Manipulativeness assessments of the non-manipulative discrediting content were significantly higher among participants who had seen the video before (vs. not seen), $b = 0.45$, $SE = 0.08$, $t(18{,}650) = 5.55$, $p < .001$, and unsure whether they had seen the video before (vs. not seen), $b = 0.27$, $SE = 0.07$, $t(18{,}650) = 3.91$, $p < .001$. Manipulation discernment of the discrediting content was significantly lower among participants who had seen the video before (vs. not seen), $b = -0.42$, $SE = 0.13$, $t(17{,}500) = 3.09$, $p = .002$, and unsure whether they had seen the video before (vs. not seen), $b = -0.48$, $SE = 0.12$, $t(17{,}510) = 4.15$, $p < .001$.

Technique recognition

Technique recognition of the manipulative discrediting content was significantly lower among participants who had seen the video before (vs. not seen), $b = -0.04$, $SE = 0.02$, $t(18{,}660) = 2.03$, $p = .043$, and were unsure whether they had seen the video before (vs. not seen), $b = -0.06$, $SE = 0.02$, $t(18{,}670) = 3.57$, $p < .001$. Technique recognition of the non-manipulative discrediting content was significantly lower among participants who had seen the video before (vs. not seen), $b = -0.16$, $SE = 0.04$, $t(17{,}890) = 3.78$, $p < .001$, and

participants who were unsure whether they had seen the video before (vs. not seen), b = -0.08, SE = 0.04, $t(17{,}890) = 2.38$, $p = .017$. Technique discernment of the discrediting content was significantly lower among participants who had seen the video before (vs. not seen), b = -0.07, SE = 0.01, $t(19{,}420) = 5.33$, $p < .001$, and participants who were unsure whether they had seen the video before (vs. not seen), b = -0.06, SE = 0.01, $t(19{,}430) = 5.18$, $p < .001$.

Willingness to share

Willingness to share the manipulative discrediting content was significantly higher among participants who had seen the video before (vs. not seen), b = 0.96, SE = 0.09, $t(18{,}270) = 10.80$, $p < .001$, and participants who were unsure whether they had seen the video before (vs. not seen), b = 0.70, SE = 0.08, $t(18{,}280) = 9.26$, $p < .001$. Willingness to share the non-manipulative discrediting content was significantly higher among participants who had seen the video before (vs. not seen), b = 1.05, SE = 0.09, $t(18{,}650) = 11.86$, $p < .001$, and participants who were unsure whether they had seen the video before (vs. not seen), b = 0.60, SE = 0.08, $t(18{,}650) = 7.96$, $p < .001$.

Confidence in detecting manipulation

Confidence in detecting manipulation was significantly higher among participants who had seen the videos before (vs. not seen), b = 0.46, SE = 0.06, $t(16{,}150) = 7.42$, $p < .001$, and participants who had seen the videos before (vs. unsure), b = 0.41, SE = 0.08, $t(16{,}140) = 5.22$, $p < .001$.

Table S1

Means, Standard Deviations, and Sample Size Ns between Conditions for the Scapegoating Content.

	Control			Scapegoating						Decontextualization						Discrediting					
				Long			Short			Long			Short			Long			Short		
Dependent variable	*N*	*M*	*SD*	*N*	*M*	*SD*	*N*	*M*	*SD*	*N*	*M*	*SD*	*N*	*M*	*SD*	*N*	*M*	*SD*	*N*	*M*	*SD*
Manipulation discernment	3,778	0.64	2.08	3,919	0.81	2.12	1,219	0.82	1.99	3,313	0.86	2.29	1,020	0.93	2.19	3,287	0.82	2.37	1,015	0.88	2.37
Assessments (manipulative)	3,832	4.50	1.71	3,943	4.69	1.63	1,221	4.54	1.60	3,570	4.64	1.87	1,092	4.59	1.82	3,539	4.60	1.87	1,092	4.47	1.86
Assessments (non-manipulative)	3,898	3.85	1.50	3,954	3.88	1.40	1,229	3.72	1.39	3,659	3.76	1.65	1,138	3.68	1.62	3,652	3.75	1.70	1,129	3.60	1.63
Technique discernment	3,952	0.47	0.25	3,978	0.49	0.24	1,231	0.51	0.22	3,916	0.46	0.29	1,210	0.51	0.28	3,904	0.48	0.29	1,206	0.53	0.28
Technique (manipulative)	3,873	0.45	0.36	3,961	0.58	0.33	1,227	0.59	0.31	3,681	0.51	0.41	1,124	0.59	0.39	3,656	0.52	0.41	1,133	0.58	0.40
Technique (non-manipulative)	3,898	0.48	0.38	3,954	0.41	0.34	1,229	0.43	0.33	3,659	0.42	0.41	1,138	0.45	0.41	3,652	0.45	0.41	1,129	0.49	0.41
Sharing discernment	3,778	0.23	1.31	3,919	0.33	1.29	1,219	0.37	1.21	3,313	0.23	1.37	1,020	0.26	1.40	3,287	0.32	1.44	1,015	0.22	1.35
Sharing (manipulative)	3,832	2.76	1.79	3,943	2.88	1.76	1,221	2.59	1.66	3,570	2.68	1.85	1,092	2.50	1.73	3,539	2.71	1.86	1,092	2.52	1.78
Sharing (non-manipulative)	3,898	3.00	1.79	3,954	3.21	1.71	1,229	2.96	1.71	3,659	2.91	1.84	1,138	2.78	1.81	3,652	3.02	1.86	1,129	2.75	1.77
Confidence	3,892	4.58	1.23	3,898	2.94	2.01	1,206	3.35	1.94	-	-	-	-	-	-	-	-	-	-	-	-

Table S2

Means, Standard Deviations, and Sample Size Ns between Conditions for the Decontextualization Content.

	Control			Scapegoating						Decontextualization						Discrediting					
	-			Long			Short			Long			Short			Long			Short		
Dependent variable	*N*	*M*	*SD*	*N*	*M*	*SD*	*N*	*M*	*SD*	*N*	*M*	*SD*	*N*	*M*	*SD*	*N*	*M*	*SD*	*N*	*M*	*SD*
Manipulation discernment	3,780	0.62	1.91	3,321	0.69	2.15	1,031	0.67	2.12	3,922	0.82	1.79	1,211	0.73	1.74	3,311	0.67	2.26	1,022	0.71	2.15
Assessments (manipulative)	3,849	4.45	1.65	3,558	4.55	1.82	1,107	4.34	1.87	3,949	4.64	1.55	1,219	4.46	1.56	3,552	4.49	1.86	1,085	4.34	1.83
Assessments (non-manipulative)	3,895	3.83	1.51	3,689	3.83	1.69	1,137	3.69	1.74	3,977	3.82	1.37	1,227	3.73	1.35	3,670	3.80	1.69	1,143	3.63	1.70
Technique discernment	3,967	0.37	0.23	3,926	0.36	0.27	1,213	0.38	0.26	4,004	0.39	0.21	1,235	0.40	0.21	3,911	0.37	0.27	1,206	0.40	0.27
Technique (manipulative)	3,891	0.31	0.32	3,691	0.38	0.39	1,146	0.34	0.38	3,967	0.42	0.33	1,228	0.38	0.33	3,682	0.36	0.39	1,130	0.33	0.38
Technique (non-manipulative)	3,895	0.43	0.37	3,689	0.35	0.39	1,137	0.42	0.40	3,977	0.36	0.32	1,227	0.41	0.32	3,670	0.37	0.40	1,143	0.46	0.41
Sharing discernment	3,780	0.22	1.22	3,321	0.23	1.31	1,031	0.18	1.29	3,922	0.43	1.23	1,211	0.37	1.20	3,311	0.30	1.46	1,022	0.23	1.28
Sharing (manipulative)	3,849	2.72	1.73	3,558	2.77	1.84	1,107	2.55	1.81	3,949	2.65	1.66	1,219	2.57	1.60	3,552	2.67	1.81	1,085	2.48	1.73
Sharing (non-manipulative)	3,895	2.94	1.74	3,689	2.98	1.83	1,137	2.74	1.82	3,977	3.08	1.66	1,227	2.94	1.64	3,670	2.96	1.81	1,143	2.72	1.78
Confidence	3,892	4.58	1.23	-	-	-	-	-	-	3,890	4.48	1.24	1,203	4.41	1.18	-	-	-	-	-	-

Table S3

Means, Standard Deviations, and Sample Size Ns between Conditions for the Discrediting Content.

	Control			Scapegoating						Decontextualization						Discrediting					
	-			Long			Short			Long			Short			Long			Short		
Dependent variable	*N*	*M*	*SD*	*N*	*M*	*SD*	*N*	*M*	*SD*	*N*	*M*	*SD*	*N*	*M*	*SD*	*N*	*M*	*SD*	*N*	*M*	*SD*
Manipulation discernment	3,760	1.03	2.47	3,307	1.12	2.63	1,030	1.16	2.55	3,267	1.26	2.59	1,018	1.30	2.54	3,924	1.33	2.52	1,205	1.37	2.37
Assessments (manipulative)	3,824	4.66	1.89	3,565	4.75	2.01	1,101	4.65	2.01	3,533	4.87	1.95	1,099	4.74	1.96	3,950	4.91	1.84	1,215	4.87	1.75
Assessments (non-manipulative)	3,889	3.63	1.52	3,665	3.61	1.68	1,140	3.51	1.66	3,654	3.61	1.73	1,127	3.47	1.66	3,964	3.59	1.47	1,220	3.50	1.39
Technique discernment	3,954	0.49	0.25	3,923	0.48	0.29	1,211	0.52	0.28	3,921	0.49	0.29	1,208	0.53	0.28	3,991	0.50	0.24	1,230	0.52	0.22
Technique (manipulative)	3,870	0.52	0.37	3,671	0.57	0.41	1,142	0.64	0.40	3,665	0.59	0.41	1,145	0.64	0.40	3,965	0.60	0.33	1,219	0.61	0.33
Technique (non-manipulative)	3,805	0.65	0.88	3,413	0.53	0.75	1,071	0.58	0.77	3,398	0.55	0.76	1,064	0.58	0.77	3,938	0.56	0.74	1,209	0.60	0.77
Sharing discernment	3,760	0.40	1.31	3,307	0.40	1.40	1,030	0.38	1.34	3,267	0.40	1.44	1,018	0.39	1.38	3,924	0.64	1.44	1,205	0.53	1.41
Sharing (manipulative)	3,824	2.46	1.73	3,565	2.55	1.82	1,101	2.33	1.77	3,533	2.45	1.78	1,099	2.28	1.69	3,950	2.47	1.70	1,215	2.33	1.63
Sharing (non-manipulative)	3,889	2.86	1.74	3,665	2.97	1.84	1,140	2.67	1.79	3,654	2.84	1.82	1,127	2.65	1.72	3,964	3.11	1.66	1,220	2.86	1.61
Confidence	3,892	4.58	1.23	-	-	-	-	-	-	-	-	-	-	-	-	3,892	4.31	1.61	1,203	3.40	1.96

Table S4

Pearson's r Bivariate Correlation Coefficients between the Main Variables.

	decon_sr	scap_sr	dis_sr	scap_sf	decon_sf	dis_sf	decon_r	scap_r	dis_r	dir_exp	s_exp	sr_exp	de_exp	der_exp	decon_sdec	scap_sdec	dis_sdec	sf_exp	poltol	mocri	di_exp	dif_exp	decon_disc	decon_f	dis_disc	dis_f	scap_disc	scap_f	conf_all	digilit	def_exp	Education	vote_num	Age_1	male_num	Pol	indust	rich	west	edu_ind	demo
decon_sr	1.00	0.74	0.74	0.69	0.73	0.65	0.17	0.20	0.24	-0.07	-0.17	-0.13	-0.14	-0.09	0.38	0.08	0.11	-0.10	-0.16	-0.15	-0.18	-0.14	-0.16	-0.03	-0.25	-0.13	-0.20	-0.07	0.04	0.01	-0.09	-0.04	0.00	-0.06	0.00	0.03	0.00	-0.06	0.14	-0.19	-0.17
scap_sr	0.74	1.00	0.75	0.72	0.69	0.66	0.17	0.20	0.24	-0.07	-0.15	-0.10	-0.16	-0.12	0.08	0.37	0.12	-0.11	-0.16	-0.14	-0.17	-0.14	-0.16	-0.04	-0.24	-0.12	-0.20	-0.07	0.05	0.01	-0.08	-0.03	0.00	-0.05	0.02	0.01	0.00	-0.06	0.13	-0.18	-0.16
dis_sr	0.74	0.75	1.00	0.67	0.69	0.68	0.16	0.21	0.25	-0.05	-0.16	-0.12	-0.15	-0.13	0.08	0.12	0.41	-0.10	-0.16	-0.13	-0.13	-0.13	-0.15	-0.03	-0.24	-0.11	-0.19	-0.06	0.07	0.01	-0.07	-0.02	0.00	-0.05	0.04	0.02	-0.01	-0.07	0.14	-0.19	-0.17
scap_sf	0.69	0.72	0.67	1.00	0.72	0.75	0.19	0.23	0.27	-0.07	-0.23	-0.16	-0.19	-0.14	-0.04	-0.37	-0.09	-0.17	-0.21	-0.20	-0.22	-0.19	-0.21	-0.07	-0.29	-0.16	-0.23	-0.08	0.02	-0.04	-0.12	-0.06	-0.02	-0.05	0.00	0.05	0.00	-0.07	0.15	-0.19	-0.17
decon_sf	0.73	0.69	0.69	0.72	1.00	0.75	0.20	0.22	0.27	-0.08	-0.20	-0.15	-0.20	-0.13	-0.36	-0.04	-0.08	-0.13	-0.20	-0.19	-0.21	-0.18	-0.21	-0.06	-0.29	-0.16	-0.24	-0.10	0.02	-0.03	-0.14	-0.06	-0.02	-0.05	0.01	0.04	0.00	-0.05	0.13	-0.16	-0.14
dis_sf	0.65	0.66	0.68	0.75	0.75	1.00	0.20	0.24	0.28	-0.08	-0.22	-0.16	-0.18	-0.14	-0.12	-0.12	-0.39	-0.15	-0.22	-0.21	-0.25	-0.21	-0.22	-0.07	-0.30	-0.16	-0.26	-0.11	0.01	-0.05	-0.10	-0.05	-0.04	-0.05	0.01	0.05	0.01	-0.05	0.14	-0.16	-0.14
decon_r	0.17	0.17	0.16	0.19	0.20	0.20	1.00	0.38	0.37	-0.06	-0.11	-0.14	-0.18	-0.25	-0.04	-0.04	-0.04	-0.02	-0.07	-0.11	-0.13	-0.06	-0.56	0.26	-0.17	0.08	-0.14	0.15	0.01	0.00	0.01	-0.01	-0.01	0.01	-0.01	0.02	0.01	-0.05	0.05	-0.07	-0.06
scap_r	0.20	0.20	0.21	0.23	0.22	0.24	0.38	1.00	0.43	-0.08	-0.21	-0.24	-0.13	-0.13	-0.02	-0.04	-0.04	-0.06	-0.11	-0.16	-0.15	-0.09	-0.16	0.16	-0.26	0.01	-0.60	0.13	0.01	-0.01	-0.04	-0.03	-0.02	0.03	-0.01	0.05	0.00	-0.03	0.07	-0.09	-0.07
dis_r	0.24	0.24	0.25	0.27	0.27	0.28	0.37	0.43	1.00	-0.12	-0.16	-0.16	-0.14	-0.14	-0.04	-0.05	-0.04	-0.07	-0.13	-0.18	-0.23	-0.12	-0.20	0.11	-0.65	-0.03	-0.27	0.05	0.00	-0.03	-0.05	-0.05	-0.03	0.02	0.01	0.05	0.01	-0.03	0.05	-0.06	-0.05
dir_exp	-0.07	-0.07	-0.05	-0.07	-0.08	-0.08	-0.06	-0.08	-0.12	1.00	0.18	0.28	0.16	0.25	0.01	0.01	0.05	-0.03	0.05	0.09	0.43	-0.02	0.04	-0.01	0.10	0.03	0.07	0.01	0.01	0.02	-0.03	0.02	-0.01	-0.04	-0.03	-0.03	-0.01	0.02	-0.04	0.07	0.07
s_exp	-0.17	-0.15	-0.16	-0.23	-0.20	-0.22	-0.11	-0.21	-0.16	0.18	1.00	0.66	0.24	0.24	0.04	0.10	0.07	0.64	0.19	0.23	0.32	0.19	0.14	0.06	0.21	0.14	0.24	0.12	0.07	0.09	0.08	0.07	0.03	0.00	-0.01	-0.07	0.02	-0.04	0.04	0.03	-0.01
sr_exp	-0.13	-0.10	-0.12	-0.16	-0.15	-0.16	-0.14	-0.24	-0.16	0.28	0.66	1.00	0.27	0.37	0.02	0.08	0.04	-0.04	0.10	0.18	0.30	0.02	0.09	-0.03	0.13	0.04	0.18	0.01	0.04	0.04	-0.02	0.04	-0.01	-0.03	-0.03	-0.05	0.01	0.03	-0.05	0.09	0.09
de_exp	-0.14	-0.16	-0.15	-0.19	-0.20	-0.18	-0.18	-0.13	-0.14	0.16	0.24	0.27	1.00	0.63	0.08	0.04	0.04	0.07	0.15	0.20	0.24	0.10	0.20	0.09	0.17	0.11	0.16	0.08	0.07	0.09	0.60	0.10	0.03	-0.03	0.02	-0.06	0.00	0.02	-0.10	0.11	0.10
der_exp	-0.09	-0.12	-0.13	-0.14	-0.13	-0.14	-0.25	-0.13	-0.14	0.25	0.24	0.37	0.63	1.00	0.05	0.02	0.01	-0.04	0.09	0.14	0.26	-0.01	0.16	-0.05	0.09	0.01	0.09	-0.01	0.01	0.02	-0.11	0.00	-0.03	-0.02	-0.02	-0.03	-0.03	0.09	-0.06	0.13	0.12
decon_sdec	0.38	0.08	0.08	-0.04	-0.36	-0.12	-0.04	-0.02	-0.04	0.01	0.04	0.02	0.08	0.05	1.00	0.16	0.25	0.04	0.06	0.05	0.04	0.05	0.07	0.05	0.06	0.05	0.05	0.05	0.03	0.05	0.07	0.02	0.03	-0.01	-0.01	-0.02	0.00	-0.01	0.01	-0.04	-0.04
scap_sdec	0.08	0.37	0.12	-0.37	-0.04	-0.12	-0.04	-0.04	-0.05	0.01	0.10	0.08	0.04	0.02	0.16	1.00	0.29	0.08	0.07	0.08	0.06	0.06	0.06	0.04	0.07	0.06	0.05	0.02	0.04	0.05	0.05	0.04	0.02	0.01	0.03	-0.07	0.00	0.01	-0.02	0.00	0.00
dis_sdec	0.11	0.12	0.41	-0.09	-0.08	-0.39	-0.04	-0.04	-0.04	0.05	0.07	0.04	0.04	0.01	0.25	0.29	1.00	0.06	0.08	0.10	0.13	0.09	0.08	0.06	0.07	0.05	0.08	0.06	0.07	0.09	0.04	0.05	0.04	0.00	0.03	-0.04	-0.01	-0.02	0.01	-0.04	-0.04
sf_exp	-0.10	-0.11	-0.10	-0.17	-0.13	-0.15	-0.02	-0.06	-0.07	-0.03	0.64	-0.04	0.07	-0.04	0.04	0.08	0.06	1.00	0.18	0.15	0.14	0.26	0.12	0.12	0.17	0.16	0.17	0.16	0.06	0.09	0.14	0.06	0.06	0.04	0.03	-0.05	0.01	-0.09	0.10	-0.04	-0.11
poltol	-0.16	-0.16	-0.16	-0.21	-0.20	-0.22	-0.07	-0.11	-0.13	0.05	0.19	0.10	0.15	0.09	0.06	0.07	0.08	0.18	1.00	0.18	0.18	0.19	0.14	0.09	0.20	0.16	0.16	0.11	0.06	0.15	0.10	0.06	0.04	0.05	0.04	-0.07	-0.01	0.00	0.03	-0.05	-0.07
mocri	-0.15	-0.14	-0.13	-0.20	-0.19	-0.21	-0.11	-0.16	-0.18	0.09	0.23	0.18	0.20	0.14	0.05	0.08	0.10	0.15	0.18	1.00	0.23	0.18	0.16	0.09	0.25	0.18	0.21	0.13	0.07	0.12	0.13	0.13	0.07	0.00	-0.01	-0.10	0.01	0.07	-0.12	0.15	0.15
di_exp	-0.18	-0.17	-0.13	-0.22	-0.21	-0.25	-0.13	-0.15	-0.23	0.43	0.32	0.30	0.24	0.26	0.04	0.06	0.13	0.14	0.18	0.23	1.00	0.65	0.14	0.05	0.27	0.16	0.17	0.08	0.05	0.08	0.06	0.06	0.05	-0.01	0.00	-0.05	0.01	0.04	-0.07	0.12	0.10
dif_exp	-0.14	-0.14	-0.13	-0.19	-0.18	-0.21	-0.06	-0.09	-0.12	-0.02	0.19	0.02	0.10	-0.01	0.05	0.06	0.09	0.26	0.19	0.18	0.65	1.00	0.13	0.10	0.22	0.20	0.15	0.11	0.05	0.09	0.14	0.06	0.08	0.04	0.03	-0.04	0.02	0.02	-0.03	0.06	0.03
decon_disc	-0.16	-0.16	-0.15	-0.21	-0.21	-0.22	-0.56	-0.16	-0.20	0.04	0.14	0.09	0.20	0.16	0.07	0.06	0.08	0.12	0.14	0.16	0.14	0.13	1.00	0.65	0.38	0.34	0.29	0.23	0.07	0.09	0.14	0.07	0.02	0.02	0.04	-0.03	0.00	0.01	-0.04	0.02	0.02
decon_f	-0.03	-0.04	-0.03	-0.07	-0.06	-0.07	0.26	0.16	0.11	-0.01	0.06	-0.03	0.09	-0.05	0.05	0.04	0.06	0.12	0.09	0.09	0.05	0.10	0.65	1.00	0.29	0.48	0.22	0.42	0.09	0.11	0.17	0.07	0.02	0.03	0.03	-0.02	0.02	-0.03	-0.01	-0.04	-0.03
dis_disc	-0.25	-0.24	-0.24	-0.29	-0.29	-0.30	-0.17	-0.26	-0.65	0.10	0.21	0.13	0.17	0.09	0.06	0.07	0.07	0.17	0.20	0.25	0.27	0.22	0.38	0.29	1.00	0.78	0.46	0.35	0.08	0.12	0.14	0.10	0.04	-0.01	0.01	-0.06	-0.02	0.00	-0.05	0.05	0.04
dis_f	-0.13	-0.12	-0.11	-0.16	-0.16	-0.16	0.08	0.01	-0.03	0.03	0.14	0.04	0.11	0.01	0.05	0.06	0.05	0.16	0.16	0.18	0.16	0.20	0.34	0.48	0.78	1.00	0.40	0.51	0.12	0.13	0.14	0.09	0.03	0.01	0.02	-0.03	-0.02	-0.02	-0.03	0.01	0.01
scap_disc	-0.20	-0.20	-0.19	-0.23	-0.24	-0.26	-0.14	-0.60	-0.27	0.07	0.24	0.18	0.16	0.09	0.05	0.05	0.08	0.17	0.16	0.21	0.17	0.15	0.29	0.22	0.46	0.40	1.00	0.71	0.06	0.11	0.12	0.08	0.04	-0.02	0.02	-0.07	0.00	0.02	-0.07	0.07	0.06
scap_f	-0.07	-0.07	-0.06	-0.08	-0.10	-0.11	0.15	0.13	0.05	0.01	0.12	0.01	0.08	-0.01	0.05	0.02	0.06	0.16	0.11	0.13	0.08	0.11	0.23	0.42	0.35	0.51	0.71	1.00	0.09	0.12	0.11	0.07	0.03	0.00	0.02	-0.04	0.00	0.00	-0.03	0.00	0.01
conf_all	0.04	0.05	0.07	0.02	0.02	0.01	0.01	0.01	0.00	0.01	0.07	0.04	0.07	0.01	0.03	0.04	0.07	0.06	0.06	0.07	0.05	0.05	0.07	0.09	0.08	0.12	0.06	0.09	1.00	0.27	0.08	0.11	0.05	-0.05	0.10	0.02	0.03	-0.02	0.02	-0.07	-0.04
digilit	0.01	0.01	0.01	-0.04	-0.03	-0.05	0.00	-0.01	-0.03	0.02	0.09	0.04	0.09	0.02	0.05	0.05	0.09	0.09	0.15	0.12	0.08	0.09	0.09	0.11	0.12	0.13	0.11	0.12	0.27	1.00	0.11	0.19	0.07	-0.20	0.09	-0.01	-0.02	-0.05	0.07	-0.08	-0.07
def_exp	-0.09	-0.08	-0.07	-0.12	-0.14	-0.10	0.01	-0.04	-0.05	-0.03	0.08	-0.02	0.60	-0.11	0.07	0.05	0.04	0.14	0.10	0.13	0.06	0.14	0.14	0.17	0.14	0.14	0.12	0.11	0.08	0.11	1.00	0.14	0.06	-0.03	0.04	-0.05	0.03	-0.06	-0.07	0.02	0.02
Education	-0.04	-0.03	-0.02	-0.06	-0.06	-0.05	-0.01	-0.03	-0.05	0.02	0.07	0.04	0.10	0.00	0.02	0.04	0.05	0.06	0.06	0.13	0.06	0.06	0.07	0.07	0.10	0.09	0.08	0.07	0.11	0.19	0.14	1.00	0.09	-0.08	0.00	-0.03	0.03	-0.08	-0.02	-0.09	-0.03
vote_num	0.00	0.00	0.00	-0.02	-0.02	-0.04	-0.01	-0.02	-0.03	-0.01	0.03	-0.01	0.03	-0.03	0.03	0.02	0.04	0.06	0.04	0.07	0.05	0.08	0.02	0.02	0.04	0.03	0.04	0.03	0.05	0.07	0.06	0.09	1.00	0.09	0.04	0.00	0.01	-0.04	-0.05	0.07	0.05
Age_1	-0.06	-0.05	-0.05	-0.05	-0.05	-0.05	0.01	0.03	0.02	-0.04	0.00	-0.03	-0.03	-0.02	-0.01	0.01	0.00	0.04	0.05	0.00	-0.01	0.04	0.02	0.03	-0.01	0.01	-0.02	0.00	-0.05	-0.20	-0.03	-0.08	0.09	1.00	0.12	-0.03	0.01	0.03	-0.08	0.12	0.11
male_num	0.00	0.02	0.04	0.00	0.01	0.01	-0.01	-0.01	0.01	-0.03	-0.01	-0.03	0.02	-0.02	-0.01	0.03	0.03	0.03	0.04	-0.01	0.00	0.03	0.04	0.03	0.01	0.02	0.02	0.02	0.10	0.09	0.04	0.00	0.04	0.12	1.00	0.03	0.00	0.00	0.00	0.01	0.00
Pol	0.03	0.01	0.02	0.05	0.04	0.05	0.02	0.05	0.05	-0.03	-0.07	-0.05	-0.06	-0.03	-0.02	-0.07	-0.04	-0.05	-0.07	-0.10	-0.05	-0.04	-0.03	-0.02	-0.06	-0.03	-0.07	-0.04	0.02	-0.01	-0.05	-0.03	0.00	-0.03	0.03	1.00	-0.01	-0.02	0.03	-0.01	-0.01
indust	0.00	0.00	-0.01	0.00	0.00	0.01	0.01	0.00	0.01	-0.01	0.02	0.01	0.00	-0.03	0.00	0.00	-0.01	0.01	-0.01	0.01	0.01	0.02	0.00	0.02	-0.02	-0.02	0.00	0.00	0.03	-0.02	0.03	0.03	0.01	0.01	0.00	-0.01	1.00	0.26	-0.32	0.13	0.21
rich	-0.06	-0.06	-0.07	-0.07	-0.05	-0.05	-0.05	-0.03	-0.03	0.02	-0.04	0.03	0.02	0.09	-0.01	0.01	-0.02	-0.09	0.00	0.07	0.04	0.02	0.01	-0.03	0.00	-0.02	0.02	0.00	-0.02	-0.05	-0.06	-0.08	-0.04	0.03	0.00	-0.02	0.26	1.00	-0.45	0.48	0.58
west	0.14	0.13	0.14	0.15	0.13	0.14	0.05	0.07	0.05	-0.04	0.04	-0.05	-0.10	-0.06	0.01	-0.02	0.01	0.10	0.03	-0.12	-0.07	-0.03	-0.04	-0.01	-0.05	-0.03	-0.07	-0.03	0.02	0.07	-0.07	-0.02	-0.05	-0.08	0.00	0.03	-0.32	-0.45	1.00	-0.73	-0.81
edu_ind	-0.19	-0.18	-0.19	-0.19	-0.16	-0.16	-0.07	-0.09	-0.06	0.07	0.03	0.09	0.11	0.13	-0.04	0.00	-0.04	-0.04	-0.05	0.15	0.12	0.06	0.02	-0.04	0.05	0.01	0.07	0.00	-0.07	-0.08	0.02	-0.09	0.07	0.12	0.01	-0.01	0.13	0.48	-0.73	1.00	0.94
demo	-0.17	-0.16	-0.17	-0.17	-0.14	-0.14	-0.06	-0.07	-0.05	0.07	-0.01	0.09	0.10	0.12	-0.04	0.00	-0.04	-0.11	-0.07	0.15	0.10	0.03	0.02	-0.03	0.04	0.01	0.06	0.01	-0.04	-0.07	0.02	-0.03	0.05	0.11	0.00	-0.01	0.21	0.58	-0.81	0.94	1.00

Note. scap_disc = Manipulation discernment for scapegoating content; scap_sf = Manipulativeness assessments of manipulative scapegoating content; scap_sr = Manipulativeness assessments of non-manipulative scapegoating content; decon_disc = Manipulation discernment for

decontextualization content; decon_sf = Manipulativeness assessments of manipulative decontextualization content; decon_sr = Manipulativeness assessments of non-manipulative decontextualization content; dis_disc = Manipulation discernment for discrediting content; dis_sf = Manipulativeness assessments of manipulative discrediting content; dis_sr = Manipulativeness assessments of non-manipulative discrediting content; s_exp = Technique discernment for scapegoating content; sf_exp = Technique recognition for manipulative scapegoating content; sr_exp = Technique recognition for non-manipulative scapegoating content; de_exp = Technique discernment for decontextualization content; def_exp = Technique recognition for manipulative decontextualization content; der_exp = Technique recognition for non-manipulative decontextualization content; di_exp = Technique discernment for discrediting content; dif_exp = Technique recognition for manipulative discrediting content; dir_exp = Technique recognition for non-manipulative discrediting content; scap_sdec = Sharing discernment for scapegoating content; scap_sf = Sharing intentions for manipulative scapegoating content; scap_sr = Sharing intentions for non-manipulative scapegoating content; decon_sdec = Sharing discernment for decontextualization content; decon_sf = Sharing intentions for manipulative decontextualization content; decon_sr = Sharing intentions for non-manipulative decontextualization content; dis_sdec = Sharing discernment for discrediting content; dis_sf = Sharing intentions for manipulative discrediting content; dis_sr = Sharing intentions for non-manipulative discrediting content; conf_all = Confidence in detecting manipulation; Education = Educational attainment; Age_1 = Age; Pol = Political ideology; mocri = General manipulation discernment ability; digilit = Digital literacy; poltol = Political tolerance; west = Lattitude; edu_ind = Education index; indust = Industrialization index; rich = GDP per capita; demo = Democratic index; male = Gender (1 = Male, 0 = Female); Vote = Voting behaviour (1 = Voted, 0 = Did not vote).

Section 6: Comparison of intervention effects by video length

Manipulativeness assessments

Scapegoating

There were no significant differences in manipulativeness assessments of the manipulative, b = -0.06, SE = 0.04, t(4132.99) = 1.69, p = .090, or non-manipulative scapegoating content by video length, b = -0.01, SE = 0.03, t(14,910) = 0.41, p = .684, nor in manipulation discernment of the scapegoating content, b = -0.02, SE = 0.05, t(11,880) = 0.45, p = .650.

Decontextualization

There were no significant differences in manipulativeness assessments of the manipulative, b = -0.05, SE = 0.04, t(9081.08) = 1.35, p = .177, or non-manipulative decontextualization content by video length, b = -0.01, SE = 0.03, t(9,407) = 0.20, p = .842, nor in manipulation discernment of the decontextualization content, b = -0.03, SE = 0.04, t(5646) = 0.69, p = .489.

Discrediting

There were no significant differences in manipulativeness assessments of the manipulative discrediting content by video length, b = -0.07, SE = 0.04, t(8,474.15) = 1.77, p = .077, or in manipulativeness assessments of the non-manipulative discrediting content, b = -0.04, SE = 0.04, t(8,794.32) = 1.06, p = .289, nor in manipulation discernment of the discrediting content, b = -0.03, SE = 0.06, t(9,849.58) = 0.48, p = .630.

Technique recognition

Scapegoating

There were no significant differences in technique recognition of the manipulative scapegoating content by video length, b = 0.01, SE = 0.01, t(18,554.99) = 0.99, p = .321, or in technique recognition of the non-manipulative scapegoating content, b = -0.01, SE = 0.01,

t(14,450.00) = 1.19, *p* = .234, nor in technique discernment of the scapegoating content, *b* = 0.02, *SE* = 0.01, *t*(17,530.00) = 1.36, *p* = .173.

Decontextualization

There were no significant differences in technique recognition of the manipulative decontextualization content by video length, *b* = 0.01, *SE* = 0.01, *t*(18,170.00) = 1.25, *p* = .210, or in technique recognition of the non-manipulative decontextualization content, *b* = -0.01, *SE* = 0.01, *t*(17,530.00) = 1.36, *p* = .175, nor in technique discernment of the decontextualization content, *b* = -0.01, *SE* = 0.01, *t*(17,530.00) = 1.36, *p* = .175.

Discrediting

There were no significant differences in technique recognition of the manipulative discrediting content by video length, *b* = 0.01, *SE* = 0.01, *t*(18,340.00) = 0.75, *p* = .455, or in technique recognition of the non-manipulative discrediting content, *b* = -0.002, *SE* = 0.02, *t*(10,460.00) = 0.09, *p* = .925, nor in technique discernment of the discrediting content, *b* = 0.01, *SE* = 0.02, *t*(12,390.00) = 0.69, *p* = .491.

Willingness to share

Scapegoating

There were no significant differences in willingness to share the manipulative scapegoating content by video length, *b* = 0.04, *SE* = 0.04, *t*(17,990.00) = 1.10, *p* = .271, or in willingness to share the non-manipulative scapegoating content, *b* = 0.02, *SE* = 0.04, *t*(18,350.00) = 0.63, *p* = .529, nor in sharing decisions for the scapegoating content, *b* = -0.02, *SE* = 0.03, *t*(4,961.07) = 0.68, *p* = .495.

Decontextualization

There was no significant difference in willingness to share the non-manipulative decontextualization content by video length, *b* = 0.03, *SE* = 0.04, *t*(18,460.00) = 0.76, *p* = .450. However, willingness to share the manipulative decontextualization content was

significantly higher for participants who watched short (vs. long) videos, *b* = 0.08, *SE* = 0.04, *t*(18,230.00) = 2.11, *p* = .035, and sharing decisions for the decontextualization content were significantly lower for participants who watched short (vs. long) videos, *b* = -0.06, *SE* = 0.03, *t*(6,301.47) = 2.09, *p* = .037.

Discrediting

There were no significant differences in willingness to share the manipulative discrediting content by video length, *b* = 0.07, *SE* = 0.04, *t*(18,290.00) = 1.86, *p* = .063, or in willingness to share the non-manipulative discrediting content, *b* = 0.03, *SE* = 0.04, *t*(18,400.00) = 0.67, *p* = .502, nor in sharing decisions for the discrediting content, *b* = -0.02, *SE* = 0.03, *t*(5,670.84) = 0.55, *p* = .584.

Confidence in detecting relevant manipulation

There were no significant differences in confidence in detecting relevant manipulation by video length, *b* = 0.01, *SE* = 0.03, *t*(14,970.00) = 0.17, *p* = .862.

Section 7: Exploratory multilevel models by individual item variation

To assess the variance accounted for by individual items in each model, we ran a series of exploratory multilevel models that were comparable to our main analyses. This time, we included a composite of the individual items from each respective analysis as the dependent variable and accounted for individual items as a random intercept. For the manipulation discernment and sharing discernment analyses, the non-manipulative items were reverse-coded so that higher scores indicated higher manipulativeness ratings and lower sharing intentions for manipulative content.

Manipulation discernment

Scapegoating

The long scapegoating condition (vs. control) had a positive significant effect on manipulation discernment of the scapegoating content, *b* = 0.07, *SE* = 0.02, *t*(104105) = 3.99,

$p < .001$, but the short scapegoating video (vs. control) had a non-significant effect, $b = 0.01$, $SE = 0.03$, $t(80250) = 0.30$, $p = .762$. Positive significant effects were also found for the long decontextualization, $b = 0.14$, $SE = 0.02$, $t(104105) = 6.44$, $p < .001$, and discrediting videos (vs. control), $b = 0.09$, $SE = 0.02$, $t(104105) = 4.44$, $p < .001$, but not the short decontextualization, $b = 0.05$, $SE = 0.03$, $t(93760) = 1.49$, $p = .137$, or discrediting videos (vs. control), $b = 0.04$, $SE = 0.03$, $t(93780) = 1.12$, $p = .265$. The conditions had a significant effect on all items, $\chi^2(6) = 52.05$, $p < .001$. The variance attributable to the item level was substantial ($\sigma^2 = 0.10$), indicating meaningful differences between items in average endorsement. However, residual variance remained dominant ($\sigma^2 = 4.10$), indicating that the majority of variance stemmed from individual differences.

Decontextualization

The long decontextualization condition (vs. control) had a positive significant effect on manipulation discernment of the decontextualization content, $b = 0.10$, $SE = 0.02$, $t(97730) = 5.83$, $p < .001$, but the short decontextualization video (vs. control) had a non-significant effect, $b = 0.02$, $SE = 0.03$, $t(29360) = 0.81$, $p = .418$. A positive significant effect was also found for the long discrediting video (vs. control), $b = 0.06$, $SE = 0.02$, $t(97730) = 3.01$, $p = .003$, and for the long scapegoating video, $b = 0.05$, $SE = 0.02$, $t(97730) = 2.38$, $p = .017$. However, the short discrediting video had no significant effect, $b = 0.003$, $SE = 0.03$, $t(52290) = 0.08$, $p = .938$, nor did the short scapegoating video, $b = -0.001$, $SE = 0.03$, $t(51980) = -0.03$, $p = .977$. The variance attributable to the item level was meaningful ($\sigma^2 = 0.16$), again indicating variability in item endorsement, while the dominant source of variance remained residual ($\sigma^2 = 4.02$), suggesting substantial individual-level differences in discernment.

Discrediting

The long discrediting condition (vs. control) significantly increased manipulation discernment of the discrediting content, $b = 0.15$, $SE = 0.02$, $t(97{,}630) = 7.96$, $p < .001$, and the short discrediting video also had a positive effect, $b = 0.11$, $SE = 0.03$, $t(79{,}050) = 3.90$, $p < .001$. The long decontextualization condition also showed a significant positive effect, $b = 0.14$, $SE = 0.02$, $t(97{,}630) = 6.10$, $p < .001$, as did the short decontextualization condition, $b = 0.08$, $SE = 0.04$, $t(89{,}810) = 2.20$, $p = .028$. The long scapegoating video also significantly increased discernment, $b = 0.06$, $SE = 0.02$, $t(97{,}630) = 2.83$, $p = .005$, but the short scapegoating video had no significant effect, $b = -0.02$, $SE = 0.04$, $t(89{,}730) = -0.47$, $p = .638$. The variance attributable to the item level was modest ($\sigma^2 = 0.08$), and the dominant variance component remained residual ($\sigma^2 = 4.32$), again indicating that most variance in scores was due to individual-level differences.

Technique discernment

Scapegoating

The long scapegoating condition significantly increased technique discernment of the scapegoating content, $b = 0.17$, $SE = 0.02$, $t(104{,}105) = 10.18$, $p < .001$. The short scapegoating video also produced a significant effect, $b = 0.15$, $SE = 0.03$, $t(104{,}105) = 5.89$, $p < .001$. By contrast, none of the discrediting or decontextualization videos significantly influenced scapegoating technique discernment: for example, the long decontextualization video had a small negative effect, $b = -0.07$, $SE = 0.02$, $t(104{,}105) = -3.46$, $p < .001$, while the short decontextualization ($b = -0.01$, $SE = 0.03$, $t(104{,}105) = -0.18$, $p = .861$), long discrediting ($b = -0.01$, $SE = 0.02$, $t(104{,}105) = -0.36$, $p = .722$), and short discrediting ($b = 0.03$, $SE = 0.03$, $t(104{,}105) = 0.94$, $p = .349$) conditions showed no significant effects. The variance attributable to items was modest ($\sigma^2 = 0.06$). Nonetheless, residual variance ($\sigma^2 = 3.77$) remained dominant, reflecting substantial individual-level variability.

Decontextualization

The short decontextualization video significantly increased technique discernment of the decontextualization content, $b = 0.15$, $SE = 0.03$, $t(97{,}600) = 5.76$, $p < .001$, as did the long decontextualization video, though with a smaller effect, $b = 0.04$, $SE = 0.02$, $t(97{,}730) = 2.26$, $p = .024$. In contrast, the discrediting videos showed no significant effects on decontextualization technique discernment ($b = -0.00$, $SE = 0.02$, $t(97{,}730) = -0.09$, $p = .926$ for the long video; $b = 0.01$, $SE = 0.03$, $t(97{,}700) = 0.28$, $p = .777$ for the short video). The long scapegoating video also had a modest significant effect, $b = 0.05$, $SE = 0.02$, $t(97{,}730) = 2.52$, $p = .012$, while the short scapegoating video did not, $b = 0.02$, $SE = 0.03$, $t(97{,}700) = 0.52$, $p = .601$. There was modest variance attributable to items ($\sigma^2 = 0.05$). However, residual variance remained dominant ($\sigma^2 = 3.65$), suggesting the bulk of variability in technique discernment was due to individual-level differences.

Discrediting

Both discrediting videos significantly increased technique discernment of the discrediting content. The long video had a large effect, $b = 0.14$, $SE = 0.02$, $t(97{,}630) = 8.10$, $p < .001$, and the short video had an even larger effect, $b = 0.19$, $SE = 0.03$, $t(97{,}540) = 7.53$, $p < .001$. The long and short scapegoating videos also significantly increased discrediting technique discernment ($b = 0.12$, $SE = 0.02$, $t(97{,}630) = 6.05$, $p < .001$; $b = 0.10$, $SE = 0.03$, $t(97{,}620) = 3.07$, $p = .002$, respectively). The short, $b = 0.05$, $SE = 0.03$, $t(97{,}620) = 1.68$, $p = .094$, and long decontextualization videos had no effect, $b = -0.01$, $SE = 0.02$, $t(97{,}630) = -0.69$, $p = .492$. Substantial variance was observed at the item level ($\sigma^2 = 0.10$). Residual variance remained highest ($\sigma^2 = 3.53$), indicating individual-level differences explained most of the variation in responses.

Sharing discernment

Scapegoating

Both scapegoating videos significantly improved sharing discernment of the scapegoating content. The long video had a large positive effect, $b = 0.13$, $SE = 0.003$, $t(310{,}500) = 49.42$, $p < .001$, as did the short video, $b = 0.13$, $SE = 0.004$, $t(310{,}500) = 34.00$, $p < .001$. In contrast, all other interventions—including the long and short decontextualization and discrediting videos—significantly reduced sharing discernment (*b*s = –0.12, all *SE*s ≈ 0.003–0.004, all *p*s < .001). Random effect variances by items was negligible ($\sigma^2 < 0.001$). The residual variance ($\sigma^2 = 0.21$) accounted for nearly all the variance, indicating that individual-level differences dominated responses.

Decontextualization

Both decontextualization videos significantly improved sharing discernment of the decontextualization content. The long video had a large positive effect, $b = 0.12$, $SE = 0.003$, $t(310{,}500) = 47.97$, $p < .001$, as did the short video, $b = 0.12$, $SE = 0.004$, $t(310{,}500) = 32.94$, $p < .001$. In contrast, all other videos—including the scapegoating and discrediting conditions—significantly reduced sharing discernment (*b*s = –0.12, all *SE*s ≈ 0.003–0.004, all *p*s < .001). Random effect variances for item was near zero, with nearly all variance captured at the residual level ($\sigma^2 = 0.21$).

Discrediting

The discrediting videos significantly improved sharing discernment of the discrediting content. The long video had a strong positive effect, $b = 0.13$, $SE = 0.003$, $t(310{,}500) = 48.79$, $p < .001$, as did the short video, $b = 0.13$, $SE = 0.004$, $t(310{,}500) = 33.19$, $p < .001$. In contrast, all other videos—including those about decontextualization and scapegoating—significantly reduced sharing discernment (all *b*s ≈ –0.12, all *SE*s ≈ 0.003–0.004, all *p*s <

.001). Random intercept variance for item was negligible ($\sigma^2 \approx 0$), with nearly all variance captured by the residual term ($\sigma^2 = 0.21$).

Section 8: Violin Plots for Means between Conditions

Manipulativeness assessments

Manipulative scapegoating content

Figure S89

Violin Plot for the Mean Manipulativeness Assessments of Manipulative Scapegoating Content between Conditions.

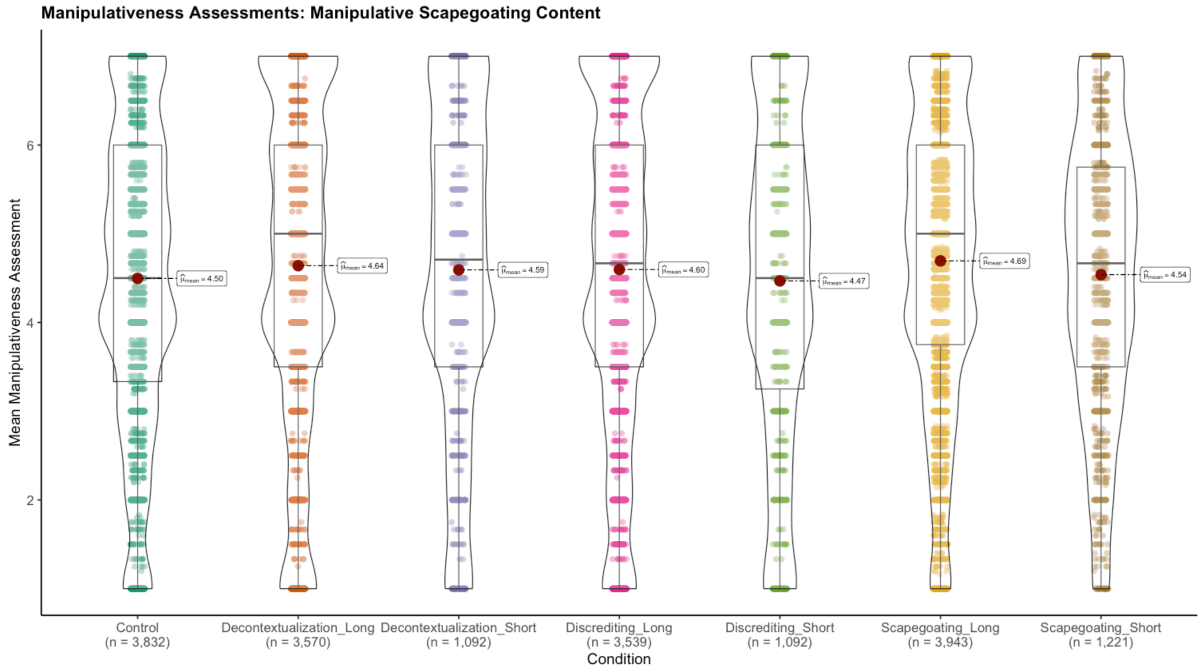

Non-Manipulative scapegoating content

Figure S90

Violin Plot for the Mean Manipulativeness Assessments of Non-Manipulative Scapegoating Content between Conditions.

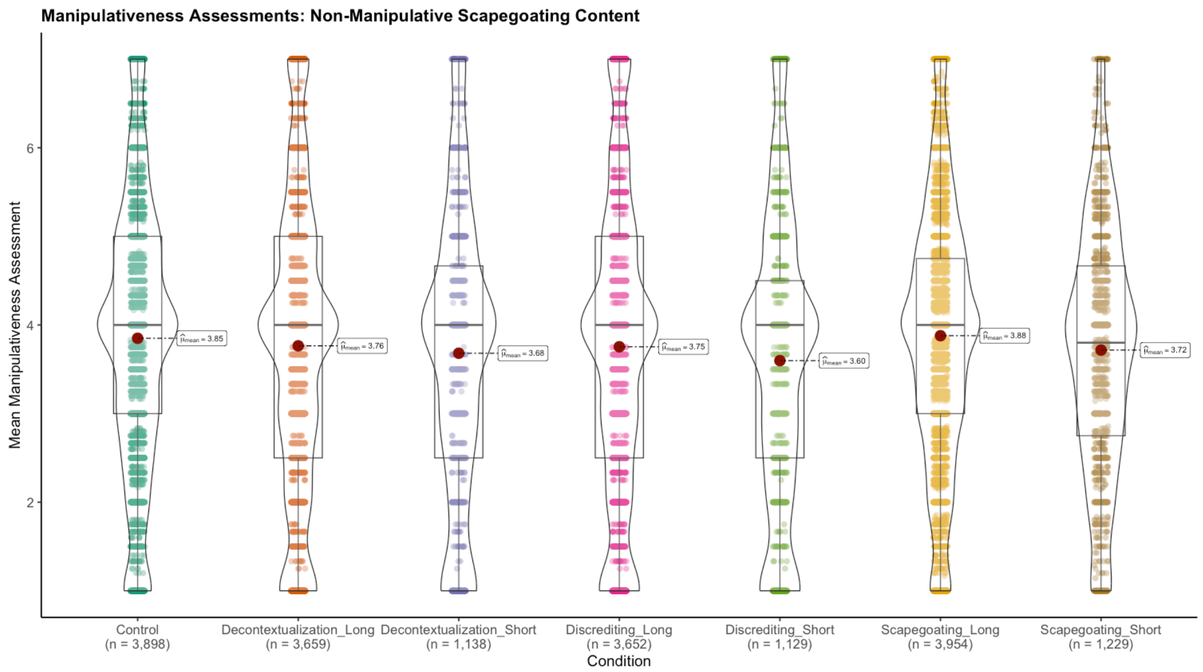

Manipulation discernment of Scapegoating content

Figure S91

Violin Plot for the Mean Manipulation Discernment of Scapegoating Content between Conditions.

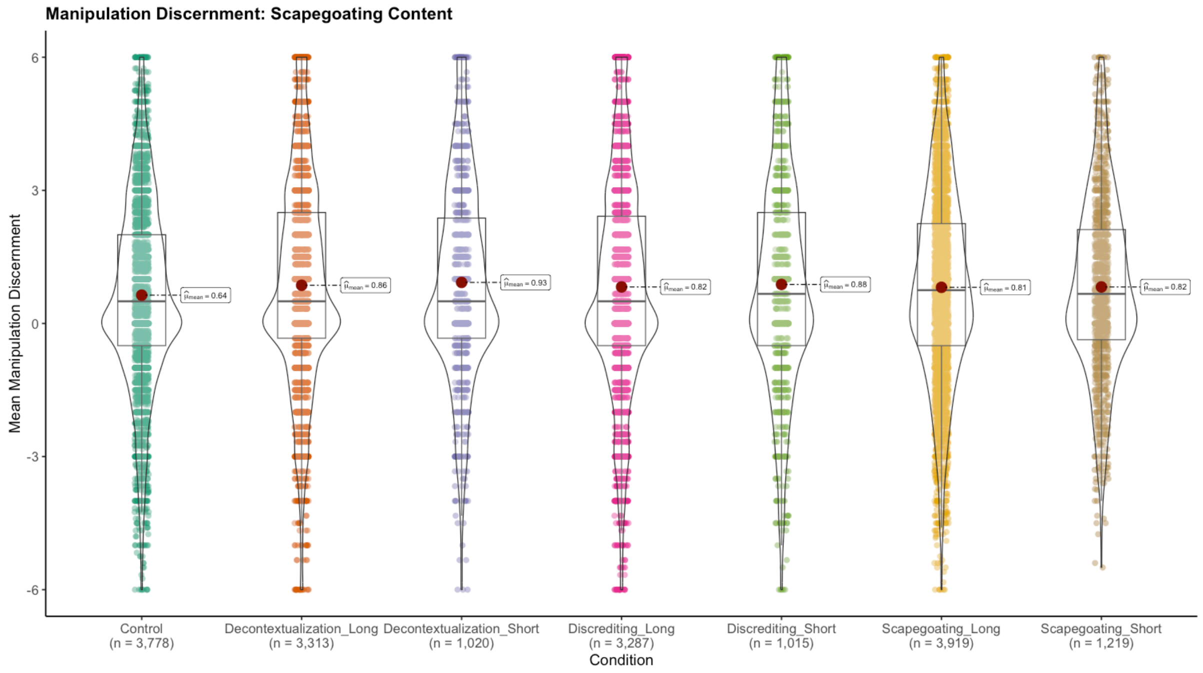

Manipulative decontextualization content

Figure S92

Violin Plot for the Mean Manipulativeness Assessments of Manipulative Decontextualization Content between Conditions.

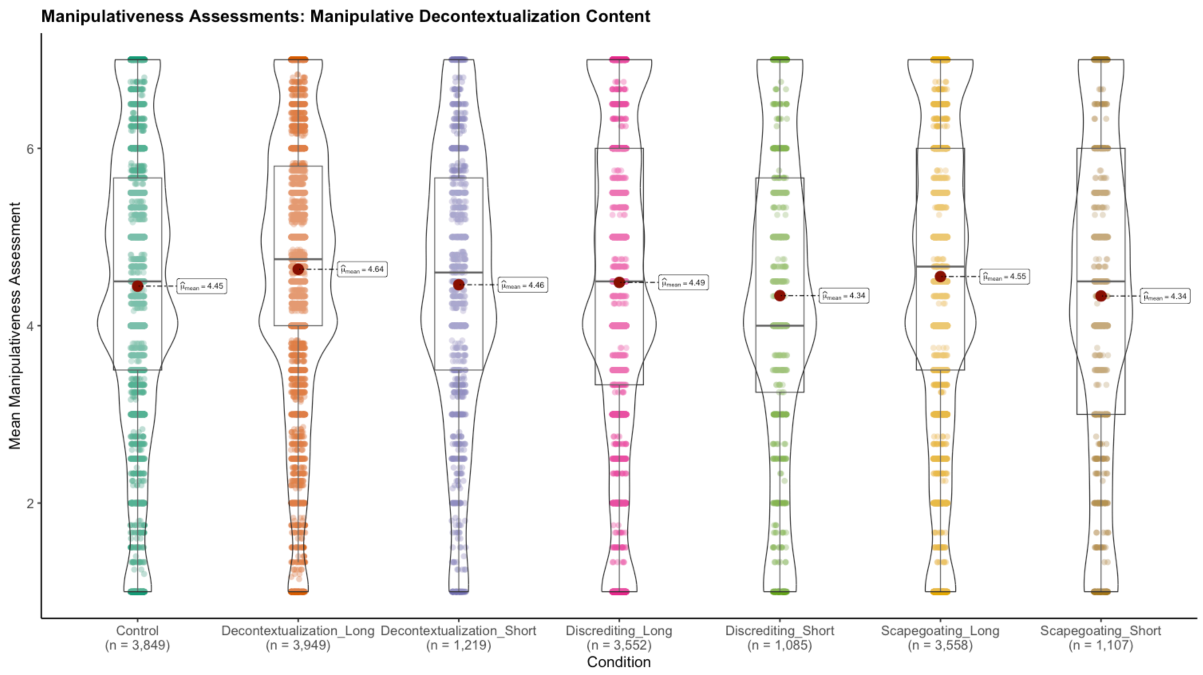

Non-manipulative decontextualization content

Figure S93

Violin Plot for the Mean Manipulativeness Assessments of Non-Manipulative Decontextualization Content between Conditions.

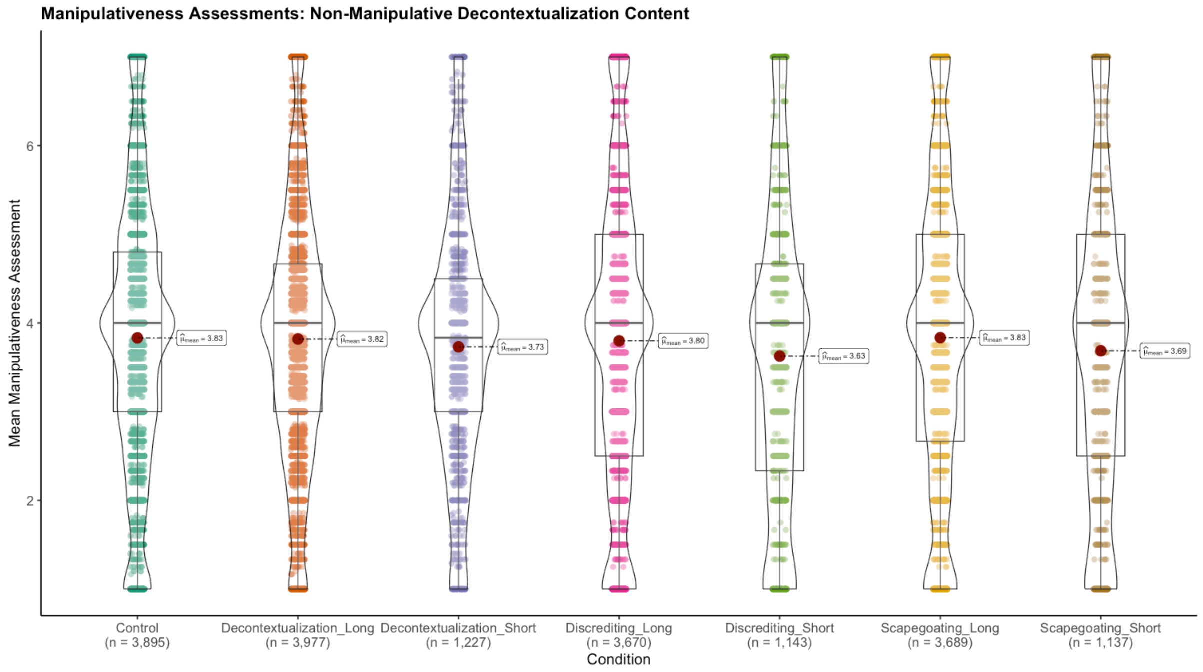

Manipulation discernment of Decontextualization content

Figure S94

Violin Plot for the Mean Manipulation Discernment of Decontextualization Content between Conditions.

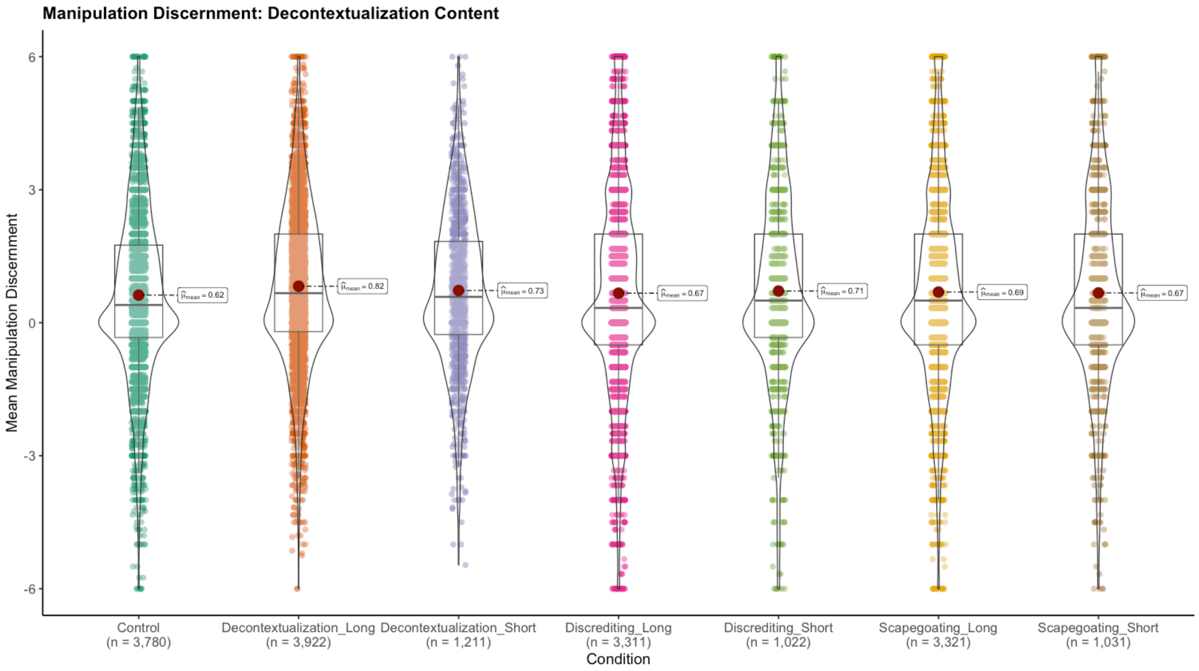

Manipulative Discrediting content

Figure S95

Violin Plot for the Mean Manipulativeness Assessments of Manipulative Discrediting Content between Conditions.

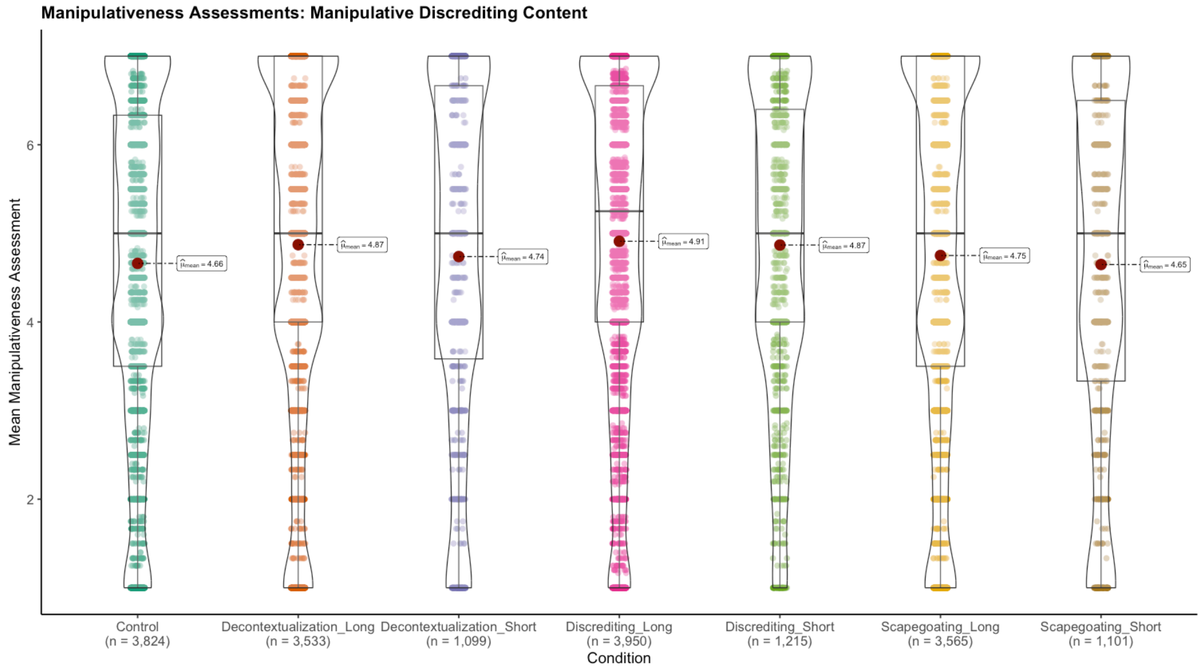

Non-manipulative Discrediting content

Figure S96

Violin Plot for the Mean Manipulativeness Assessments of Non-Manipulative Discrediting Content between Conditions.

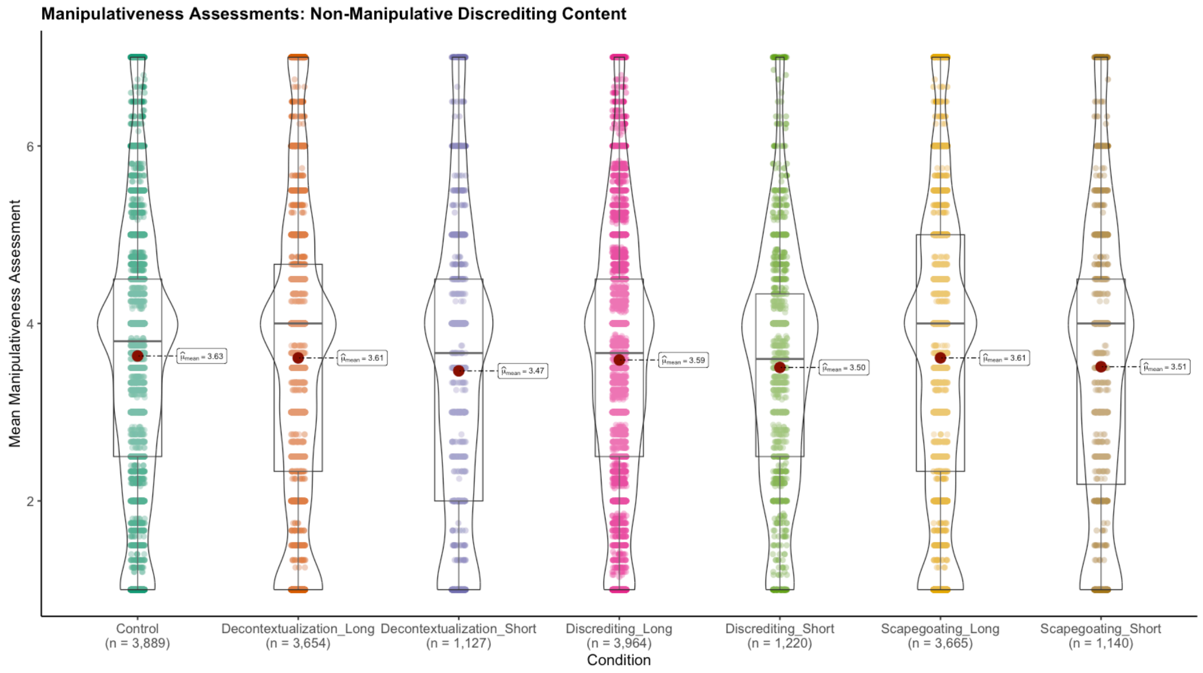

Manipulation discernment of Discrediting content

Figure S97

Violin Plot for the Mean Manipulation Discernment of Discrediting Content between Conditions.

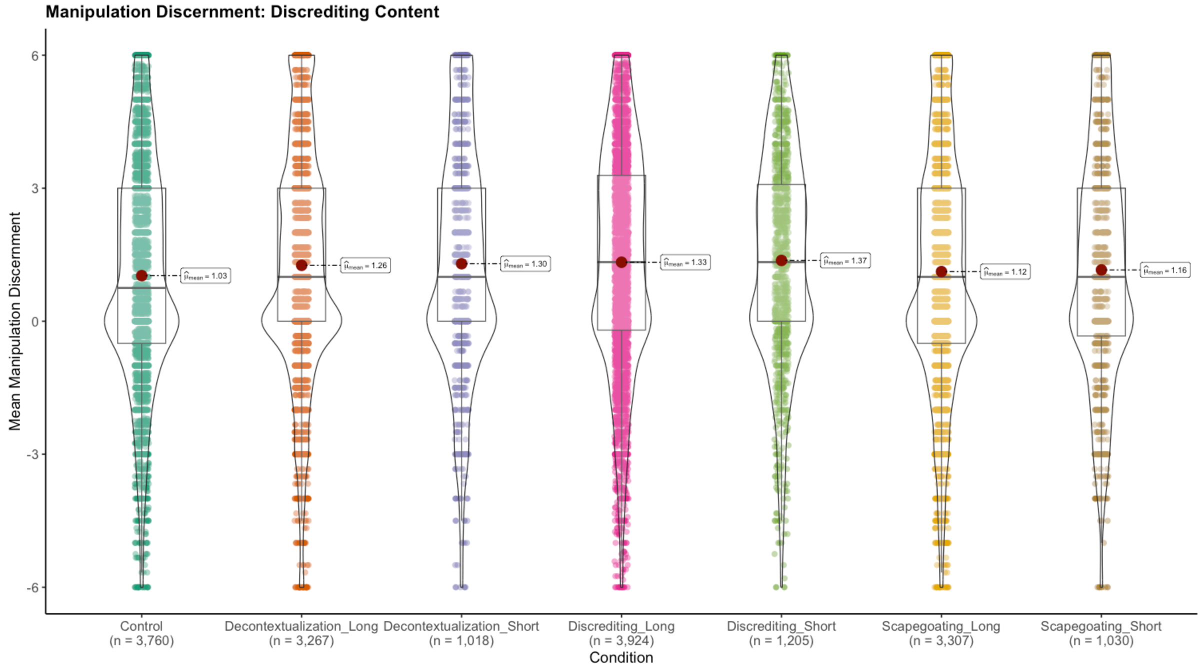

Technique recognition

Manipulative Scapegoating content

Figure S98

Violin Plot for the Mean Technique Recognition of Manipulative Scapegoating Content between Conditions.

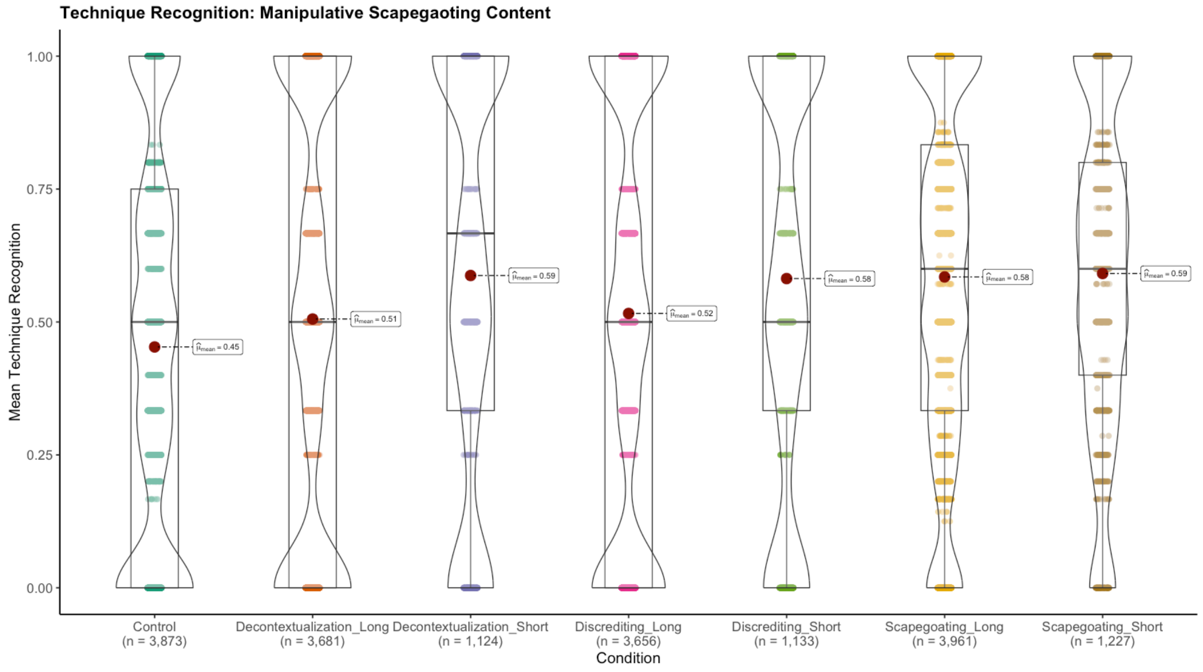

Non-manipulative Scapegoating content

Figure S99

Violin Plot for the Mean Technique Recognition of Non-Manipulative Scapegoating Content between Conditions.

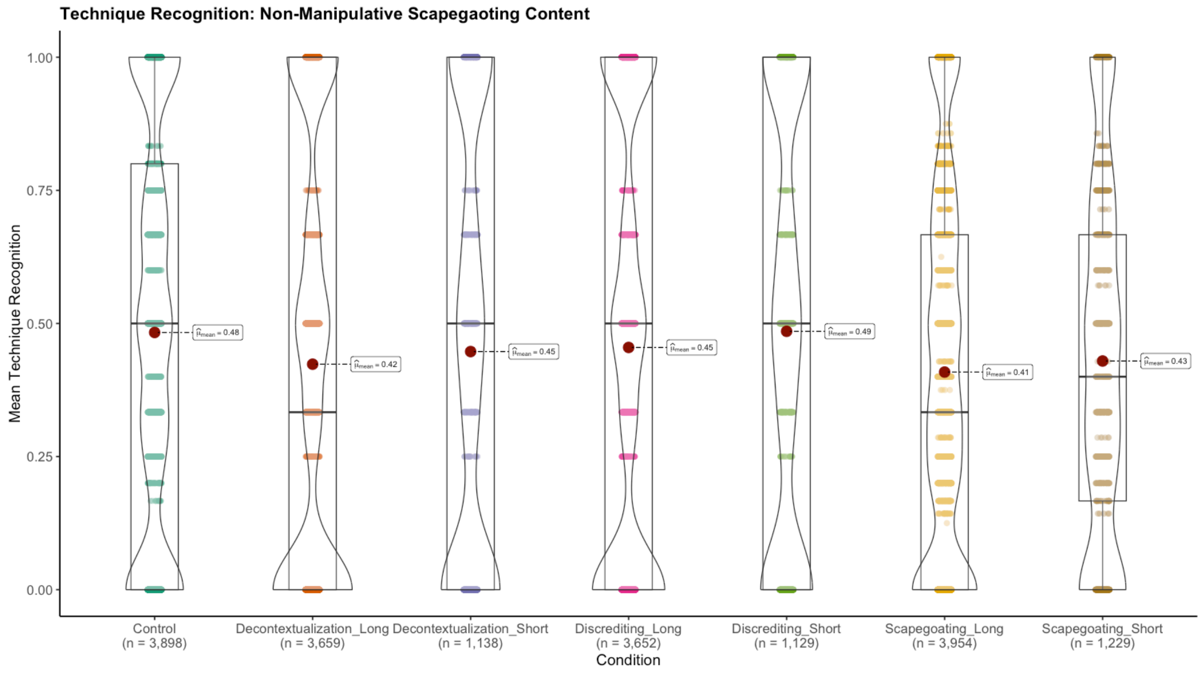

Technique discernment of Scapegoating content

Figure S100

Violin Plot for the Mean Technique Discernent of Scapegoating Content between Conditions.

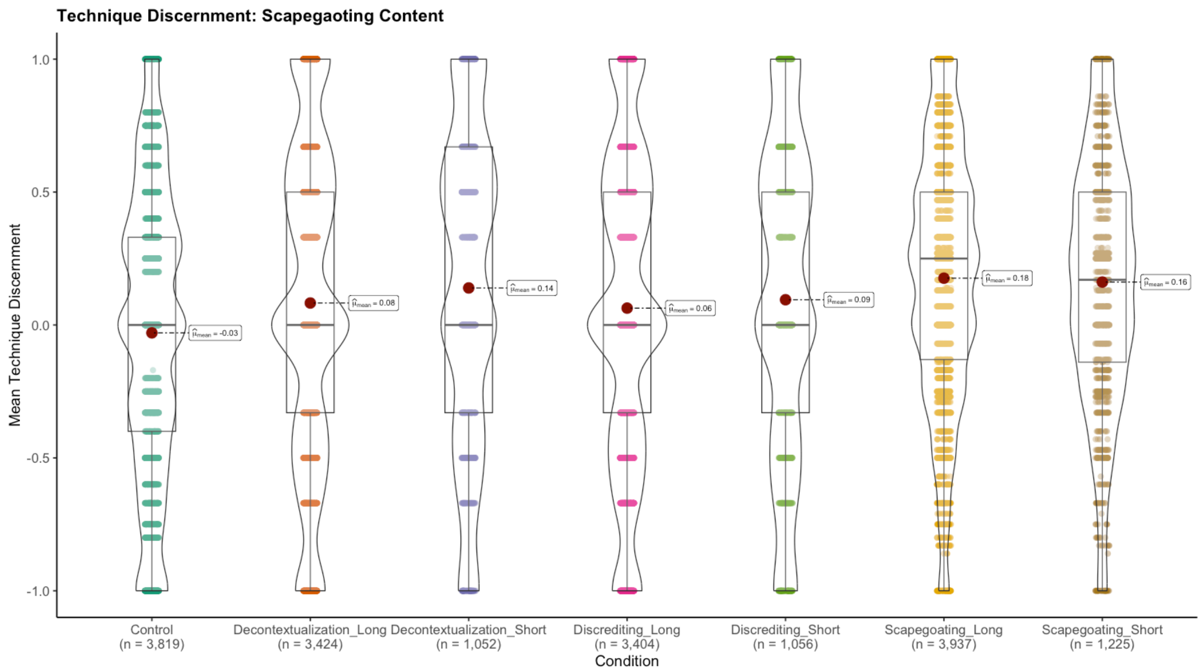

Manipulative Decontextualization content

Figure S101

Violin Plot for the Mean Technique Recognition of Manipulative Decontextualization Content between Conditions.

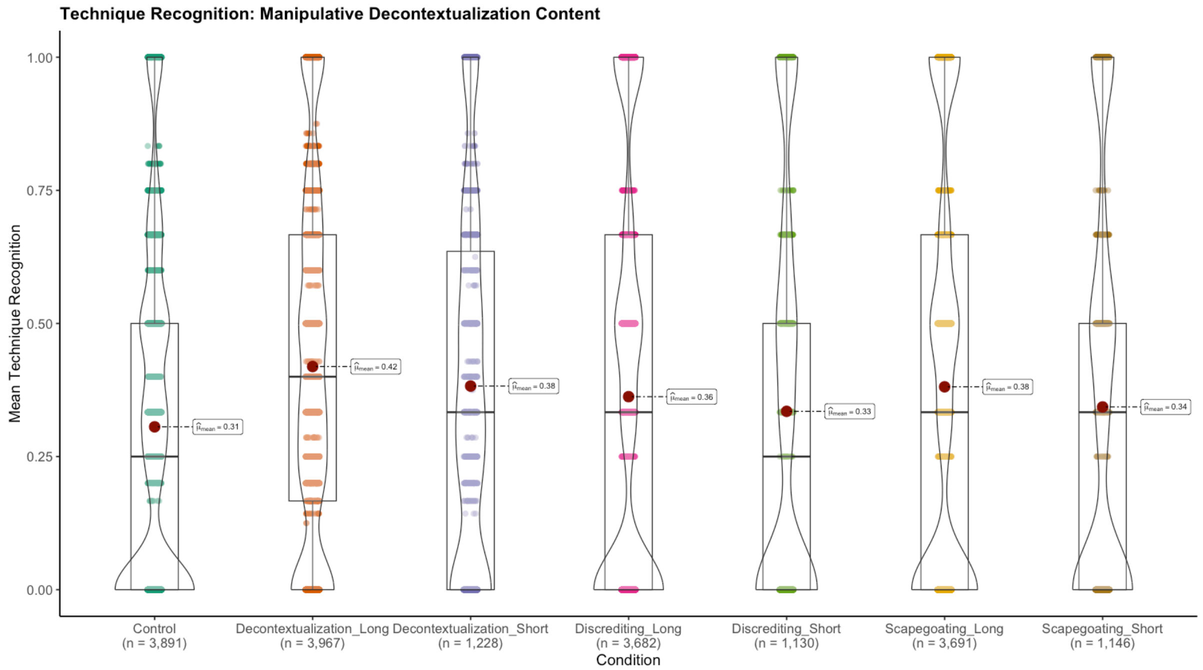

Non-manipulative Decontextualization content

Figure S102

Violin Plot for the Mean Technique Recognition of Non-Manipulative Decontextualization Content between Conditions.

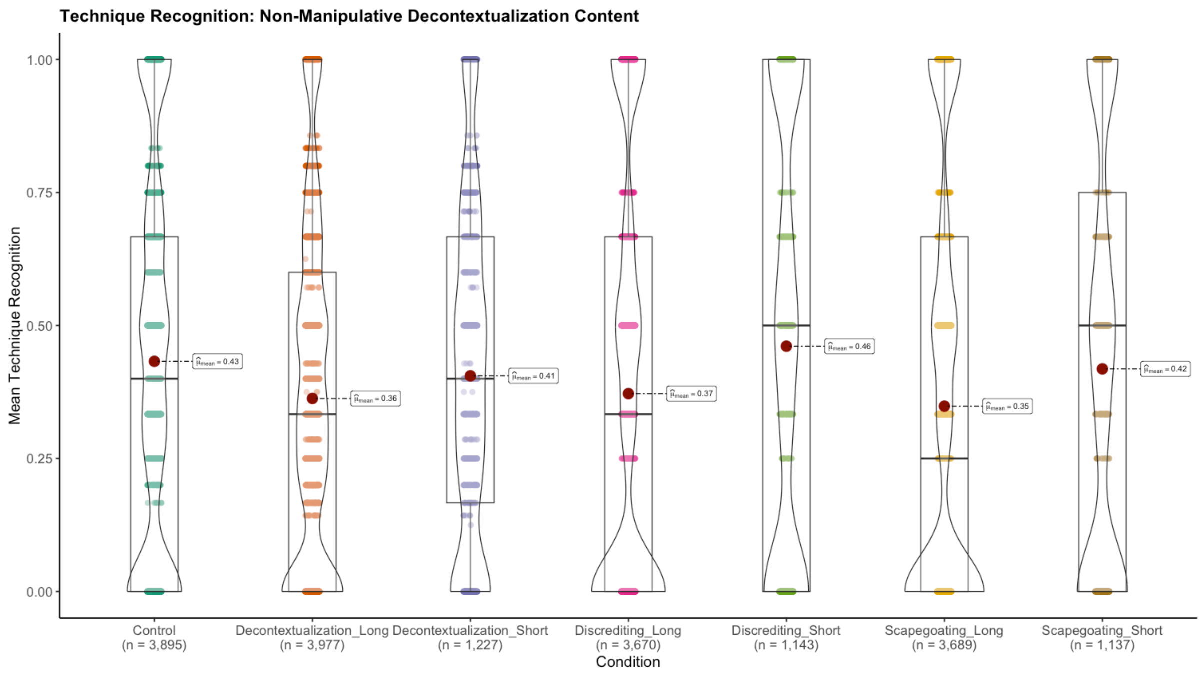

Technique discernment of Decontextualization content

Figure S103

Violin Plot for the Mean Technique Discernment of Decontextualization Content between Conditions.

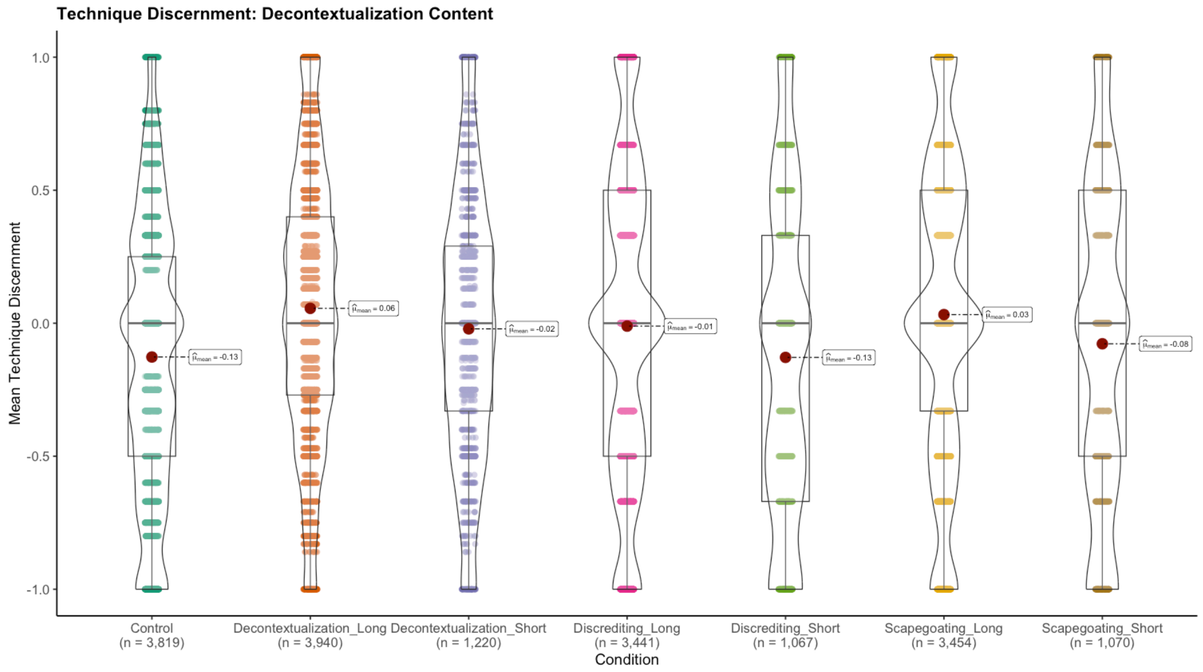

Manipulative Discrediting content

Figure S104

Violin Plot for the Mean Technique Recognition of Manipulative Discrediting Content between Conditions.

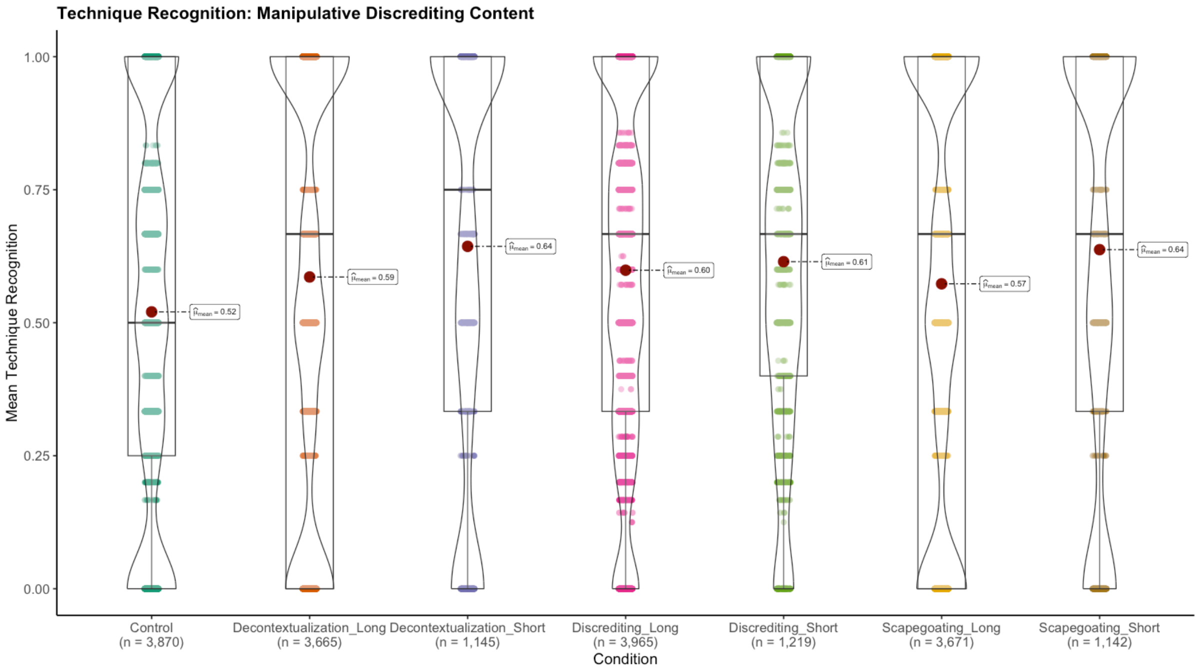

Non-manipulative Discrediting content

Figure S105

Violin Plot for the Mean Technique Recognition of Non-Manipulative Discrediting Content between Conditions.

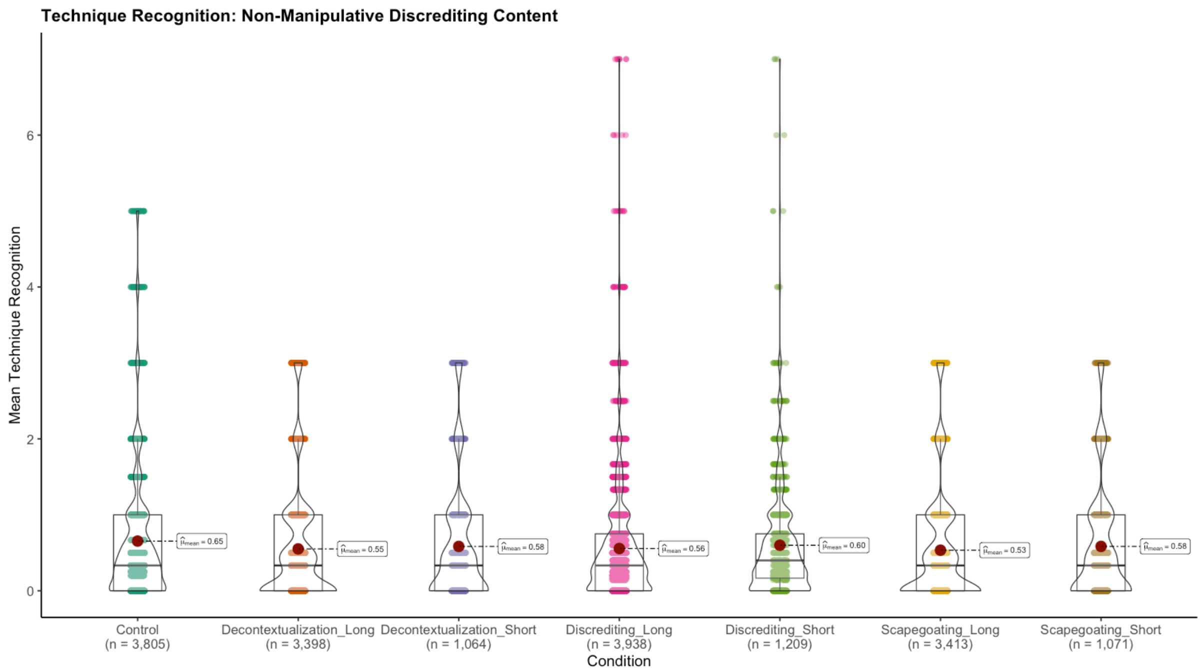

Manipulation discernment of Discrediting content

Figure S106

Violin Plot for the Mean Technique Discernment of Discrediting Content between Conditions.

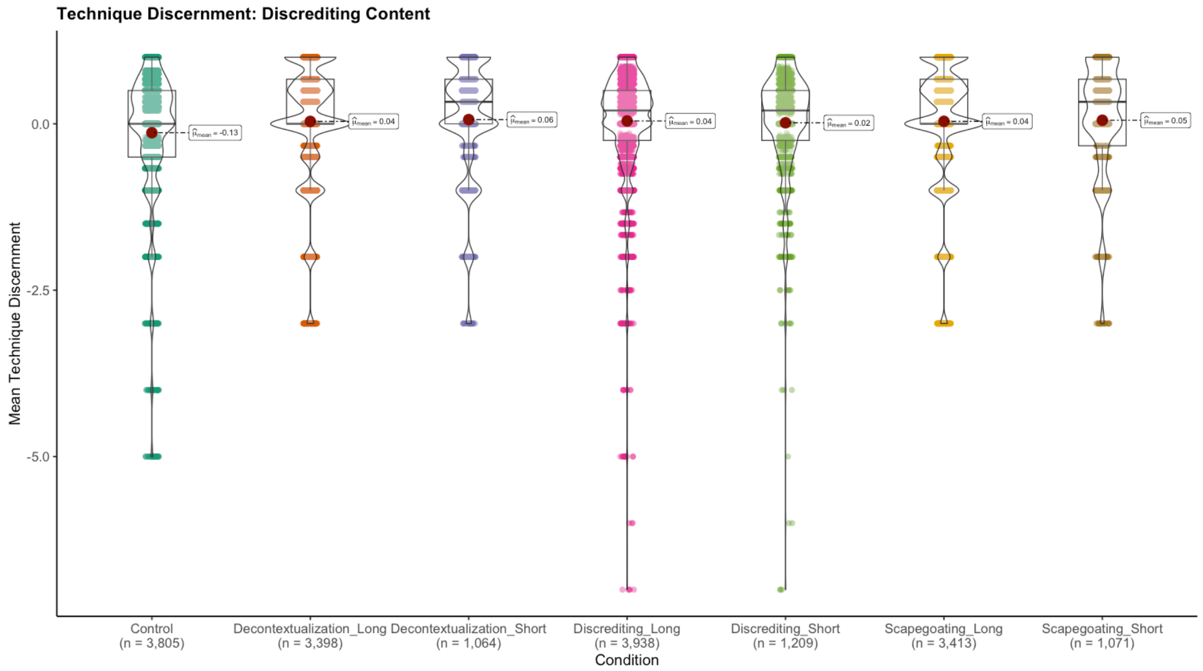

Sharing decisions

Manipulative Scapegoating content

Figure S107

Violin Plot for the Mean Willingness to Share Manipulative Scapegoating Content between Conditions.

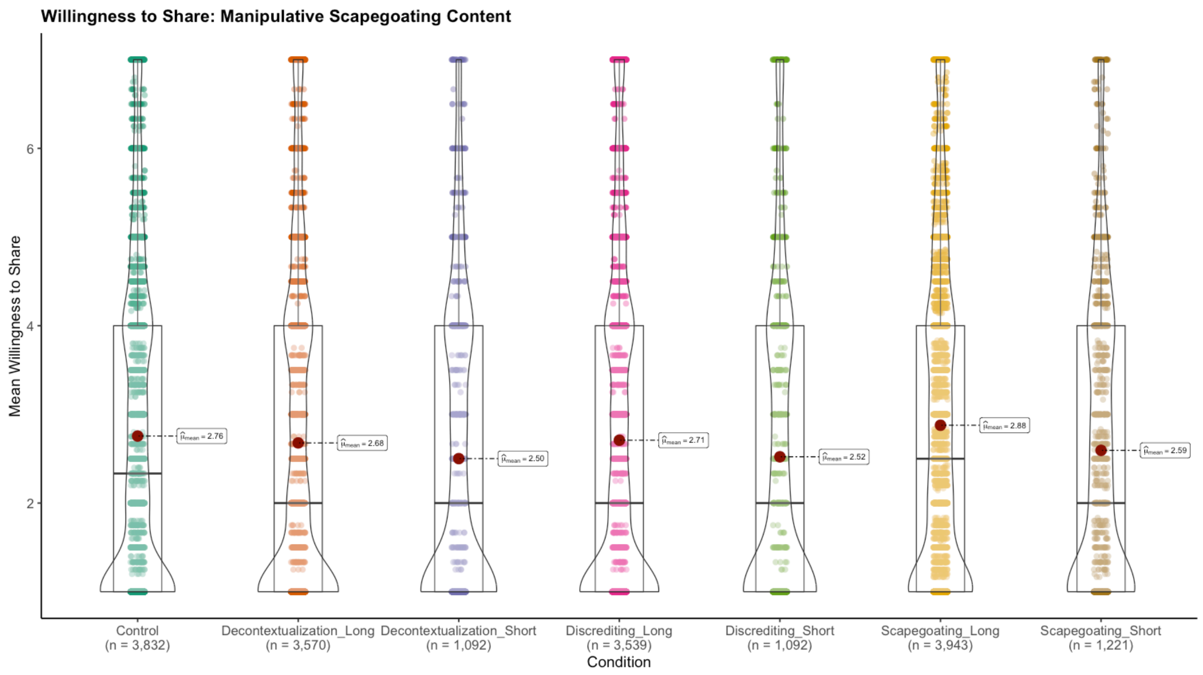

Non-manipulative Scapegoating content

Figure S108

Violin Plot for the Mean Willingness to Share Non-Manipulative Scapegoating Content between Conditions.

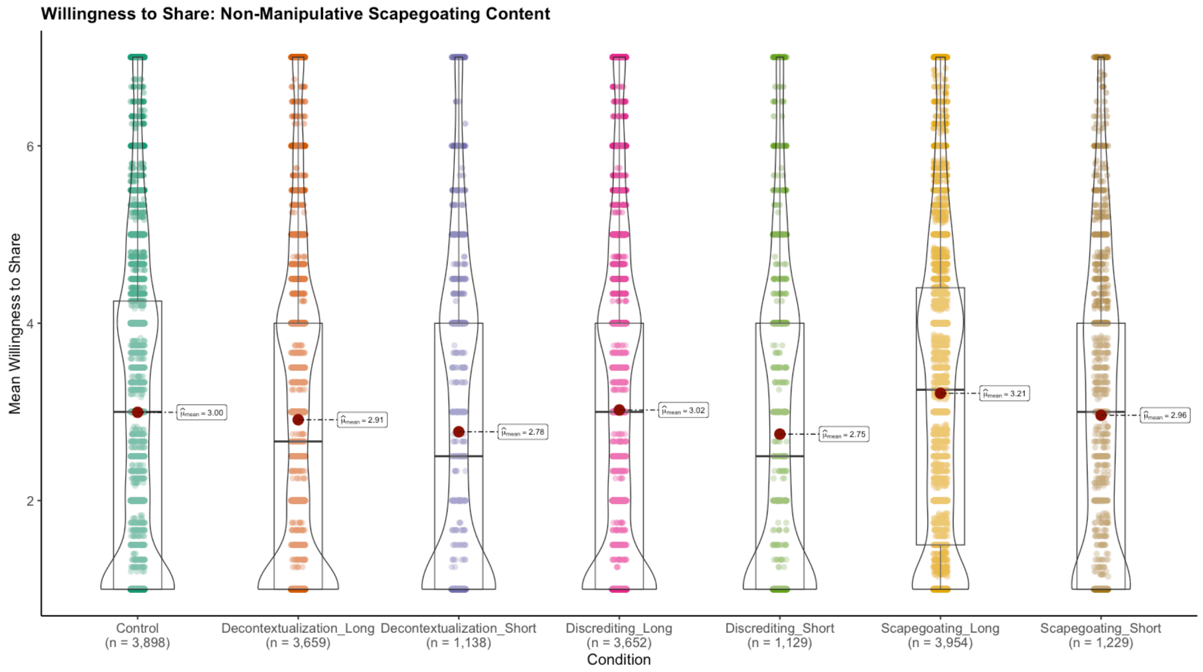

Sharing discernment of Scapegoating content

Figure S109

Violin Plot for the Mean Sharing Discernment for Scapegoating Content between Conditions.

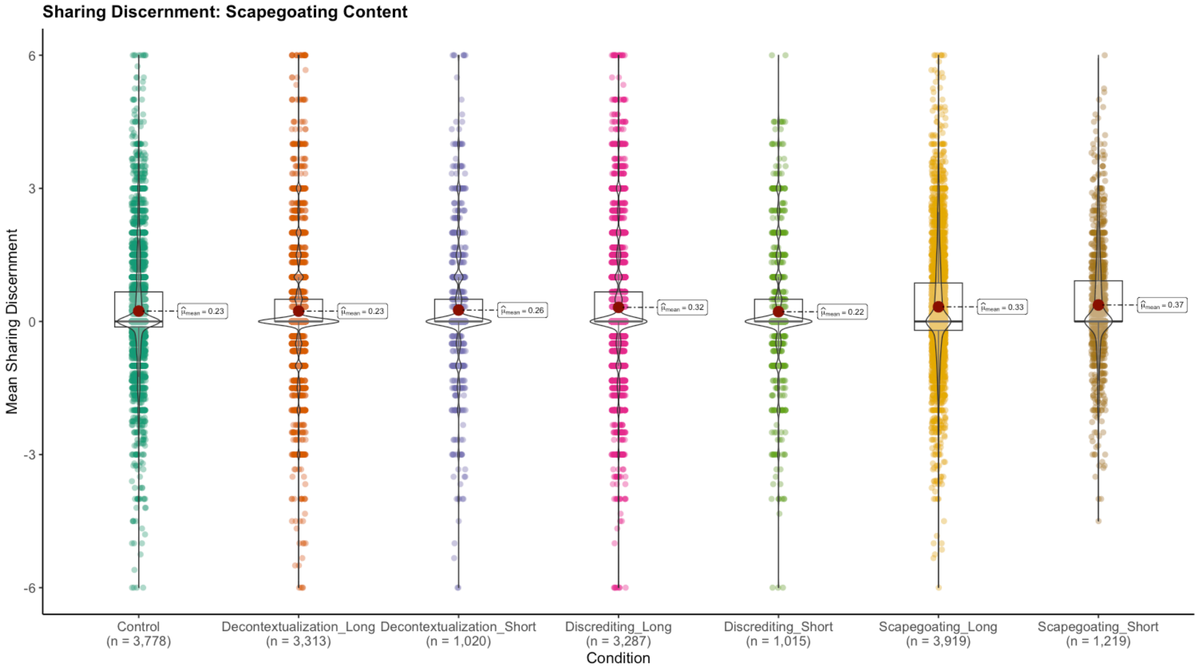

Manipulative Decontextualization content

Figure S110

Violin Plot for the Mean Willingness to Share Manipulative Decontextualization Content between Conditions.

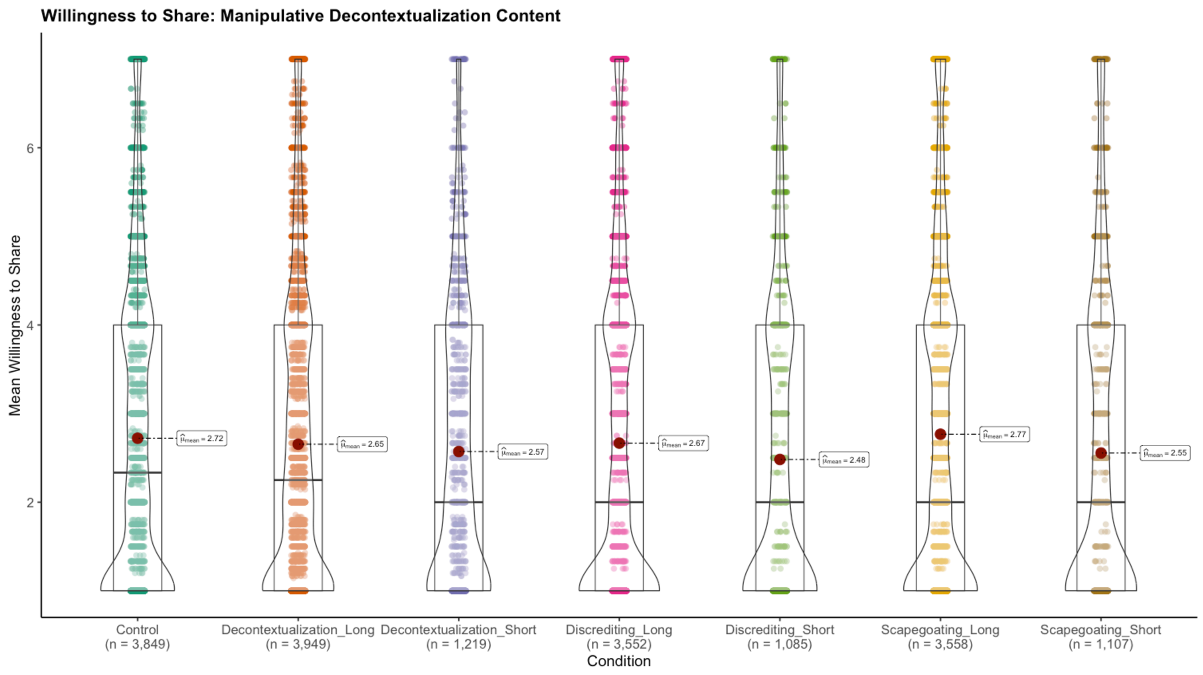

Non-manipulative Decontextualization content

Figure S111

Violin Plot for the Mean Willingness to Share Non-Manipulative Decontextualization Content between Conditions.

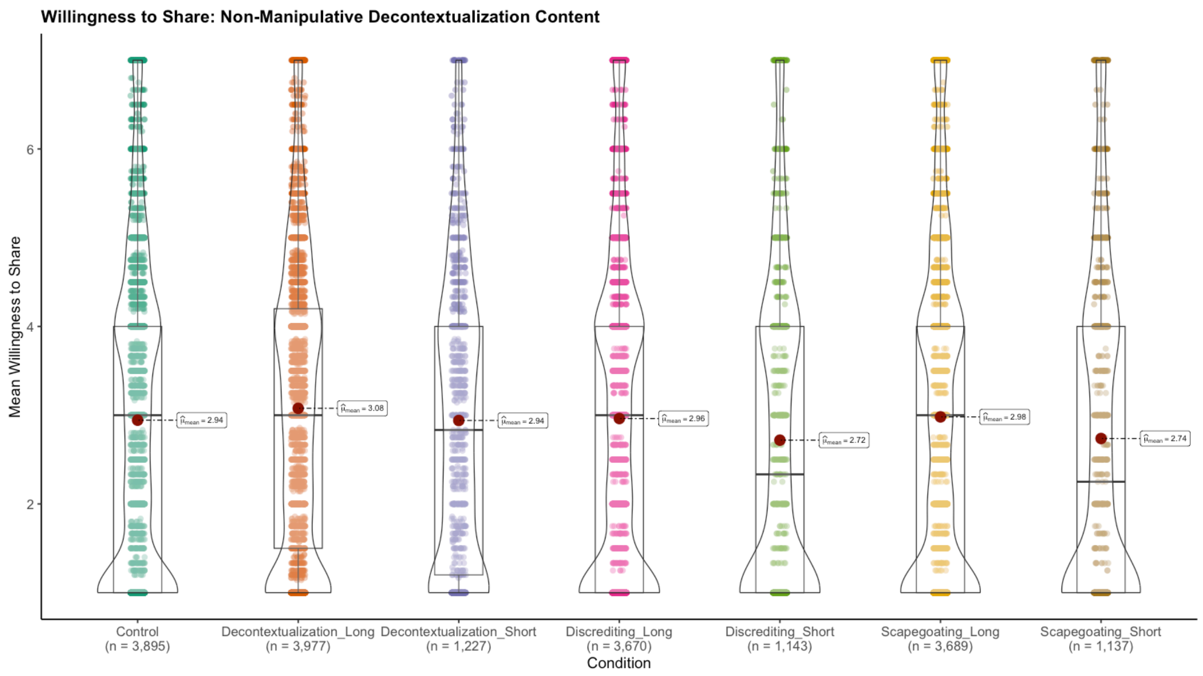

Sharing discernment of Decontextualization content

Figure S112

Violin Plot for the Mean Sharing Discernment for Decontextualization Content between Conditions.

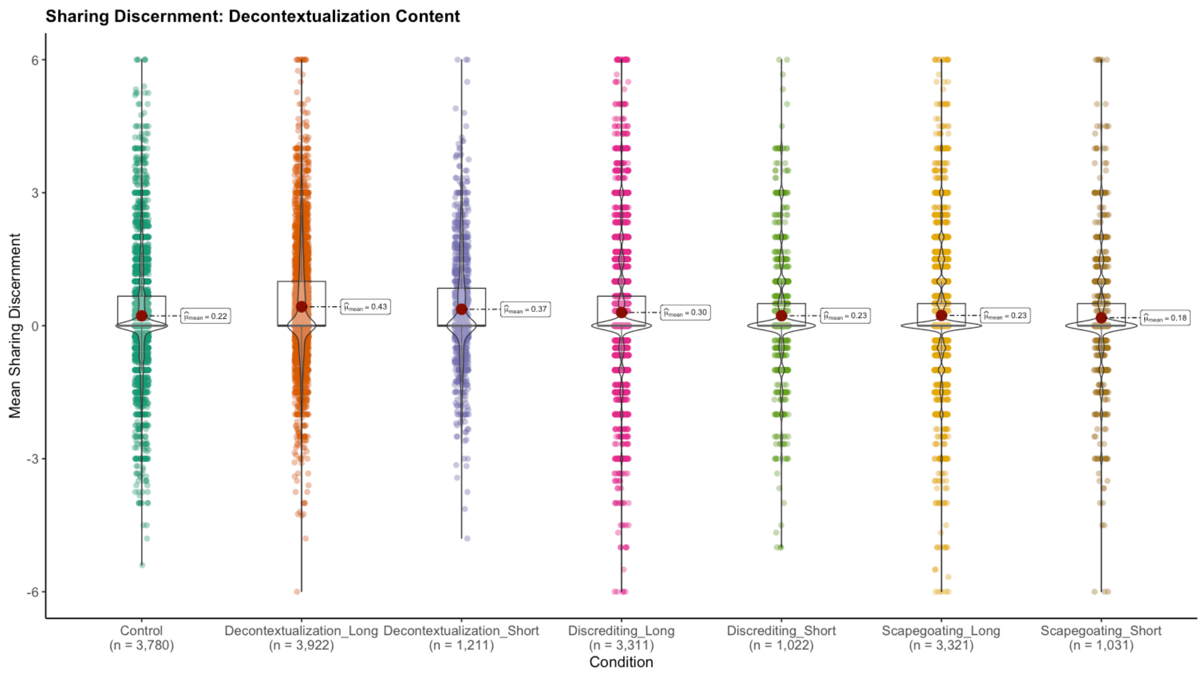

Manipulative Discrediting content

Figure S113

Violin Plot for the Mean Willingness to Share Manipulative Discrediting Content between Conditions.

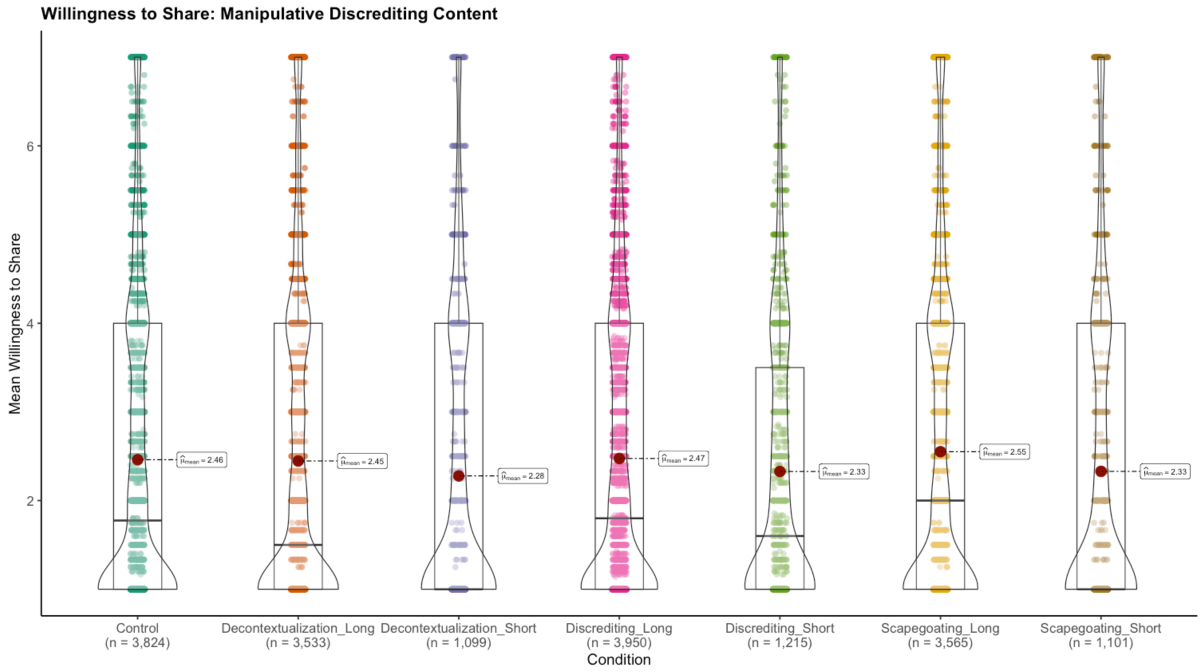

Non-manipulative Discrediting content

Figure S114

Violin Plot for the Mean Willingness to Share Non-Manipulative Discrediting Content between Conditions.

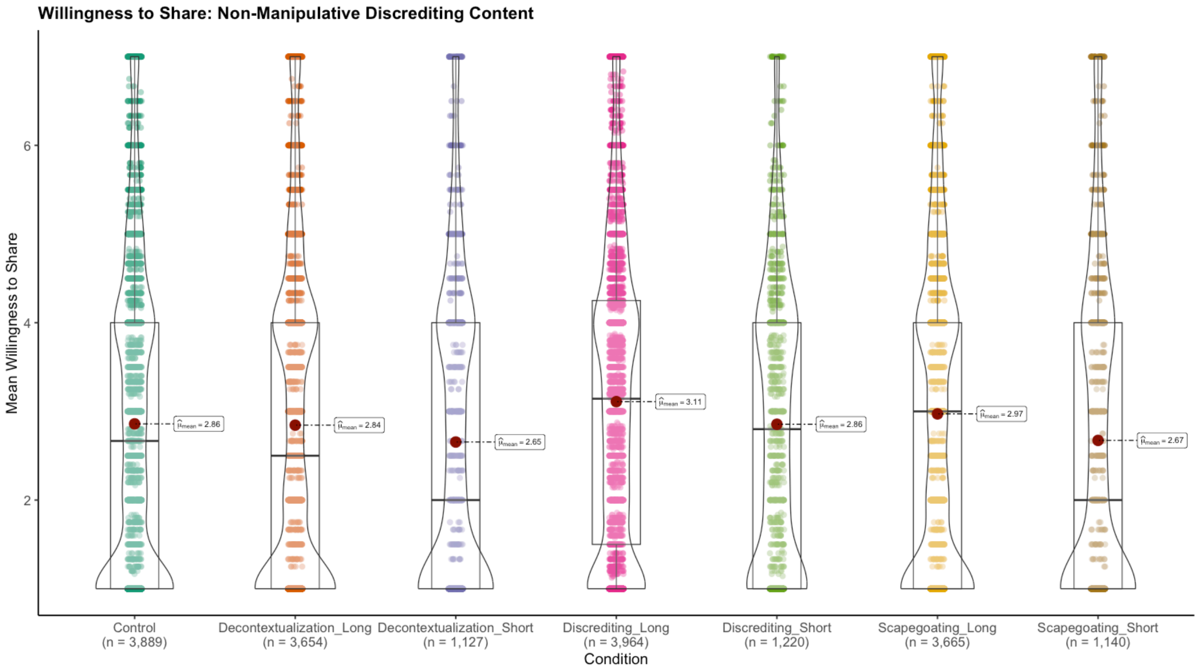

Sharing discernment of Discrediting content

Figure S115

Violin Plot for the Mean Sharing Discernment for Discrediting Content between Conditions.

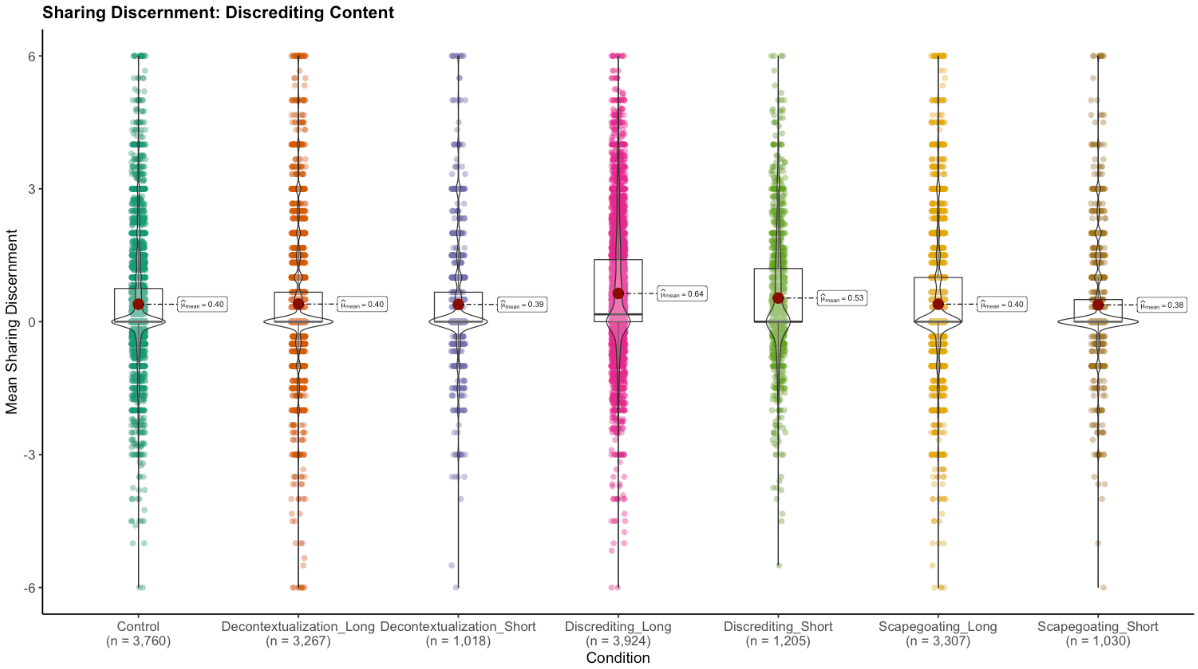

Confidence in detecting manipulation

Figure S116

Violin Plot for the Mean Confidence in Detecting Manipulation between Conditions.

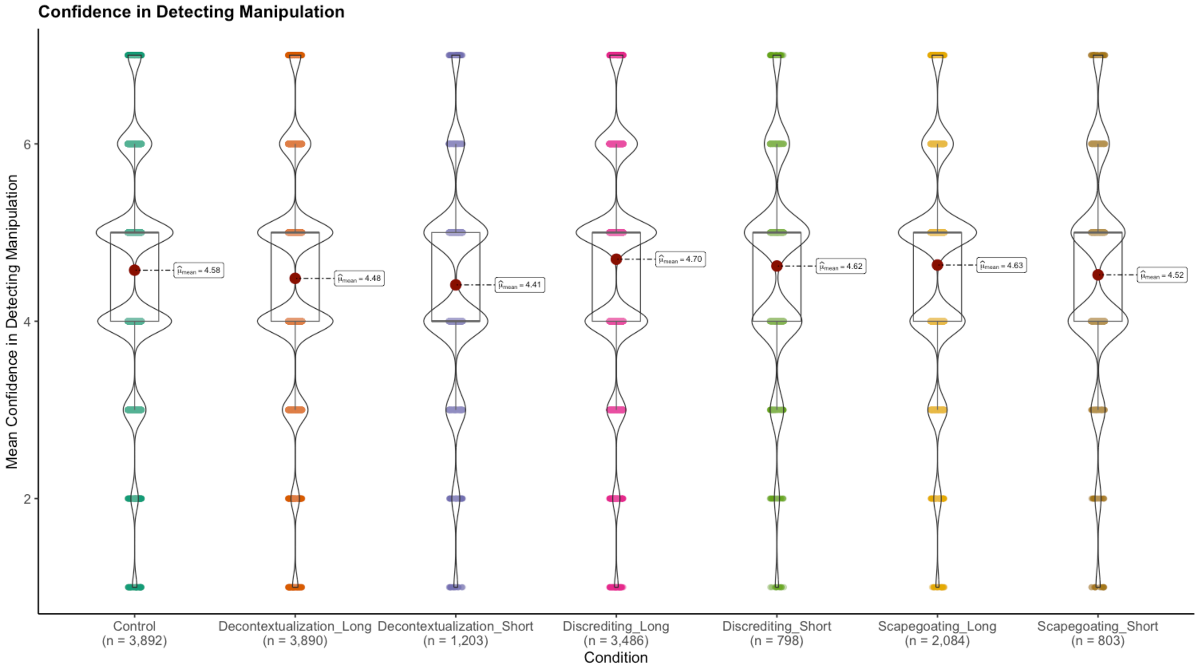

Section 9: Skepticism and Naïveté

Manipulativeness assessments

For the continuous manipulativeness assessment measures, the long scapegoating video (vs. control) significantly reduced Naïveté of the scapegoating content, the long

decontextualization video (vs. control) significantly reduced Naïveté of the decontextualization and discrediting content, and the long discrediting video (vs. control) significantly reduced Naïveté of the discrediting content (see Figure S117). All other effects were non-significant.

Figure S117

Forest Plot of the Unstadardized Beta Coefficients for the Effects of all Videos on all Continuous Assessment Skepticism and Naïveté outcomes.

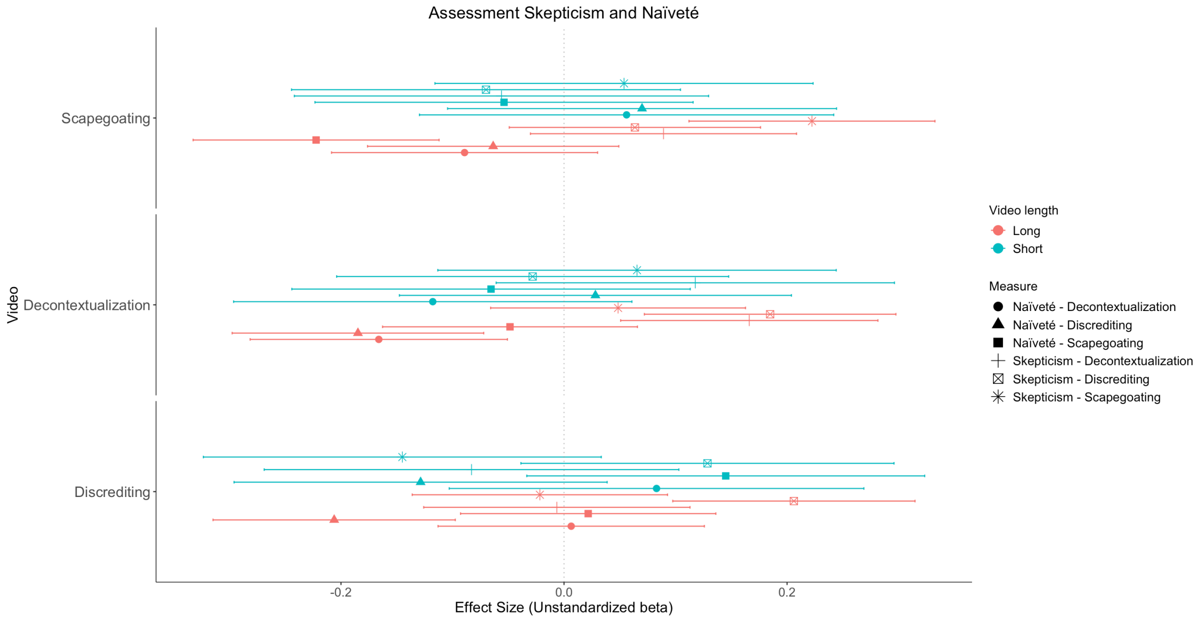

Technique recognition

All videos (vs. control), regardless of length, significantly increased technique skepticism and decreased technique Naïveté (see Figure S118).

Figure S118

Forest Plot of the Unstadardized Beta Coefficients for the Effects of all Videos on all Technique Skepticism and Naïveté outcomes.

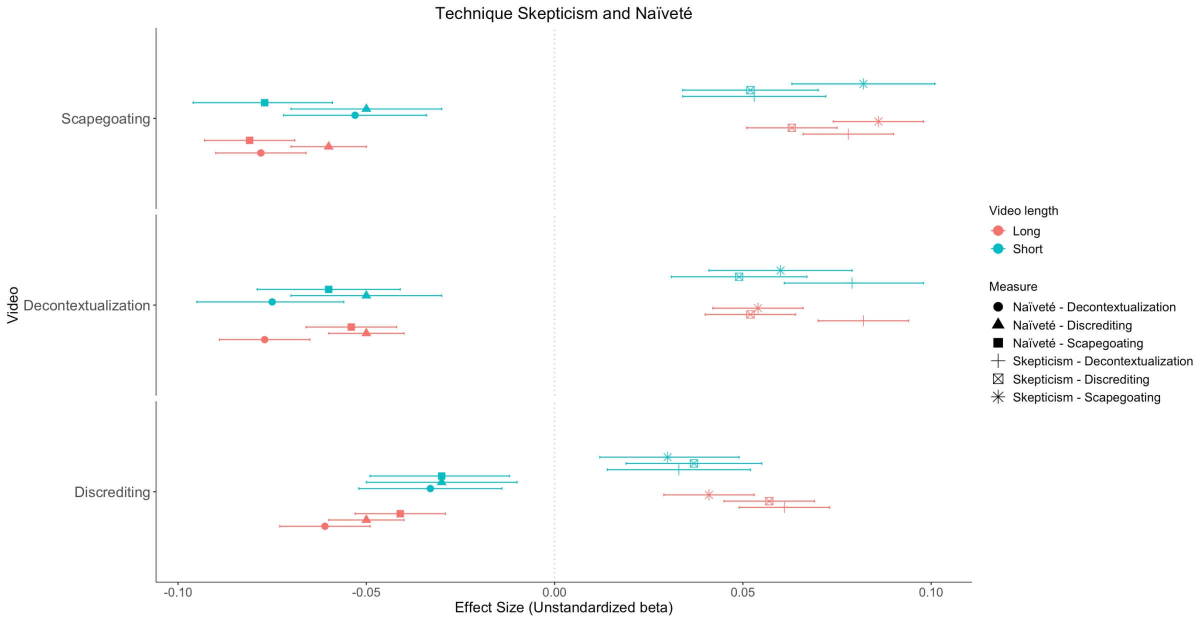
Technique Skepticism and Naïveté
Video
Scapegoating
Decontextualization
Discrediting
-0.10
-0.05
0.00
0.05
0.10
Effect Size (Unstandardized beta)
Video length
Long
Short
Measure
Naïveté - Decontextualization
Naïveté - Discrediting
Naïveté - Scapegoating
Skepticism - Decontextualization
Skepticism - Discrediting
Skepticism - Scapegoating
